# Catalytic Asymmetric (*ene*–*endo*)‑Carbonyl–Ene Type Cyclizations

**DOI:** 10.1021/jacs.5c11553

**Published:** 2025-09-10

**Authors:** Lixia Shi, Nobuya Tsuji, Chendan Zhu, Markus Leutzsch, Joyce A. A. Grimm, Benjamin List

**Affiliations:** 1 Max-Planck-Institut für Kohlenforschung, Kaiser-Wilhelm-Platz 1, 45470 Mülheim an der Ruhr, Germany; 2 Institute for Chemical Reaction Design and Discovery (ICReDD), 12810 Hokkaido University, N21 W10, Kita-ku, Sapporo 001-0021, Japan

## Abstract

The carbonyl–ene reaction between aldehydes and olefins constitutes a perfectly atom economic approach to homoallylic alcohols with concomitant C–C bond formation. However, the scope of catalytic asymmetric intermolecular versions is currently limited to activated substrates, while of the two intramolecular types only catalytic asymmetric (*ene*–*exo*)-carbonyl–ene type cyclizations can be considered mature. The corresponding (*ene*–*endo*)-cyclizations would arguably find equal utility in chemical synthesis but remain underdeveloped. Herein, we report an efficient regio- and enantioselective catalytic asymmetric (*ene*–*endo*)-carbonyl–ene type cyclization of unbiased alkenyl aldehydes using a strong and confined IDPi Brønsted acid catalyst. High enantioselectivities of up to 98:2 e.r. were obtained for a range of homoallylic alcohols.

## Graphical abstract

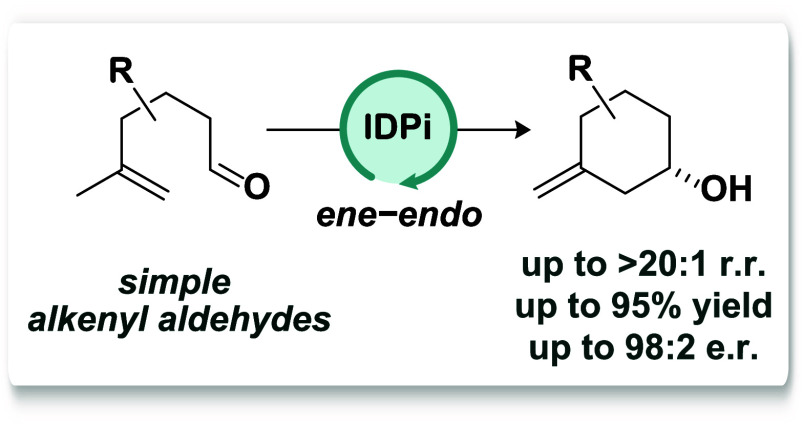

Among the allylations of aldehydes to homoallylic alcohols, the carbonyl–ene reaction clearly constitutes the most direct and atom economic approach.^1,2^ As a consequence, substantial effort has been devoted to the development of catalytic asymmetric variants.^3–6^ In essence, there are three different types of such carbonyl–ene reactions: (1) intermolecular ones, and (2) intramolecular (*ene*–*exo*) (“type I”) and (3) (*ene*–*endo*) (“type II”)-cyclizations (Figure 1A). When the latter two processes occur in a stepwise, cationic fashion, they are also sometimes referred to as Prins cyclizations. While intermolecular versions are challenging and have previously only been reported with activated, typically glyoxylate-type aldehydes or activated olefins,^7–20^ (*ene*–*exo*)-carbonyl–ene type cyclizations are well-documented with chiral organic and metal-based acid catalysts.^21–31^ In contrast, catalytic asymmetric (*ene*–*endo*)-cyclizations are rare though would deliver useful cyclic homoallylic alcohols with an exocyclic olefin.^22,24,32–34^ We report here a direct asymmetric (*ene*–*endo*)-carbonyl–ene type cyclization of simple alkenyl aldehydes. We have identified a broadly applicable, strong, and confined imidodiphosphorimidate (IDPi) catalyst that cyclizes a variety of unbiased substrates with excellent selectivity (Figure 1B).

**Figure 1:**
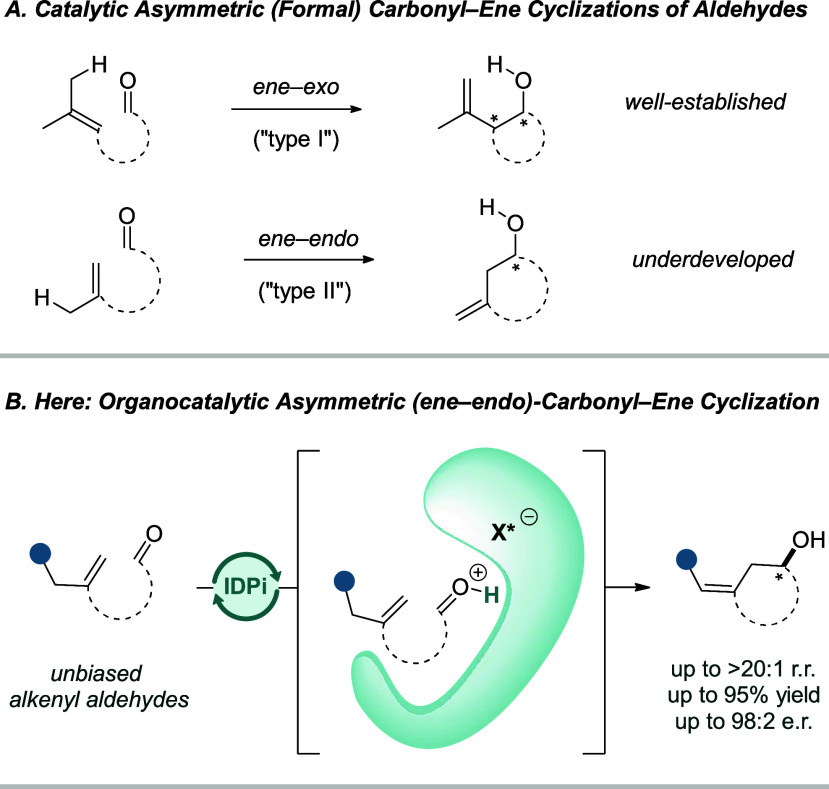
(A) Catalytic asymmetric (formal) carbonyl–ene cyclizations of aldehydes. (B) A highly acidic and confined acid enables a catalytic asymmetric (*ene*–*endo*)-carbonyl–ene type cyclization of simple alkenyl aldehydes. Regiomeric ratio (r.r.) is the ratio of the *exo* vs *endo* isomer.

Since Yamamoto et al. in 1986 reported the first example of a catalytic enantioselective (*ene*–*exo*)-carbonyl–ene cyclization of 3-methylcitronellal, using a stoichiometric amount of a chiral BINOL-Zn­(II) catalyst,^21^ various chiral Lewis acids, such as BINOL-Ti­(IV),^22^ bisoxazoline-Cu­(II),^23^ Schiff base-Cr­(III)^24,25^ or BINOL-Al­(III)^26,27^ have been described as catalysts of related reactions. Organocatalytic versions have also been reported in recent years, often with exciting and complementary results. For example, in 2015, our group developed an organocatalytic enantioselective five-membered-ring-forming (*ene*–*exo*)-carbonyl–ene type cyclization using a confined Brønsted acid imidodiphosphoric acid (IDP) catalyst.^28^ In 2021, the Jacobsen group reported the cooperative asymmetric catalysis by HCl and chiral dual-hydrogen-bond donors (HBDs) of highly enantioselective (*ene*–*exo*)-Prins cyclizations, to access six-membered ring homoallylic alcohols.^29^ More recently, our group achieved the efficient cyclization of α,β-unsaturated aldehydes using a highly fluorinated confined iminoimidodiphosphate (*i*IDP) Brønsted acid. This catalyst mediates the enantioselective cyclization of neral to the non-natural (1*R*,6*S*)-*trans*-isopiperitenol enantiomer, which in turn enables single step routes to menthol and cannabinoids.^31^ To our knowledge, only very few (*ene*–*endo*)-carbonyl–ene type cyclizations have previously been reported. Ziegler et al. reported the first enantioselective intramolecular (*ene*–*endo*)-carbonyl–ene reaction of a dialdehyde, employing a series of chiral Lewis acid catalysts to afford the corresponding cyclic product with modest enantioselectives (60:40 e.r. to 69:31 e.r.).^32^ While Mikami and co-workers demonstrated a BINOL-Ti­(IV)-catalyzed seven-membered (*ene*–*endo*)-cyclization of homoallylic ether, the reaction proceeded with only moderate reactivity and enantioselectivity.^22^ The Jacobsen group also disclosed a few examples of (*ene*–*endo*)-carbonyl–ene type cyclizations by applying a chiral Schiff base-Cr­(III) complex. These examples were limited to Thorpe–Ingold-type geminally disubstituted substrates.^24^ During the preparation of this article, the Ishihara group reported a highly enantio- and (*E*)-selective 6-*endo*–*trig* cyclization of 5-substituted 5-hexanals catalyzed by a Lewis-acid-assisted chiral Brønsted acid (LBA). This system seems to be limited to aryl-substituted substrates, while simple alkyl-substituted substrates remain underexplored.^34^ A general catalytic method for asymmetric (*ene*–*endo*)-carbonyl–ene type cyclizations of simple alkenyl aldehydes would clearly expand the synthetic arsenal and is the topic of this work. Our group has introduced a class of strong and chiral confined IDPi Brønsted acids and demonstrated their utility in a variety of asymmetric transformation of unactivated substrates.^35–43^ Because of their proven ability to provide a confined active site that readily accommodates cyclic, cationic transition states of various cyclizations, we expected that this class of catalysts could also enable highly regio- and stereoselective (*ene*–*endo*)-carbonyl–ene type cyclization of simple alkenyl aldehydes to enantioenriched homoallylic alcohols.

We initially evaluated the cyclization of 5-methylhex-5-enal (**1a**) as a model substrate in dichloromethane at 0 °C for 6 h using a variety of chiral Brønsted acid catalysts covering a broad p*K*_a_ range. The weaker phosphoric acid (CPA, p*K*_a_ ∼ 13 in MeCN)^44^ **S1** and IDP (p*K*_a_ ∼ 11.3 in MeCN)^35^ **S2** showed low reactivity, while the more acidic disulfonimide (DSI, p*K*_a_ ∼ 8.4 in MeCN)^35^ **S3** or *i*IDP (p*K*_a_ ∼ 9.0 in MeCN)^35^ **S4-5** resulted in full conversion of aldehyde **1a** into homoallylic alcohol **2a** with moderate yield and poor regio- and enantioselectivities (see Supporting Information for details). Speculating that the high reactivity and confinement of the IDPi (p*K*_a_ ∼ 4.5 to 2.0 in MeCN)^35^ would be beneficial for reactivity and stereocontrol, we also tested this catalyst class. Among our IDPi libraries, catalysts with sterically larger π-substituents in the 3,3′-positions turned out to be particularly promising in terms of enantioselectivity (Table 1, entries 1–6). Indeed, IDPi **7a**, bearing 2-pyrene substituents in the 3,3′-positions of the BINOL backbone and triflyl groups in the inner core, furnished product **2a** in a 9:1 ratio of *exo* and *endo* products and with a promising 94:6 e.r. for *exo*-**2a** (entry 6). Extending the perfluoroalkyl sulfonyl chain of the inner core, IDPi **7b** further increased the regio- (*exo*/*endo* = 94:6) and enantioselectivity (95:5 e.r.) (entry 7). Therefore, we chose IDPi **7b** for further optimizations regarding solvents and reaction temperature (entry 8–10). Gratifyingly, when the reaction was performed at –20 °C in CHCl_3_ with 1 mol% catalyst loading, a 70% yield of product **2a** was observed with excellent regio- (96:4) and enantioselectivity (98:2).

**Table 1:**
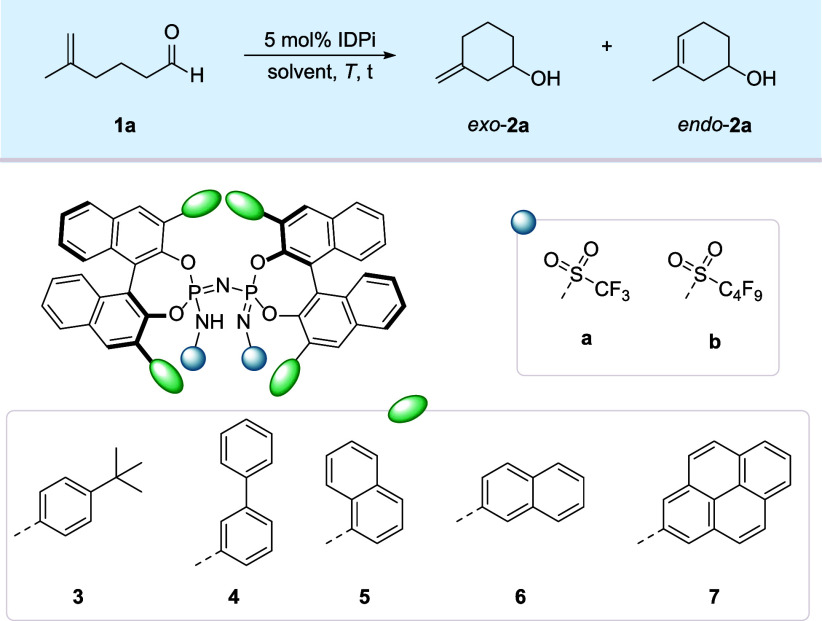
Reaction Development

entry^a^	IDPi	solvent	*T* (°C)	time (h)	yield (%)^b^	*exo*:*endo* [Table-fn t1fn2.a]	e.r.^c^
1	**3a**	DCM	0	6	85	87:13	58:42
2	**4a**	DCM	0	6	64	74:26	86.5:13.5
3	**5a**	DCM	0	6	78	86:14	89:11
4	**6a**	DCM	0	6	67	73:27	90.5:9.5
5	**6a** ^d^	DCM	0	6	63	93:7	91.5:8.5
6	**7a** ^d^	DCM	0	6	63	90:10	94:6
7	**7b** ^d^	DCM	0	6	55	94:6	95:5
8	**7b** ^d^	PhMe	0	6	49	94:6	94:6
9	**7b** ^d^	CHCl_3_	0	6	65	92:8	96:4
10	**7b** ^d^	CHCl_3_	–20	16	70	96:4	98:2

a Reactions were performed with 5-methylhex-5-enal **1a** (0.012 mmol, 1.0 equiv.) and catalysts (5 mol%) in DCM (0.06 M).

b Determined by ^1^H NMR analysis using mesitylene as internal standard.

c Determined by ^1^H NMR analysis.

d The enantioselectivity of ***exo*-** **2a** was determined by GC analysis.

e The catalyst loading was 1 mol%.

With optimized reaction conditions in hand, the scope and generality of the (*ene*–*endo*)-carbonyl–ene type cyclization was explored and the results are shown in Figure 2. Several 5-substituted 5-hexenals were investigated. Substituents with linear (**1a**–**c**), branched (**1d**), terminal alkene (**1e**, **f**) and terminal aryl (**1g**, **1h**) aliphatic groups were well tolerated, furnishing the corresponding homoallylic alcohols **2** in good yields with high enantioselectivities up to 98:2. While an acyclic branched aliphatic substituent in the 5-position was well tolerated, moderate enantiomeric ratios were obtained in the reactions of substrates bearing cyclic substituents close to the olefin such as cyclopentyl (**1j**), cyclohexyl (**1k**), isobutyl (**1l**), and methylcyclohexyl (**1m**). Notably, functionalized aldehydes possessing ether (**1n**), thiophene (**1o**), hydroxyl (**1p**), ester (**1q**), or bromide (**1r**) functional groups were smoothly transformed, delivering the corresponding products in moderate to good yields and excellent enantiomeric ratios. High (*E*)-stereoselectivity was observed across all of the above 5-substituted 5-hexenals, with the sole exception of aldehyde **1n** bearing an ether group, which exhibited a diminished *E*/*Z* ratio of 7.7:1. The regiomeric ratio of product **2n** was also significantly reduced to 4.2:1 in this special case. We speculate that an additional Lewis base could interact with the cationic intermediate in a different way, thereby diminishing the selectivities. Moreover, our methodology proved to be effective for seven-membered (*ene*–*endo*)-cyclizations of simple alkenyl aldehydes, delivering products **2s** and **2t** in moderate to good yields with excellent enantiomeric ratios. 3,3-Dimethyl substituted aldehyde **1u** was also efficiently converted, furnishing the corresponding product **2u** in 75% yield and 97.5:2.5 e.r. Furthermore, catalyst **7b** gave aromatic homoallylic alcohol **2v** in reasonable yield with an excellent enantiomeric ratio. Regioselectivity and enantioselectivity were somewhat reduced in reactions involving trisubstituted or tetrasubstituted alkenyl aldehydes, α-disubstituted aldehydes, and a ketone (6-methylhept-6-en-2-one) (see Supporting Information for details). When we attempted to employ a simple alkenyl aldehyde that would yield a five-membered product in an (*ene*–*endo*)-cyclization, no reaction was observed (see Supporting Information for details).

**Figure 2:**
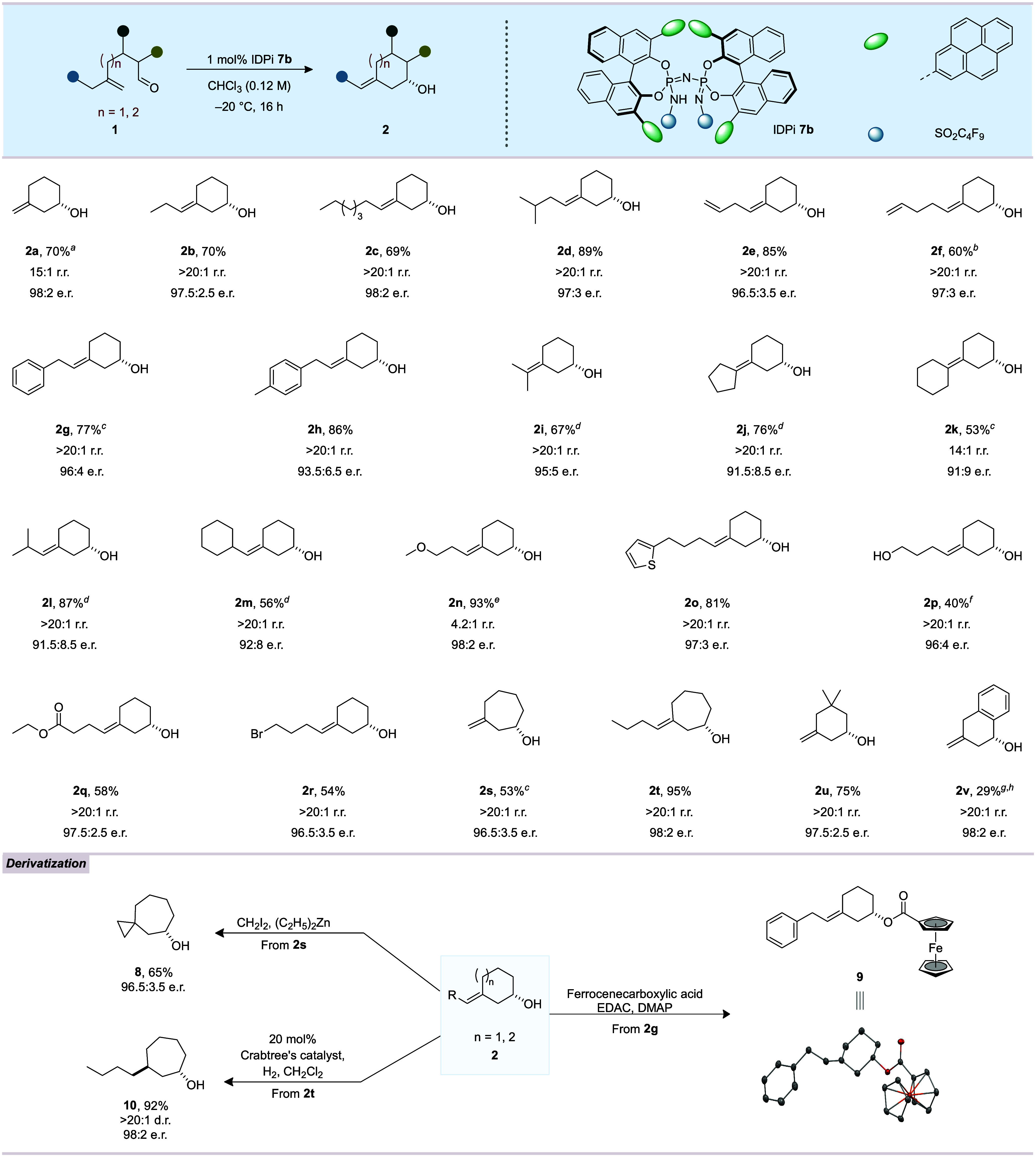
Substrate scope and derivatization. Reactions were performed at a 0.3 mmol scale. Isolated yields were reported after chromatographic purification. The enantiomeric ratio (e.r.) was determined by GC or HPLC analysis. The diastereomeric ratio (d.r.) was determined by ^1^H NMR. Regiomeric ratio (r.r.) is the ratio of the *exo* vs *endo* isomer. ^*a*^Performed on a 1.2 mmol scale. ^*b*^With 2 mol% catalyst at −20 °C for 24 h. ^*c*^At −40 °C for 24 h. ^*d*^At −60 °C for 24 h. ^*e*^A stereoselectivity of 7.7:1 (*E*/*Z*) was observed. ^*f*^The cascade bicyclization product 1-oxaspiro[5.5]­undecan-8-ol was isolated as a side product in 40% yield. ^*g*^With 1.5 mol% catalyst at −20 °C for 48 h. ^*h*^The aromatized ene product 2-methylnaphthalene was isolated in 65% yield. EDAC, *N*-(3-dimethylaminopropyl)-*N*′-ethylcarbodiimid-hydrochloride; DMAP, 4-dimethylaminopyridine. See Supporting Information for the detailed reaction conditions.

We also explored the synthetic utility of the obtained enantiopure homoallylic alcohols. For example, the unsaturated seven-membered alcohol **2s** reacted in a Simmons–Smith cyclopropanation to bicyclic product **8** without deterioration of the enantiopurity. A Steglich esterification of alcohol **2g** with ferrocenecarboxylic acid gave ester **9**, the absolute configuration of which was determined to be (*S*) by single-crystal X-ray diffraction. Furthermore, hydrogenation of **2t** employing 20 mol% of Crabtree’s catalyst afforded the saturated derivative **10** with excellent diastereoselectivity (d.r. >20:1).

To elucidate the reaction mechanism, a kinetic study of the cyclization of substrate **1a** with IDPi **7b** was performed using ^1^H NMR analysis (see Supporting Information for details). The reaction achieved nearly full conversion in 5 h with only 0.1 mol% catalyst loading. The consumption of substrate **1a** shows a characteristic first-order exponential decay, which has been observed for other intramolecular IDPi-catalyzed reactions, for which only one substrate molecule is involved in the rate-limiting step of the reaction.^38,41^ To investigate this reaction further, the deuterium kinetic isotope effect (KIE) of an equimolar intermolecular competition experiment between 5-methylhex-5-enal and deuterated 5-methylhex-5-enal-1-*d* was determined (see Supporting Information for details).^45^ A secondary inverse KIE of 0.77 ± 0.02 was observed, indicating that C–C bond formation is at least partially rate-limiting. Based on these experiments and previous reports,^28,31^ we can propose a plausible reaction mechanism (Figure 3). Accordingly, the catalytic cycle is initiated with the protonation of aldehyde **1a** by IDPi **7b**. This results in the formation of the oxocarbenium ion-IDPi anion pair **I**. Subsequently, enantiodetermining and rate-limiting C–C bond formation occurs, which affords ion pair **II**. Finally, kinetically controlled deprotonation in a confined environment gives the exocyclic product **2a** and regenerates the catalyst. In the case of longer 5-substituents in the substrate, the alkyl group would point outward from the catalyst, likely leading to the (*E*)-product as the major isomer (see Supporting Information Figure S7 for further discussion).

**Figure 3:**
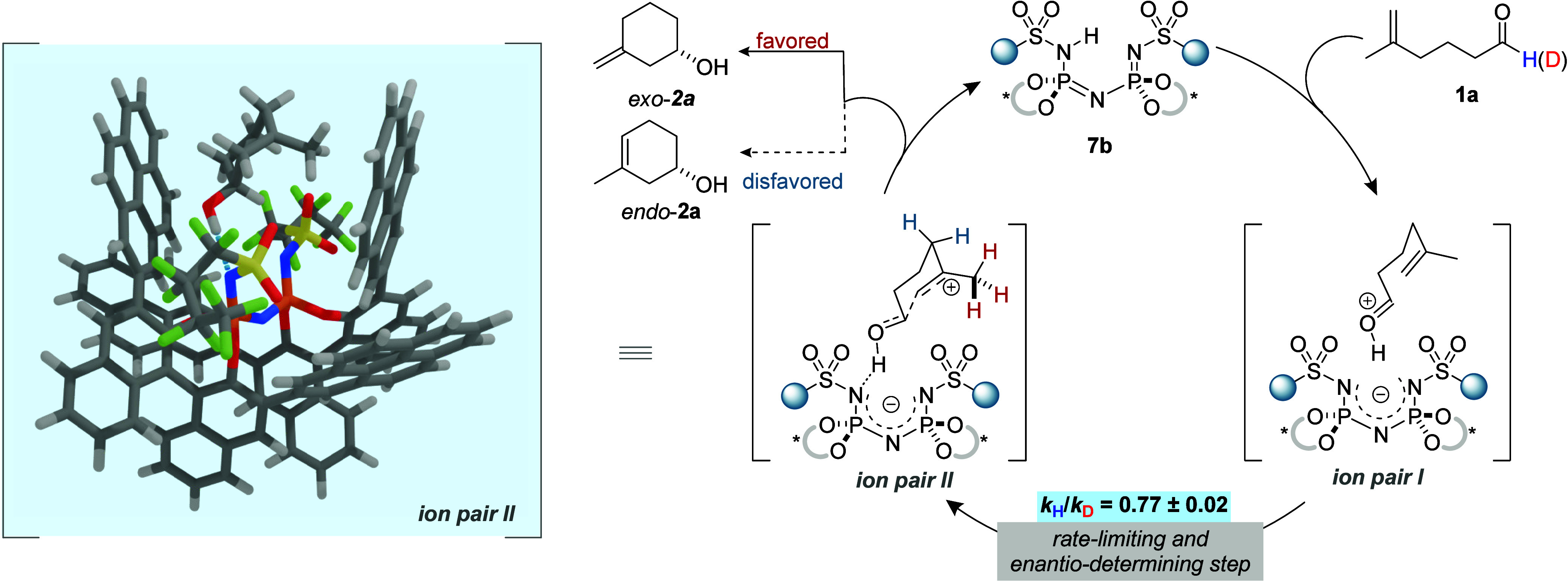
Proposed catalytic cycle for the IDPi catalyzed asymmetric (*ene*–*endo*)-carbonyl–ene type cyclization with 3D structure of ion pair **II**. See Supporting Information for a detailed computational discussion.

In summary, we have developed an organocatalytic asymmetric (*ene*–*endo*)-carbonyl–ene type cyclization of simple alkenyl aldehydes using a highly acidic and confined IDPi catalyst. This method displays a broad substrate scope, providing a variety of six- and seven-membered-ring homoallylic alcohols with an exocyclic olefin in good yields and excellent regio- and enantioselectivitites. We anticipate that the presented approach may aid in the asymmetric synthesis of biologically active natural products.

## Supplementary Material



## Notes

Additional detailed synthetic protocols, analytic data for all compounds, NMR spectra, and GC/HPLC traces (PDF)

## References

[1] Clarke M. L., France M. B. (2008). The Carbonyl Ene Reaction. Tetrahedron.

[2] Snider B. B. (2014). 2.03 Prins
Reactions and Carbonyl, Imine, and Thiocarbonyl Ene Reactions. In Comprehensive Organic Synthesis (Second Edition).

[3] Mikami K., Shimizu M. (1992). Asymmetric Ene Reactions
in Organic Synthesis. Chem. Rev..

[4] Liu X., Zheng K., Feng X. (2014). Advancements
in Catalytic Asymmetric
Intermolecular Ene-Type Reactions. Synthesis.

[5] Bakhtiari A., Safaei-Ghomi J. J. S. (2019). Effects of Chiral Ligands on the Asymmetric Carbonyl–Ene
Reaction. Synlett.

[6] Balha M., Parida C., Chandra
Pan S. (2021). Organocatalytic Asymmetric Ene Reactions. Asian
J. Org. Chem..

[7] Maruoka K., Hoshino Y., Shirasaka T., Yamamoto H. (1988). Asymmetric Ene Reaction
Catalyzed by Chiral Organoaluminum Reagent. Tetrahedron Lett..

[8] Mikami K., Terada M., Nakai T. (1990). Catalytic
Asymmetric Glyoxylate–Ene
Reaction: A Practical Access to α-Hydroxy Esters in High Enantiomeric
Purities. J. Am. Chem. Soc..

[9] Evans D. A., Tregay S. W., Burgey C. S., Paras N. A., Vojkovsky T. (2000). *C*
_2_-Symmetric
Copper­(II) Complexes as Chiral Lewis Acids.
Catalytic Enantioselective Carbonyl–Ene Reactions with Glyoxylate
and Pyruvate Esters. J. Am. Chem. Soc..

[10] Mikami K., Aikawa K., Kainuma S., Kawakami Y., Saito T., Sayo N., Kumobayashi H. (2004). Enantioselective
Catalysis of Carbonyl–Ene
and Friedel–Crafts Reactions with Trifluoropyruvate by ‘Naked’
Palladium­(II) Complexes with SEGPHOS ligands. Tetrahedron: Asymmetry.

[11] Zhao J.-F., Tsui H.-Y., Wu P.-J., Lu J., Loh T.-P. (2008). Highly
Enantioselective Carbonyl–Ene Reactions Catalyzed by In­(III)-PyBox
Complex. J. Am. Chem. Soc..

[12] Zhang X., Wang M., Ding R., Xu Y.-H., Loh T.-P. (2015). Highly
Enantioselective and Anti-Diastereoselective Catalytic Intermolecular
Glyoxylate–Ene Reactions: Effect of the Geometrical Isomers
of Alkenes. Org. Lett..

[13] Yamada T., Ikeno T., Ohtsuka Y., Kezuka S., Sato M., Iwakura I. (2006). Manganese and Cobalt
3-Oxobutylideneaminato Complexes:
Design and Application for Enantioselective Reactions. Sci. Technol. Adv. Mater..

[14] Zheng K., Shi J., Liu X., Feng X. (2008). Asymmetric Carbonyl–Ene Reaction
Catalyzed by Chiral *N,N’*-Dioxide-Nickel­(II)
Complex: Remarkably Broad Substrate Scope. J.
Am. Chem. Soc..

[15] Clarke M. L., Jones C. E., France M. B. (2007). The First
Organocatalytic Carbonyl–Ene
Reaction: Isomerisation-Free C–C Bond Formations Catalysed
by H-bonding Thio-Ureas. Beilstein J. Org. Chem..

[16] Rueping M., Theissmann T., Kuenkel A., Koenigs R. M. (2008). Highly Enantioselective
Organocatalytic Carbonyl–Ene Reaction with Strongly Acidic,
Chiral Brønsted Acids as Efficient Catalysts. Angew. Chem., Int. Ed..

[17] Kikuchi J., Aramaki H., Okamoto H., Terada M. (2019). F_10_BINOL-Derived
Chiral Phosphoric Acid-Catalyzed Enantioselective Carbonyl–Ene
Reaction: Theoretical Elucidation of Stereochemical Outcomes. Chem. Sci..

[18] Kikuchi J., Aizawa Y., Terada M. (2020). Chiral Strong
Brønsted Acid-Catalyzed
Enantioselective Addition Reaction of Simple Olefins with Ethyl Glyoxylate. Org. Chem. Front..

[19] Zheng K., Yin C., Liu X., Lin L., Feng X. (2011). Catalytic Asymmetric
Addition of Alkyl Enol Ethers to 1,2-Dicarbonyl Compounds: Highly
Enantioselective Synthesis of Substituted 3-Alkyl-3-Hydroxyoxindoles. Angew. Chem., Int. Ed..

[20] Cao Q., Yang Y., Mei Y., Ji M., Wang F., Feng X., Cao W. (2025). Catalytic Asymmetric
Construction
of 1,5-Remote Si- and C-Stereocenters via Desymmetrizing Ene Reaction
of Bis­(methallyl)­silanes. Chem. Sci..

[21] Sakane S., Maruoka K., Yamamoto H. (1986). Asymmetric
Cyclization of Unsaturated
Aldehydes Catalyzed by a Chiral Lewis Acid. Tetrahedron.

[22] Mikami K., Sawa E., Terada M. (1991). Asymmetric Catalysis
by Chiral Titanium
Perchlorate for Carbonyl–Ene Cyclization. Tetrahedron: Asymmetry.

[23] Yang D., Yang M., Zhu N.-Y. (2003). Chiral
Lewis Acid-Catalyzed Enantioselective
Intramolecular Carbonyl Ene Reactions of Unsaturated α-Keto
Esters. Org. Lett..

[24] Grachan M. L., Tudge M. T., Jacobsen E. N. (2008). Enantioselective
Catalytic Carbonyl–Ene
Cyclization Reactions. Angew. Chem., Int. Ed..

[25] Rajapaksa N. S., Jacobsen E. N. (2013). Enantioselective Catalytic Transannular Ketone–Ene
Reactions. Org. Lett..

[26] Itoh H., Maeda H., Yamada S., Hori Y., Mino T., Sakamoto M. (2015). BINOL-Al Catalysed
Asymmetric Cyclization and Amplification:
Preparation of Optically Active Menthol Analogs. Org. Biomol. Chem..

[27] Itoh H., Maeda H., Yamada S., Hori Y., Mino T., Sakamoto M. (2016). BINOL-Al Catalyzed
Kinetic Resolution of Citronellal
Analogues: Synthesis of a Variety of Fragrances. Tetrahedron: Asymmetry.

[28] Liu L., Leutzsch M., Zheng Y., Alachraf M. W., Thiel W., List B. (2015). Confined Acid-Catalyzed
Asymmetric Carbonyl–Ene Cyclization. J. Am. Chem. Soc..

[29] Kutateladze D. A., Jacobsen E. N. (2021). Cooperative Hydrogen-Bond-Donor
Catalysis with Hydrogen
Chloride Enables Highly Enantioselective Prins Cyclization Reactions. J. Am. Chem. Soc..

[30] Ishihara H., Huang J., Mochizuki T., Hatano M., Ishihara K. (2021). Enantio- and
Diastereoselective Carbonyl–Ene Cyclization–Acetalization
Tandem Reaction Catalyzed by Tris­(pentafluorophenyl)­borane-Assisted
Chiral Phosphoric Acids. ACS Catal..

[31] Grimm J. A. A., Zhou H., Properzi R., Leutzsch M., Bistoni G., Nienhaus J., List B. (2023). Catalytic Asymmetric Synthesis of
Cannabinoids and Menthol from Neral. Nature.

[32] Ziegler F. E., Sobolov S. B. (1990). Synthesis of a Highly Functionalized Carbon Ring Skeleton
for the Trichothecene Anguidine. J. Am. Chem.
Soc..

[33] Tsukamoto H., Kawase A., Doi T. (2019). Palladium-Catalyzed Umpolung Type-II
Cyclization of Allylic Carbonate-Aldehydes Leading to 3-Methylenecycloalkanol
Derivatives. Adv. Synth. Catal..

[34] Huang J., Zhang Y., Ishihara K. (2025). Enantio- and E-selective Carbonyl–Ene
Cyclization of 5-Substituted 5-Hexenals Catalyzed by Tris­(pentafluorophenyl)­borane-Assisted
Chiral Phosphoric Acids. Org. Lett..

[35] Schreyer L., Properzi R., List B. (2019). IDPi Catalysis. Angew. Chem., Int. Ed..

[36] Tsuji N., Kennemur J. L., Buyck T., Lee S., Prévost S., Kaib P. S. J., Bykov D., Farès C., List B. (2018). Activation of Olefins via Asymmetric
Brønsted Acid Catalysis. Science.

[37] Bae H. Y., Höfler D., Kaib P. S. J., Kasaplar P., De C. K., Döhring A., Lee S., Kaupmees K., Leito I., List B. (2018). Approaching sub-ppm-level
Asymmetric Organocatalysis of a Highly
Challenging and Scalable Carbon–Carbon Bond Forming Reaction. Nat. Chem..

[38] Ouyang J., Kennemur J. L., De C. K., Farès C., List B. (2019). Strong and Confined Acids Enable a Catalytic Asymmetric Nazarov Cyclization
of Simple Divinyl Ketones. J. Am. Chem. Soc..

[39] Zhang P., Tsuji N., Ouyang J., List B. (2021). Strong and Confined
Acids Catalyze Asymmetric Intramolecular Hydroarylations of Unactivated
Olefins with Indoles. J. Am. Chem. Soc..

[40] Raut R. K., Matsutani S., Shi F., Kataoka S., Poje M., Mitschke B., Maeda S., Tsuji N., List B. (2024). Catalytic
Asymmetric Fragmentation of Cyclopropanes. Science.

[41] Wakchaure V. N., DeSnoo W., Laconsay C. J., Leutzsch M., Tsuji N., Tantillo D. J., List B. (2024). Catalytic Asymmetric Cationic Shifts
of Aliphatic Hydrocarbons. Nature.

[42] Brunen S., Mitschke B., Leutzsch M., List B. (2023). Asymmetric Catalytic
Friedel–Crafts Reactions of Unactivated Arenes. J. Am. Chem. Soc..

[43] Luo N., Turberg M., Leutzsch M., Mitschke B., Brunen S., Wakchaure V. N., Nöthling N., Schelwies M., Pelzer R., List B. (2024). The Catalytic
Asymmetric Polyene
Cyclization of Homofarnesol to Ambrox. Nature.

[44] Kütt A., Tshepelevitsh S., Saame J., Lõkov M., Kaljurand I., Selberg S., Leito I. (2021). Strengths of Acids
in Acetonitrile. Eur. J. Org. Chem..

[45] Simmons E. M., Hartwig J. F. (2012). On the Interpretation of Deuterium Kinetic Isotope
Effects in C–H Bond Functionalizations by Transition-Metal
Complexes. Angew. Chem., Int. Ed..

