# Supplementary material for: Catalytic Asymmetric (ene–endo)‑Carbonyl–Ene Type Cyclizations
Source: J Am Chem Soc. 2025 Sep 10;147(38):34225–30. doi: 10.1021/jacs.5c11553 (PMC12464972; doi:10.1021/jacs.5c11553)
Supplement: Supplementary file 1 [file ja5c11553_si_001.pdf]

Supporting Information for

Catalytic Asymmetric (*ene–endo*)-Carbonyl–Ene Type Cyclizations

Lixia Shi[1], Nobuya Tsuji[2], Chendan Zhu[1], Markus Leutzsch[1], Joyce A. A. Grimm[1,2] and Benjamin List[1,2]*

[1]Max-Planck-Institut für Kohlenforschung, Kaiser-Wilhelm-Platz 1, 45470 Mülheim an der Ruhr, Germany

[2]Institute for Chemical Reaction Design and Discovery (ICReDD), Hokkaido University, N21 W10, Kita-ku, Sapporo, 001-0021, Japan

*Email: list@kofo.mpg.de

Contents

1. General Information and Instruments 2

2. Synthesis of Catalysts 4

3. Substrates Synthesis 7

4. Reaction Development 22

5. Enantioselective (*ene–endo*)-Carbonyl–Ene Type Cyclization 23

6. Limitations of the Method 33

7. Derivatization 35

8. Crystallographic Data 37

9. Mechanistic Experiments 42

9.1. Kinetic Study via ^{1}H NMR 42

9.2. Kinetic Isotope Effects Determination from an Intermolecular Competition 43

10. Computational Study 48

10.1. Method 48

10.2. Results and Discussion 48

10.3. Cartesian Coordinates of the Optimized Structures 49

11. NMR Spectra 56

12. GC and HPLC Traces 116

13. References 140

1. General Information and Instruments

Chemicals and Reagents

Chemicals were purchased from commercial suppliers (Abcr, Acros Organics, Alfa Aesar, Apollo Scientific, Fisher Scientific, Fluorochem, Sigma-Aldrich, Strem Chemicals, TCI Deutschland, BLDpharm) as reagent grade and used without further purification unless otherwise stated.

Solvents

Solvents (Et_2O, THF, 1,4-dioxane, CH_2Cl_2, $CHCl_3$, PhMe, pentane, hexane) were dried by distillation from an appropriate drying agent in the technical department of the Max-Planck-Institut für Kohlenforschung and received in Schlenk flasks under argon. Additional solvents (MeCN, EtOAc, MeOH, MTBE) were purchased from commercial suppliers and dried over molecular sieves.

Inert Gas

Dry argon was purchased from Air Liquide with >99.5% purity.

Thin Layer Chromatography

Thin-layer chromatography (TLC) was performed using silica gel pre-coated polyester sheets (Polygram SIL G/UV_{254}, 0.2 mm, with fluorescent indicator; Macherey-Nagel) which was visualized by irradiation with UV light (λ = 254 or 365 nm), basic $KMnO_4$, and/or phosphomolybdic acid (PMA). *$KMnO_4$ stain*: aqueous solution of NaOH (10 wt%, 1.25 mL), $KMnO_4$ (1.50 g), K_2CO_3 (10.00 g) in H_2O (200 mL); *PMA stain*: PMA (20.00 g) in EtOH (200 mL). Preparative TLC was performed on silica gel pre-coated TLC plates SIL G-25 UV_{254}, 0.25 mm silica gel with fluorescent indicator (Macherey-Nagel).

Column Chromatography

Flash column chromatography (FCC) was carried out using Merck (60 Å, 230–400 mesh, particle size 0.040–0.063 mm) using technical grade solvents. Elution was accelerated using compressed air. All reported yields refer to chromatographically and spectroscopically pure compounds unless otherwise stated.

Nomenclature

Nomenclature follows the suggestions proposed by the computer program ChemDraw Professional (23.1.2) of PerkinElmer®.

Nuclear Magnetic Resonance Spectroscopy

^{1}H, ^{13}C, ^{19}F, ^{31}P nuclear magnetic resonance (NMR) spectra were recorded on a Bruker Avance III 500 MHz NMR or a Bruker Avance III 600 MHz NMR spectrometer in a suitable deuterated solvent. The solvent employed and respective measuring frequencies (reported in MHz) are indicated for each experiment. The resonance multiplicity is described as s (singlet), d (doublet), t (triplet), q (quadruplet), p (pentet), hept (heptet), m (multiplet) and b (broad). All spectra were recorded at 298 K unless otherwise noted and processed with MestReNova 15.0.0. Multiplicity and coupling constants are reported as observed. The residual solvent signal was used as the internal reference in ^{1}H NMR and ^{13}C NMR spectra (e.g. $CDCl_3$ = 7.26 ppm in ^{1}H NMR, CD_2Cl_2 = 5.32 ppm in ^{1}H NMR, $CDCl_3$ = 77.16 ppm in ^{13}C NMR

and CD_2Cl_2 = 53.84 ppm in ^{13}C NMR),[1,2] which are reported as follows: chemical shift δ in ppm (multiplicity, coupling constant J in Hz, number of protons). All heteronuclear spectra were proton broadband decoupled unless noted otherwise.

Mass Spectrometry

Electron impact (EI, 70 eV) mass spectrometry was performed on a Thermo Fisher Scientific Q Exactive GC Orbitrap GC-MS/MS system (LRMS and HRMS). Chemical ionization (CI) mass spectrometry was performed on a Thermo Fisher Scientific Q. Exactive GC Orbitrap GC-MS/MS system (LRMS and HRMS). Electrospray ionization (ESI) was performed on a Thermo Fisher Scientific Q Exactive Plus Orbitrap (LRMS and HRMS). The ionization method and mode of detection employed is indicated for the respective experiment and all masses are reported in atomic mass units divided by elementary charge number (*m/z*).

Specific Rotations

Specific rotations $[\alpha]_D^T$ were measured on a Rudolph RA AUTOPOL® IV Automatic Polarimeter at the indicated temperature with a sodium lamp (sodium D line, λ = 589 nm). Measurements were performed in an acid resistant 1 mL cell (50 mm length) with concentrations (g/(100 mL)) reported in the corresponding solvent.

High Performance Liquid Chromatography

High performance liquid chromatography (HPLC) was performed on a Shimadzu LC-40AD (SIL-40C XS autosampler, DGU-405 degasser, CTO-40C column oven, SPD-M40 PDA detector, SCL-40 controller, LC-40D XS pump) or Prominence LC-2030C (LC-2030 autosampler, LC-2030/2040 degasser, LC-2030 column oven, LC-2030 PDA detector, LC-2030 pump) using CHIRALPAK® and CHIRALCEL®columns. All solvents used were HPLC-grade solvents purchased from Sigma-Aldrich or VWR. The specific column employed, and the respective solvent mixtures are indicated for each experiment.

Gas Chromatography

Gas chromatography (GC) analyses were performed on an Agilent Technologies 6890N, Agilent Technologies 6890, Agilent Technologies 7890A, Agilent Technologies 7890B (splitmode capillary injection system, flame ionization detector (FID)). The conditions for achiral and chiral separation employed are described in detail for the individual experiments.

Gas Chromatography-Mass Spectrometry

Gas chromatography-mass spectrometry analyses were performedon a Thermo Fisher Scientific Q Exactive Orbitrap /Thermo Scientific Trace 1310. The columns and conditions employed are described in detail for the individual experiments.

2. Synthesis of Catalysts

Procedure for cross-coupling reactions and subsequent MOM-deprotection and (S,S)-IDPi 7a and 7b synthesis

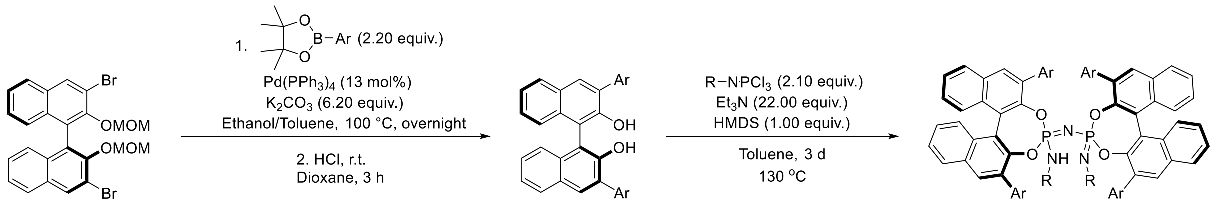

(*S*)-3,3'-di(pyren-2-yl)-[1,1'-binaphthalene]-2,2'-diol:

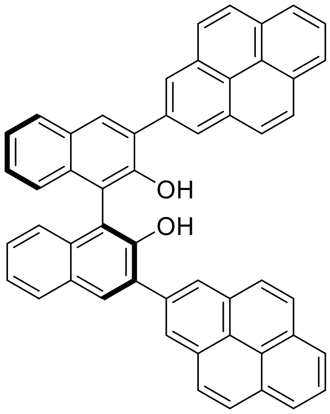

In a flame-dried flask under Ar, (*S*)-3,3'-dibromo-2,2'-bis(methoxymethoxy)-1,1'-binaphthalene (1.20 g, 2.25 mmol, 1.00 equiv.) and 2-(4,4,5,5-tetramethyl-1,3,2-dioxaborolan-2-yl)-pyrene (1.60 g, 4.87 mmol, 2.16 equiv.) were dissolved in potassium carbonate aqueous solution (2 mol/L, 7 mL, 14 mmol, 6.20 equiv.), toluene (12 mL) and ethanol (8 mL). After degassing the reaction mixture, $Pd(PPh_3)_4$ (340 mg, 0.29 mmol, 13 mol%) was subsequently added and the reaction was sealed and heated to reflux for 16 h. The reaction mixture was cooled to room temperature (r.t.), exposed to air, diluted with H_2O (15 mL) and CH_2Cl_2 (15 mL). The layers were separated and the aqueous layer was further extracted with CH_2Cl_2 (3 × 15 mL). The combined organic layers were washed with brine, dried over Na_2SO_4, filtered and concentrated under reduced pressure. The crude reaction product was subjected to MOM-deprotection without prior purification. A flame-dried flask was charged with crude MOM-protected 3,3'-substituted BINOL and HCl (4 M in dioxane, 5 mL, 8.90 equiv.) was added at r.t.. The reaction was stirred until full conversion of the starting material. The solvent was removed under reduced pressure. Purification by column chromatography (CC) (silica gel, hexane/DCM, 3:2) afforded the desired compound as a pale-yellow solid (1.29 g, 83% yield).

$\mathbf{R_f}$ (hexane:DCM 3:2) = 0.23.

^{1}H-NMR (501 MHz, $CDCl_3$) δ 8.57 (s, 4H), 8.27 (s, 2H), 8.21 (d, *J* = 7.6 Hz, 4H), 8.17–8.10 (m, 8H), 8.06–7.99 (m, 4H), 7.51–7.44 (m, 2H), 7.44–7.39 (m, 4H), 5.64 (s, 2H).

^{13}C-NMR (126 MHz, $CDCl_3$) δ 150.5, 135.2, 133.4, 132.5, 131.5, 131.4, 131.1, 129.8, 128.8, 128.0, 127.7, 127.6, 126.2, 125.3, 124.7, 124.6, 124.2, 113.0.

HRMS (ESI) *m/z* calculated for $C_{52}H_{29}O_2$ $[M\text{-}H]^-$: 685.217305, found: 685.218090.

$[\boldsymbol{\alpha}]_{\boldsymbol{D}}^{\mathbf{20}}$ = +198.8 (*c* = 0.41, $CHCl_3$).

(*S*,*S*)-Imidodiphosphorimidate 7a (IDPi 7a):

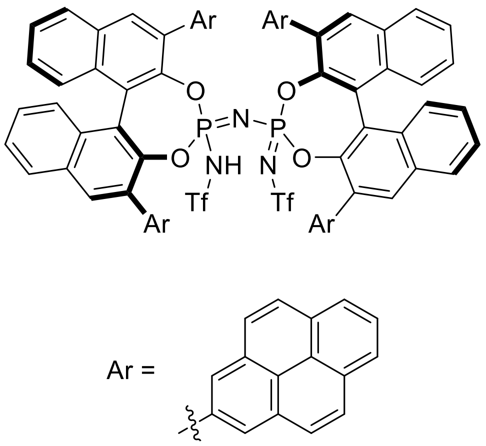

In a schlenk tube under Ar, (*S*)-3,3'-di(pyren-2-yl)-[1,1'-binaphthalene]-2,2'-diol (150 mg, 0.22 mmol, 2.28 equiv.) was dissolved in toluene (1 mL), perfluorobutylsulfonylphosphorimidoyl trichloride (32 µL, 0.20 mmol, 2.09 equiv.) and trimethylamine (0.3 mL, 2.15 mmol, 22.44 equiv.) were added and the reaction was stirred at 60 °C for 2 h. Then 1,1,1,3,3,3-hexamethyldisilazane (20 µL, 0.10 mmol, 1.00 equiv.) was added to the reaction mixture and heated to 130 °C for 3 d. The reaction mixture was cooled to r.t. and concentrated under reduced pressure. Purification by CC (silica gel, CH_2Cl_2/pentane, 100:1 to 50:1), to afford the desired IDPi as a salt. The product was acidified by passing over a short plug of DOWEX 50WX8 (H-form) in DCM and obtained as a yellow solid after removing the solvent (60 mg, 36% yield).

R*f* (CH_2Cl_2:pentane 50:1) = 0.40.

^{1}H NMR (501 MHz, CD_2Cl_2) δ 8.37 (d, *J* = 8.5 Hz, 2H), 8.27 (s, 2H), 8.23 (d, *J* = 8.3 Hz, 2H), 8.16 (d, *J* = 8.2 Hz, 2H), 8.06–7.98 (m, 10H), 7.96–7.86 (m, 14H), 7.76 (dt, *J* = 14.8, 7.5 Hz, 6H), 7.64 (d, *J* = 8.5 Hz, 2H), 7.59–7.52 (m, 6H), 7.14 (s, 2H), 6.88 (s, 4H), 6.52 (d, *J* = 9.0 Hz, 4H).

^{13}C NMR (126 MHz, CD_2Cl_2) δ 145.1, 143.6, 134.6, 134.5, 134.0, 133.4, 132.7, 132.43, 132.38, 132.2, 131.8, 131.5, 131.2, 130.6, 130.5, 129.5, 128.4, 128.2, 127.9, 127.6, 127.5, 127.3, 127.1, 127.0, 126.8, 126.7, 126.6, 126.00, 125.97, 125.6, 125.0, 124.8, 124.6, 124.05, 123.99, 123.9, 123.3, 122.3, 122.0. (other signals not detected or observed)

^{19}F NMR (471 MHz, CD_2Cl_2) δ –79.3.

^{31}P NMR (203 MHz, CD_2Cl_2) δ –17.1.

HRMS (ESI) *m/z* calculated for $C_{106}H_{56}F_6N_3O_8P_2S_2$ $[M-H]^-$: 1738.28937, found: 1738.29146.

(*S*,*S*)-Imidodiphosphorimidate 7b (IDPi 7b):

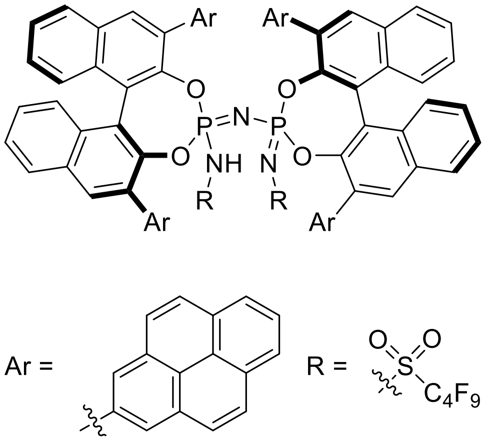

In a schlenk tube under Ar, (*S*)-3,3'-di(pyren-2-yl)-[1,1'-binaphthalene]-2,2'-diol (159.4 mg, 0.23 mmol, 2.30 equiv.) was dissolved in toluene (1.3 mL), $PCl_3SO_2C_4F_9$ (48 µL, 0.21 mmol, 2.05 equiv.) and trimethylamine (0.3 mL, 2.15 mmol, 21.37 equiv.) were added and the reaction mixture was stirred at 60 °C for 2 h. Then 1,1,1,3,3,3-hexamethyldisilazane (21 µL, 0.10 mmol, 1.00 equiv.) was added to the reaction mixture and heated to 130 °C for 3 d. The reaction mixture was cooled to r.t. and concentrated under reduced pressure. Purification by CC (silica gel, CH_2Cl_2/pentane, 3:2 to 4:1), to afford the desired

IDPi as a salt. The product was acidified by passing over a short plug of DOWEX 50WX8 (H-form) in DCM and obtained as a yellow solid after removing the solvent (115 mg, 56% yield).

R*f* (CH_2Cl_2:pentane 4:1) = 0.37.

^{1}H NMR (501 MHz, CD_2Cl_2) δ 8.33–8.26 (m, 2H), 8.26–8.20 (m, 4H), 8.13 (d, *J* = 8.2 Hz, 2H), 7.99 (dd, *J* = 20.2, 8.5 Hz, 10H), 7.94–7.81 (m, 14H), 7.74 (dt, *J* = 22.0, 7.6 Hz, 6H), 7.59 (d, *J* = 8.6 Hz, 2H), 7.52 (t, *J* = 8.0 Hz, 6H), 7.10 (s, 2H), 6.96 (s, 4H), 6.53 (s, 4H).

^{13}C NMR (126 MHz, CD_2Cl_2) δ 145.2, 134.5, 134.5, 134.1, 133.7, 132.9, 132.7, 132.4, 132.2, 131.8, 131.4, 131.3, 130.6, 130.5, 129.4, 128.1, 127.9, 127.62, 127.58, 127.3, 127.0, 126.8, 126.7, 126.5, 125.9, 125.5, 125.0, 124.6, 124.0, 123.9, 123.3, 122.1. (other signals not detected or observed)

^{19}F NMR (471 MHz, CD_2Cl_2) δ –81.2 (t, *J* = 10.4 Hz, 6F), –111.3– –113.8 (m, 4F), –120.9 – –122.1 (m, 4F), –126.2 (q, *J* = 11.2 Hz, 4F).

^{31}P NMR (203 MHz, $CDCl_3$) δ –15.6.

HRMS (ESI) *m/z* calculated for $C_{112}H_{56}F_{18}N_3O_8P_2S_2$ [M-H]$^-$: 2038.27021, found: 2038.27241.

3. Substrates Synthesis

Preparation of Grignard reagents

A 50 mL two-necked round-bottom flask, which was flame-dried and argon-flushed prior to use, was equipped with a magnetic stir bar, magnesium turnings (60 mmol, 3.00 equiv.) and a crystal of iodine. The flask was heated until purple fumes was visible, then cooled to r.t. over 10 minutes with vigorous stirring. 10 mL of anhydrous THF was added, and a solution of alkyl bromide (20 mmol, 1.00 equiv.) in 10 mL THF was added dropwise. The generated dark brown solution was stirred at r.t. for 2 h and the concentration was determined by titration before use.

Synthesis of 1a–1o

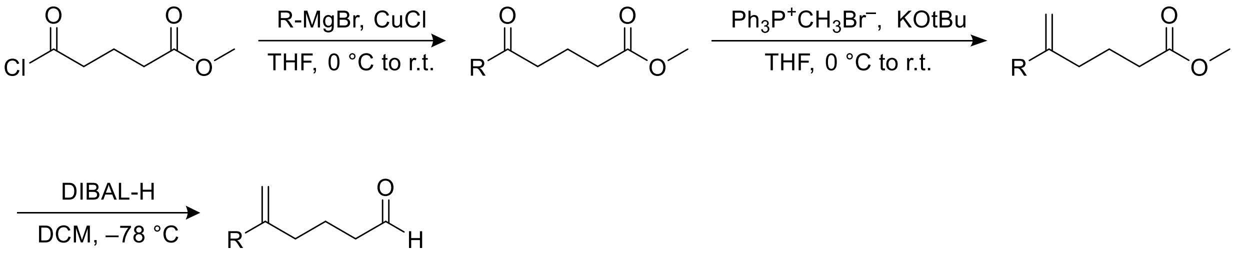

1a–1o

The reactions were modified from the literature procedures.[3,4] General procedure: To a solution of methyl 5-chloro-5-oxopentanoate (1.00 equiv.) and CuCl (1.10 equiv.) in THF, Grignard reagent (1.10 equiv.) was added dropwise at 0 °C under argon atmosphere. The solution was stirred at r.t. until full consumption of the starting material. The reaction was quenched with ice water, filtered through a pad of Celite. Then the organic layer of resulting filtrate was washed with 10% HCl three times, dried over Na_2SO_4 and concentrated under reduced pressure. The crude mixture was purified by CC (silica gel, pentane/EtOAc, 15:1). The obtained product was used for the next Wittig olefination step.

The triphenylmethylphosphonium bromide (1.50 equiv.) was dissolved in THF under argon atmosphere, and then potassium *tert*-butoxide (1.30 equiv.) was added at 0 °C. After stirring at 0 °C for 1 h, the corresponding ketone ester obtained from first step (1.00 equiv.) was added dropwise. The mixture was stirred at r.t. until full consumption of the starting material. After addition of H_2O, the aqueous phase was extracted with Et_2O. The combined organic layer was washed with H_2O, brine and dried over Na_2SO_4, then concentrated under reduced pressure. The crude mixture was purified by CC (silica gel, pentane/EtOAc, 20:1). The obtained ester was used for the next reduction step.

To a stirred solution of corresponding ester obtained from the second step (1.00 equiv.) in CH_2Cl_2 at –78 °C was added diisobutylaluminum hydride (1.50 equiv.) over a period of 10 min under agron atmosphere. After 15 min, the reaction mixture was quenched with saturated aqueous solution of NH_4Cl

and allowed to warm to r.t. over 0.5 h. The product was extracted with DCM, dried over Na_2SO_4, filtered and concentrated under reduced pressure. Purification by CC (silica gel, pentane/EtOAc, 20:1) afforded the desired aldehyde.

5-methylhex-5-enal (1a)

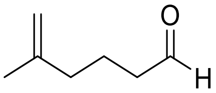

Prepared according to general procedure. Colorless oil, 47% yield.

^{1}H NMR (501 MHz, $CDCl_3$) δ 9.78 (t, *J* = 1.7 Hz, 1H), 4.74 (t, *J* = 1.8 Hz, 1H), 4.70–4.66 (m, 1H), 2.43 (td, *J* = 7.3, 1.7 Hz, 2H), 2.08–2.00 (m, 2H), 1.78 (p, *J* = 7.4 Hz, 2H), 1.71 (t, *J* = 1.1 Hz, 3H).

^{13}C NMR (126 MHz, $CDCl_3$) δ 202.6, 144.8, 111.0, 43.4, 37.1, 22.3, 20.0.

HRMS (CI) *m/z* calculated for $C_7H_{13}O$ $[M+H]^+$: 113.096090, found: 113.096210.

5-methyleneoctanal (1b)

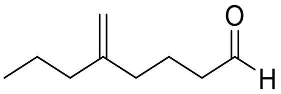

Prepared according to general procedure. Colorless oil, 54% yield.

^{1}H NMR (501 MHz, CD_2Cl_2) δ 9.74 (t, *J* = 1.7 Hz, 1H), 4.84–4.63 (m, 2H), 2.41 (td, *J* = 7.3, 1.7 Hz, 2H), 2.07–1.95 (m, 4H), 1.75 (p, *J* = 7.4 Hz, 2H), 1.44 (h, *J* = 7.4 Hz, 2H), 0.90 (t, *J* = 7.4 Hz, 3H).

^{13}C NMR (126 MHz, CD_2Cl_2) δ 202.8, 149.3, 109.7, 43.8, 38.3, 35.6, 21.3, 20.5, 14.0.

HRMS (CI) *m/z* calculated for $C_9H_{20}N_1O$ $[M+NH_4]^+$: 158.15394, found: 158.15369.

5-methylenedodecanal (1c)

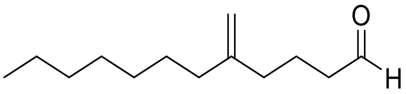

Prepared according to general procedure. Colorless oil, 47% yield.

^{1}H NMR (501 MHz, CD_2Cl_2) δ 9.77–9.70 (m, 1H), 4.78–4.68 (m, 2H), 2.46–2.35 (m, 2H), 2.09–1.95 (m, 4H), 1.74 (h, *J* = 7.7 Hz, 2H), 1.41 (q, *J* = 7.6 Hz, 2H), 1.32–1.24 (m, 8H), 0.93–0.84 (m, 3H).

^{13}C NMR (126 MHz, CD_2Cl_2) δ 202.8, 149.6, 109.6, 43.8, 36.2, 35.6, 32.3, 29.8, 29.6, 28.2, 23.1, 20.5, 14.3.

HRMS (CI) *m/z* calculated for $C_{13}H_{28}N_1O$ $[M+NH_4]^+$: 214.21654, found: 214.21674.

8-methyl-5-methylenenonanal (1d)

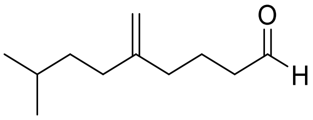

Prepared according to general procedure. Colorless oil, 73% yield.

^{1}H NMR (501 MHz, CD_2Cl_2) δ 9.74 (t, *J* = 1.7 Hz, 1H), 4.75 (d, *J* = 1.8 Hz, 1H), 4.71 (dd, *J* = 2.1, 1.1 Hz, 1H), 2.41 (td, *J* = 7.3, 1.7 Hz, 2H), 2.08–1.97 (m, 4H), 1.75 (p, *J* = 7.4 Hz, 2H), 1.61–1.48 (m, 1H), 1.35–1.26 (m, 2H), 0.89 (d, *J* = 6.6 Hz, 6H).
^{13}C NMR (126 MHz, CD_2Cl_2) δ 202.8, 149.9, 109.5, 43.8, 37.5, 35.7, 34.0, 28.3, 22.7, 20.5.
HRMS (CI) *m/z* calculated for $C_{11}H_{24}N_1O$ $[M+NH_4]^+$: 186.185239, found: 186.185270.

5-methylenenon-8-enal (1e)

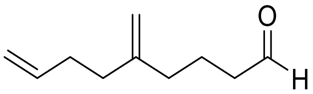

Prepared according to general procedure. Colorless oil, 64% yield.
^{1}H NMR (501 MHz, CD_2Cl_2) δ 9.80–9.69 (m, 1H), 5.92–5.75 (m, 1H), 5.03 (dq, *J* = 17.1, 1.8 Hz, 1H), 4.97–4.92 (m, 1H), 4.81–4.74 (m, 2H), 2.42 (td, *J* = 7.4, 1.6 Hz, 2H), 2.25–2.17 (m, 2H), 2.08 (dt, *J* = 23.1, 8.0 Hz, 4H), 1.75 (p, *J* = 7.4 Hz, 2H).
^{13}C NMR (126 MHz, CD_2Cl_2) δ 202.7, 148.7, 138.9, 114.7, 110.1, 43.7, 35.7, 35.5, 32.4, 20.5.
HRMS (CI) *m/z* calculated for $C_{10}H_{17}O$ $[M+H]^+$: 153.127390, found: 153.127630.

5-methylenedec-9-enal (1f)

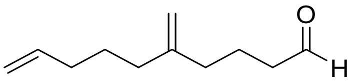

Prepared according to general procedure. Colorless oil, 64% yield.
^{1}H NMR (501 MHz, CD_2Cl_2) δ 9.74 (t, *J* = 1.7 Hz, 1H), 5.83 (ddt, *J* = 17.0, 10.2, 6.7 Hz, 1H), 5.01 (dq, *J* = 17.1, 1.8 Hz, 1H), 4.95 (dt, *J* = 10.2, 1.8 Hz, 1H), 4.75 (dd, *J* = 12.9, 1.8 Hz, 2H), 2.41 (td, *J* = 7.3, 1.7 Hz, 2H), 2.09–2.00 (m, 6H), 1.80–1.70 (m, 2H), 1.57–1.47 (m, 2H).
^{13}C NMR (126 MHz, CD_2Cl_2) δ 202.7, 149.2, 139.2, 114.7, 109.9, 43.7, 35.6, 33.8, 27.4, 20.5.
HRMS (CI) *m/z* calculated for $C_{11}H_{22}N_1O$ $[M+NH_4]^+$: 184.169589, found: 184.169570.

5-methylene-7-phenylheptanal (1g)

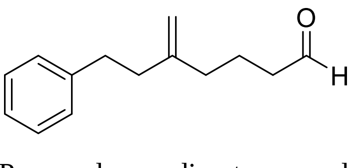

Prepared according to general procedure. Colorless oil, 71% yield.
^{1}H NMR (501 MHz, CD_2Cl_2) δ 9.75 (t, *J* = 1.6 Hz, 1H), 7.32–7.13 (m, 5H), 4.84–4.75 (m, 2H), 2.79–2.72 (m, 2H), 2.42 (td, *J* = 7.3, 1.7 Hz, 2H), 2.36–2.29 (m, 2H), 2.10 (t, *J* = 7.6 Hz, 2H), 1.78 (p, *J* = 7.4 Hz, 2H).
^{13}C NMR (126 MHz, CD_2Cl_2) δ 202.7, 148.8, 142.7, 128.7, 128.7, 126.1, 110.2, 43.7, 38.0, 35.8, 34.6, 20.5.
HRMS (EI) *m/z* calculated for $C_{14}H_{18}O$ $[M]^+$: 202.135215, found: 202.135300.

5-methylene-7-(*p*-tolyl)heptanal (1h)

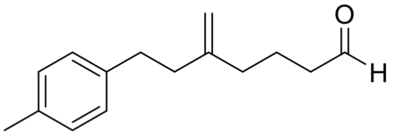

Prepared according to general procedure. Colorless oil, 53% yield.

^{1}H NMR (501 MHz, CD_2Cl_2) δ 9.74 (t, *J* = 1.7 Hz, 1H), 7.17–7.00 (m, 4H), 4.83–4.79 (m, 1H), 4.77 (s, 1H), 2.74–2.66 (m, 2H), 2.42 (td, *J* = 7.3, 1.7 Hz, 2H), 2.36–2.26 (m, 5H), 2.10 (t, *J* = 7.7 Hz, 2H), 1.77 (p, *J* = 7.4 Hz, 2H).

^{13}C NMR (126 MHz, CD_2Cl_2) δ 202.7, 148.9, 139.5, 135.7, 129.3, 128.6, 110.2, 43.7, 38.1, 35.8, 34.2, 21.1, 20.5.

HRMS (EI) *m/z* calculated for $C_{15}H_{20}O$ $[M]^+$: 216.150865, found: 216.151190.

6-methyl-5-methyleneheptanal (1i)

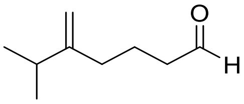

Prepared according to general procedure. Colorless oil, 44% yield.

^{1}H NMR (501 MHz, CD_2Cl_2) δ 9.74 (t, *J* = 1.7 Hz, 1H), 4.79 (t, *J* = 1.2 Hz, 1H), 4.69 (q, *J* = 1.5 Hz, 1H), 2.42 (td, *J* = 7.3, 1.7 Hz, 2H), 2.23 (pd, *J* = 6.8, 1.1 Hz, 1H), 2.11–2.01 (m, 2H), 1.81–1.68 (m, 2H), 1.02 (d, *J* = 6.9 Hz, 6H).

^{13}C NMR (126 MHz, CD_2Cl_2) δ 202.8, 155.6, 107.2, 43.9, 34.0, 34.0, 22.0, 20.9.

HRMS (EI) *m/z* calculated for $C_9H_{16}O$ $[M]^+$: 140.119565, found: 140.119710.

5-cyclopentylhex-5-enal (1j)

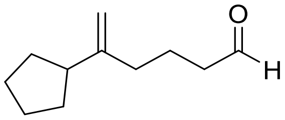

Prepared according to general procedure. Colorless oil, 82% yield.

^{1}H NMR (501 MHz, CD_2Cl_2) δ 9.75 (t, *J* = 1.7 Hz, 1H), 4.80 (d, *J* = 1.6 Hz, 1H), 4.70 (s, 1H), 2.46–2.33 (m, 3H), 2.11–2.03 (m, 2H), 1.84–1.73 (m, 4H), 1.72–1.63 (m, 2H), 1.61–1.53 (m, 2H), 1.42–1.30 (m, 2H).

^{13}C NMR (126 MHz, CD_2Cl_2) δ 202.8, 152.8, 107.4, 46.3, 43.9, 35.2, 31.9, 25.3, 20.9.

HRMS (EI) *m/z* calculated for $C_{11}H_{18}O$ $[M]^+$: 166.135215, found: 166.135260.

5-cyclohexylhex-5-enal (1k)

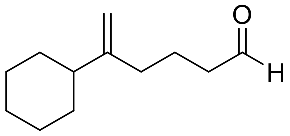

Prepared according to general procedure. Colorless oil, 54% yield.

^{1}H NMR (501 MHz, CD_2Cl_2) δ 9.74 (t, *J* = 1.7 Hz, 1H), 4.76 (s, 1H), 4.69 (q, *J* = 1.5 Hz, 1H), 2.41 (td, *J* = 7.3, 1.7 Hz, 2H), 2.09–2.00 (m, 2H), 1.84 (tt, *J* = 11.6, 3.2 Hz, 1H), 1.78–1.71 (m, 6H), 1.70–1.64 (m, 1H), 1.28 (qt, *J* = 13.1, 3.3 Hz, 2H), 1.21–1.09 (m, 3H).

^{13}C NMR (126 MHz, CD_2Cl_2) δ 202.9, 154.9, 107.7, 44.4, 43.9, 34.6, 32.9, 27.2, 26.8, 21.0.

HRMS (CI) *m/z* calculated for $C_{12}H_{21}O$ $[M+H]^+$: 181.15869, found: 181.15868.

7-methyl-5-methyleneoctanal (1l)

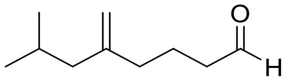

Prepared according to general procedure. Colorless oil, 58% yield.

^{1}H NMR (501 MHz, CD_2Cl_2) δ 9.74 (t, *J* = 1.7 Hz, 1H), 4.75 (q, *J* = 1.6 Hz, 1H), 4.73 (dt, *J* = 2.1, 1.1 Hz, 1H), 2.41 (td, *J* = 7.3, 1.7 Hz, 2H), 2.05–2.00 (m, 2H), 1.89 (dd, *J* = 7.3, 1.1 Hz, 2H), 1.75 (p, *J* = 7.4 Hz, 3H), 0.87 (d, *J* = 6.5 Hz, 6H).

^{13}C NMR (126 MHz, CD_2Cl_2) δ 202.8, 148.3, 111.0, 43.8, 35.3, 26.4, 22.7, 22.6, 20.5.

HRMS (CI) *m/z* calculated for $C_{10}H_{19}O$ $[M+H]^+$: 155.143040, found: 155.143080.

5-(cyclohexylmethyl)hex-5-enal (1m)

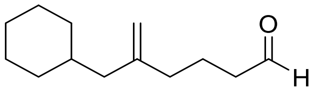

Prepared according to general procedure. Colorless oil, 70% yield.

^{1}H NMR (501 MHz, CD_2Cl_2) δ 9.74 (d, *J* = 1.7 Hz, 1H), 4.74 (d, *J* = 2.0 Hz, 1H), 4.71 (s, 1H), 2.41 (td, *J* = 7.3, 1.7 Hz, 2H), 2.01 (t, *J* = 7.6 Hz, 2H), 1.90 (d, *J* = 7.2 Hz, 2H), 1.81–1.65 (m, 7H), 1.46–1.36 (m, 1H), 1.25–1.12 (m, 3H), 0.90–0.80 (m, 2H).

^{13}C NMR (126 MHz, CD_2Cl_2) δ 202.8, 147.7, 111.0, 44.5, 43.8, 35.9, 35.4, 33.7, 27.0, 26.8, 20.5.

HRMS (EI) *m/z* calculated for $C_{13}H_{22}O$ $[M]^+$: 194.166515, found: 194.166290.

8-methoxy-5-methyleneoctanal (1n)

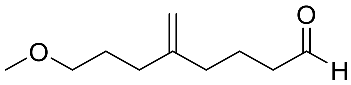

Prepared according to general procedure. Colorless oil, 64% yield.

^{1}H NMR (501 MHz, $CDCl_3$) δ 9.78–9.72 (m, 1H), 4.74 (d, *J* = 16.6 Hz, 2H), 3.38–3.33 (m, 2H), 3.33–3.27 (m, 3H), 2.48–2.36 (m, 2H), 2.04 (t, *J* = 6.7 Hz, 4H), 1.81–1.61 (m, 4H).

^{13}C NMR (126 MHz, $CDCl_3$) δ 202.5, 148.1, 110.1, 72.4, 58.7, 43.4, 35.4, 32.3, 27.8, 20.1.

HRMS (ESI) *m/z* calculated for $C_{10}H_{18}O_2Na$ $[M+Na]^+$: 193.119899, found: 193.119950.

5-methylene-9-(thiophen-2-yl)nonanal (1o)

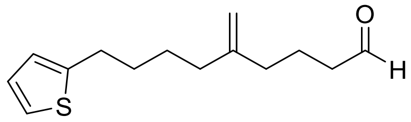

Prepared according to general procedure. Colorless oil, 50% yield.

^{1}H NMR (501 MHz, $CDCl_3$) δ 9.77 (t, *J* = 1.7 Hz, 1H), 7.11 (dd, *J* = 5.1, 1.2 Hz, 1H), 6.91 (dd, *J* = 5.1, 3.4 Hz, 1H), 6.78 (d, *J* = 3.6 Hz, 1H), 4.76 (s, 1H), 4.73 (s, 1H), 2.84 (t, *J* = 7.6 Hz, 2H), 2.43 (td, *J* = 7.3, 1.7 Hz, 2H), 2.04 (t, *J* = 7.6 Hz, 4H), 1.77 (p, *J* = 7.4 Hz, 2H), 1.69 (p, *J* = 7.6 Hz, 2H), 1.50 (p, *J* = 7.7 Hz, 2H).

^{13}C NMR (126 MHz, $CDCl_3$) δ 202.6, 148.4, 145.6, 126.8, 124.1, 122.9, 110.1, 43.5, 35.6, 35.3, 31.5, 29.9, 27.2, 20.1.

HRMS (ESI) *m/z* calculated for $C_{14}H_{20}O_1S_1Na$ $[M+Na]^+$: 259.112707, found: 259.112630.

Synthesis of 1p–1r

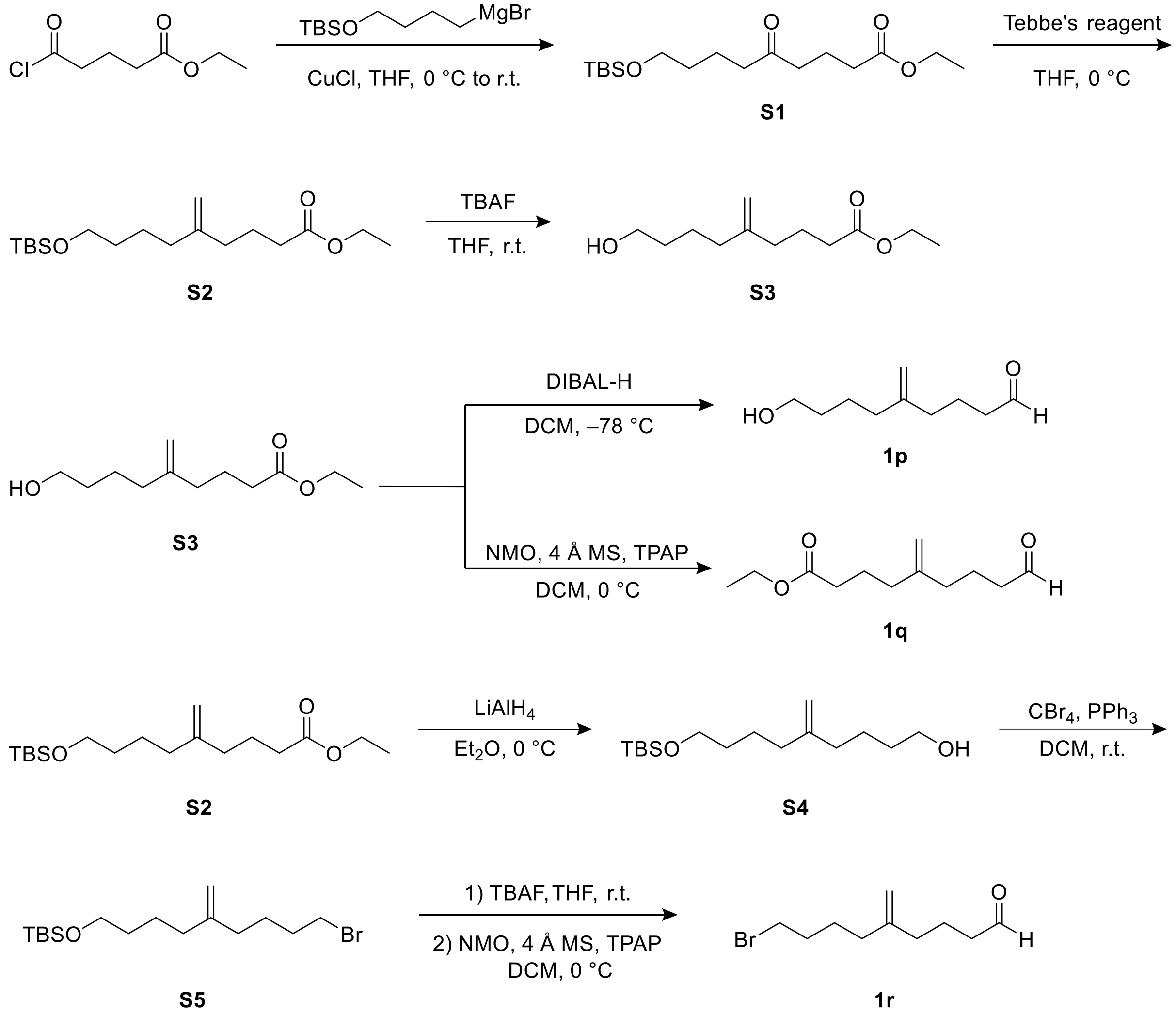

ethyl 9-((*tert*-butyldimethylsilyl)oxy)-5-oxononanoate (S1)

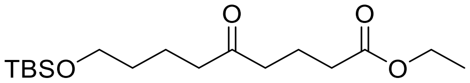

To a solution of ethyl 5-chloro-5-oxopentanoate (6.60 g, 36.95 mmol, 1.00 equiv.) and CuCl (4.10 g, 41.41 mmol, 1.12 equiv.) in THF (40 mL), The prepared Grignard reagent (4-((*tert*-butyldimethylsilyl)oxy)butyl)magnesium bromide (40 mL, 1 M in THF, 1.10 equiv.) was added dropwise at 0 °C under argon atmosphere. The solution was stirred at r.t. until full consumption of the starting material. The reaction was quenched with ice water, filtered through a pad of Celite. Then the organic layer of resulting filtrate was washed with 10% HCl three times, dried over Na_2SO_4 and concentrated under reduced pressure. The crude mixture was purified by CC (silica gel, pentane/EtOAc, 15:1) to give **S1** as a colorless oil (11.70 g, 96% yield).

^{1}H NMR (501 MHz, $CDCl_3$) δ 4.11 (q, *J* = 7.2 Hz, 2H), 3.59 (t, *J* = 6.2 Hz, 2H), 2.43 (dt, *J* = 25.3, 7.3 Hz, 4H), 2.31 (t, *J* = 7.3 Hz, 2H), 1.95–1.82 (m, 2H), 1.66–1.57 (m, 2H), 1.53–1.46 (m, 2H), 1.24 (t, *J* = 7.1 Hz, 3H), 0.88 (s, 9H), 0.03 (s, 6H).

^{13}C NMR (126 MHz, $CDCl_3$) δ 210.3, 173.3, 62.9, 60.5, 42.7, 41.6, 33.5, 32.4, 26.1, 20.4, 19.1, 18.5, 14.4, -5.2.

HRMS (ESI) *m/z* calculated for $C_{17}H_{34}O_4Si_1Na_1$ $[M+Na]^+$: 353.211858, found: 353.211870.

ethyl 9-((*tert*-butyldimethylsilyl)oxy)-5-methylenenonanoate (S2)

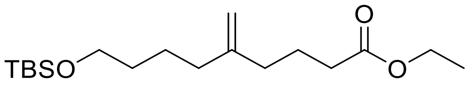

The reaction was modified from the literature procedure.[5] To a stirred solution of **S1** (9.52 g, 28.80 mmol, 1.00 equiv.) in THF (120 mL) was added Tebbe reagent (60 mL, 0.5 M in toluene, 1.04 equiv.) at 0 °C under Ar. The reaction mixture was stirred at 0 °C until complete conversion of the starting material. The reaction was quenched with aqueous NaOH (30 %), filtered through a pad of Celite. Then the organic layer was dried over Na_2SO_4 and concentrated under reduced pressure. The crude mixture was purified by CC (silica gel, pentane/EtOAc, 20:1) to give **S2** as a colorless oil (6.00 g, 63% yield).

^{1}H NMR (501 MHz, $CDCl_3$) δ 4.73 (d, *J* = 10.4 Hz, 2H), 4.12 (q, *J* = 7.1 Hz, 2H), 3.61 (t, *J* = 6.2 Hz, 2H), 2.29 (t, *J* = 7.5 Hz, 2H), 2.09–1.96 (m, 4H), 1.76 (p, *J* = 7.6 Hz, 2H), 1.52–1.41 (m, 4H), 1.25 (t, *J* = 7.1 Hz, 3H), 0.89 (s, 9H), 0.04 (s, 6H).

^{13}C NMR (126 MHz, $CDCl_3$) δ 173.8, 148.8, 109.8, 63.2, 60.4, 35.7, 35.4, 34.0, 32.7, 26.1, 24.1, 23.1, 18.5, 14.4, -5.1.

HRMS (CI) *m/z* calculated for $C_{18}H_{40}N_1O_3Si_1$ $[M+NH_4]^+$: 346.277197, found: 346.276980.

ethyl 9-hydroxy-5-methylenenonanoate (S3)

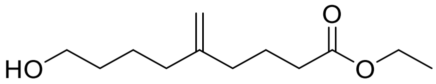

To a stirred solution of **S2** (1.80 g, 5.48 mmol, 1.00 equiv.) in THF (10 mL) was added TBAF (5.5 mL, 1 M in THF, 1.00 equiv.) at r.t. under Ar. The reaction mixture was stirred at r.t. until complete conversion of the starting material. The reaction mixture was concentrated under reduced pressure and purified by CC (silica gel, pentane/EtOAc, 5:1) to give **S3** as a colorless oil (943.7 mg, 80% yield).

^{1}H NMR (501 MHz, $CDCl_3$) δ 4.72 (d, *J* = 7.1 Hz, 2H), 4.10 (q, *J* = 7.1 Hz, 2H), 3.62 (t, *J* = 6.3 Hz, 2H), 2.27 (t, *J* = 7.5 Hz, 2H), 2.05–1.98 (m, 4H), 1.80–1.70 (m, 2H), 1.60–1.44 (m, 4H), 1.23 (t, *J* = 7.1 Hz, 3H).

^{13}C NMR (126 MHz, $CDCl_3$) δ 173.9, 148.5, 109.9, 62.9, 60.4, 35.6, 35.3, 33.9, 32.5, 23.9, 23.1, 14.4.

HRMS (ESI) *m/z* calculated for $C_{12}H_{22}O_3Na_1$ $[M+Na]^+$: 237.146114, found: 237.145980.

9-((*tert*-butyldimethylsilyl)oxy)-5-methylenenonan-1-ol (S4)

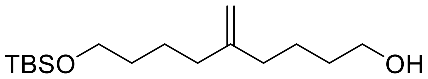

To a suspension of $LiAlH_4$ (13 mL, 1 M in THF, 1.53 equiv.) in Et_2O (15 mL) at 0 °C was slowly added an Et_2O solution of **S2** (2.80 g, 8.52 mmol, 1.00 equiv.). The reaction mixture was stirred at 0 °C until complete conversion of the starting material. H_2O was added very slowly until no more gas evolution was observed. Then, more H_2O was added and stirring was continued at r.t. until the reaction mixture became white. The reaction mixture was filtered on a pad of Celite® and concentrated under reduced pressure. The crude product was purified by CC (silica gel, pentane/EtOAc, 5:1) to give **S4** as colorless oil (2.34 g, 96% yield).

^{1}H NMR (501 MHz, $CDCl_3$) δ 4.71 (s, 2H), 3.63 (dt, *J* = 17.6, 6.3 Hz, 4H), 2.02 (q, *J* = 7.6 Hz, 4H), 1.61–1.43 (m, 8H), 0.89 (s, 9H), 0.01 (s, 6H).
^{13}C NMR (126 MHz, $CDCl_3$) δ 149.6, 109.2, 63.2, 63.0, 35.85, 35.82, 32.7, 32.6, 26.1, 24.1, 24.0, 18.5, -5.1.
HRMS (ESI) *m/z* calculated for $C_{16}H_{34}O_2Si_1Na_1$ $[M+Na]^+$: 309.222028, found: 309.222320.

((9-bromo-5-methylenenonyl)oxy)(*tert*-butyl)dimethylsilane (S5)

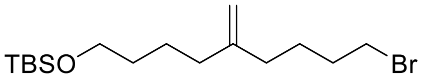

The reaction was modified from the literature procedure.[6] A oven dried flask under Ar was charged with Ph_3P (2.20 g, 8.38 mmol, 2.00 equiv.), CBr_4 (2.80 g, 8.44 mmol, 2.02 equiv.) and **S4** (1.20 g, 4.19 mmol, 1.00 equiv.) followed by addition of anhydrous CH_2Cl_2 (25 mL) at 0 °C. The mixture was stirred at r.t. for 20 h. The mixture was filtered on a pad of silica gel with hexane. The filtrate was evaporated under vacumm to give **S5** as colorless oil without purification (1.35 g, 92% yield).

^{1}H NMR (501 MHz, $CDCl_3$) δ 4.72 (d, *J* = 5.7 Hz, 2H), 3.62 (t, *J* = 6.2 Hz, 2H), 3.42 (t, *J* = 6.8 Hz, 2H), 2.03 (q, *J* = 7.9 Hz, 4H), 1.89–1.81 (m, 2H), 1.61–1.55 (m, 2H), 1.53–1.43 (m, 4H), 0.89 (s, 9H), 0.05 (s, 6H).
^{13}C NMR (126 MHz, $CDCl_3$) δ 149.1, 109.5, 63.2, 35.8, 35.1, 33.9, 32.7, 32.5, 26.3, 26.1, 24.1, 18.5, -5.1.
HRMS (CI) *m/z* calculated for $C_{16}H_{37}N_1O_1Br_1Si_1$ $[M+NH_4]^+$: 366.182242, found: 366.181900.

9-hydroxy-5-methylenenonanal (1p)

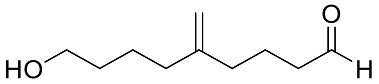

To a stirred solution of **S3** (0.45 g, 2.33 mmol, 1.00 equiv.) in CH_2Cl_2 (15 mL) at –78 °C was added diisobutylaluminum hydride (3.2 mL, 1 M in hexane, 1.52 equiv.) over a period of 10 min under agron

atmosphere. After 15 min, the reaction mixture was quenched with saturated aqueous solution of NH_4Cl and allowed to warm to r.t. over 0.5 h. The product was extracted with DCM, dried over Na_2SO_4, filtered and concentrated under reduced pressure. Purification by CC (silica gel, pentane/EtOAc, 3:1) afforded **1p** as colorless oil (190 mg, 53% yield).

^{1}H NMR (501 MHz, $CDCl_3$) δ 9.76 (t, *J* = 1.7 Hz, 1H), 4.76 (s, 1H), 4.72 (s, 1H), 3.64 (t, *J* = 6.3 Hz, 2H), 2.43 (td, *J* = 7.3, 1.7 Hz, 2H), 2.03 (q, *J* = 7.9 Hz, 5H), 1.76 (p, *J* = 7.4 Hz, 2H), 1.57–1.53 (m, 2H), 1.51–1.46 (m, 2H).
^{13}C NMR (126 MHz, $CDCl_3$) δ 202.7, 148.4, 110.1, 62.9, 43.4, 35.6, 35.2, 32.5, 23.9, 20.1.
HRMS (ESI) *m/z* calculated for $C_{10}H_{18}O_2Na$ $[M+Na]^+$: 193.119899, found: 193.120250.

ethyl 5-methylene-9-oxononanoate (1q)

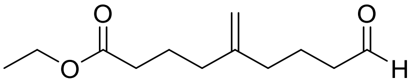

The reaction was modified from the literature procedure.[7] NMO (0.79 g, 6.74 mmol, 3.01 equiv.) and 4 Å MS (0.79 g) were added to a solution of **S3** (0.48 g, 2.24 mmol, 1.00 equiv.) in CH_2Cl_2 (30 mL). The mixture was stirred for 5 min at 0 °C and TPAP (40 mg, 5 mol%) was added. The reaction mixture was stirred at r.t. until complete conversion of the starting material. The mixture was filtered on a pad of silica gel and concentrated under reduced pressure. The crude product was purified by CC (silica gel, pentane/EtOAc, 8:1) to give **1q** as colorless oil (195.8 mg, 41% yield).

^{1}H NMR (501 MHz, $CDCl_3$) δ 9.76 (t, *J* = 1.7 Hz, 1H), 4.75 (d, *J* = 5.4 Hz, 2H), 4.11 (q, *J* = 7.1 Hz, 2H), 2.42 (td, *J* = 7.3, 1.7 Hz, 2H), 2.28 (t, *J* = 7.5 Hz, 2H), 2.08–1.98 (m, 4H), 1.81–1.70 (m, 4H), 1.24 (t, *J* = 7.1 Hz, 3H).
^{13}C NMR (126 MHz, $CDCl_3$) δ 202.5, 173.7, 147.6, 110.7, 60.4, 43.4, 35.2, 35.1, 33.9, 23.0, 20.1, 14.4.
HRMS (ESI) *m/z* calculated for $C_{12}H_{20}O_3Na$ $[M+Na]^+$: 235.130464, found: 235.130450.

9-bromo-5-methylenenonanal (1r)

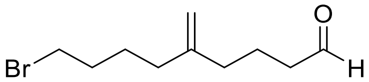

To a stirred solution of **S5** (1.00 g, 2.86 mmol, 1.00 equiv.) in THF (5 mL) was added TBAF (4.0 mL, 1 M in THF, 1.40 equiv.) at r.t. under Ar. The reaction mixture was stirred at r.t. until complete conversion of the starting material. The reaction mixture was concentrated under reduced pressure and purified by CC (silica gel, pentane/EtOAc, 3:1) to give the alcohol product as a colorless oil (0.50 g, 74% yield). The obtained alcohol (0.50 g, 2.13 mmol, 1.00 equiv.) was used for next Ley-Griffith oxidation to give **1r** as colorless oil (130 mg, 26% yield).

^{1}H NMR (501 MHz, $CDCl_3$) δ 9.77 (t, *J* = 1.7 Hz, 1H), 4.77 (s, 1H), 4.75 (s, 1H), 3.41 (t, *J* = 6.8 Hz, 2H), 2.44 (td, *J* = 7.3, 1.7 Hz, 2H), 2.05–2.00 (m, 4H), 1.88–1.82 (m, 2H), 1.79–1.74 (m, 2H), 1.60–1.55 (m, 2H).
^{13}C NMR (126 MHz, $CDCl_3$) δ 202.5, 147.9, 110.5, 43.5, 35.2, 35.0, 33.9, 32.4, 26.2, 20.1.
HRMS (ESI) *m/z* calculated for $C_{10}H_{17}Br_1O_1Na$ $[M+Na]^+$: 255.03550, found: 255.03549.

Synthesis of 1s–1t

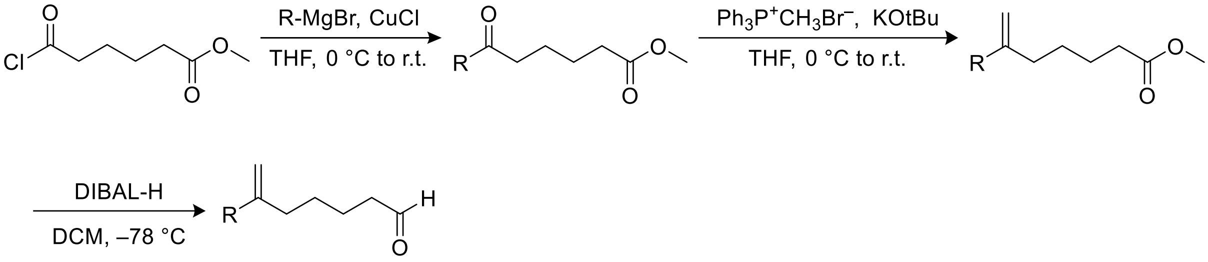

1s, 1t

The synthetic protocol follows the same methodology as employed for substrates **1a**–**1o**, utilizing methyl 6-chloro-6-oxohexanoate as starting material in the Grignard reaction step.

6-methylhept-6-enal (1s)

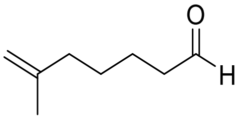

Prepared according to general procedure. Colorless oil, 52% yield.
^{1}H NMR (501 MHz, CD_2Cl_2) δ 9.73 (t, *J* = 1.8 Hz, 1H), 4.70 (t, *J* = 1.8 Hz, 1H), 4.69–4.65 (m, 1H), 2.42 (td, *J* = 7.3, 1.8 Hz, 2H), 2.03 (t, *J* = 7.7 Hz, 2H), 1.71 (s, 3H), 1.65–1.57 (m, 2H), 1.51–1.42 (m, 2H).
^{13}C NMR (126 MHz, CD_2Cl_2) δ 202.9, 146.1, 110.2, 44.1, 37.8, 27.4, 22.4, 22.1.
HRMS (EI) *m/z* calculated for $C_8H_{14}O$ $[M]^+$: 126.103915, found: 126.103990.

6-methylenedecanal (1t)

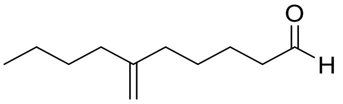

Prepared according to general procedure. Colorless oil, 60% yield.
^{1}H NMR (501 MHz, CD_2Cl_2) δ 9.73 (t, *J* = 1.8 Hz, 1H), 4.70 (dt, *J* = 7.2, 1.5 Hz, 2H), 2.42 (td, *J* = 7.3, 1.8 Hz, 2H), 2.18–1.95 (m, 4H), 1.61 (p, *J* = 7.3 Hz, 2H), 1.50–1.25 (m, 6H), 0.90 (t, *J* = 7.3 Hz, 3H).
^{13}C NMR (126 MHz, CD_2Cl_2) δ 203.0, 150.2, 109.0, 44.2, 36.1, 36.0, 30.4, 27.6, 22.9, 22.2, 14.2.
HRMS (CI) *m/z* calculated for $C_{11}H_{21}O$ $[M+H]^+$: 169.158690, found: 169.158810.

Synthesis of 1u

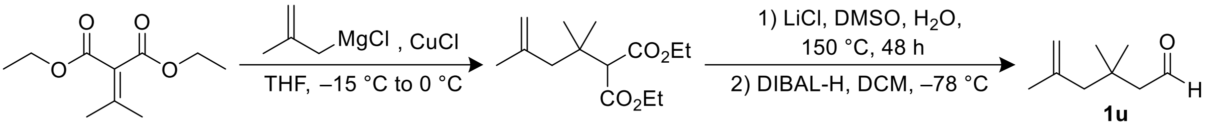

1u

Diethyl 2-(2,4-dimethylpent-4-en-2-yl)malonate

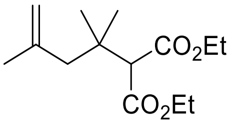

To a solution of CuCl (1.90 g, 19 mmol, 1.20 equiv.) in 50 mL dry THF, the solution of (2-methylallyl)-magnesiumchloride (45 mL, 0.5 M in THF, 1.50 equiv.) was added at –15 °C and the mixture was stirred for 15 min. Then diethyl-isopropylidenmalonate (3.00 g, 15 mmol, 1.00 equiv.) was added. After 2 h, the mixture was quenched with saturated aqueous solution of NH_4Cl, extracted with MTBE, dried over Na_2SO_4 and concentrated under reduced pressure. The crude mixture was purified by CC (silica gel, pentane/EtOAc, 20:1) to give the product as a colorless oil (3.80 g, 97% yield).

^{1}H NMR (501 MHz, $CDCl_3$) δ 4.91 (dq, J = 2.9, 1.5 Hz, 1H), 4.70 (dt, J = 2.6, 0.9 Hz, 1H), 4.18 (q, J = 7.1 Hz, 4H), 3.36 (s, 1H), 2.25 (s, 2H), 1.79 (t, J = 1.2 Hz, 3H), 1.26 (t, J = 7.1 Hz, 6H), 1.14 (s, 6H). The analytical data was identical to the reported value.[8]

3,3,5-trimethylhex-5-enal (1u)

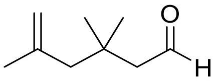

The reaction was modified from the literature procedure.[8] Diethyl 2-(2,4-dimethylpent-4-en-2-yl)malonate (1.00 g, 3.90 mmol, 1.00 equiv.) was dissolved in DMSO (5 mL), LiCl (0.60 g, 14 mmol, 3.60 equiv.) and H_2O (0.15 mL, 2.00 equiv.) were added and the mixture was refluxed. The reaction was monitored by TLC. After the completion of reaction, H_2O was added and the mixture was extracted with MTBE. The organic layer was washed with brine and dried over Na_2SO_4. The solvent was removed under vacumm to give the crude oil 0.50 g. The oil was dissolved in 6 mL DCM, then DIBAL-H (4 mL, 1 mol/L in hexane, 1.50 equiv.) was added at –78 °C over a period of 10 min under agron atmosphere. After 15 min, the reaction mixture was quenched with saturated aqueous solution of NH_4Cl and allowed to warm to r.t. over 0.5 h. The product was extracted with DCM, dried over Na_2SO_4, filtered and concentrated under reduced pressure. Purification by CC (silica gel, pentane/EtOAc, 20:1) afforded the desired aldehyde **1p** (90.6 mg, 24% yield).

^{1}H NMR (501 MHz, CD_2Cl_2) δ 9.84 (t, J = 3.0 Hz, 1H), 4.91 (dq, J = 2.8, 1.5 Hz, 1H), 4.68 (dq, J = 2.6, 0.9 Hz, 1H), 2.29 (d, J = 3.0 Hz, 2H), 2.08 (d, J = 0.8 Hz, 2H), 1.81–1.75 (m, 3H), 1.08 (s, 6H).
^{13}C NMR (126 MHz, CD_2Cl_2) δ 203.7, 143.2, 115.3, 55.3, 50.5, 34.4, 28.2, 25.4.

HRMS (EI) *m/z* calculated for $C_9H_{16}O$ $[M]^+$: 140.119565, found: 140.119610.

Synthesis of 1v

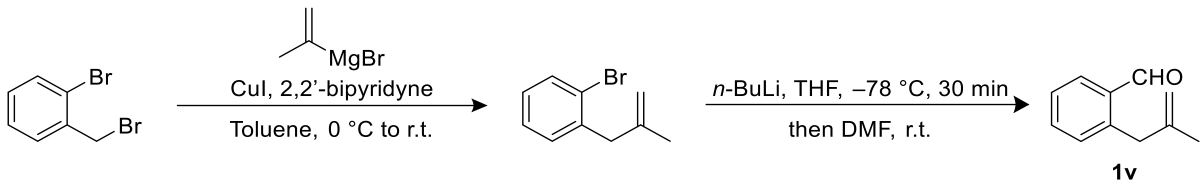

1-bromo-2-(2-methylallyl)benzene

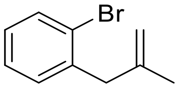

Prop-1-en-2-ylmagnesium bromide solution (20 mL, 0.5 M in THF, 1.00 equiv.) was slowly added to solution of 2-bromobenzyl bromide (2.50 g, 10.0 mmol, 1.00 equiv.), CuI (194 mg, 1.0 mmol, 0.10 equiv.) and 2,2'-bipyridyne (156 mg, 1.0 mmol, 0.10 equiv.) in toluene (15 mL) at 0 °C. The reaction was allowed to warm to r.t. and stirred until full consumption of starting material. Saturated aqueous solution of NH_4Cl was added and the mixture was extracted with Et_2O. The combined organic layers were dried over Na_2SO_4, concentrated under reduced pressure, and then purified by CC (silica gel, pentane) to give the product as a colorless oil (1.40 g, 66% yield).

^{1}H NMR (501 MHz, $CDCl_3$) δ 7.58 (d, *J* = 8.0 Hz, 1H), 7.28–7.23 (m, 2H), 7.10 (ddd, *J* = 8.0, 6.8, 2.3 Hz, 1H), 4.88 (s, 1H), 4.61 (s, 1H), 3.48 (s, 2H), 1.78 (s, 3H).
The analytical data was identical to the reported value.[9,10]

2-(2-methylallyl)benzaldehyde (1v)

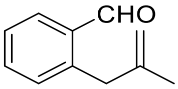

The reaction was modified from the literature procedure.[9] *n*-BuLi (4 mL, 2.5 M, 1.50 equiv.) was added dropwise to a solution of 1-bromo-2-(2-methylallyl)benzene (1.40 g, 6.6 mmol, 1.00 equiv.) in THF (10 mL) at –78 °C. After stirring for 30 min, DMF (1.5 mL, 20 mmol, 3.00 equiv.) in THF (2 mL) was added dropwise. The reaction was allowed to warm to r.t. and stirred until full consumption of starting material. Saturated aqueous solution of NH_4Cl was added and the mixture was extracted with Et_2O. The combined organic phases were dried over Na_2SO_4 and concentrated under reduced pressure. Purification of the crude residue by CC (silica gel, pentane/EtOAc, 30:1) afforded the desired aldehyde as yellow oil (0.83 g, 78% yield).

^{1}H NMR (501 MHz, $CDCl_3$) δ 10.24 (d, *J* = 1.2 Hz, 1H), 7.87 (dt, *J* = 7.8, 1.6 Hz, 1H), 7.53 (tt, *J* = 7.5, 1.7 Hz, 1H), 7.39 (td, *J* = 7.5, 1.8 Hz, 1H), 7.28 (d, *J* = 7.6 Hz, 1H), 4.84 (q, *J* = 1.5 Hz, 1H), 4.45 (s, 1H), 3.73 (s, 2H), 1.78 (s, 3H).
^{13}C NMR (126 MHz, $CDCl_3$) δ 192.2, 145.4, 142.2, 134.5, 133.9, 131.8, 130.7, 127.1, 112.6, 40.3, 23.0.
HRMS (EI) *m/z* calculated for $C_{11}H_{12}O$ $[M]^+$: 160.088265, found: 160.088090.

Synthesis of deuterium-labelled substrate 5-methylhex-5-enal-1-*d*

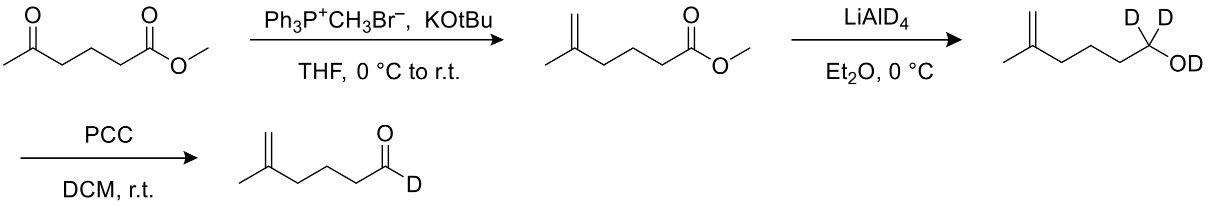

The reactions are modified from the literature procedures.[4,11] The triphenylmethylphosphonium bromide (16.50 g, 46 mmol, 1.50 equiv.) was dissolved in 80 mL THF under argon atmosphere, and then potassium *tert*-butoxide (4.40 g, 39 mmol, 1.30 equiv.) was added at 0 °C. After stirring at 0 °C for 1 h, ethyl-4-acetylbutyrate (5.00 g, 30 mmol, 1.00 equiv.) was added dropwise. The mixture was stirred at r.t. until full consumption of the starting material. After addition of H_2O, the aqueous phase was extracted with Et_2O. The combined organic layer was washed with H_2O, brine and dried over Na_2SO_4, then concentrated under reduced pressure. The crude mixture was purified by CC (silica gel, pentane/EtOAc, 25:1) to give the product as colorless oil (4 g, 84% yield). The obtained ester methyl 5-methylhex-5-enoate was used for the next reduction step.

To a suspension of $LiAlD_4$ (1.22 g, 29 mmol, 1.50 equiv.) in Et_2O (20 mL) at 0 °C was slowly added an Et_2O solution of the methyl 5-methylhex-5-enoate (3.00 g, 19 mmol, 1.00 equiv.). The reaction mixture was stirred at 0 °C until complete conversion of the starting material (~1 h). H_2O was added very slowly until no more gas evolution was observed. Then, more H_2O was added and stirring was continued at r.t. until the reaction mixture became white. The reaction mixture was filtered on a pad of Celite® and concentrated under reduced pressure. The crude product was purified by CC (silica gel, pentane/EtOAc, 9:1) to give the product as colorless oil (2.13 g, 95% yield). The obtained alcohol was used for the next oxidation step.

To a stirred solution of pyridiniumchlorochromate (5.5 g, 26 mmol, 1.50 equiv.) in DCM at r.t. was added the previously obtained alcohol (2.00 g, 17 mmol, 1.00 equiv.). The reaction mixture was stirred at r.t. until full consumption of the starting material. The mixture was extracted with DCM, dried over Na_2SO_4, filtered and concentrated under reduced pressure. Purification by CC (silica gel, pentane/Et_2O, 20:1) afforded the deuterated aldehyde as colorless oil (0.80 g, 41% yield).

5-methylhex-5-enal-1-*d*

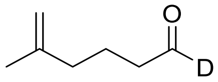

^{1}H NMR (501 MHz, CD_2Cl_2) δ 4.76–4.72 (m, 1H), 4.72–4.66 (m, 1H), 2.40 (t, *J* = 7.3 Hz, 2H), 2.08–2.01 (m, 2H), 1.75 (p, *J* = 7.5 Hz, 2H), 1.71 (t, *J* = 1.2 Hz, 3H).

^{13}C NMR (126 MHz, CD_2Cl_2) δ 202.5 (t, *J* = 26.2 Hz), 145.5, 110.8, 43.4 (t, *J* = 3.7 Hz), 37.4, 22.3, 20.3.

HRMS (EI) *m/z* calculated for $C_7H_{11}D_1O$ $[M]^+$: 113.094542, found: 113.094600.

4. Reaction Development

General procedure for the catalytic cyclization of 1a with different Brønsted acid catalysts.

In an oven-dried GC vial equipped with a teflon-coated magnetic stirring bar, different Brønsted acid catalysts (5 mol%) and DCM (0.06 M, 0.2 mL) were added, and the resultant solution was stirred for 5 min at 0 °C. Aldehyde **1a** (0.012 mmol, 1.0 equiv.) was added and the reaction mixture was stirred for an additional 6 h. The reaction was quenched with one drop of triethylamine. The yield of cyclization product was determined by ^{1}H NMR analysis with mesitylene as an internal standard. The ratio of *exo* and *endo* products was determined by ^{1}H NMR. The enantiomeric ratio was determined by GC analysis after purification by prep. TLC (pentane/EtOAc, 5:1).

Table S1. Initial screening with commonly used Brønsted acid catalysts:[a]

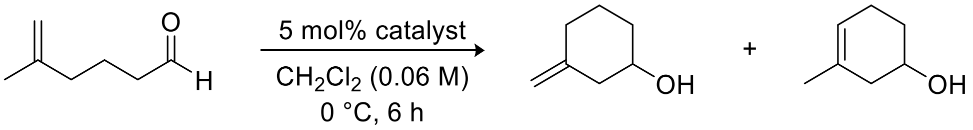

1a *exo*-**2a** *endo*-**2a**

entry	catalyst	conversion (%)	yield (%)	*exo*:*endo*	e.r. (*exo*)
1	**S1**	20	14	89:11	54:46
2	**S2**	31	29	95:5	80:20
3	**S3**	>95%	63	68:32	51:49
4	**S4**	>95%	67	70:30	59.5:40.5
5	**S5**	>95%	55	80:20	65:35

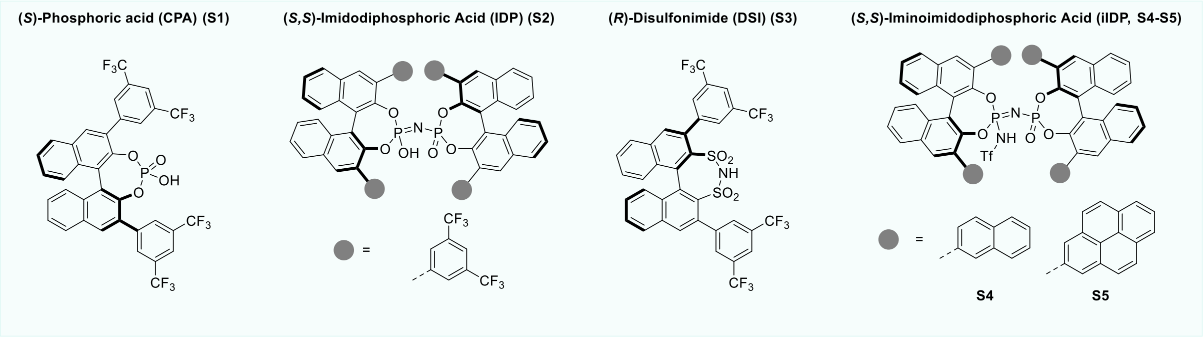

[a]Reactions were performed with 5-methylhex-5-enal **1a** (0.012 mmol, 1.00 equiv.) and catalysts (5 mol%) in DCM (0.06 M, 0.2 mL) at 0 °C for 6 h. Conversion and yield were determined by ^{1}H NMR using mesitylene as internal standard, the ratio of *exo* and *endo* products was determined by ^{1}H NMR and enantiomeric ratio (e.r.) was determined by GC analysis.

5. Enantioselective (*ene–endo*)-Carbonyl–Ene Type Cyclization

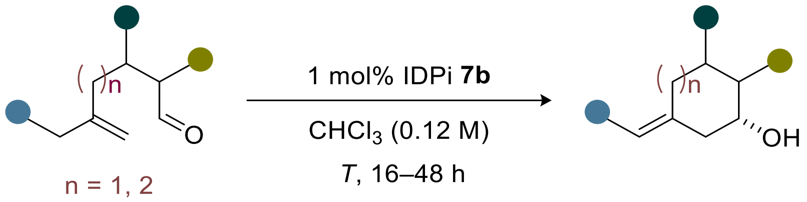

1 **2**

General Procedure:

In an oven-dried vial, IDPi catalyst **7b** (0.003 mmol, 1 mol%) was dissolved in 2.5 mL anhydrous chloroform and if required cooled down to the desired reaction temperature. Aldehyde **1** (0.3 mmol) was added in one portion and the reaction was stirred for 16–48 h. The reaction was quenched with 100 µL triethylamine. After evaporation of the solvent, purification of the crude mixture by CC (silica gel, pentane/EtOAc, 10:1) afforded the corresponding homoallylic alcohols.

(*S*)-3-methylenecyclohexan-1-ol (2a)

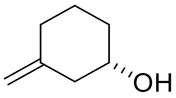

The reaction was performed at –20 °C for 16 h with 1.2 mmol aldehyde. Colorless oil (95.0 mg, 70% yield).

$\mathbf{R}_f$ (pentane:EtOAc 5:1) = 0.33.

^{1}H NMR (501 MHz, $CDCl_3$) δ 4.74 (s, 1H), 4.70 (s, 1H), 3.76 (tt, *J* = 7.8, 3.6 Hz, 1H), 2.49 (dd, *J* = 13.0, 4.0 Hz, 1H), 2.18–1.98 (m, 3H), 1.90–1.81 (m, 1H), 1.80–1.72 (m, 1H), 1.53–1.45 (m, 1H), 1.44–1.34 (m, 1H).

^{13}C NMR (126 MHz, $CDCl_3$) δ 146.1, 110.1, 70.2, 44.1, 34.4, 34.4, 23.9.

HRMS (CI) *m/z* for $C_7H_{16}N_1O$ $[M+NH_4]^+$: 130.122639, found: 130.122840.

$[\alpha]_D^{20}$ = +16.7 (*c* = 0.82, $CHCl_3$).

Chiral GC (BGB-178/BGB-15; Temperature program: 220/65 120 min iso 8/min 240, 2 min/350): t_R (*minor*) = 37.57 min (2.24%), t_R (*major*) = 41.61 min (97.76%), e.r. = 98:2.

(*S*,*E*)-3-propylidenecyclohexan-1-ol (2b)

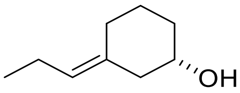

The reaction was performed at –20 °C for 16 h. Colorless oil (30.0 mg, 70% yield).

$\mathbf{R}_f$ (pentane:EtOAc 5:1) = 0.33.

^{1}H NMR (501 MHz, CD_2Cl_2) δ 5.17 (t, *J* = 7.2 Hz, 1H), 3.64 (s, 1H), 2.39 (dd, *J* = 12.7, 4.0 Hz, 1H), 2.28–2.20 (m, 1H), 2.07–1.95 (m, 3H), 1.94–1.79 (m, 2H), 1.75–1.66 (m, 1H), 1.48–1.39 (m, 1H), 1.37–1.27 (m, 1H), 0.93 (t, *J* = 7.5 Hz, 3H).

^{13}C NMR (126 MHz, CD_2Cl_2) δ 135.6, 126.8, 70.6, 46.0, 35.4, 27.9, 24.0, 21.0, 14.9.

HRMS (EI) *m/z* for $C_9H_{16}O$ $[M]^+$: 140.119565, found: 140.119690.

$[\alpha]_D^{20}$ = +11.4 (*c* = 0.76, $CHCl_3$).

Chiral GC (BGB-174/BGB-170; Temperature program: 220/45 800 min iso 8/min 240, 2 min iso/350): t_R (*minor*) = 517.69 min (2.49%), t_R (*major*) = 536.07 min (97.51%), e.r. = 97.5:2.5.

(*S*,*E*)-3-heptylidenecyclohexan-1-ol (2c)

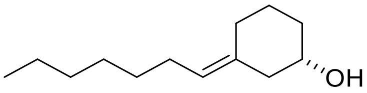

The reaction was performed at –20 °C for 16 h. Colorless oil (40.0 mg, 69% yield).

$\mathbf{R}_f$ (pentane:EtOAc 5:1) = 0.37.

^{1}H NMR (600 MHz, $CDCl_3$) δ 5.17 (tt, *J* = 7.3, 1.2 Hz, 1H), 3.72 (tt, *J* = 7.9, 3.8 Hz, 1H), 2.44–2.39 (m, 1H), 2.20 (dddd, *J* = 11.7, 7.3, 4.4, 0.9 Hz, 1H), 2.04 (dddd, *J* = 12.7, 8.2, 1.4, 0.7 Hz, 1H), 2.02–1.97 (m, 2H), 1.97–1.91 (m, 1H), 1.88–1.81 (m, 1H), 1.71 (dtt, *J* = 13.6, 6.9, 4.1 Hz, 1H), 1.50 (dtd, *J* = 12.6, 8.7, 3.9 Hz, 1H), 1.35 (dtt, *J* = 13.1, 9.1, 3.9 Hz, 1H), 1.32–1.22 (m, 8H), 0.88 (t, *J* = 7.0 Hz, 3H).

^{13}C NMR (151 MHz, $CDCl_3$) δ 135.4, 125.4, 70.4, 45.8, 34.9, 31.9, 30.2, 29.1, 27.8, 27.5, 23.6, 22.8, 14.2.

HRMS (CI) *m/z* for $C_{13}H_{28}N_1O$ $[M+NH_4]^+$: 214.216539, found: 214.216380.

$[\alpha]_D^{20}$ = +8.0 (*c* = 0.58, $CHCl_3$).

Chiral GC (BGB-176/BGB-15; Temperature program: 220/110 175 min iso 8/min 240/350): t_R (*minor*) = 134.46 min (2.12%), t_R (*major*) = 141.56 min (97.88%), e.r. = 98:2.

(*S*,*E*)-3-(3-methylbutylidene)cyclohexan-1-ol (2d)

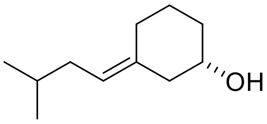

The reaction was performed at –20 °C for 16 h. Colorless oil (46.3 mg, 89% yield).

$\mathbf{R}_f$ (pentane:EtOAc 5:1) = 0.37.

^{1}H NMR (501 MHz, CD_2Cl_2) δ 5.18 (tt, *J* = 7.4, 1.3 Hz, 1H), 3.65 (dq, *J* = 8.7, 4.3 Hz, 1H), 2.41 (dd, *J* = 12.5, 4.1 Hz, 1H), 2.30–2.19 (m, 1H), 2.07–1.96 (m, 1H), 1.93–1.86 (m, 3H), 1.75–1.64 (m, 1H), 1.61–1.53 (m, 2H), 1.48–1.40 (m, 1H), 1.36–1.28 (m, 1H), 0.88 (dd, *J* = 6.6, 3.1 Hz, 6H).

^{13}C NMR (126 MHz, CD_2Cl_2) δ 136.7, 123.9, 70.7, 46.3, 36.9, 35.4, 29.3, 28.1, 23.9, 22.5, 22.4.

HRMS (EI) *m/z* for $C_{11}H_{20}O$ $[M]^+$: 168.150865, found: 168.150770.

$[\alpha]_D^{20}$ = +9.2 (*c* = 0.56, $CHCl_3$).

Chiral GC (Hydrodex-gamma-TBDAc; Temperature program: 220/60 400 min iso 8/min 230, 3 min iso/350): t_R (*minor*) = 271.67 min (2.85%), t_R (*major*) = 282.46 min (97.15%), e.r. = 97:3.

(*S*,*E*)-3-(but-3-en-1-ylidene)cyclohexan-1-ol (2e)

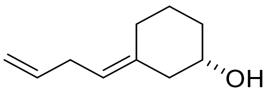

The reaction was performed at –20 °C for 16 h. Colorless oil (40.0 mg, 85% yield).

R*f* (pentane:EtOAc 5:1) = 0.30.

¹H NMR (501 MHz, CD_2Cl_2) δ 5.80 (ddt, *J* = 16.4, 10.1, 6.1 Hz, 1H), 5.19 (tt, *J* = 7.4, 1.3 Hz, 1H), 5.01 (dq, *J* = 17.1, 1.8 Hz, 1H), 4.94 (dq, *J* = 10.1, 1.6 Hz, 1H), 3.67 (tt, *J* = 8.3, 3.9 Hz, 1H), 2.84–2.69 (m, 2H), 2.43 (dd, *J* = 12.7, 4.1 Hz, 1H), 2.29–2.21 (m, 1H), 2.03 (ddd, *J* = 12.6, 8.5, 1.3 Hz, 1H), 1.95–1.81 (m, 2H), 1.78–1.67 (m, 1H), 1.51–1.38 (m, 1H), 1.39–1.28 (m, 1H).

¹³C NMR (126 MHz, CD_2Cl_2) δ 137.9, 137.6, 121.7, 114.4, 70.7, 46.0, 35.3, 32.0, 28.0, 23.9.

HRMS (ESI) *m/z* for $C_{10}H_{16}Na_1O$ $[M+Na]^+$: 175.10934, found: 175.10945.

$[\boldsymbol{\alpha}]_{\boldsymbol{D}}^{\mathbf{20}}$ = +5.3 (*c* = 0.68, $CHCl_3$).

Chiral HPLC (150 mm, OZ-3, 4.6 mm; n-Heptane/2-Propanol = 99.5:0.5; Flow rate = 1 mL/min; λ = 220 nm): t_R (*major*) = 12.33 min (96.33%), t_R (*minor*) = 13.09 min (3.67%), e.r. = 96.5:3.5.

(*S*,*E*)-3-(pent-4-en-1-ylidene)cyclohexan-1-ol (2f)

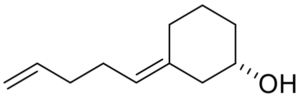

The reaction was performed at –20 °C for 24 h with 2 mol% catalyst loading. Colorless oil (29.8 mg, 60% yield).

R*f* (pentane:EtOAc 5:1) = 0.37.

¹H NMR (501 MHz, CD_2Cl_2) δ 5.87–5.77 (m, 1H), 5.21–5.12 (m, 1H), 5.01 (dq, *J* = 17.2, 1.6 Hz, 1H), 4.97–4.92 (m, 1H), 3.65 (tt, *J* = 8.2, 3.7 Hz, 1H), 2.40 (dd, *J* = 12.7, 4.0 Hz, 1H), 2.29–2.19 (m, 1H), 2.14–2.06 (m, 4H), 2.03–1.98 (m, 1H), 1.96–1.90 (m, 1H), 1.86–1.80 (m, 1H), 1.74–1.66 (m, 1H), 1.47–1.41 (m, 1H), 1.36–1.29 (m, 1H).

¹³C NMR (126 MHz, CD_2Cl_2) δ 139.1, 136.6, 124.2, 114.7, 70.5, 46.0, 35.3, 34.6, 28.1, 27.3, 23.9.

HRMS (CI) *m/z* for $C_{11}H_{22}N_1O$ $[M+NH_4]^+$: 184.169588, found: 184.169660.

$[\boldsymbol{\alpha}]_{\boldsymbol{D}}^{\mathbf{20}}$ = +14.5 (*c* = 0.47, $CHCl_3$).

Chiral GC (Hydrodex-gamma-TBDAc; Temperature program: 220/70 300 min iso 8/min 240, 3 min iso/350): t_R (*minor*) = 221.14 min (3.12%), t_R (*major*) = 229.11 min (96.88%), e.r. = 97:3.

(*S*,*E*)-3-(2-phenylethylidene)cyclohexan-1-ol (2g)

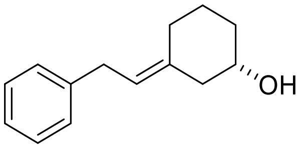

The reaction was performed at –40 °C for 24 h. White solid (49.6 mg, 77% yield).

R*f* (pentane:EtOAc 5:1) = 0.27.

¹H NMR (501 MHz, CD_2Cl_2) δ 7.31–7.24 (m, 2H), 7.21–7.13 (m, 3H), 5.36 (tt, *J* = 7.5, 1.3 Hz, 1H), 3.70 (tt, *J* = 8.3, 3.9 Hz, 1H), 3.44–3.32 (m, 2H), 2.49–2.36 (m, 2H), 2.09–1.99 (m, 2H), 1.93–1.86 (m, 1H), 1.83–1.74 (m, 1H), 1.53–1.45 (m, 1H), 1.43–1.35 (m, 1H).

¹³C NMR (126 MHz, CD_2Cl_2) δ 142.1, 137.3, 128.7, 128.7, 126.1, 123.4, 70.7, 46.1, 35.4, 34.0, 28.1, 24.0.

HRMS (EI) *m/z* for $C_{14}H_{18}O$ $[M]^+$: 202.135215, found: 202.135120.

$[\alpha]_D^{20}$ = +1.0 (*c* = 0.61, $CHCl_3$).

Chiral HPLC (150 mm, OJ-3R, 4.6 mm; CH_3CN/H_2O = 40:60; Flow rate = 1 mL/min; λ = 220 nm): t_R (*minor*) = 10.79 min (3.89%), t_R (*major*) = 12.63 min (96.11%), e.r. = 96:4.

(*S*,*E*)-3-(2-(*p*-tolyl)ethylidene)cyclohexan-1-ol (2h)

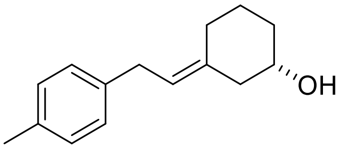

The reaction was performed at –20 °C for 16 h. White solid (56.4 mg, 86% yield).

R*f* (pentane:EtOAc 5:1) = 0.33.

¹H NMR (501 MHz, CD_2Cl_2) δ 7.07 (q, *J* = 8.0 Hz, 4H), 5.38–5.33 (m, 1H), 3.69 (tt, *J* = 8.6, 3.8 Hz, 1H), 3.32 (dd, *J* = 7.5, 3.1 Hz, 2H), 2.51–2.34 (m, 2H), 2.30 (s, 3H), 2.08–1.98 (m, 2H), 1.92–1.85 (m, 1H), 1.81–1.73 (m, 1H), 1.53–1.33 (m, 2H).

¹³C NMR (126 MHz, CD_2Cl_2) δ 138.9, 137.0, 135.7, 129.4, 128.5, 123.7, 70.7, 46.1, 35.4, 33.6, 28.1, 24.0, 21.1.

HRMS (EI) *m/z* for $C_{15}H_{20}O$ $[M]^+$: 216.150865, found: 216.150850.

$[\alpha]_D^{20}$ = +0.4 (*c* = 0.54, $CHCl_3$).

Chiral HPLC (150 mm, OD-3, 4.6 mm; n-Heptane/2-Propanol = 99:1; Flow rate = 1 mL/min; λ = 220 nm): t_R (*minor*) = 15.54 min (6.43%), t_R (*major*) = 16.34 min (93.57%), e.r. = 93.5:6.5.

(*S*)-3-(propan-2-ylidene)cyclohexan-1-ol (2i)

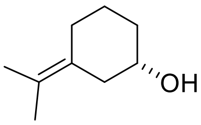

The reaction was performed at –60 °C for 24 h. Colorless oil (31.0 mg, 67% yield).

R*f* (pentane:EtOAc 5:1) = 0.40.

¹H NMR (501 MHz, CD_2Cl_2) δ 3.68–3.53 (m, 1H), 2.63 (dd, *J* = 13.0, 3.9 Hz, 1H), 2.39–2.26 (m, 1H), 1.99–1.92 (m, 1H), 1.91–1.81 (m, 2H), 1.73–1.66 (m, 7H), 1.45–1.37 (m, 1H), 1.35–1.25 (m, 1H).

¹³C NMR (126 MHz, CD_2Cl_2) δ 128.8, 124.0, 70.6, 39.7, 35.6, 29.7, 24.2, 20.3, 20.2.

HRMS (EI) *m/z* for $C_9H_{16}O$ $[M]^+$: 140.119565, found: 140.119690.

$[\alpha]_D^{20}$ = +13.0 (*c* = 0.48, $CHCl_3$).

Chiral GC (Cyclosil B; Temperature program: 220/80 1/min 220, 5 min iso/350): t_R (*minor*) = 49.01 min (4.75%), t_R (*major*) = 49.36 min (95.25%), e.r. = 95:5.

(*S*)-3-cyclopentylidenecyclohexan-1-ol (2j)

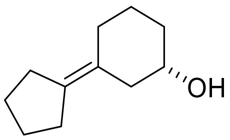

The reaction was performed at –60 °C for 24 h. Colorless oil (38.9 mg, 76% yield).

R*f* (pentane:EtOAc 5:1) = 0.33.

^{1}H NMR (501 MHz, CD_2Cl_2) δ 3.66–3.58 (m, 1H), 2.52 (dd, *J* = 12.9, 4.1 Hz, 1H), 2.25–2.15 (m, 5H), 1.99–1.80 (m, 3H), 1.74–1.67 (m, 1H), 1.65–1.60 (m, 4H), 1.42–1.27 (m, 2H).

^{13}C NMR (126 MHz, CD_2Cl_2) δ 136.4, 125.8, 70.4, 41.1, 35.5, 31.0, 30.5, 27.1, 24.0.

HRMS (EI) *m/z* for $C_{11}H_{18}O$ $[M]^+$: 166.135215, found: 166.135470.

$[\alpha]_D^{20}$ = +12.5 (*c* = 0.53, $CHCl_3$).

Chiral GC (Lipodex-G; Temperature program: 220/65 860 min iso 8/min 220/350): t_R (*major*) = 458.24 min (91.74%), t_R (*minor*) = 539.02 min (8.26%), e.r. = 91.5:8.5.

(*S*)-[1,1'-bi(cyclohexylidene)]-3-ol (2k)

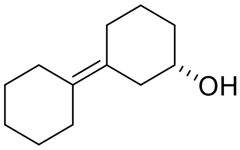

The reaction was performed at –40 °C for 24 h. White solid (30.0 mg, 53% yield).

R*f* (pentane:EtOAc 5:1) = 0.33.

^{1}H NMR (501 MHz, $CDCl_3$) δ 3.68 (tt, *J* = 8.0, 3.7 Hz, 1H), 2.65 (dd, *J* = 12.9, 3.7 Hz, 1H), 2.30 (ddd, *J* = 13.5, 6.7, 3.9 Hz, 1H), 2.23–2.17 (m, 4H), 2.11–2.03 (m, 1H), 2.00–1.92 (m, 1H), 1.91–1.84 (m, 1H), 1.74–1.65 (m, 1H), 1.56–1.46 (m, 7H), 1.39–1.28 (m, 1H).

^{13}C NMR (126 MHz, $CDCl_3$) δ 133.7, 125.2, 70.6, 38.9, 35.4, 30.6, 30.4, 29.2, 28.94, 28.87, 27.2, 24.5.

HRMS (EI) *m/z* for $C_{12}H_{20}O$ $[M]^+$: 180.150865, found: 180.150930.

$[\alpha]_D^{20}$ = +8.7 (*c* = 0.46, $CHCl_3$).

Chiral GC (Hydrodex-gamma DiMOM; Temperature program: 220/80 770 min iso 8/min 240/350): t_R (*major*) = 418.67 min (91.04%), t_R (*minor*) = 439.26 min (8.96%), e.r. = 91:9.

(*S,E*)-3-(2-methylpropylidene)cyclohexan-1-ol (2l)

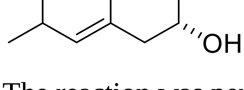

The reaction was performed at –60 °C for 24 h. Colorless oil (42.2 mg, 87% yield).

R*f* (pentane:EtOAc 5:1) = 0.33.

^{1}**H NMR** (501 MHz, CD_2Cl_2) δ 4.99 (dt, *J* = 9.0, 1.3 Hz, 1H), 3.69–3.56 (m, 1H), 2.63–2.49 (m, 1H), 2.42–2.32 (m, 1H), 2.25 (dt, *J* = 13.4, 5.2 Hz, 1H), 2.01–1.81 (m, 3H), 1.76–1.67 (m, 1H), 1.49–1.29 (m, 2H), 0.93 (dd, *J* = 6.7, 5.3 Hz, 6H).

^{13}**C NMR** (126 MHz, CD_2Cl_2) δ 134.0, 133.0, 70.7, 46.1, 35.4, 28.3, 27.0, 24.2, 23.7, 23.7.

HRMS (ESI) *m/z* for $C_{10}H_{18}Na_1O$ $[M+Na]^+$: 177.124984, found: 177.125080.

$[\alpha]_D^{20}$ = +6.9 (*c* = 0.61, $CHCl_3$).

Chiral GC (Cyclodextrin-H; Temperature program: 220/85 40 min iso 8/min 180, 3 min iso/ 350): t_R (*minor*) = 16.03 min (8.26%), t_R (*major*) = 17.22 min (91.74%), e.r. = 91.5:8.5.

(*S*,*E*)-3-(cyclohexylmethylene)cyclohexan-1-ol (2m)

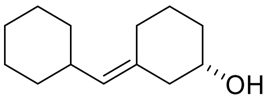

The reaction was performed at –60 °C for 24 h. Colorless oil (32.7 mg, 56% yield).

$\mathbf{R}_f$ (pentane:EtOAc 5:1) = 0.33.

^{1}**H NMR** (501 MHz, CD_2Cl_2) δ 5.00 (d, *J* = 8.9 Hz, 1H), 3.72–3.51 (m, 1H), 2.36 (dd, *J* = 12.6, 4.1 Hz, 1H), 2.27–2.16 (m, 2H), 1.98–1.81 (m, 3H), 1.73–1.59 (m, 5H), 1.47–1.39 (m, 1H), 1.32–1.22 (m, 4H), 1.20–1.13 (m, 1H), 1.09–0.99 (m, 2H).

^{13}**C NMR** (126 MHz, CD_2Cl_2) δ 134.4, 131.6, 70.7, 46.1, 36.8, 35.4, 34.2, 34.1, 28.4, 26.5, 24.3.

HRMS (EI) *m/z* for $C_{13}H_{22}O$ $[M]^+$: 194.166515, found: 194.166580.

$[\alpha]_D^{20}$ = +5.8 (*c* = 0.58, $CHCl_3$).

Chiral GC (lvadex-7/PS086; Temperature program: 220/100 170 min iso 8/min 230/350): t_R (*minor*) = 129.69 min (8.16%), t_R (*major*) = 138.41 min (91.84%), e.r. = 92:8.

(*S*,*E*)-3-(3-methoxypropylidene)cyclohexan-1-ol (2n)

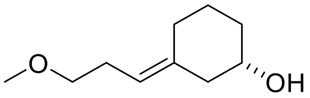

The reaction was performed at –20 °C for 16 h. Colorless oil (50.5 mg, 93% yield).

$\mathbf{R}_f$ (pentane:EtOAc 2:1) = 0.27.

^{1}**H NMR** (501 MHz, $CDCl_3$) δ 5.17 (t, *J* = 7.1 Hz, 1H), 3.73 (tt, *J* = 7.9, 3.7 Hz, 1H), 3.34 (d, *J* = 9.9 Hz, 5H), 2.43 (dd, *J* = 12.7, 3.9 Hz, 1H), 2.34–2.27 (m, 2H), 2.24–2.18 (m, 1H), 2.09–2.04 (m, 1H), 2.02–1.93 (m, 2H), 1.88–1.81 (m, 1H), 1.55–1.47 (m, 1H), 1.41–1.30 (m, 1H).

^{13}**C NMR** (126 MHz, $CDCl_3$) δ 137.8, 120.8, 72.7, 70.2, 58.7, 45.7, 34.8, 28.1, 28.0, 23.5.

HRMS (CI) *m/z* for $C_{10}H_{22}N_1O_2$ $[M+NH_4]^+$: 188.164504, found: 188.164450.

$[\alpha]_D^{20}$ = +2.7 (*c* = 0.44, $CHCl_3$).

Chiral GC (Cyclodextrin-H; Temperature program: 220/70 350 min, 8/min 180, 3 min iso/350): t_R (*minor*) = 210.97 min (1.80%), t_R (*major*) = 214.60 min (98.20%), e.r. = 98:2.

(*S*,*E*)-3-(4-(thiophen-2-yl)butylidene)cyclohexan-1-ol (2o)

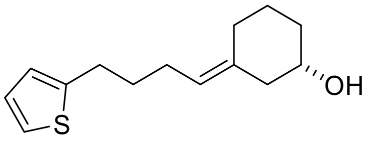

The reaction was performed at –20 °C for 16 h. Colorless oil (57.3 mg, 81% yield).

R*f* (pentane:EtOAc 5:1) = 0.28.

^{1}H NMR (501 MHz, $CDCl_3$) δ 7.11 (dd, *J* = 5.1, 1.2 Hz, 1H), 6.91 (dd, *J* = 5.1, 3.4 Hz, 1H), 6.81–6.74 (m, 1H), 5.19 (t, *J* = 7.3 Hz, 1H), 3.73 (tt, *J* = 8.0, 3.8 Hz, 1H), 2.82 (t, *J* = 7.6 Hz, 2H), 2.44 (dd, *J* = 12.7, 4.0 Hz, 1H), 2.24–2.17 (m, 1H), 2.13–2.03 (m, 3H), 1.97–1.82 (m, 2H), 1.76–1.69 (m, 3H), 1.55–1.45 (m, 1H), 1.41–1.30 (m, 1H).

^{13}C NMR (126 MHz, $CDCl_3$) δ 145.6, 136.4, 126.8, 124.3, 124.2, 123.0, 70.4, 45.8, 35.0, 32.1, 29.5, 27.9, 26.9, 23.6.

HRMS (EI) *m/z* for $C_{14}H_{20}O_1S_1$ $[M]^+$: 236.122937, found: 236.123040.

$[\alpha]_D^{20}$ = +5.7 (*c* = 0.42, $CHCl_3$).

Chiral HPLC (150 mm, IB-3, 4.6 mm; n-Heptane/2-Propanol = 99:1; Flow rate = 1 mL/min; λ = 220 nm): t_R (*major*) = 13.24 min (96.94%), t_R (*minor*) = 15.62 min (3.06%), e.r. = 97:3.

(*S*,*E*)-3-(4-hydroxybutylidene)cyclohexan-1-ol (2p)

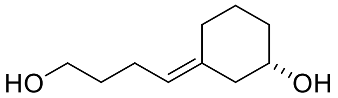

The reaction was performed at –20 °C for 16 h. Colorless oil (22.0 mg, 40% yield).

R*f* (pentane:EtOAc 1:2) = 0.32.

^{1}H NMR (501 MHz, $CDCl_3$) δ 5.19 (t, *J* = 7.4 Hz, 1H), 3.73 (tt, *J* = 7.9, 3.7 Hz, 1H), 3.63 (td, *J* = 6.4, 1.6 Hz, 2H), 2.41 (dd, *J* = 12.7, 3.9 Hz, 1H), 2.26–2.17 (m, 1H), 2.14–2.02 (m, 3H), 2.01–1.94 (m, 1H), 1.89–1.81 (m, 1H), 1.74–1.69 (m, 1H), 1.61 (p, *J* = 6.8 Hz, 2H), 1.54–1.44 (m, 1H), 1.42–1.29 (m, 1H).

^{13}C NMR (126 MHz, $CDCl_3$) δ 136.4, 124.3, 70.3, 62.7, 45.6, 34.8, 33.0, 27.8, 24.0, 23.5.

HRMS (ESI) *m/z* for $C_{10}H_{18}Na_1O_2$ $[M+Na]^+$: 193.11990, found: 193.11995.

$[\alpha]_D^{20}$ = +11.7 (*c* = 0.65, $CHCl_3$).

Chiral GC (Hydrodex-gamma-TBDAc; Temperature program: 220/100 450 min, iso 8/min 250/350): t_R (*minor*) = 340.77 min (3.86%), t_R (*major*) = 352.53 min (96.14%), e.r. = 96:4.

ethyl (*S*,*E*)-4-(3-hydroxycyclohexylidene)butanoate (2q)

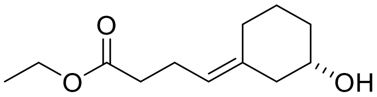

The reaction was performed at –20 °C for 16 h. Colorless oil (38.5 mg, 58% yield).

R*f* (pentane:EtOAc 2:1) = 0.33.

^{1}H NMR (501 MHz, $CDCl_3$) δ 5.12 (d, *J* = 7.7 Hz, 1H), 4.10 (q, *J* = 7.1 Hz, 2H), 3.70 (tt, *J* = 8.0, 3.8 Hz, 1H), 2.39 (dd, *J* = 12.7, 4.0 Hz, 1H), 2.34–2.27 (m, 4H), 2.25–2.18 (m, 1H), 2.02 (dd, *J* = 12.8, 8.2 Hz, 1H), 1.98–1.91 (m, 1H), 1.87–1.80 (m, 1H), 1.75–1.66 (m, 1H), 1.53–1.43 (m, 1H), 1.38–1.29 (m, 1H), 1.23 (t, *J* = 7.2 Hz, 3H).

^{13}C NMR (126 MHz, $CDCl_3$) δ 173.4, 137.2, 122.7, 70.2, 60.4, 45.6, 34.8, 27.8, 23.5, 23.2, 14.4.

HRMS (ESI) *m/z* for $C_{12}H_{20}Na_1O_3$ $[M+Na]^+$: 235.13047, found: 235.13032.

$[\alpha]_D^{20}$ = +13.8 (*c* = 0.48, $CHCl_3$).

Chiral GC (lvadex-7/PS086; Temperature program: 220/110 135 min, iso 8/min 230/350): t_R (*minor*) = 98.69 min (2.65%), t_R (*major*) = 104.56 min (97.35%), e.r. = 97.5:2.5.

(*S,E*)-3-(4-bromobutylidene)cyclohexan-1-ol (2r)

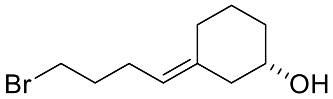

The reaction was performed at –20 °C for 16 h with 0.13 mmol aldehyde. Colorless oil (16.2 mg, 54% yield).

R*f* (pentane:EtOAc 5:1) = 0.21.

^{1}H NMR (501 MHz, $CDCl_3$) δ 5.12 (t, *J* = 7.4 Hz, 1H), 3.72 (tt, *J* = 8.1, 3.9 Hz, 1H), 3.40 (t, *J* = 6.6 Hz, 2H), 2.43 (dd, *J* = 12.8, 4.0 Hz, 1H), 2.31–2.23 (m, 1H), 2.22–2.14 (m, 2H), 2.09–2.02 (m, 1H), 2.00–1.91 (m, 1H), 1.94–1.83 (m, 3H), 1.80–1.69 (m, 1H), 1.55–1.45 (m, 1H), 1.42–1.31 (m, 1H).

^{13}C NMR (126 MHz, $CDCl_3$) δ 137.5, 122.8, 70.4, 45.7, 34.9, 33.6, 32.9, 27.9, 25.9, 23.6.

HRMS (ESI) *m/z* for $C_{10}H_{17}Br_1Na_1O$ $[M+Na]^+$: 255.035509, found: 255.035400.

$[\alpha]_D^{20}$ = +12.5 (*c* = 0.40, $CHCl_3$).

Chiral GC (lvadex-7/PS086; Temperature program: 220/120 65 min, iso 8/min 230/350): t_R (*minor*) = 46.41 min (3.33%), t_R (*major*) = 48.48 min (96.67%), e.r. = 96.5:3.5.

(*S*)-3-methylenecycloheptan-1-ol (2s)

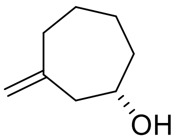

The reaction was performed at –40 °C for 24 h. Colorless oil (20.0 mg, 53% yield).

R*f* (pentane:EtOAc 5:1) = 0.33.

^{1}H NMR (501 MHz, CD_2Cl_2) δ 4.89–4.74 (m, 2H), 3.86–3.71 (m, 1H), 2.52 (dd, *J* = 13.2, 3.5 Hz, 1H), 2.38–2.22 (m, 3H), 1.79–1.71 (m, 2H), 1.62–1.59 (m, 2H), 1.57–1.50 (m, 1H), 1.43–1.33 (m, 1H).

^{13}C NMR (126 MHz, CD_2Cl_2) δ 146.4, 114.2, 70.7, 44.3, 39.2, 36.4, 27.5, 22.9.

HRMS (CI) *m/z* for $C_8H_{18}N_1O$ $[M+NH_4]^+$: 144.138289, found: 144.138440.

$[\alpha]_D^{20}$ = +23.3 (*c* = 0.29, $CHCl_3$).

Chiral GC (BGB-174/BGB-1701; Temperature program: 220/75 40 min iso 8/min 240/350): t_R (*minor*) = 33.58 min (3.27%), t_R (*major*) = 34.40 min (96.73%), e.r. = 96.5:3.5.

(*S*,*E*)-3-butylidenecycloheptan-1-ol (2t)

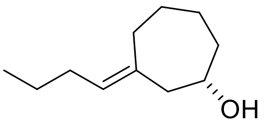

The reaction was performed at –20 °C for 16 h. Colorless oil (48.7 mg, 95% yield).

R*f* (pentane:EtOAc 5:1) = 0.40.

^{1}H NMR (501 MHz, CD_2Cl_2) δ 5.30–5.22 (m, 1H), 3.81–3.66 (m, 1H), 2.47 (dd, *J* = 13.2, 3.3 Hz, 1H), 2.33–2.15 (m, 3H), 2.02–1.93 (m, 2H), 1.73–1.68 (m, 1H), 1.63–1.47 (m, 4H), 1.43–1.31 (m, 3H), 0.90 (t, *J* = 7.4 Hz, 3H).

^{13}C NMR (126 MHz, CD_2Cl_2) δ 135.4, 129.7, 71.1, 45.9, 38.6, 30.4, 30.2, 26.6, 23.3, 23.3, 14.1.

HRMS (EI) *m/z* for $C_{11}H_{20}O$ $[M]^+$: 168.150865, found: 168.151020.

$[\alpha]_D^{20}$ = +15.2 (*c* = 0.63, $CHCl_3$).

Chiral GC (BGB-177/BGB-15; Temperature program: 220/90 185 min iso 8/min 230/350): t_R (*minor*) = 160.11 min (1.80%), t_R (*major*) = 170.07 min (98.20%), e.r. = 98:2.

(*S*)-3,3-dimethyl-5-methylenecyclohexan-1-ol (2u)

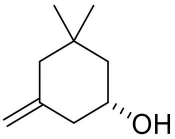

The reaction was performed at –20 °C for 16 h. Colorless oil (33.0 mg, 75% yield).

R*f* (pentane:EtOAc 5:1) = 0.33.

^{1}H NMR (501 MHz, CD_2Cl_2) δ 4.77 (t, *J* = 1.7 Hz, 1H), 4.67 (dq, *J* = 2.6, 1.4 Hz, 1H), 3.73 (tt, *J* = 10.7, 4.6 Hz, 1H), 2.59–2.51 (m, 1H), 1.90–1.81 (m, 3H), 1.70 (ddt, *J* = 12.6, 3.8, 1.7 Hz, 1H), 1.23–1.17 (m, 1H), 0.99 (s, 3H), 0.82 (s, 3H).

^{13}C NMR (126 MHz, CD_2Cl_2) δ 145.4, 110.8, 68.5, 48.6, 47.9, 44.9, 32.8, 32.1, 25.8.

HRMS (ESI) *m/z* for $C_9H_{16}Na_1O$ $[M+Na]^+$: 163.10934, found: 163.10932.

$[\alpha]_D^{20}$ = –3.8 (*c* = 0.57, $CHCl_3$).

Chiral GC (lvadex-7/PS086; Temperature program: 220/60 180 min iso 8/min 230, 3 min iso/350): t_R (*minor*) = 85.74 min (2.36%), t_R (*major*) = 94.45 min (97.64%), e.r. = 97.5:2.5.

(*R*)-3-methylene-1,2,3,4-tetrahydronaphthalen-1-ol (2v)

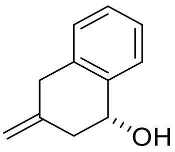

The reaction was performed at –20 °C for 48 h with 1.5 mol% catalyst loading. White solid (14.8 mg, 29% yield).

R$_f$ (pentane:EtOAc 5:1) = 0.37.

^{1}H NMR (501 MHz, $CDCl_3$) δ 7.42 (dd, *J* = 7.2, 1.9 Hz, 1H), 7.25 - 7.19 (m, 2H), 7.13 (dd, *J* = 7.5, 1.6 Hz, 1H), 5.07 (s, 1H), 5.00 (s, 1H), 4.86-4.80 (m, 1H), 3.57 (s, 2H), 2.73 (dd, *J* = 13.2, 4.3 Hz, 1H), 2.62 (dd, *J* = 13.1, 4.8 Hz, 1H).

^{13}C NMR (126 MHz, $CDCl_3$) δ 141.0, 138.6, 136.1, 129.0, 128.7, 128.2, 126.5, 112.2, 69.7, 41.1, 37.2.

HRMS (EI) *m/z* for $C_{11}H_{12}O$ $[M]^+$: 160.088265, found: 160.088470.

$[\alpha]_D^{20}$ = +10.7 (*c* = 0.30, $CHCl_3$).

Chiral HPLC (150 mm, AD-3R, 4.6 mm; CH_3CN/H_2O = 50:50; Flow rate = 1 mL/min; λ = 220 nm): t_R (*minor*) = 4.77 min (2.16%), t_R (*major*) = 5.41 min (97.84%), e.r. = 98:2.

6. Limitations of the Method

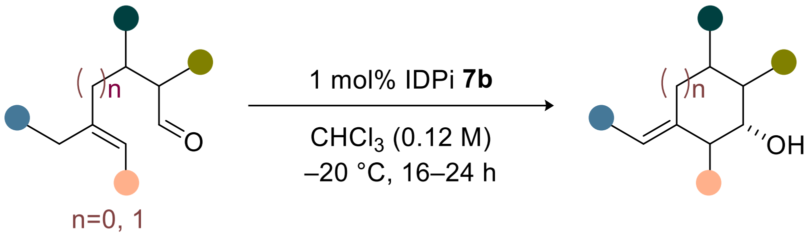

1 **2**

IDPi catalyst **7b** (1 mol%) was dissolved in 0.2 mL anhydrous chloroform and if desired cooled down to the desired reaction temperature. Aldehyde **1** (0.024 mmol, 1 equiv.) was added in one portion and the reaction was stirred for 16–24 h. The reaction was quenched with one drop of trimethylamine, followed by the addition of mesitylene (2 µL, 0.014 mmol, 0.6 equiv.) as an internal standard. The composition of the reaction mixture was analyzed via ^{1}H NMR spectroscopy of an aliquot in $CDCl_3$. The crude reaction mixture was then directly purified by preparative thin layer chromatography to furnish the chiral product. Chiral GC analysis was performed to give the corresponding enantiomeric ratio.

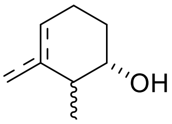

full conv.[a]
90% yield
exo:*endo* = 60:40
exo:*trans*:*cis* = 1:1
endo:*trans*:*cis* = 1:1
exo-trans: 72.5:27.5 e.r.
exo-cis: 51:49 e.r.
endo-trans: 83:17 e.r.
endo-cis: 57:43 e.r.

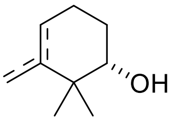

full conv.
96% yield
exo:*endo* = 35:65
exo: 51.5:48.5 e.r.
endo: 59.5:40.5 e.r.

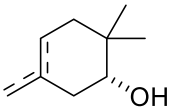

full conv.[b]
87% yield
exo:*endo* = 1:1
exo: 71:29 e.r.
endo: 65:35 e.r.

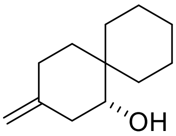

full conv.[b]
70% yield
68:32 e.r.

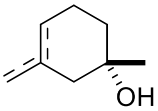

full conv.
86% yield
exo:*endo* = 9:1
exo: 84:16 e.r.
endo: 41:59 e.r.

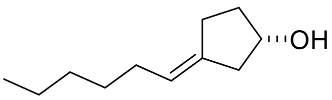

no reaction

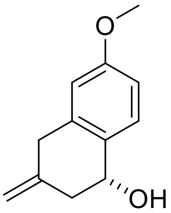

full conv.[c]
0% yield

Figure S1. *Limitions of the method.* [a] *Using (E/Z)-5-methylhept-5-enal mixtures with 1:1 ratio;* [b]*At –60 °C for 24 h.* [c]*The side product is the aromatized ene product 2-methoxy-7-methylnaphthalene.*

7. Derivatization

(*S*)-spiro[2.6]nonan-5-ol (8)

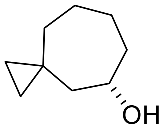

The reactions was modified from a literature method.[12] A solution of Et_2Zn (2 mL, 1.0 M in hexane, 2 mmol, 5.00 equiv.) in anhydrous DCM (4 mL) was cooled to –20 °C, then diiodomethane (0.26 mL, 3.2 mmol, 8.00 equiv.) was very carefully added over 10 min, during this time, voluminous white precipitate was gradually formed, then stirred for a further 10 min at the same temperature. A solution of (*S*)-3-methylenecycloheptan-1-ol (**2s**) (50 mg, 0.40 mmol, 1.00 equiv.) was very carefully added, then stirred for a further 30 min at –20 to –10 °C. The reaction was warmed to r.t. and stirred overnight. The reaction was quenched by the addition of saturated aqueous solution of NH_4Cl, then diluted with H_2O. The layers were separated, the aqueous layer was extracted with DCM, and the combined organics dried over Na_2SO_4 and concentrated under reduced pressure. Purification by CC (silica gel, Pentane/Et_2O, 5:1) afforded the desired product **8** as a colourless oil (36 mg, 65% yield).

^{1}H NMR (501 MHz, $CDCl_3$) δ 3.92–3.85 (m, 1H), 2.00–1.91 (m, 1H), 1.81 (ddd, J = 13.9, 9.0, 1.0 Hz, 1H), 1.73–1.61 (m, 3H), 1.58–1.52 (m, 3H), 1.48–1.45 (m, 1H), 1.10–1.12 (m, 1H), 0.38–0.33 (m, 2H), 0.32–0.24 (m, 2H).

^{13}C NMR (126 MHz, $CDCl_3$) δ 72.1, 47.3, 38.2, 37.8, 26.7, 23.1, 16.5, 15.0, 12.7.

HRMS (ESI) *m/z* for $C_9H_{15}O$ $[M-H]^-$: 139.112840, found: 139.112850.

$[\alpha]_D^{20}$ = –1.8 (*c* = 0.56, $CHCl_3$).

Chiral GC (Hydrodex-beta-TBDAc-CD; Temperature program: 220/75 75 min iso 8/min 220, 2 min iso/350): t_R (*minor*) = 64.51 min (3.61%), t_R (*major*) = 68.47 min (96.39%), e.r. = 96.5:3.5.

(*S,E*)-3-(2-phenylethylidene)cyclohexyl ferrocenecarboxylate (9)

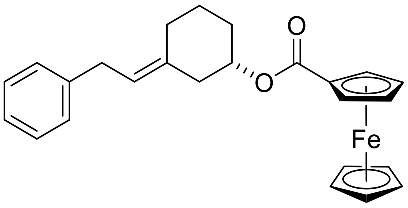

The reaction was modified from a literature method.[13] A oven dried 25 mL flask under Ar was charged with (*S,E*)-3-(2-phenylethylidene)cyclohexan-1-ol (**2g**) (15 mg, 0.074 mmol, 1.00 equiv.), ferrocenecarboxylic acid (22 mg, 0.096 mmol, 1.30 equiv.) and 4-dimethylaminopyridine (DMAP, 25 mg, 0.205 mmol, 2.80 equiv.) followed by addition of anhydrous DCM (5 mL). The reaction mixture was cooled to 0 °C and 1-ethyl-3-(3-dimethyllaminopropyl) carbodiimide hydrochloride (EDAC, 25 mg, 0.13 mmol, 1.80 equiv.) was added. The mixture was allowed to r.t. and stirred overnight. The

reaction mixture was then diluted with saturated aqueous solution of $NaHCO_3$ and extracted with DCM. The combined organics were dried over Na_2SO_4 and concentrated under reduced pressure. The crude product was purified by CC (silica gel, Pentane/EtOAc, 30:1). The product was obtained as a yellow soild (25 mg, 81% yield).

^{1}H NMR (501 MHz, $CDCl_3$) δ 7.33–7.27 (m, 2H), 7.24–7.17 (m, 3H), 5.46–5.40 (m, 1H), 5.07–5.00 (m, 1H), 4.76 (t, *J* = 1.9 Hz, 2H), 4.36 (t, *J* = 2.0 Hz, 2H), 4.16 (s, 5H), 3.41 (tt, *J* = 15.4, 7.5 Hz, 2H), 2.55 (dd, *J* = 13.1, 4.1 Hz, 1H), 2.44–2.37 (m, 1H), 2.31 (dd, *J* = 13.1, 8.0 Hz, 1H), 2.24–2.17 (m, 1H), 2.03–1.96 (m, 1H), 1.90–1.83 (m, 1H), 1.78–1.71 (m, 1H), 1.60–1.50 (m, 1H).
^{13}C NMR (126 MHz, $CDCl_3$) δ 171.1, 141.5, 136.1, 128.6, 128.5, 126.0, 123.5, 72.1, 71.3, 70.33, 70.28, 69.9, 42.2, 33.8, 31.8, 27.9, 23.7.
HRMS (ESI) *m/z* for $C_{25}H_{26}Fe_1Na_1O_2$ $[M+Na]^+$: 437.11744, found: 437.11722.

(1*S*,3*S*)-3-butylcycloheptan-1-ol (10)

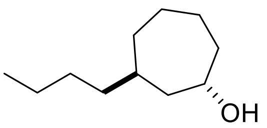

The reaction was modified from a literature method.[14] An oven dried 5 mL vial was charged with (*S*,*E*)-3-butylidenecycloheptan-1-ol (**2t**) (14.5 mg, 0.09 mmol, 1.00 equiv.), bis(pyridin)(1,5-cyclooctadien)iridium(I)hexafluorphosphate (10.4 mg, 0.017 mmol, 20 mol%) and tricyclohexylphosphine (4.9 mg, 0.017 mmol, 20 mol%) followed by addition of anhydrous DCM (2 mL). An atmosphere of hydrogen was introduced and the resulting suspension was stirred at r.t. for 3 h. The reaction mixture was concentrated under reduced pressure. The crude product was purified by CC (silica gel, Pentane/Et_2O, 5:1). The product was obtained as colorless oil (13.5 mg, 92% yield).

^{1}H NMR (501 MHz, CD_2Cl_2) δ 3.98–3.87 (m, 1H), 1.89–1.81 (m, 1H), 1.73–1.66 (m, 4H), 1.64–1.58 (m, 2H), 1.55–1.50 (m, 1H), 1.40–1.35 (m, 1H), 1.30–1.23 (m, 7H), 1.12–1.05 (m, 1H), 0.92–0.86 (m, 3H).
^{13}C NMR (126 MHz, CD_2Cl_2) δ 70.5, 43.7, 38.2, 38.0, 35.4, 33.6, 30.0, 28.7, 24.7, 23.4, 14.3.
HRMS (ESI) *m/z* for $C_{11}H_{22}Na_1O_1$ $[M+Na]^+$: 193.156284, found: 193.156440.
$[\boldsymbol{\alpha}]_{\boldsymbol{D}}^{\mathbf{20}}$ = –3.6 (*c* = 0.39, $CHCl_3$).
Chiral GC (lvadex-7/PS086; Temperature program: 220/80 150 min iso 8/min 230/350): t_R (*minor*) = 99.58 min (2.13%), t_R (*major*) = 100.70 min (97.87%), e.r. = 98:2.

8. Crystallographic Data

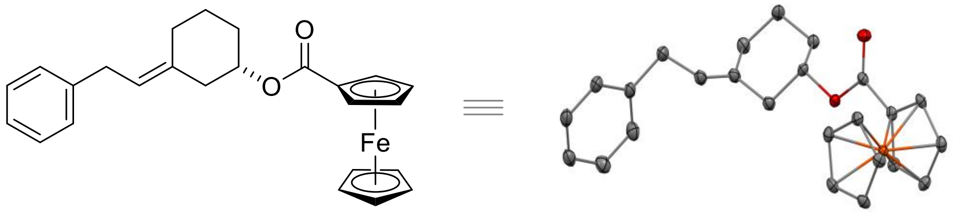

9 2470409

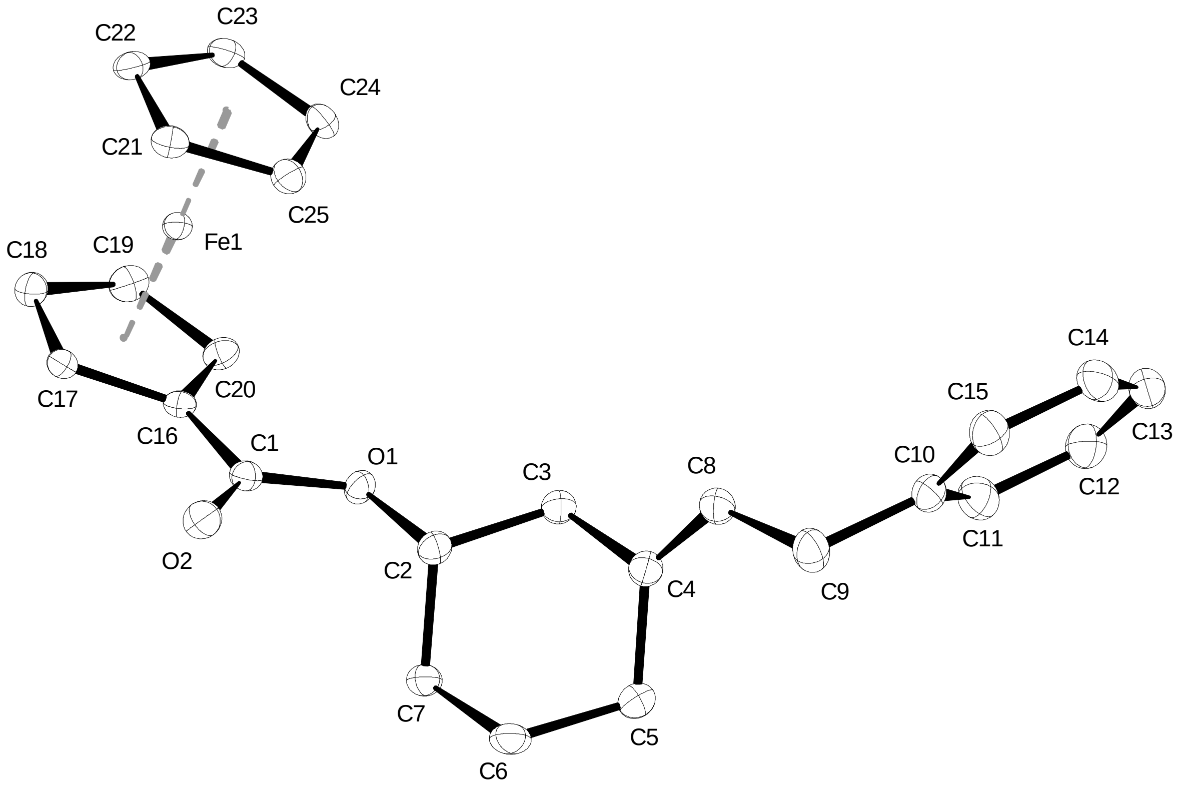

Table S2. Crystal data and structure refinement.

Identification code	15884	
Empirical formula	$C_{25} H_{26} Fe O_2$	
Color	intense yellow	
Formula weight	414.31 g · mol^{-1}	
Temperature	100(2) K	
Wavelength	0.71073 Å	
Crystal system	ORTHORHOMBIC	
Space group	**$P2_12_12_1$, (no. 19)**	
Unit cell dimensions	a = 5.7666(17) Å	α= 90°.
	b = 9.337(3) Å	β= 90°.
	c = 37.130(12) Å	γ = 90°.

Volume	1999.2(11) $Å^3$	
Z	4	
Density (calculated)	1.377 $Mg \cdot m^{-3}$	
Absorption coefficient	0.772 mm^{-1}	
F(000)	872 e	
Crystal size	0.121 x 0.105 x 0.071 mm^3	
θ range for data collection	2.194 to 31.062°.	
Index ranges	$-8 \le h \le 8$, $-13 \le k \le 13$, $-53 \le l \le 53$	
Reflections collected	151988	
Independent reflections	6402 [R_{int} = 0.1305]	
Reflections with I>2σ(I)	5996	
Completeness to θ = 25.242°	99.8 %	
Absorption correction	Numerical	
Max. and min. transmission	0.97 and 0.85	
Refinement method	Full-matrix least-squares on F^2	
Data / restraints / parameters	6402 / 0 / 258	
Goodness-of-fit on F^2	1.115	
Final R indices [I>2σ(I)]	R_1 = 0.0316	wR^2 = 0.0686
R indices (all data)	R_1 = 0.0358	wR^2 = 0.0702
Absolute structure parameter	0.012(14)	
Largest diff. peak and hole	0.3 and -0.6 $e \cdot Å^{-3}$	

Table S3. Bond lengths [Å] and angles [°].

Fe(1)-C(16)	2.025(2)	Fe(1)-C(17)	2.039(2)
Fe(1)-C(18)	2.054(2)	Fe(1)-C(19)	2.055(2)
Fe(1)-C(20)	2.036(2)	Fe(1)-C(21)	2.043(2)
Fe(1)-C(22)	2.040(2)	Fe(1)-C(23)	2.047(2)
Fe(1)-C(24)	2.052(2)	Fe(1)-C(25)	2.047(2)
O(1)-C(1)	1.340(3)	O(1)-C(2)	1.459(3)
O(2)-C(1)	1.214(3)	C(1)-C(16)	1.465(3)
C(2)-C(3)	1.515(3)	C(2)-C(7)	1.513(3)
C(2)-H(2)	0.99(3)	C(3)-C(4)	1.510(3)
C(4)-C(5)	1.503(3)	C(4)-C(8)	1.330(3)
C(5)-C(6)	1.523(3)	C(6)-C(7)	1.524(3)
C(8)-C(9)	1.504(3)	C(9)-C(10)	1.516(3)
C(10)-C(11)	1.390(4)	C(10)-C(15)	1.388(4)
C(11)-C(12)	1.382(4)	C(12)-C(13)	1.384(4)
C(13)-C(14)	1.383(4)	C(14)-C(15)	1.391(4)
C(16)-C(17)	1.428(3)	C(16)-C(20)	1.435(3)
C(17)-C(18)	1.418(3)	C(18)-C(19)	1.424(3)
C(19)-C(20)	1.415(3)	C(21)-C(22)	1.425(3)
C(21)-C(25)	1.429(3)	C(22)-C(23)	1.425(3)
C(23)-C(24)	1.416(3)	C(24)-C(25)	1.421(3)
C(16)-Fe(1)-C(17)	41.14(9)	C(16)-Fe(1)-C(18)	68.60(9)
C(16)-Fe(1)-C(19)	68.58(9)	C(16)-Fe(1)-C(20)	41.38(9)
C(16)-Fe(1)-C(21)	121.25(9)	C(16)-Fe(1)-C(22)	156.39(9)
C(16)-Fe(1)-C(23)	161.64(9)	C(16)-Fe(1)-C(24)	125.23(9)
C(16)-Fe(1)-C(25)	108.02(9)	C(17)-Fe(1)-C(18)	40.53(9)
C(17)-Fe(1)-C(19)	68.42(10)	C(17)-Fe(1)-C(21)	106.70(9)
C(17)-Fe(1)-C(22)	120.15(9)	C(17)-Fe(1)-C(23)	155.80(9)
C(17)-Fe(1)-C(24)	161.94(9)	C(17)-Fe(1)-C(25)	124.59(9)
C(18)-Fe(1)-C(19)	40.54(9)	C(20)-Fe(1)-C(17)	69.27(9)
C(20)-Fe(1)-C(18)	68.56(9)	C(20)-Fe(1)-C(19)	40.47(9)
C(20)-Fe(1)-C(21)	157.72(9)	C(20)-Fe(1)-C(22)	160.31(9)
C(20)-Fe(1)-C(23)	124.08(10)	C(20)-Fe(1)-C(24)	108.19(9)
C(20)-Fe(1)-C(25)	122.23(9)	C(21)-Fe(1)-C(18)	123.42(9)
C(21)-Fe(1)-C(19)	160.16(9)	C(21)-Fe(1)-C(23)	68.63(9)
C(21)-Fe(1)-C(24)	68.50(9)	C(21)-Fe(1)-C(25)	40.91(9)

C(22)-Fe(1)-C(18)	106.47(9)	C(22)-Fe(1)-C(19)	123.58(9)
C(22)-Fe(1)-C(21)	40.86(9)	C(22)-Fe(1)-C(23)	40.80(9)
C(22)-Fe(1)-C(24)	68.38(9)	C(22)-Fe(1)-C(25)	68.65(9)
C(23)-Fe(1)-C(18)	120.86(9)	C(23)-Fe(1)-C(19)	
107.48(10)	C(23)-Fe(1)-C(24)	40.43(9)	C(24)-
Fe(1)-C(18)	156.65(10)	C(24)-Fe(1)-C(19)	
121.99(10)	C(25)-Fe(1)-C(18)	160.83(9)	C(25)-
Fe(1)-C(19)	157.52(9)	C(25)-Fe(1)-C(23)	68.35(9)
C(25)-Fe(1)-C(24)	40.56(9)	C(1)-O(1)-C(2)	
116.16(17)	O(1)-C(1)-C(16)	112.62(18)	O(2)-
C(1)-O(1)	123.92(19)	O(2)-C(1)-C(16)	123.5(2)
O(1)-C(2)-C(3)	105.74(17)	O(1)-C(2)-C(7)	
111.55(18)	O(1)-C(2)-H(2)	105.1(17)	C(3)-
C(2)-H(2)	109.4(16)	C(7)-C(2)-C(3)	
111.07(19)	C(7)-C(2)-H(2)	113.6(17)	C(4)-
C(3)-C(2)	110.69(18)	C(5)-C(4)-C(3)	113.3(2)
C(8)-C(4)-C(3)	120.1(2)	C(8)-C(4)-C(5)	126.6(2)
C(4)-C(5)-C(6)	110.04(19)	C(5)-C(6)-C(7)	111.6(2)
C(2)-C(7)-C(6)	109.25(18)	C(4)-C(8)-C(9)	128.2(2)
C(8)-C(9)-C(10)	111.4(2)	C(11)-C(10)-C(9)	120.9(2)
C(15)-C(10)-C(9)	120.6(2)	C(15)-C(10)-C(11)	118.5(2)
C(12)-C(11)-C(10)	120.9(2)	C(11)-C(12)-C(13)	120.4(3)
C(14)-C(13)-C(12)	119.3(2)	C(13)-C(14)-C(15)	120.3(2)
C(10)-C(15)-C(14)	120.6(2)	C(1)-C(16)-Fe(1)	
121.79(14)	C(17)-C(16)-Fe(1)	69.96(12)	C(17)-
C(16)-C(1)	123.9(2)	C(17)-C(16)-C(20)	
107.98(18)	C(20)-C(16)-Fe(1)	69.72(12)	C(16)-
C(17)-Fe(1)	68.90(12)	C(18)-C(17)-Fe(1)	70.29(13)
C(18)-C(17)-C(16)	107.76(19)	C(17)-C(18)-Fe(1)	69.18(12)
C(17)-C(18)-C(19)	108.24(19)	C(19)-C(18)-Fe(1)	69.78(12)
C(18)-C(19)-Fe(1)	69.68(13)	C(20)-C(19)-Fe(1)	69.05(12)
C(20)-C(19)-C(18)	108.5(2)	C(16)-C(20)-Fe(1)	68.90(12)
C(19)-C(20)-Fe(1)	70.48(12)	C(19)-C(20)-C(16)	
107.52(19)	C(22)-C(21)-Fe(1)	69.47(12)	C(22)-
C(21)-C(25)	107.7(2)	C(25)-C(21)-Fe(1)	69.70(12)
C(21)-C(22)-Fe(1)	69.67(12)	C(23)-C(22)-Fe(1)	69.86(12)
C(23)-C(22)-C(21)	108.00(19)	C(22)-C(23)-Fe(1)	69.34(12)
C(24)-C(23)-Fe(1)	69.95(13)	C(24)-C(23)-C(22)	108.1(2)
C(23)-C(24)-Fe(1)	69.62(12)	C(23)-C(24)-C(25)	

108.32(19)	C(25)-C(24)-Fe(1)	69.55(12)	C(21)-
C(25)-Fe(1)	69.39(12)	C(24)-C(25)-Fe(1)	69.90(12)
C(24)-C(25)-C(21)	107.93(19)		

9. Mechanistic Experiments

9.1. Kinetic Study via ^{1}H NMR

Sample preparation and data acquisition

In an oven-dried (80 °C, overnight) 5 mm NMR tube, 0.25 mL IDPi **7b** solution (0.52 mg/mL in anhydrous $CDCl_3$, 0.1 mol%) and 0.25 mL anhydrous $CDCl_3$ was added. The NMR tube was cooled to –20 °C, then the distilled 5-methylhex-5-enal **1a** (0.0632 mmol, 9 µL) was added via a syringe. The NMR tube was turned upside down and vortexed for 10 s to ensure mixing of all the components. Afterwards, the sample was quickly transferred to a Bruker 500 MHz NMR magnet precooled to 253.0 K. After quick shimming, single scan ^{1}H NMR spectra were acquired every 1 min until full conversion was observed. The acquired NMR data was imported with the *Reaction Monitoring* plugin into MestReNova 16.0.0 and processed therein (baseline correction, phase correction, integration, fitting). Generally, the first acquired NMR spectrum was used as absolute concentration reference by using a characteristic signal of the starting material **1a** for the following spectra. The following **Figure S2** shows ^{1}H NMR spectra taken at different time points during the reaction at –20 °C in presence of IDPi **7b** (0.1 mol%).

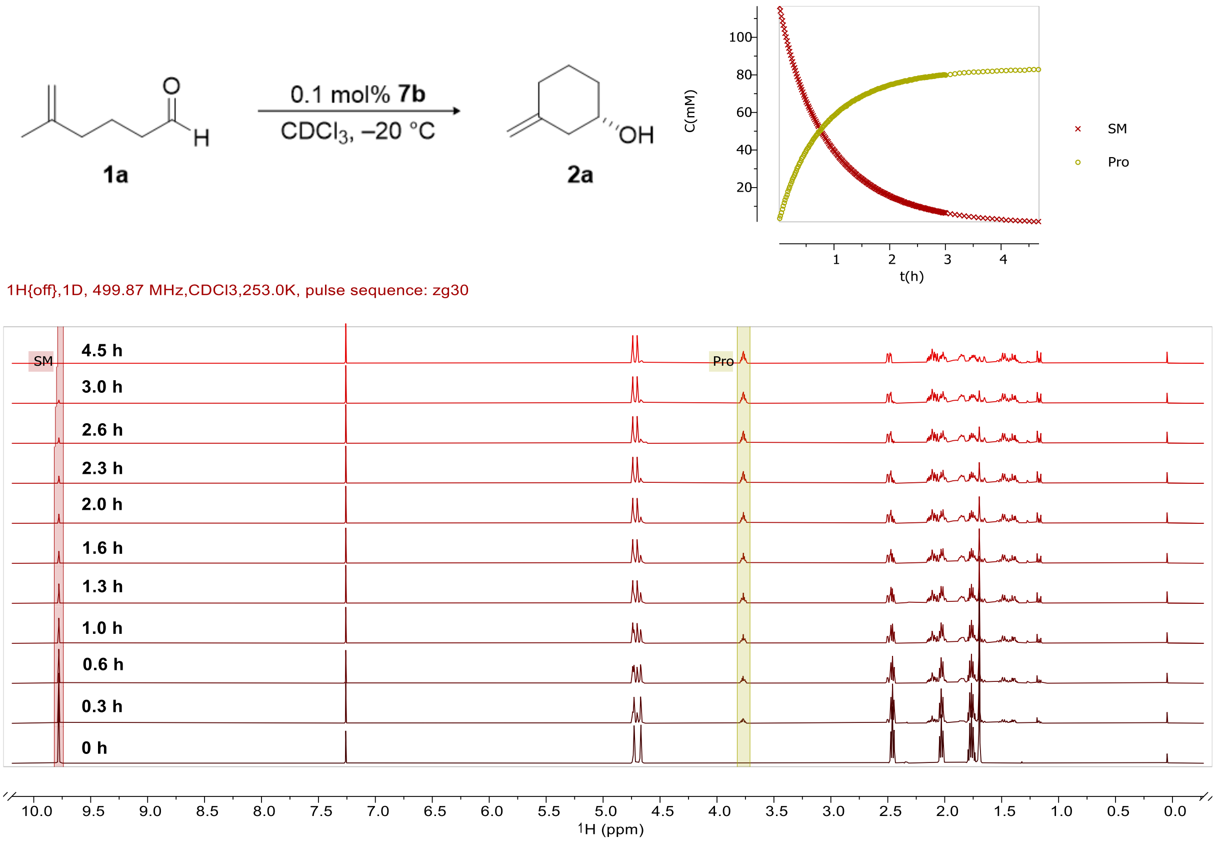

Figure S2. *^{1}H NMR reaction monitoring for the reaction of **1a** with catalyst **7b** at –20 °C in $CDCl_3$.*

9.2. Kinetic Isotope Effects Determination from an Intermolecular Competition

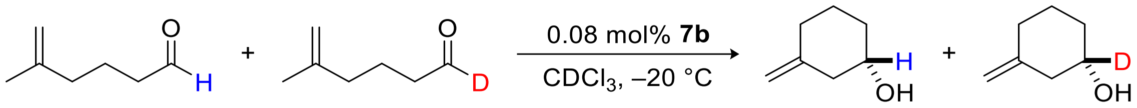

Sample preparation and data acquisition

In an oven-dried (80 °C, overnight) 5 mm NMR tube under Ar, 0.5 mL IDPi **7b** solution (0.208 mg/mL in anhydrous $CDCl_3$, 0.08 mol%) was added. The NMR tube was cooled to –20 °C, then the distilled 5-methylhex-5-enal **1a** (0.0316 mmol, 4.5 µL) and 5-methylhex-5-enal-1-*d* (0.0316 mmol, 4.5 µL) was added at the same time. The NMR tube was turned upside down and vortexed for 10 s to ensure mixing of all the components. Afterwards, the sample was quickly transferred to the precooled Bruker 600 MHz NMR magnet equipped with a cryogenically cooled BBO probe. After quick shimming, single scan ^{1}H NMR and ^{2}H NMR spectra were acquired every 5 min until a sufficient conversion was observed. The acquired NMR data was imported with the *Reaction Monitoring* plugin into MNOVA 16.0.0 and processed therein (phasing, baseline correction, integration, fitting). For the generation of the concentration profiles, the initial spectrum of the individual run was used as absolute concentration reference. Experiments were conducted in duplicates.

Run 1

The following **Figure S3** and **Figure S4** show ^{1}H NMR and ^{2}H NMR spectra taken at different time points during the reaction at –20 °C.

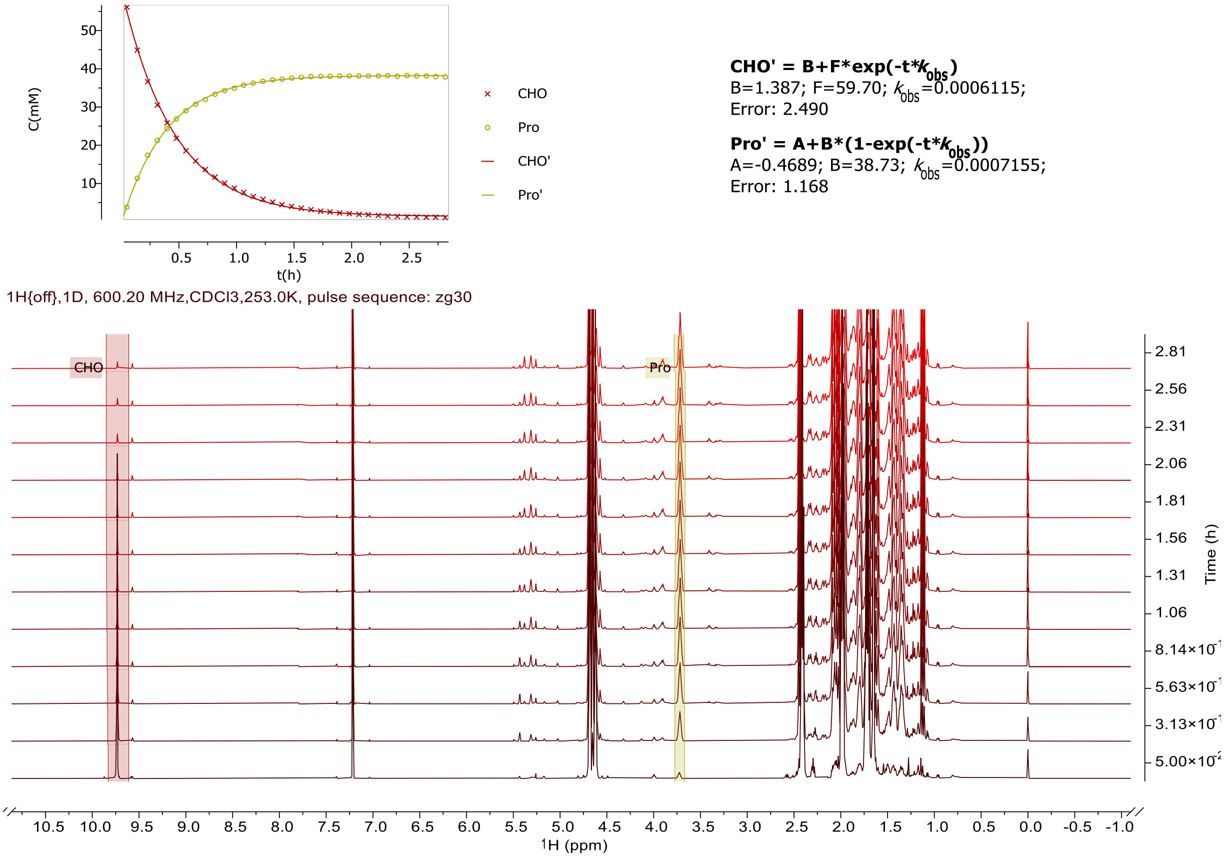

Figure S3. *Top left: ^{1}H NMR concentration profile for the intermolecular competition KIE measurement; top right: Fitting results for SM consumption and product formation; bottom: ^{1}H NMR spectra taken at different time points of the reaction*

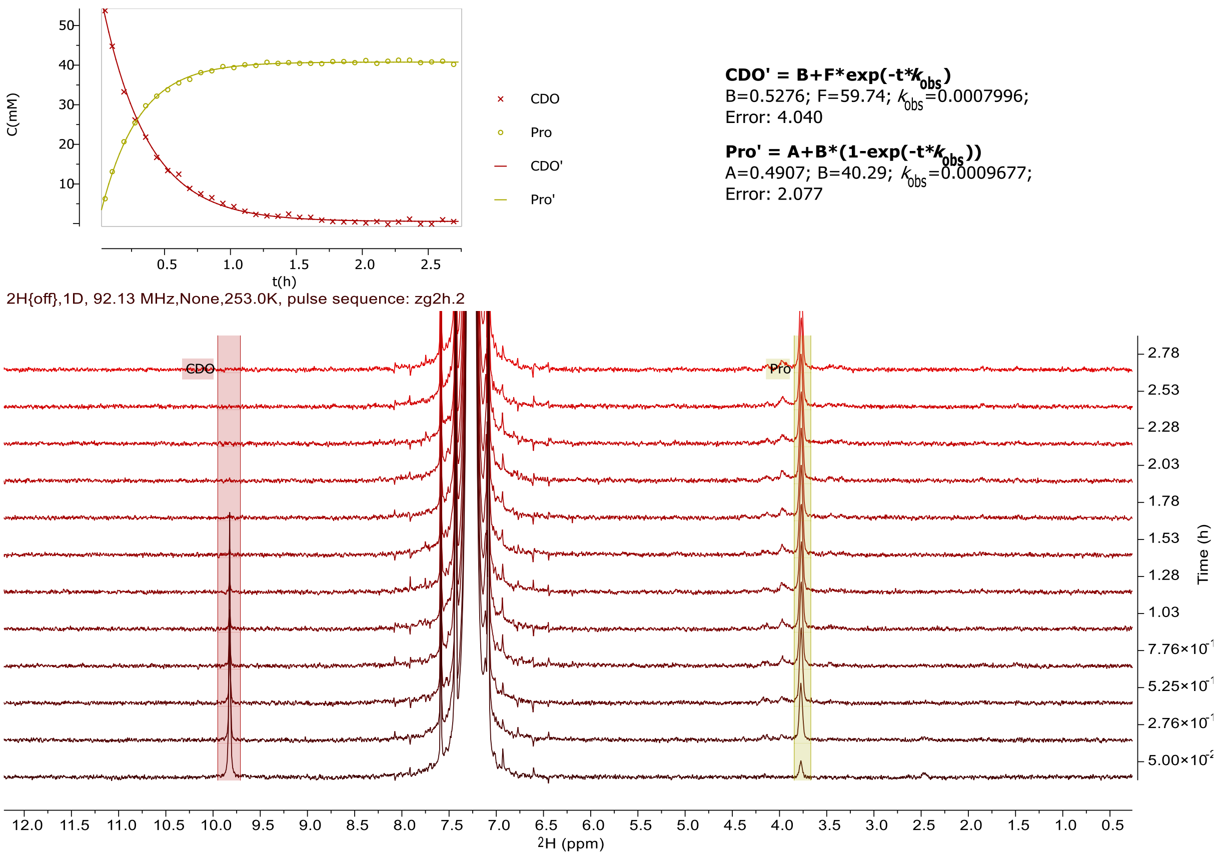

.

Figure S4. *Top left: ^{2}H NMR concentration profile for the intermolecular competition KIE measurement; top right: Fitting results for SM consumption and product formation; bottom: ^{2}H NMR spectra taken at different time points of the reaction*

Run 2

The following **Figure S5** and **Figure S6** show ^{1}H NMR and ^{2}H NMR spectra taken at different time points during the reaction at –20 °C.

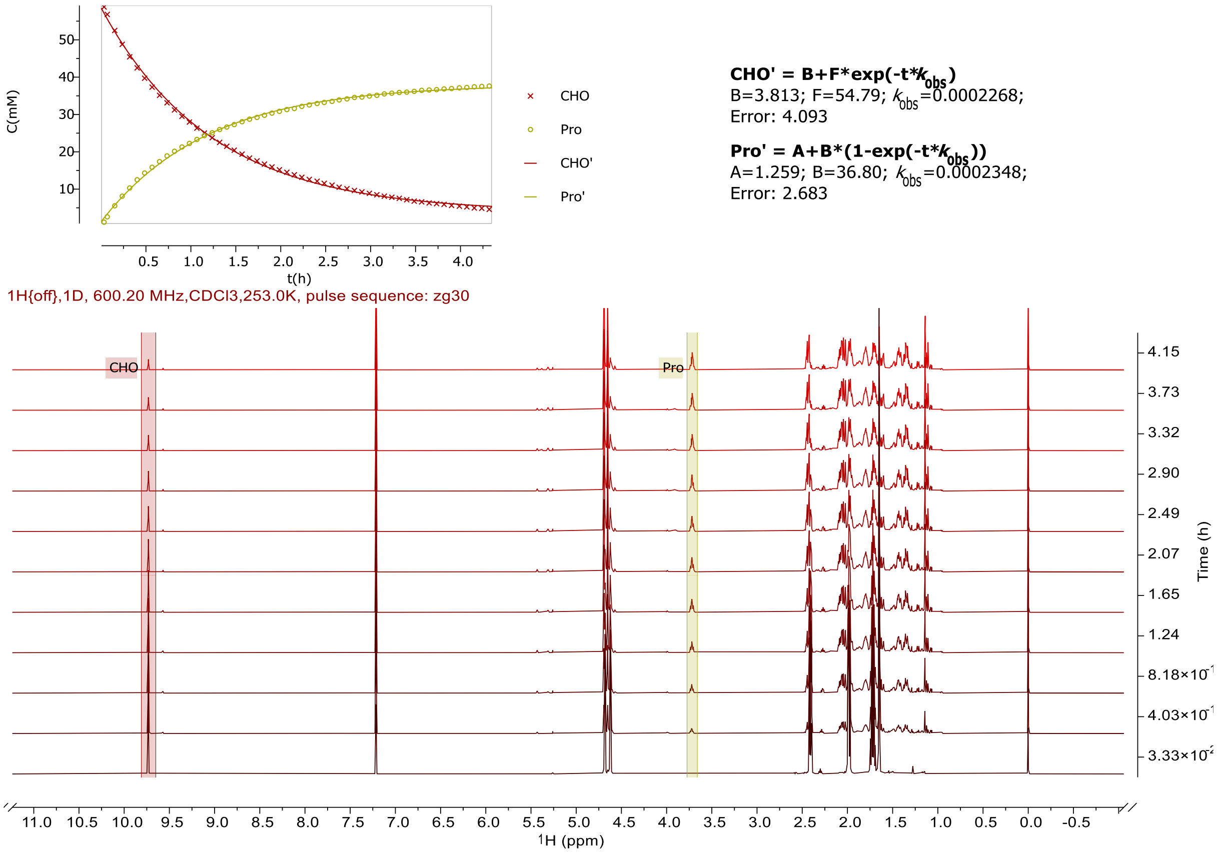

Figure S5. *Top left: ^{1}H NMR concentration profile for the intermolecular competition KIE measurement; top right: Fitting results for SM consumption and product formation; bottom: ^{1}H NMR spectra taken at different time points of the reaction.*

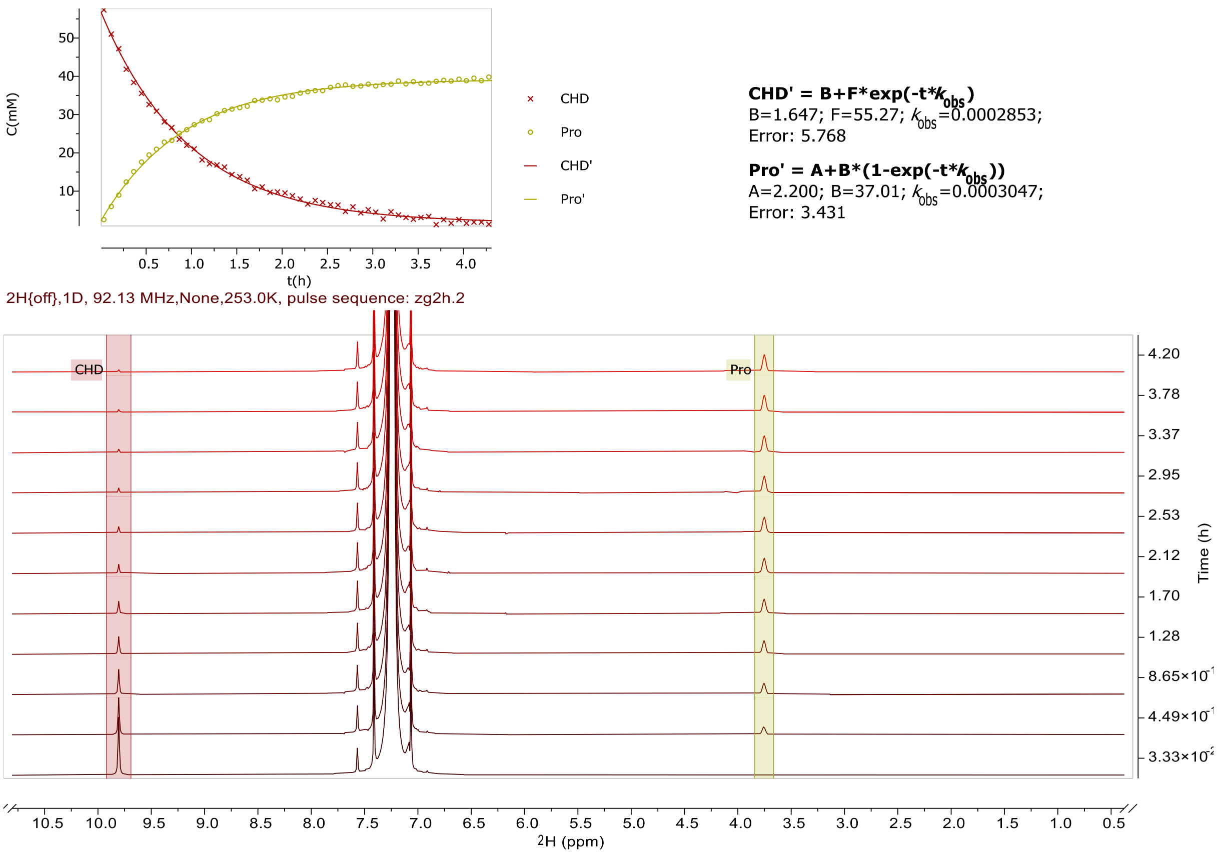

Figure S6. *Top left: ^{2}H NMR concentration profile for the intermolecular competition KIE measurement; top right: Fitting results for SM consumption and product formation; bottom: ^{2}H NMR spectra taken at different time points of the reaction.*

KIE Determination On Basis of ^{1}H/^{2}H NMR Concentration Profiles

Table S4. Determination of KIE

		Run 1		Run 2		Average
		SM	Pro	SM	Pro	
k_{obs} (s^{-1})	k_H	0.0006115	0.0007155	0.0002268	0.000235	
	k_D	0.0007996	0.0009677	0.0002853	0.0003047	
**KIE**		**0.76**	**0.74**	**0.79**	**0.77**	**0.77 ± 0.02**

10. Computational Study

10.1. Method

A conformational search has been performed on possible catalyst substrate orientations at GFN2-xTB level of theory[15] implemented in ORCA 4.2.1,[16] using SC-AFIR with constraints by GRRM program.[17,18] Molecular geometries were optimized at r^2SCAN-3c[19] implemented in ORCA 6.0.1 program.[20] Thermal free energy corrections have been performed at the same level of theory using ORCA 6.0.1 program, and the temperature was set at 253.15 K. Solvation effect has been accounted by using SMD ($CHCl_3$) solvation model as implemented in ORCA 6.0.1 program.[21] All single point energy is calculated at SMD ($CHCl_3$)-ωB97M-V/def2-TZVPP level of theory.[22,23] RI approximation was used with RIJCOSX with def2/J as an auxiliary basis set implemented in ORCA 6.0.1 program.[24] The visualizations of the molecular geometries were generated using the ChimeraX[25] version 1.9 followed by rendering with Blender version 4.0.[26]

10.2. Results and Discussion

We computed the stable conformers of ion pair **II**, identifying two major binding modes. In both cases, the hydroxy group points toward the equatorial position of the chair conformer. In one binding mode, the *exo*-methyl group is located close to the catalytically active site (ion pair **IIa**), while in the other, it points outward (ion pair **IIb**). According to Hammond's postulate, we speculate that the deprotonation step would proceed through transition states resembling these ion pairs (**Figure S7**). Although these structures are not transition states, the free energies of the ion pairs, calculated at SMD ($CHCl_3$)-ωB97M-V/def2-TZVPP//r^2SCAN-3c level of theory, qualitatively reproduce the experimental trends. While a definitive mechanistic conclusion requires explicit transition-state calculations, we expect the ion pairs to serve as useful models that capture key steric and electronic factors governing selectivity.

In ion pair **IIa**, the Lewis basic oxygens are within reach of the *exo*-methyl group but not the *endo*-methylene group, so deprotonation would preferentially occur in an *exo*-selective manner via similar transition states. This conformer already explains the high *E*-selectivity, as it minimizes steric interactions with the pyrene group, whereas a *Z*-substituent would be oriented toward the catalytic active site. In contrast, in ion pair **IIb**, the Lewis basic oxygen is located near the methylene group, presumably favoring formation of the *endo*-product. However, since this ion pair is already less stable, and deprotonation would further "push" the methyl group towards the pyrene group, the corresponding transition states are expected to be even more destabilized.

These preliminary studies already offer reasonable mechanistic insights, consistent with previous computational studies of *i*IDP-catalyzed reactions, and account for the observed selectivities to some extent. Further mechanistic analysis is ongoing.

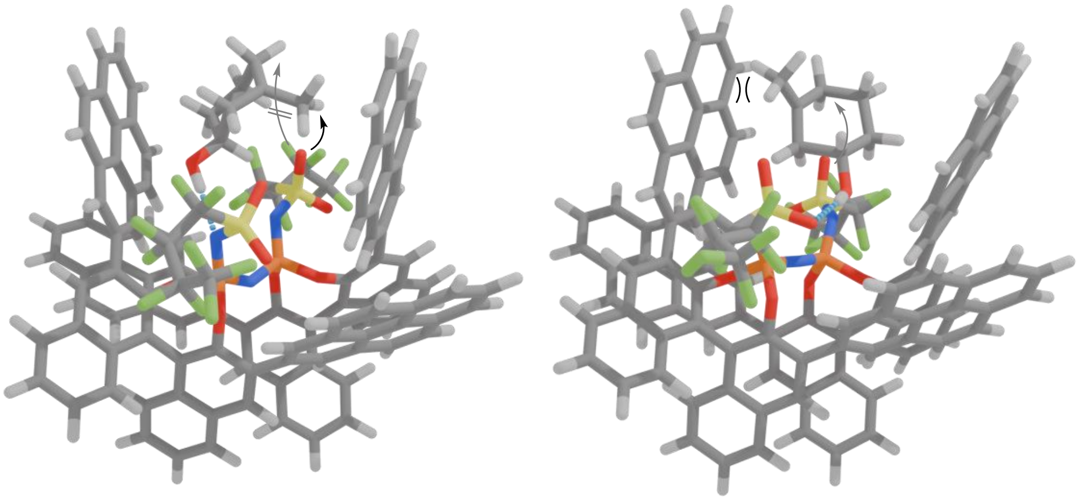

ion pair **IIa**
(leading to *exo*-**2a**)

0.0 kcal/mol

ion pair **IIb**
(leading to *endo*-**2a**)

+1.2 kcal/mol

Figure S7. *Visualization of the ion pair intermediates **IIa** and **IIb**.*

10.3. Cartesian Coordinates of the Optimized Structures

IIa

O	2.266628	2.844983	-1.850294
O	0.403190	2.344397	-0.220414
O	2.343778	-1.639355	0.724725
O	3.539119	-1.364554	-1.488780
S	-0.365889	-2.510019	-0.796037
S	-0.366677	2.370143	-3.679938
O	-0.499456	-2.247951	0.640428
O	-1.498166	-2.192615	-1.682519
O	-1.511605	1.735016	-4.354736
O	-0.537037	3.709240	-3.093301
N	1.013957	-2.044111	-1.480892
N	0.346864	1.287843	-2.737902
P	2.111107	-1.091245	-0.780788
N	1.956774	0.464161	-0.761289
P	1.218770	1.667361	-1.443939
H	0.688357	-2.161090	-3.385823
C	0.098060	3.695413	-0.169820
C	3.075019	3.307848	-0.809341
C	3.413043	-1.053113	1.401233
C	4.279245	-2.496779	-1.162714
C	-1.271281	4.054520	-0.251887
C	4.446375	-3.480255	-2.170311
C	4.423366	2.870216	-0.791015
C	3.128416	-0.046819	2.358106
C	1.423977	8.343542	-0.012202
C	1.780791	7.016221	-0.010958
C	0.796450	5.998735	-0.009050
C	-0.579063	6.383942	-0.057792
C	-0.911948	7.759849	-0.038676
C	0.066392	8.722260	-0.010482
H	2.195999	9.107336	-0.022651
H	2.829433	6.738337	-0.021593
C	1.121653	4.606167	-0.000882
C	-1.578125	5.392563	-0.160767
H	-1.961934	8.040581	-0.065477
H	-2.620509	5.699933	-0.193787
C	4.945409	5.036280	3.526371
C	5.458275	4.304137	2.484925
C	4.665799	3.990898	1.354453
C	3.323520	4.479875	1.281164
C	2.826993	5.230503	2.375129

C	3.613519	5.494154	3.470929
H	6.228403	2.896125	0.342108
H	6.483370	3.943842	2.509813
C	5.189688	3.215365	0.298075
C	2.521463	4.138391	0.143979
H	1.811320	5.607558	2.341292
H	3.204743	6.064951	4.299483
C	8.174501	-0.388714	1.908022
C	7.139550	-1.097032	1.345674
C	5.792911	-0.806588	1.673168
C	5.534026	0.253372	2.599618
C	6.624665	0.946026	3.178435
C	7.918549	0.638301	2.839133
H	9.198401	-0.625892	1.634721
H	7.353140	-1.897865	0.646371
C	4.682425	-1.482132	1.076408
C	4.204214	0.592115	2.932699
H	6.410166	1.734391	3.895209
H	4.029396	1.373529	3.668616
C	6.892078	-6.184446	1.047611
C	6.423607	-5.976385	-0.225158
C	5.722067	-4.791630	-0.556571
C	5.536034	-3.787768	0.444220
C	6.005819	-4.047543	1.754729
C	6.666064	-5.216286	2.047502
H	5.362487	-5.353760	-2.606844
H	6.569342	-6.727342	-0.997559
H	5.827997	-3.315512	2.535281
H	7.009875	-5.399510	3.061219
C	5.187181	-4.596272	-1.846775
C	4.845079	-2.586185	0.097066
H	-0.201688	9.774588	-0.005368
H	5.560956	5.265109	4.391446
H	8.746209	1.180951	3.286450
H	7.420774	-7.100821	1.292425
C	0.843036	-5.234249	-0.280281
C	-0.263739	-4.409538	-1.008682

C	0.905309	-5.302544	1.286925
C	0.928295	2.619707	-5.119638
C	1.343485	4.093918	-5.411111
C	0.219572	4.975015	-6.071783
F	2.053981	1.943697	-4.816072
F	0.418550	2.103724	-6.266184
F	2.389705	4.056217	-6.282668
F	1.792729	4.664233	-4.274223
F	0.342153	4.892316	-7.417399
F	-1.005332	4.519219	-5.736128
F	0.707201	-6.513918	-0.722338
F	2.050912	-4.777265	-0.681281
F	-0.088862	-4.638106	-2.333149
F	-1.468885	-4.909158	-0.649503
F	1.849722	-6.235616	1.568494
F	1.321853	-4.118368	1.771827
C	0.292603	6.484521	-5.686070
C	-0.381001	-5.717141	2.067095
F	-0.574034	7.170734	-6.448085
F	1.529221	6.955991	-5.918016
F	-0.022739	6.676206	-4.405746
F	-0.910134	-6.836148	1.554230
F	-0.044633	-5.950348	3.345780
F	-1.297232	-4.745354	2.046899
C	3.863150	-3.333014	-3.521513
C	3.232328	-4.427106	-4.121904
C	3.975489	-2.134404	-4.230130
C	2.719087	-4.347840	-5.420021
H	3.104640	-5.347222	-3.556328
C	3.483130	-2.016405	-5.531169
H	4.459027	-1.277595	-3.775513
C	2.854007	-3.132594	-6.145663
C	2.030116	-5.439067	-6.036748
C	3.573560	-0.788522	-6.257258
C	2.313671	-3.012913	-7.449233
C	1.505274	-5.325993	-7.286635
H	1.925086	-6.366394	-5.478808

C	3.073782	-0.676501	-7.516267
H	4.043363	0.062760	-5.775134
C	1.621222	-4.111449	-8.035633
C	2.421960	-1.778902	-8.155324
H	0.980836	-6.163941	-7.739722
H	3.150364	0.266882	-8.052247
C	1.065114	-3.959829	-9.313338
C	1.851621	-1.676648	-9.430885
C	1.183035	-2.755819	-9.998898
H	0.539416	-4.797109	-9.765829
H	1.933158	-0.737787	-9.973088
H	0.748287	-2.657335	-10.989642
C	-2.324408	3.048103	-0.504287
C	-3.290312	3.320905	-1.478473
C	-2.367219	1.830648	0.179613
C	-4.295508	2.400575	-1.783680
H	-3.221482	4.241076	-2.052082
C	-3.352543	0.879343	-0.099506
H	-1.627072	1.604046	0.938822
C	-4.334483	1.159947	-1.089236
C	-5.272725	2.647308	-2.799715
C	-3.383983	-0.387461	0.562321
C	-5.323529	0.195317	-1.404276
C	-6.227577	1.725866	-3.099445
H	-5.233199	3.592958	-3.334611
C	-4.328158	-1.315881	0.258347
H	-2.614909	-0.611699	1.294902
C	-6.285065	0.466512	-2.420760
C	-5.325047	-1.064400	-0.736497
H	-6.963601	1.929582	-3.873789
H	-4.320933	-2.282946	0.755124
C	-7.229953	-0.517815	-2.742539
C	-6.287314	-2.018199	-1.096456
C	-7.225139	-1.744558	-2.085840
H	-7.968533	-0.312182	-3.513658
H	-6.290150	-2.981504	-0.592749
H	-7.964106	-2.496603	-2.348152

C	5.011028	2.123660	-1.924341
C	5.756812	0.967328	-1.695228
C	4.887214	2.621160	-3.224433
C	6.370940	0.283132	-2.749820
H	5.833082	0.570707	-0.685886
C	5.508467	1.986321	-4.302830
H	4.314452	3.527471	-3.401859
C	6.252307	0.794099	-4.073302
C	7.088945	-0.938479	-2.551131
C	5.424312	2.495578	-5.638746
C	6.846243	0.104200	-5.160619
C	7.637668	-1.613786	-3.595841
H	7.169597	-1.328266	-1.541102
C	6.014182	1.846471	-6.677808
H	4.870658	3.414813	-5.805777
C	7.535711	-1.121514	-4.935027
C	6.730973	0.622959	-6.482128
H	8.162582	-2.550766	-3.427057
H	5.938674	2.244948	-7.686728
C	8.085044	-1.801766	-6.029280
C	7.301516	-0.089852	-7.543351
C	7.966474	-1.288936	-7.315277
H	8.605258	-2.741264	-5.861475
H	7.210369	0.300975	-8.553520
H	8.396010	-1.832211	-8.151909
C	1.737391	0.281342	2.735026
C	1.324303	1.612359	2.811203
C	0.836820	-0.740100	3.046179
C	0.010859	1.940491	3.162843
H	2.019005	2.406830	2.550183
C	-0.477914	-0.456264	3.419111
H	1.153296	-1.776511	2.993691
C	-0.910504	0.898132	3.465011
C	-0.458317	3.291763	3.183516
C	-1.417502	-1.489315	3.732538
C	-2.258722	1.205051	3.779508
C	-1.754848	3.586709	3.466801

H	0.245705	4.082845	2.944204
C	-2.706155	-1.194984	4.049394
H	-1.080377	-2.521253	3.689620
C	-2.701800	2.558722	3.771356
C	-3.177644	0.156827	4.071954
H	-2.095991	4.618948	3.454143
H	-3.411871	-1.991412	4.273103
C	-4.046191	2.834233	4.051004
C	-4.511197	0.481813	4.348121
C	-4.936111	1.805231	4.334547
H	-4.388535	3.865802	4.038352
H	-5.216767	-0.316362	4.563917
H	-5.976408	2.037022	4.543828
O	0.505262	-2.310887	-4.340821
C	-0.546552	-1.557434	-4.758543
H	-0.769750	-0.714864	-4.094057
C	-0.431284	-1.167897	-6.218139
H	-0.194587	-2.062611	-6.807967
H	0.431536	-0.496100	-6.306506
C	-1.675771	-0.466602	-6.734554
H	-1.598476	-0.300061	-7.813371
H	-1.791516	0.503312	-6.245006
C	-2.983393	-1.314363	-6.495706
H	-3.858211	-0.706386	-6.740877
H	-2.943808	-2.202130	-7.135894
C	-2.923912	-1.679322	-5.072599
C	-1.932405	-2.604613	-4.690292
H	-1.665999	-3.368754	-5.420671
H	-1.951637	-2.938727	-3.656067
C	-3.643527	-0.885358	-4.070312
H	-3.244730	-1.017398	-3.062430
H	-3.678604	0.178107	-4.339297
H	-4.694761	-1.226110	-4.065612

IIb

O	2.475108	2.668637	-2.005406
O	0.423174	2.243150	-0.555055
O	2.365441	-1.554017	0.423964
O	3.771804	-1.078566	-1.608881
S	0.437392	-3.405366	-1.361921
S	0.665588	2.409028	-4.580448
O	-0.454130	-2.790520	-0.341238
O	-0.209470	-4.091308	-2.492627
O	2.092302	2.441805	-4.944661
O	-0.291337	1.875494	-5.568105
N	1.588653	-2.454515	-1.889279
N	0.316333	1.800053	-3.154302
P	2.227242	-1.175228	-1.148857
N	1.602223	0.222788	-1.367264
P	1.217551	1.655526	-1.838007
H	-1.175075	-1.357924	-1.439314
C	0.205970	3.610815	-0.454984
C	3.229196	3.043885	-0.900962
C	3.314481	-0.893537	1.194347
C	4.587178	-2.156665	-1.274259
C	-1.119334	4.095304	-0.616456
C	4.873693	-3.112651	-2.282502
C	4.590884	2.637224	-0.857223
C	2.836918	0.031023	2.158594
C	1.924100	8.119632	-0.080422
C	2.163275	6.766604	-0.087222
C	1.094564	5.840415	-0.164028
C	-0.233488	6.349989	-0.288921
C	-0.447907	7.749760	-0.251573
C	0.607288	8.619473	-0.143689
H	2.760112	8.811234	-0.034091
H	3.183047	6.400404	-0.049259
C	1.288427	4.422691	-0.171878
C	-1.304823	5.456454	-0.483603
H	-1.466284	8.121148	-0.333275
H	-2.311113	5.861921	-0.544786
C	4.957591	4.814518	3.479690
C	5.522499	4.122615	2.437750
C	4.756659	3.762949	1.302608

C	3.392996	4.178860	1.222129
C	2.836216	4.876584	2.321528
C	3.596010	5.176949	3.426398
H	6.376850	2.753986	0.304853
H	6.565342	3.820496	2.472875
C	5.323621	3.018364	0.247840
C	2.640802	3.855940	0.049755
H	1.795600	5.179974	2.282686
H	3.145295	5.705585	4.261166
C	7.905589	0.057798	2.399580
C	7.012176	-0.637392	1.620680
C	5.614572	-0.487966	1.797383
C	5.155826	0.444790	2.779086
C	6.101844	1.119456	3.587989
C	7.449008	0.931065	3.407625
H	8.971973	-0.073217	2.240729
H	7.378051	-1.318947	0.861625
C	4.652600	-1.200798	1.013988
C	3.773536	0.679776	2.933367
H	5.735519	1.806385	4.345805
H	3.442125	1.381451	3.695437
C	7.444926	-5.608562	1.001894
C	7.054325	-5.392962	-0.295422
C	6.229734	-4.291837	-0.633961
C	5.845230	-3.367241	0.387822
C	6.230800	-3.643756	1.721988
C	7.009965	-4.735597	2.019984
H	6.017619	-4.862394	-2.701255
H	7.352759	-6.079347	-1.083884
H	5.896883	-2.986084	2.516916
H	7.286864	-4.931693	3.051636
C	5.734420	-4.134682	-1.944908
C	5.050693	-2.234413	0.025093
H	0.434129	9.691373	-0.128494
H	5.553923	5.079063	4.348220
H	8.164427	1.458060	4.032411
H	8.068476	-6.461985	1.251002

C	0.499706	-5.547572	0.607662
C	1.380561	-4.827350	-0.454347
C	1.074459	-6.929273	1.072137
C	0.133668	4.246105	-4.404184
C	0.603653	5.190977	-5.565064
C	1.961216	5.929443	-5.303353
F	-1.214862	4.260894	-4.361589
F	0.597554	4.739231	-3.233426
F	-0.340092	6.148995	-5.740790
F	0.692687	4.481077	-6.714028
F	1.736208	6.950706	-4.446068
F	2.867346	5.092630	-4.757400
F	0.393319	-4.745889	1.695491
F	-0.729242	-5.760664	0.089998
F	2.478048	-4.328891	0.140064
F	1.766056	-5.716195	-1.395348
F	0.838138	-7.848621	0.111571
F	2.410228	-6.825072	1.262286
C	2.612199	6.530588	-6.591496
C	0.455053	-7.468954	2.402197
F	3.095974	5.565433	-7.381376
F	3.626840	7.329840	-6.230409
F	1.720519	7.255577	-7.282294
F	0.843357	-8.740676	2.576545
F	0.867182	-6.753416	3.453830
F	-0.884073	-7.432485	2.345351
C	4.168376	-3.093950	-3.582556
C	3.668023	-4.302382	-4.078213
C	3.879317	-1.908336	-4.264613
C	2.851492	-4.346137	-5.210804
H	3.855604	-5.223915	-3.533413
C	3.027811	-1.901738	-5.371001
H	4.279522	-0.964617	-3.915481
C	2.494907	-3.128372	-5.851229
C	2.313549	-5.569975	-5.719651
C	2.616367	-0.680307	-5.991628
C	1.599130	-3.133385	-6.947733

C	1.478511	-5.581843	-6.793139
H	2.583580	-6.499243	-5.224401
C	1.724711	-0.677436	-7.018398
H	3.003489	0.257450	-5.605600
C	1.083963	-4.367420	-7.439546
C	1.186594	-1.899196	-7.533566
H	1.080152	-6.520874	-7.169225
H	1.395705	0.264890	-7.448892
C	0.181776	-4.345139	-8.514247
C	0.269174	-1.928937	-8.596163
C	-0.218362	-3.136813	-9.080486
H	-0.208289	-5.284340	-8.898743
H	-0.048464	-0.990566	-9.044163
H	-0.918913	-3.140154	-9.910920
C	-2.278866	3.229607	-0.923051
C	-3.271078	3.721106	-1.781391
C	-2.445325	1.965665	-0.351153
C	-4.433585	2.996456	-2.050297
H	-3.124842	4.675841	-2.279148
C	-3.588461	1.202952	-0.603106
H	-1.683992	1.556947	0.302136
C	-4.610087	1.722291	-1.443551
C	-5.452068	3.483120	-2.930849
C	-3.747075	-0.112697	-0.065150
C	-5.762110	0.947668	-1.732003
C	-6.568592	2.751732	-3.190439
H	-5.313982	4.457847	-3.392471
C	-4.853872	-0.854959	-0.332268
H	-2.953253	-0.516538	0.556403
C	-6.764558	1.459173	-2.605608
C	-5.903194	-0.353395	-1.167246
H	-7.334828	3.137616	-3.858412
H	-4.954729	-1.853632	0.086369
C	-7.895846	0.677120	-2.871955
C	-7.051570	-1.099025	-1.465724
C	-8.034615	-0.584061	-2.303110
H	-8.666431	1.066431	-3.532617
H	-7.165524	-2.090632	-1.034861
H	-8.919403	-1.176061	-2.519405
C	5.230521	1.888714	-1.961747
C	6.159747	0.887084	-1.666056
C	4.960135	2.196371	-3.299076
C	6.792403	0.157875	-2.676296
H	6.360955	0.631011	-0.629439
C	5.581142	1.500558	-4.340269
H	4.252039	2.976628	-3.552000
C	6.496952	0.455215	-4.035848
C	7.698638	-0.913350	-2.393321
C	5.299163	1.789812	-5.714205
C	7.073767	-0.313538	-5.077194
C	8.251690	-1.652798	-3.390346
H	7.923124	-1.140769	-1.354822
C	5.858981	1.057370	-6.713385
H	4.598646	2.589944	-5.937836
C	7.956305	-1.386857	-4.765234
C	6.754007	-0.024325	-6.434552
H	8.925689	-2.472995	-3.154930
H	5.620594	1.275418	-7.751542
C	8.498037	-2.145055	-5.810843
C	7.319650	-0.811252	-7.445800
C	8.179809	-1.857447	-7.133075
H	9.172607	-2.964831	-5.576764
H	7.075395	-0.596091	-8.482887
H	8.607516	-2.457492	-7.931150
C	1.389123	0.255178	2.367718
C	0.899661	1.555812	2.506170
C	0.511096	-0.826395	2.484146
C	-0.455867	1.798852	2.748642
H	1.574965	2.399381	2.387622
C	-0.845512	-0.629813	2.752279
H	0.880141	-1.841571	2.380192
C	-1.345335	0.696067	2.886199
C	-0.992435	3.123192	2.830281
C	-1.755010	-1.726175	2.899144

C	-2.724596	0.915995	3.129390
C	-2.319719	3.335422	3.035980
H	-0.316165	3.964491	2.701407
C	-3.071708	-1.515195	3.164983
H	-1.368228	-2.734958	2.782894
C	-3.232218	2.244992	3.194885
C	-3.606589	-0.192103	3.279202
H	-2.712565	4.348347	3.076281
H	-3.750865	-2.357396	3.274396
C	-4.604179	2.436925	3.400589
C	-4.968742	0.050108	3.492535
C	-5.457669	1.349948	3.547754
H	-4.997116	3.449543	3.441240
H	-5.646234	-0.793169	3.600134
H	-6.519459	1.518042	3.702529
O	-1.319126	-0.627435	-2.071649
C	-1.894236	-1.078078	-3.230911
H	-2.425440	-2.031266	-3.089852
C	-2.764662	-0.010994	-3.862647
H	-2.184842	0.916439	-3.936159
H	-3.600939	0.174122	-3.180025
C	-3.302819	-0.434044	-5.216792
H	-3.847304	0.391024	-5.685509
H	-4.002622	-1.272800	-5.110829
C	-2.151723	-0.847698	-6.210599
H	-2.583510	-1.244165	-7.133530
H	-1.542252	0.039933	-6.405177
C	-1.393074	-1.853701	-5.460723
C	-0.650066	-1.398195	-4.341049
H	-0.185138	-0.419001	-4.459822
H	-0.012008	-2.144181	-3.870967
C	-1.673435	-3.280189	-5.654664
H	-1.291777	-3.901429	-4.841197
H	-2.744016	-3.449403	-5.830525
H	-1.179455	-3.589131	-6.593412

11. NMR Spectra

(*S*)-3,3'-di(pyren-2-yl)-[1,1'-binaphthalene]-2,2'-diol

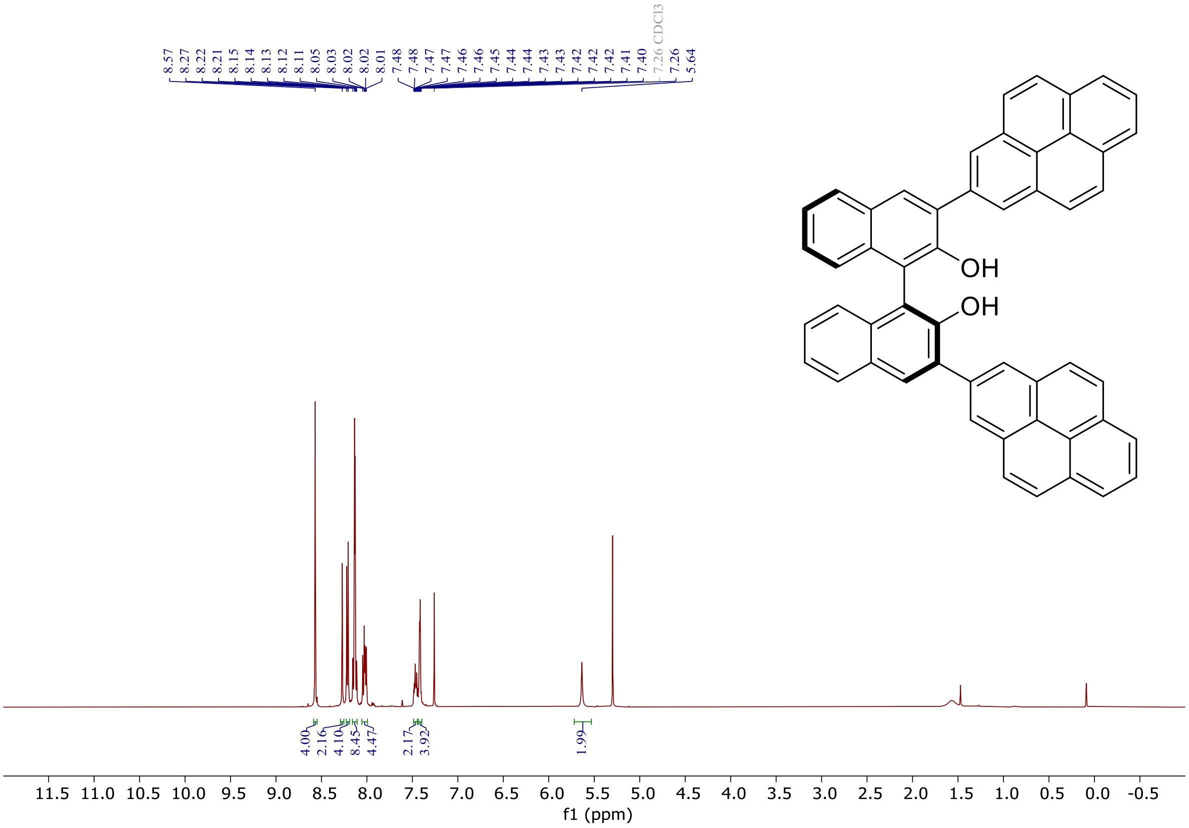

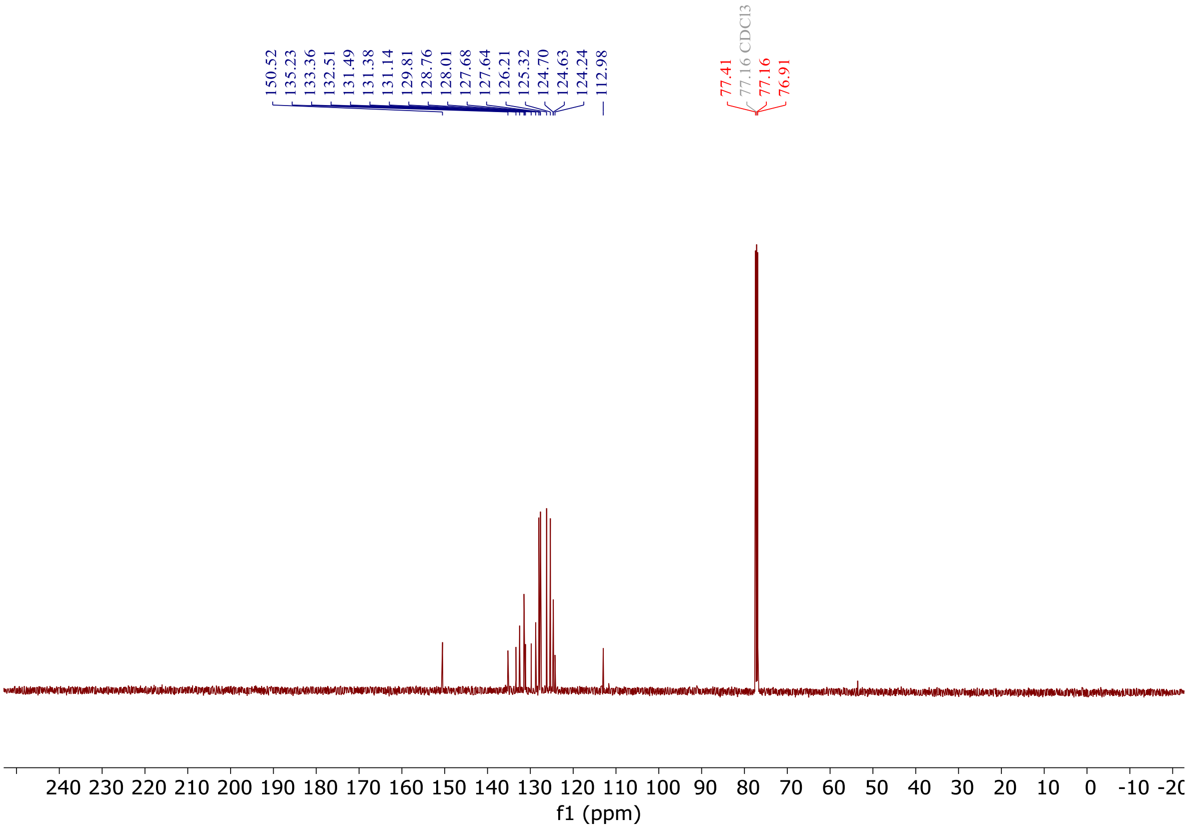

IDPi 7a

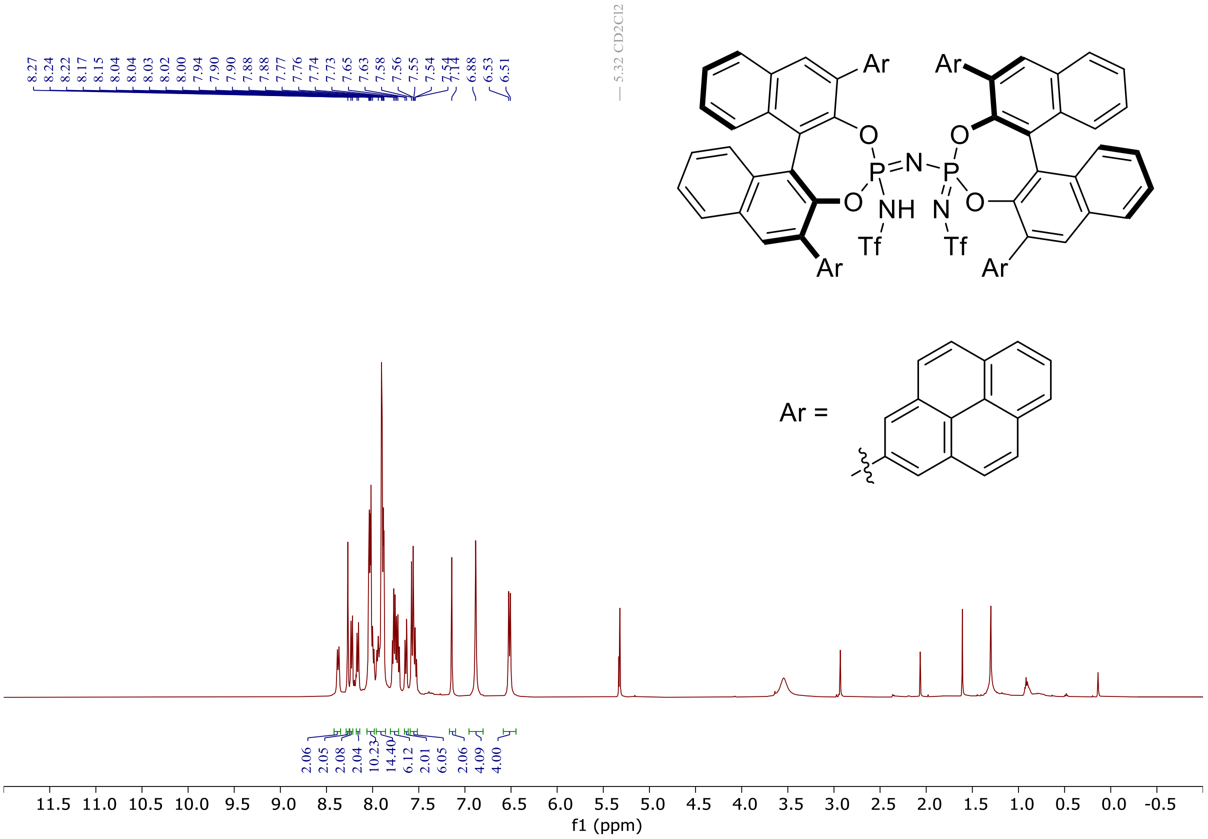
8.27 8.24 8.22 8.17 8.15 8.04 8.04 8.03 8.02 8.00 7.94 7.90 7.90 7.88 7.88 7.77 7.76 7.74 7.73 7.65 7.63 7.58 7.56 7.55 7.54 7.54 7.14 6.88 6.53 6.51
5.32 CD2Cl2
Ar
O
P
N
NH
Tf
Ar =
2.06 2.05 2.08 2.04 10.23 14.40 6.12 2.01 6.05 2.06 4.09 4.00
11.5 11.0 10.5 10.0 9.5 9.0 8.5 8.0 7.5 7.0 6.5 6.0 5.5 5.0 4.5 4.0 3.5 3.0 2.5 2.0 1.5 1.0 0.5 0.0 -0.5
f1 (ppm)

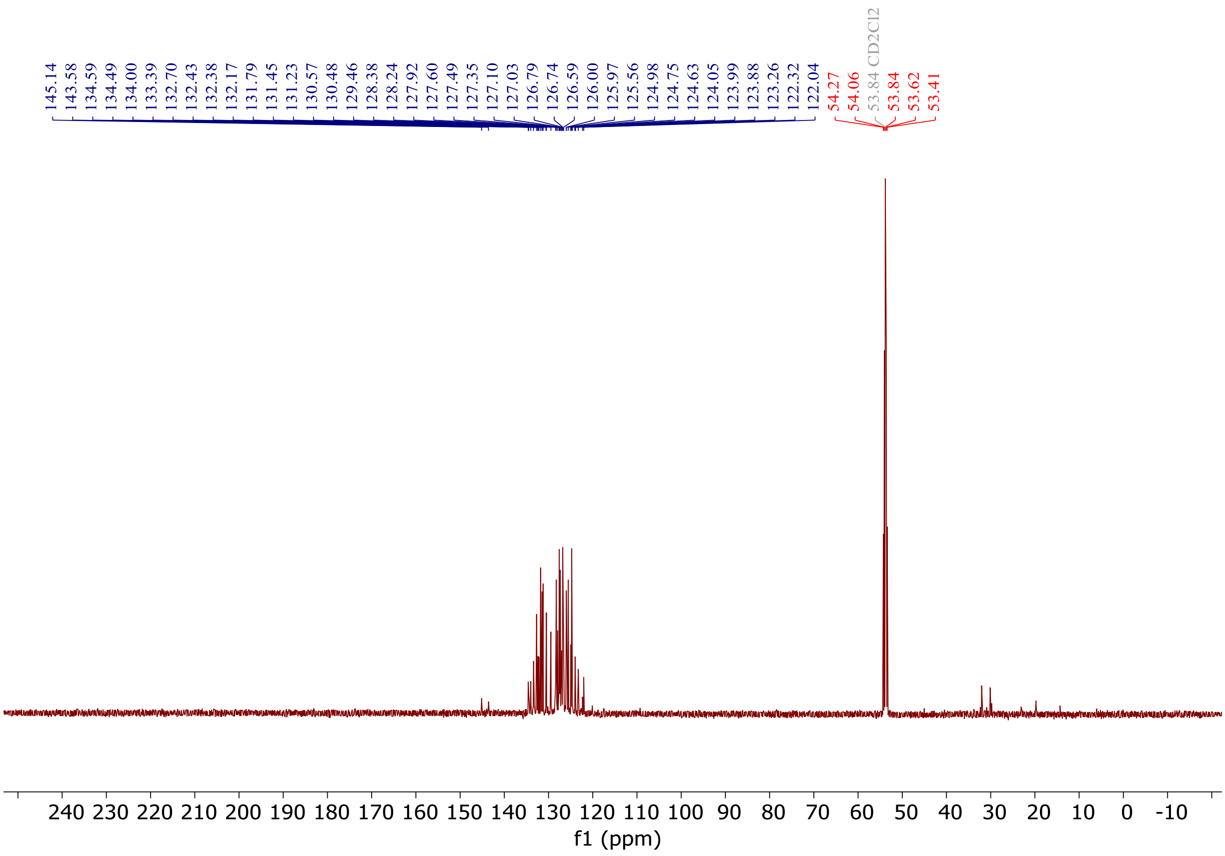
145.14 143.58 134.59 134.49 134.00 133.39 132.70 132.43 132.38 132.17 131.79 131.45 131.23 130.57 130.48 129.46 128.38 128.24 127.92 127.60 127.49 127.35 127.10 127.03 126.79 126.74 126.59 126.00 125.97 125.56 124.98 124.75 124.63 124.05 123.99 123.88 123.26 122.32 122.04
54.27 54.06 53.84 CD2Cl2 53.84 53.62 53.41
240 230 220 210 200 190 180 170 160 150 140 130 120 110 100 90 80 70 60 50 40 30 20 10 0 -10
f1 (ppm)

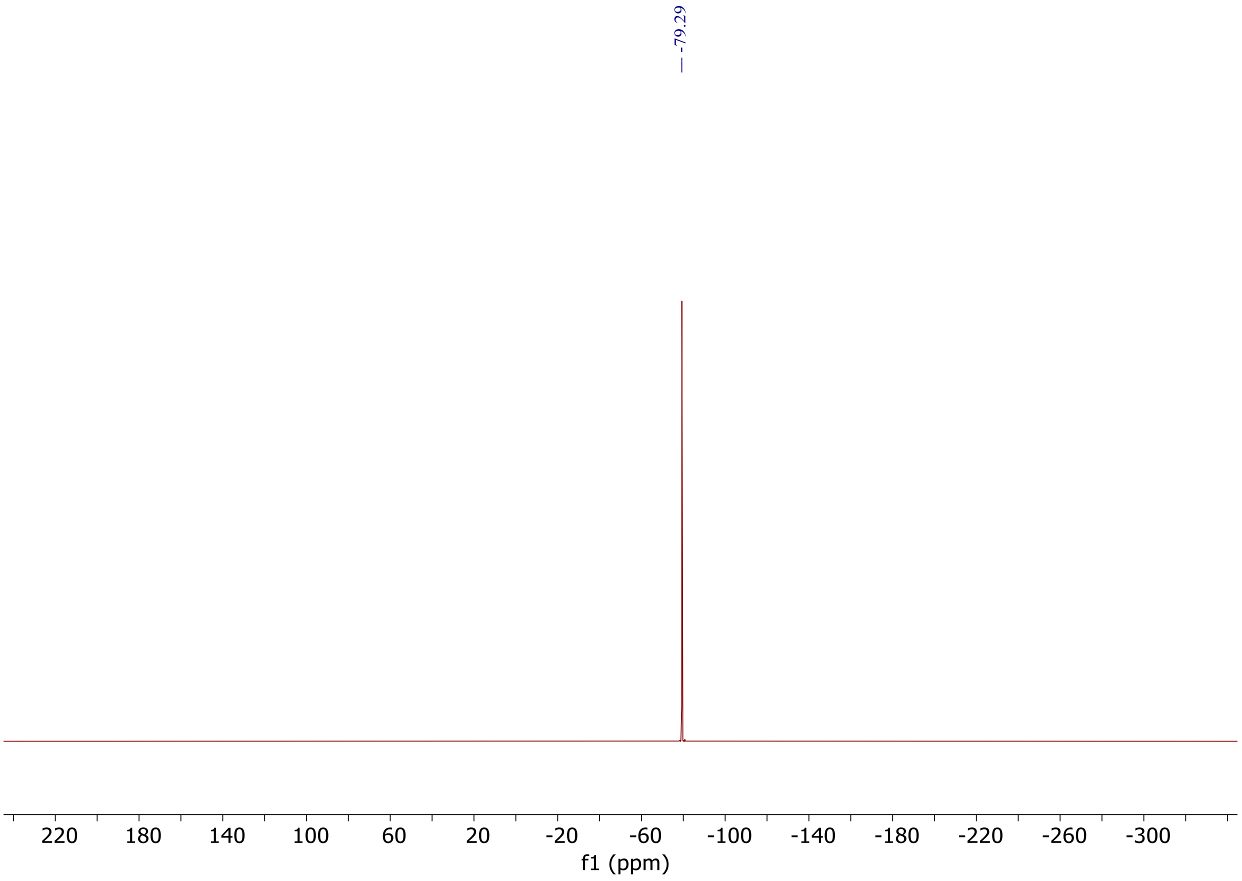
-79.29
220
180
140
100
60
20
-20
-60
-100
-140
-180
-220
-260
-300
f1 (ppm)

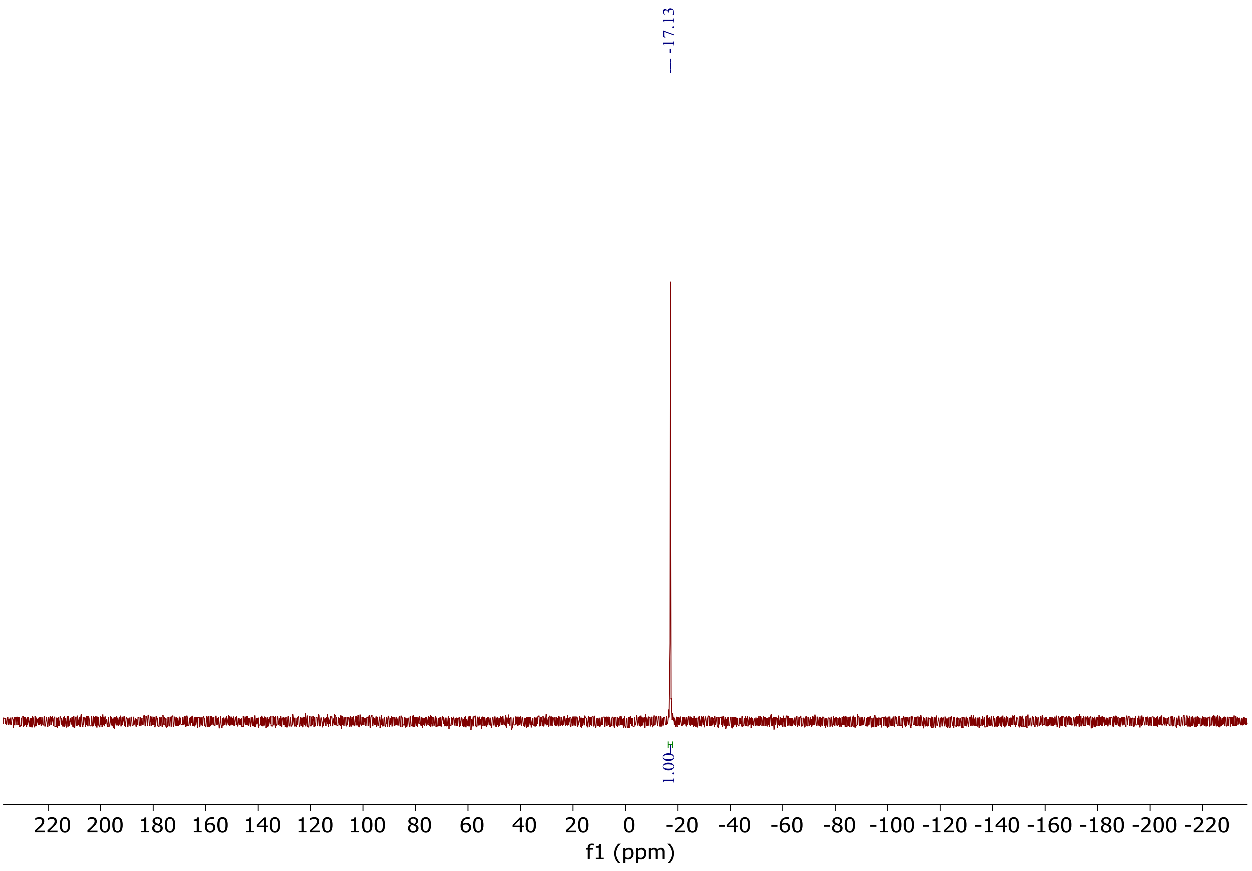
-17.13
1.00
220 200 180 160 140 120 100 80 60 40 20 0 -20 -40 -60 -80 -100 -120 -140 -160 -180 -200 -220
f1 (ppm)

IDPi 7b

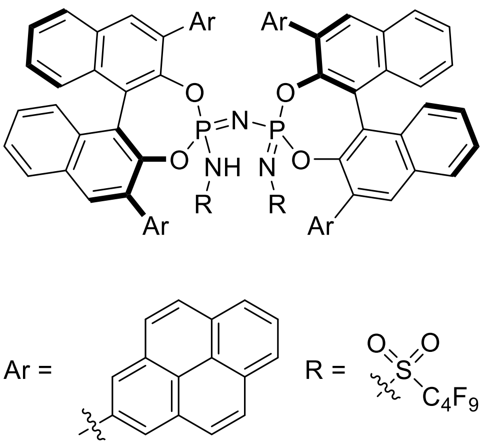

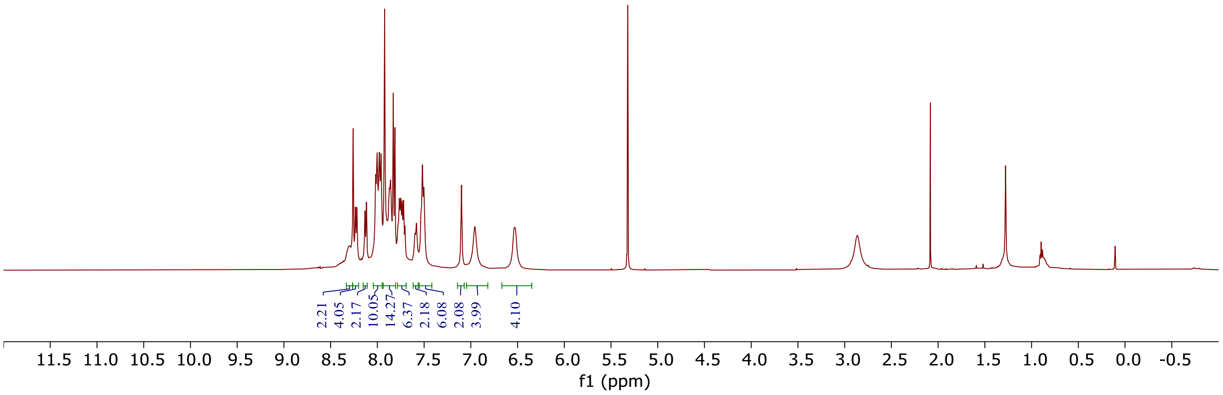
8.26
8.24
8.22
8.13
8.12
8.02
8.00
7.98
7.96
7.92
7.89
7.88
7.87
7.86
7.83
7.81
7.78
7.76
7.75
7.73
7.72
7.70
7.58
7.53
7.52
7.50
7.10
6.96
6.53
5.32 CD2Cl2
2.21
4.05
2.17
10.05
14.27
6.37
2.18
6.08
2.08
3.99
4.10
f1 (ppm)

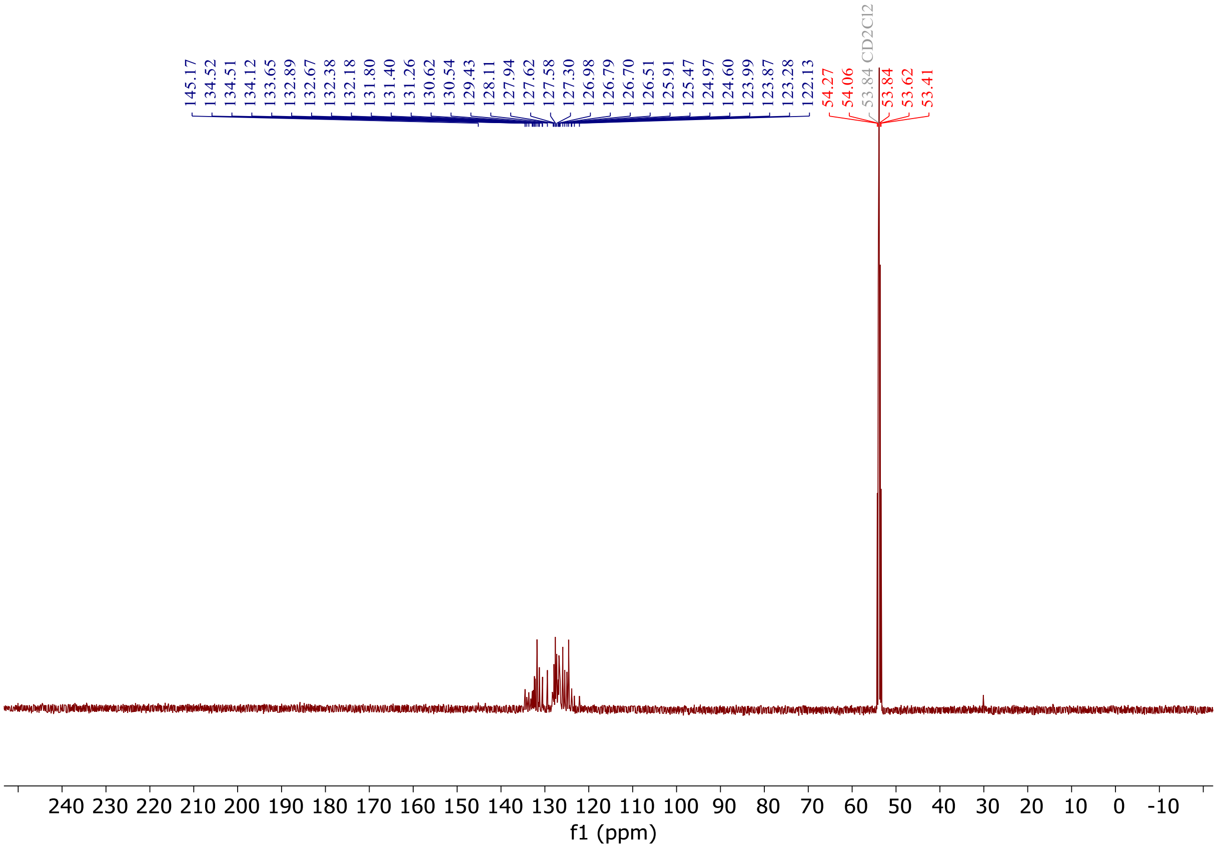
145.17
134.52
134.51
134.12
133.65
132.89
132.67
132.38
132.18
131.80
131.40
131.26
130.62
130.54
129.43
128.11
127.94
127.62
127.58
127.30
126.98
126.79
126.70
126.51
125.91
125.47
124.97
124.60
123.99
123.87
123.28
122.13
54.27
54.06
53.84 CD2Cl2
53.84
53.62
53.41
f1 (ppm)

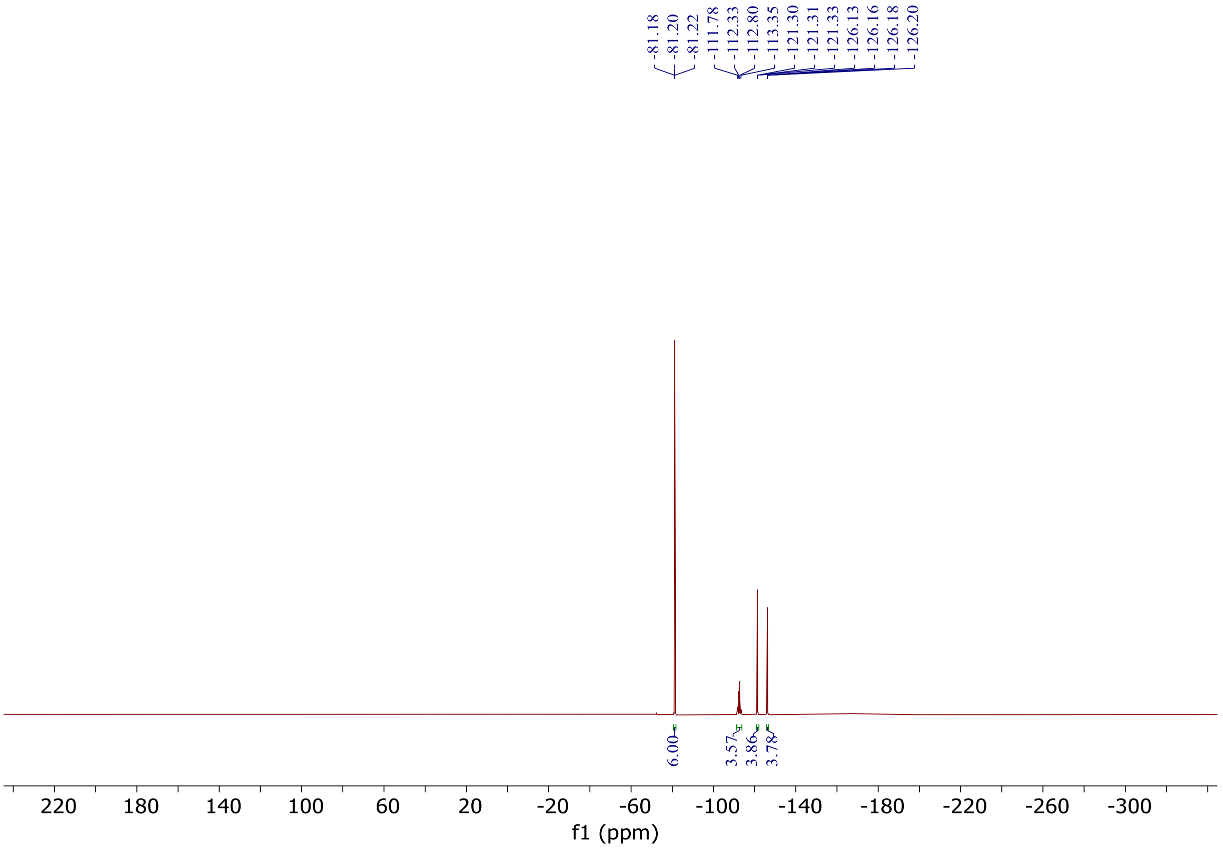
-81.18
-81.20
-81.22
-111.78
-112.33
-112.80
-113.35
-121.30
-121.31
-121.33
-126.13
-126.16
-126.18
-126.20
6.00
3.57
3.86
3.78
220 180 140 100 60 20 -20 -60 -100 -140 -180 -220 -260 -300
f1 (ppm)

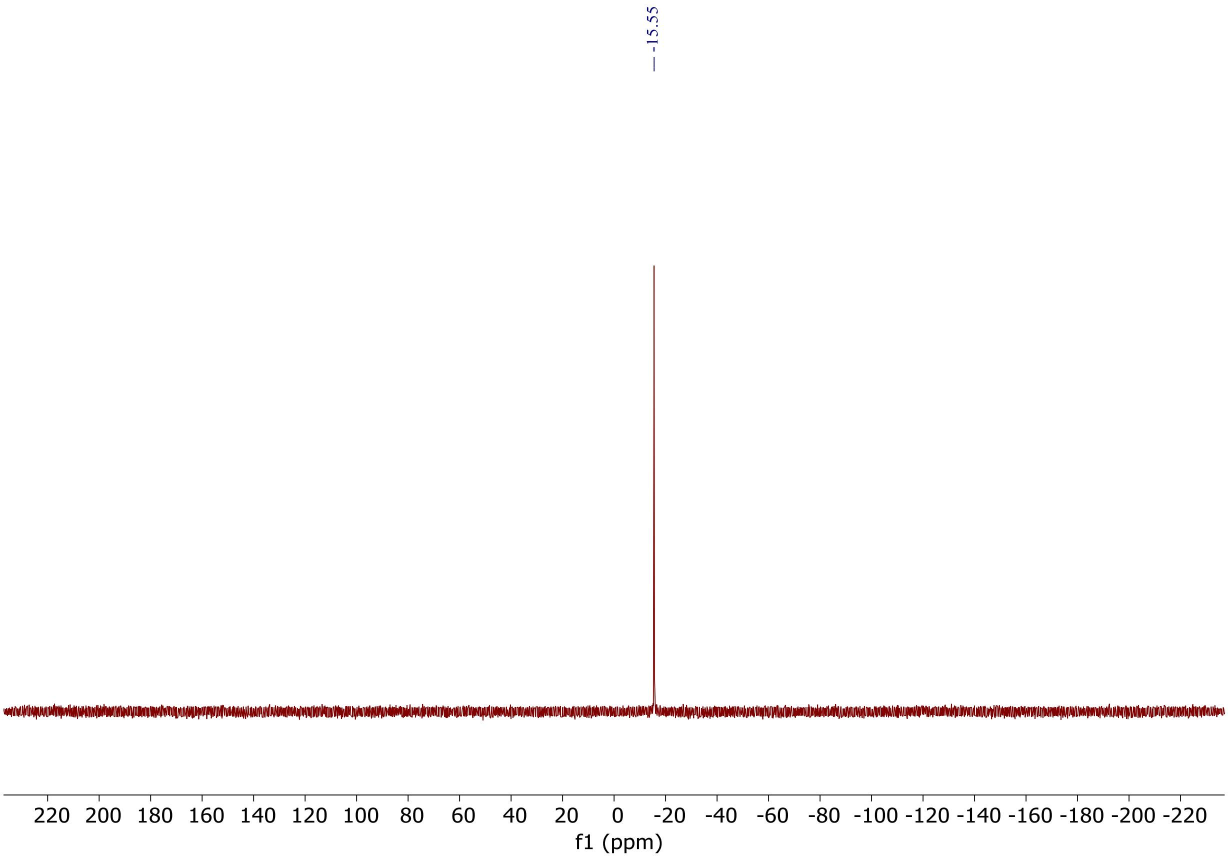
-15.55
220 200 180 160 140 120 100 80 60 40 20 0 -20 -40 -60 -80 -100 -120 -140 -160 -180 -200 -220
f1 (ppm)

5-methylhex-5-enal (1a)

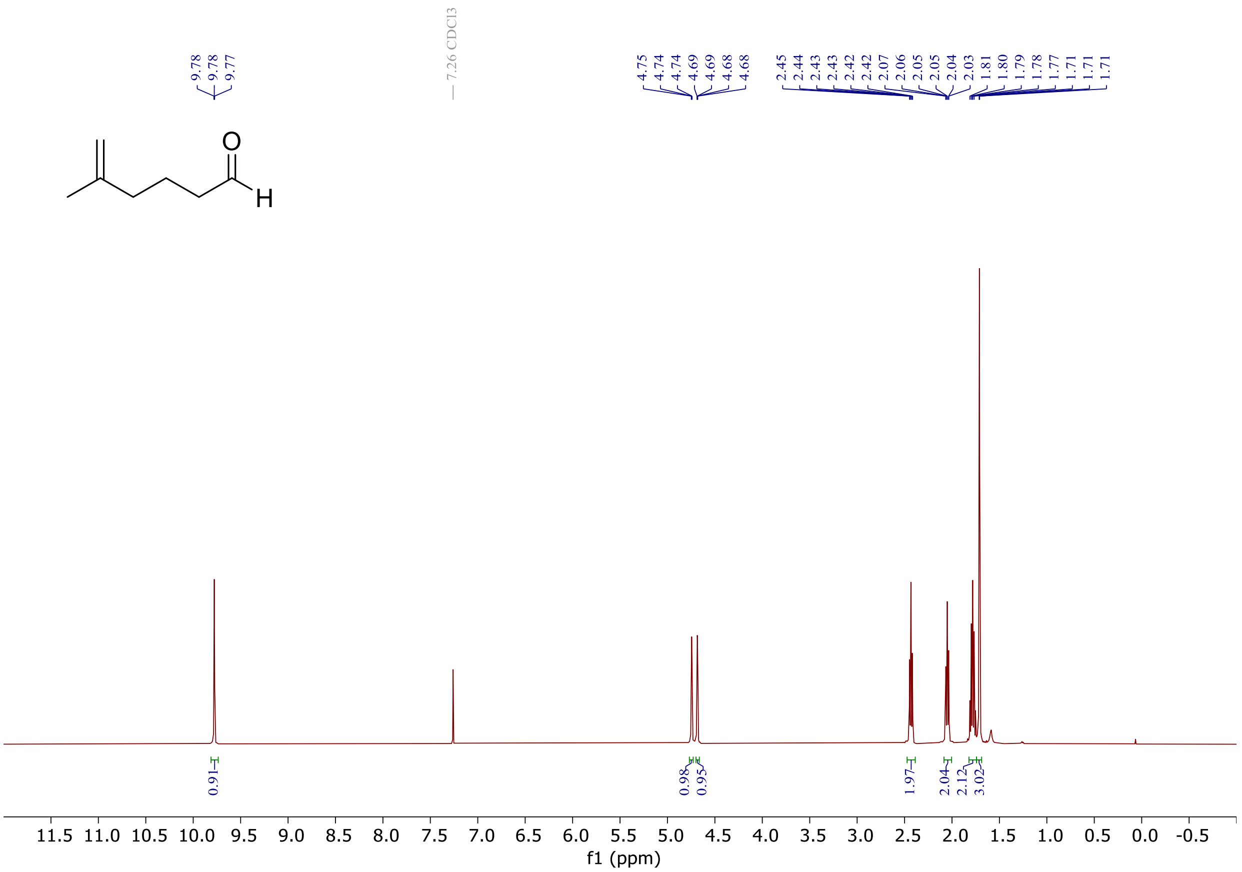

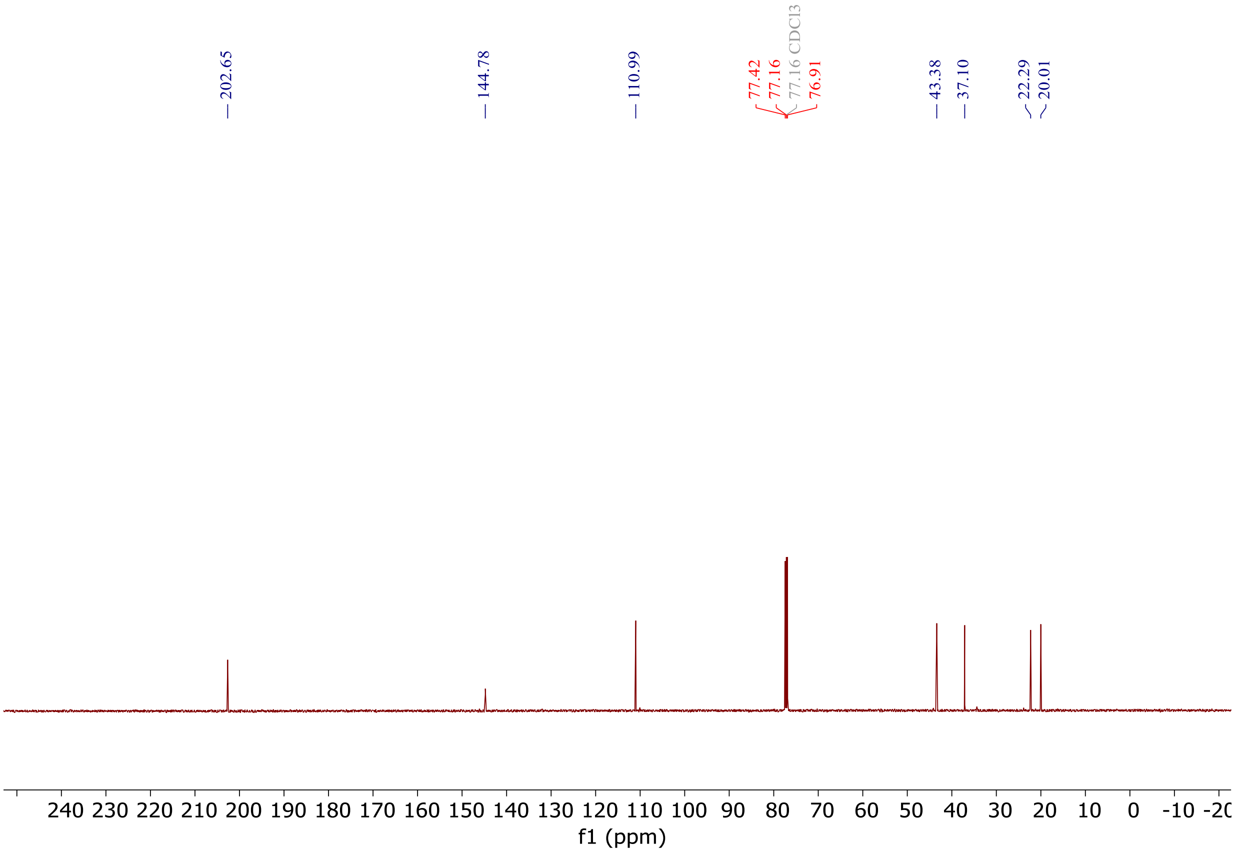

5-methyleneoctanal (1b)

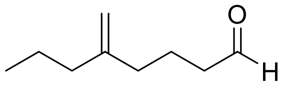

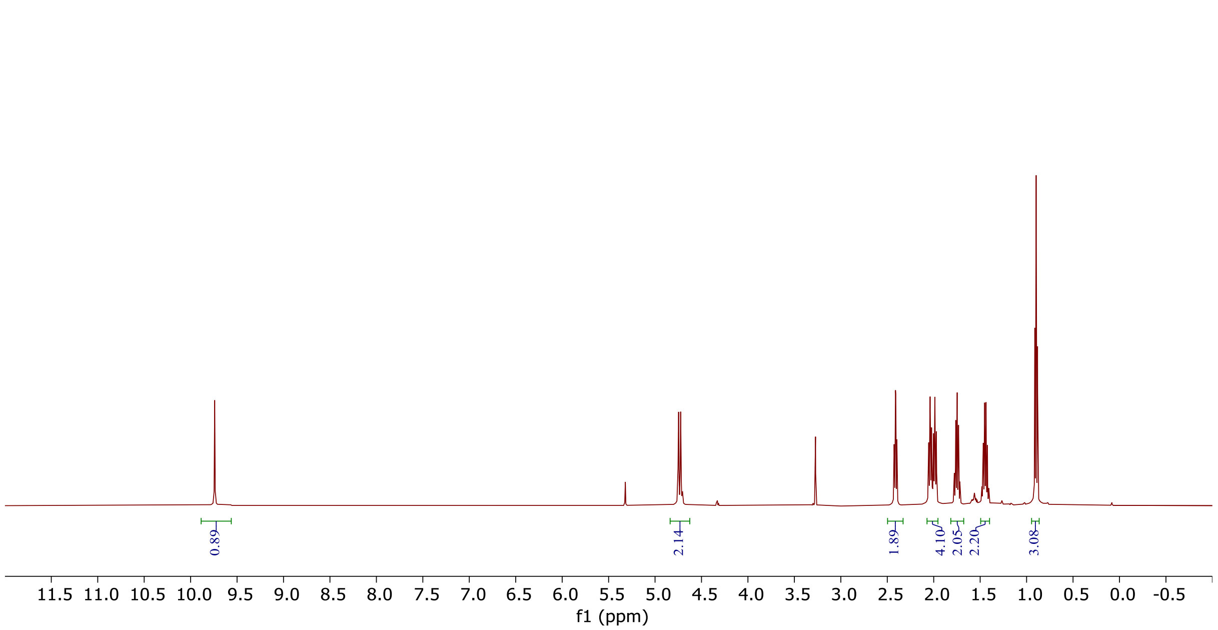

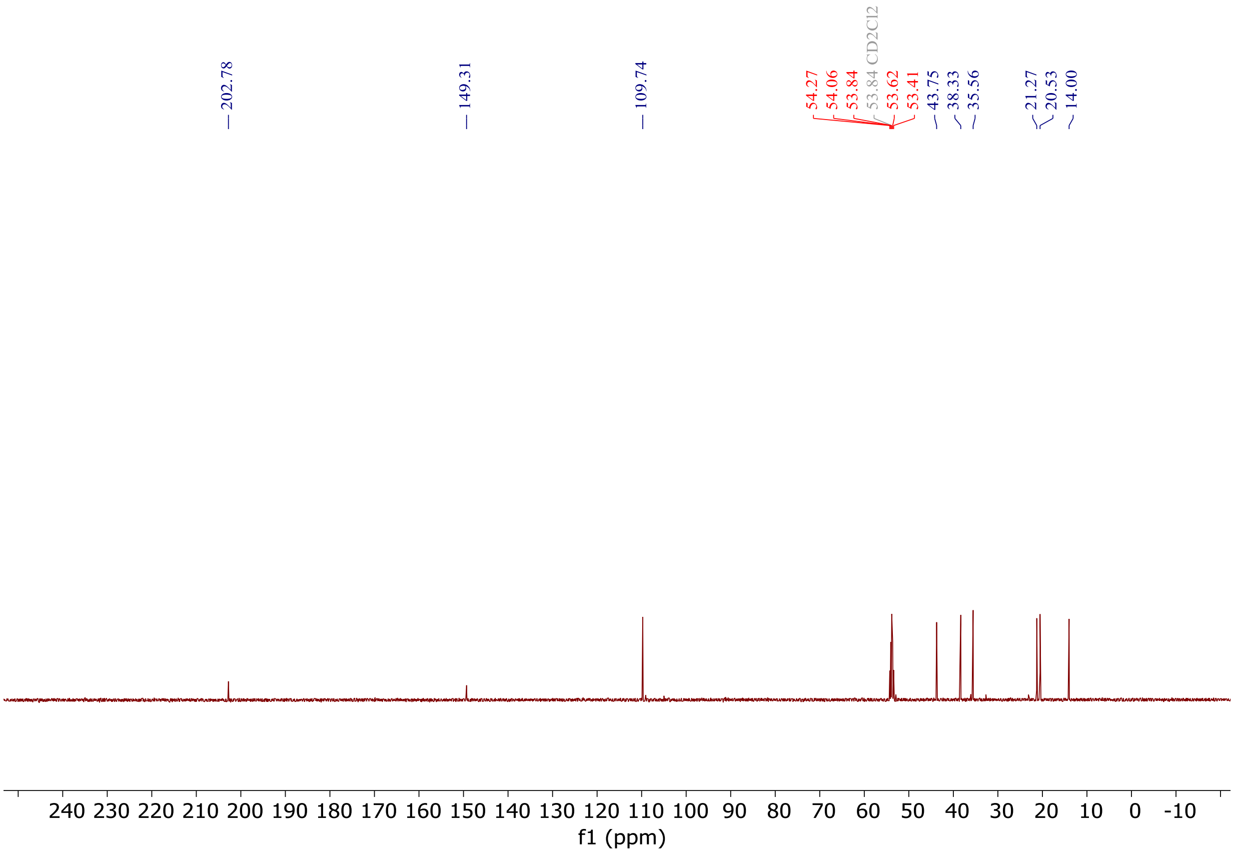

5-methylenedodecanal (1c)

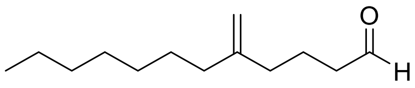

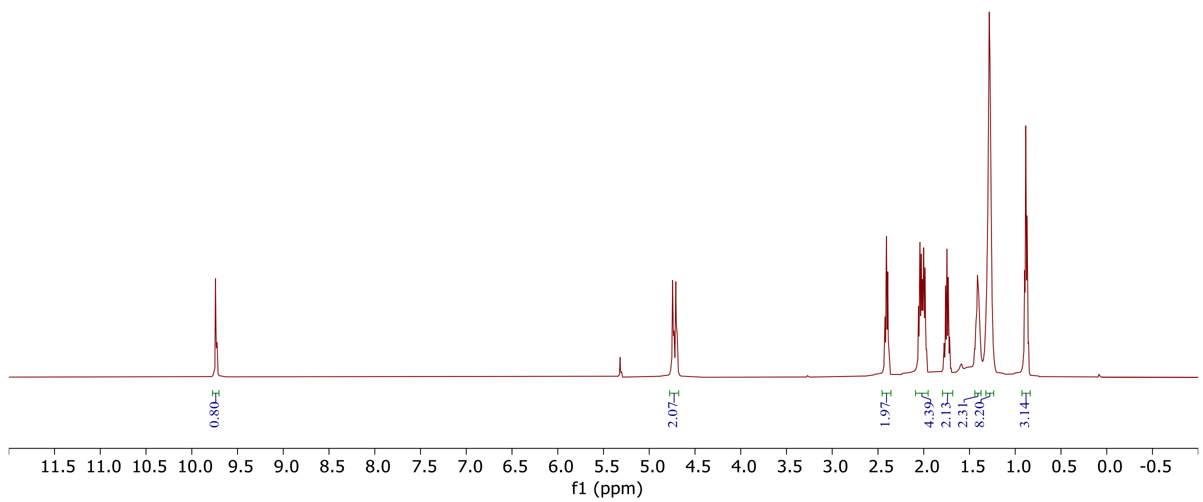

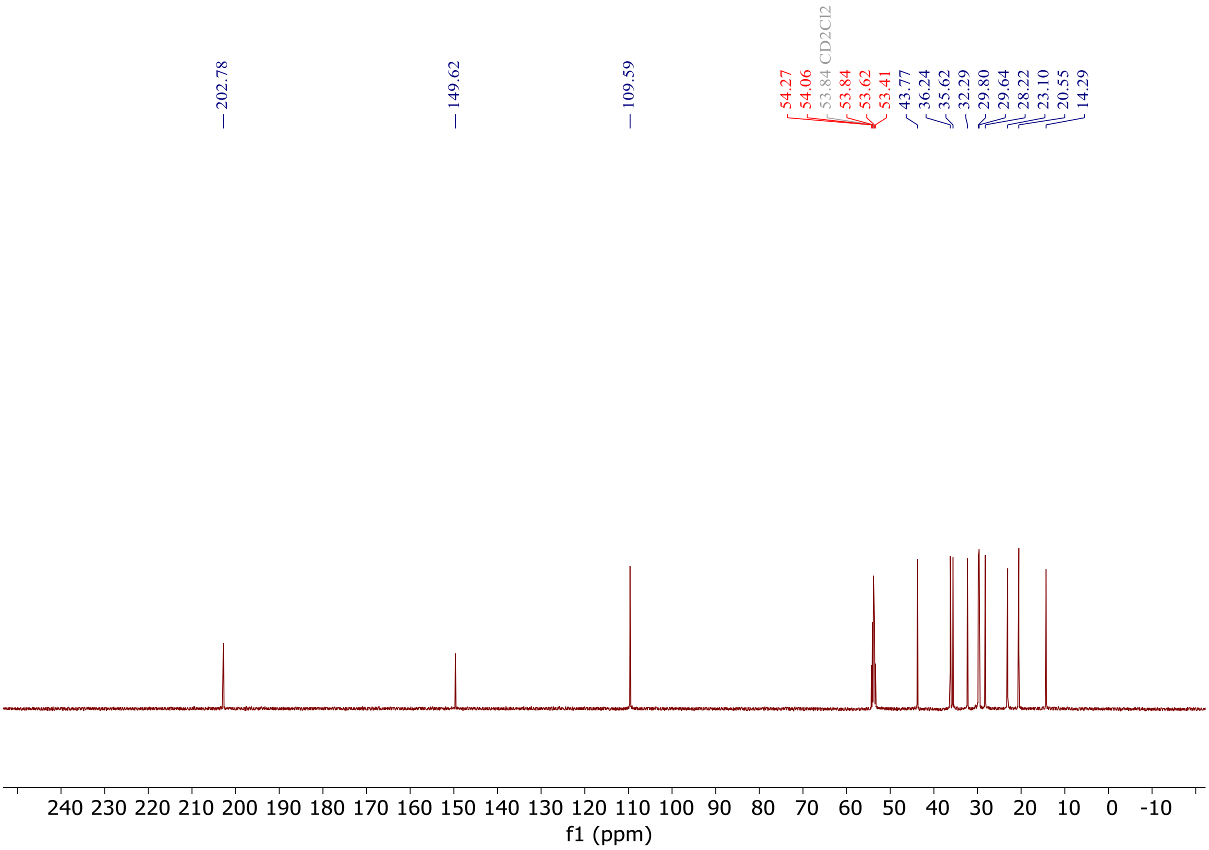

8-methyl-5-methylenenonanal (1d)

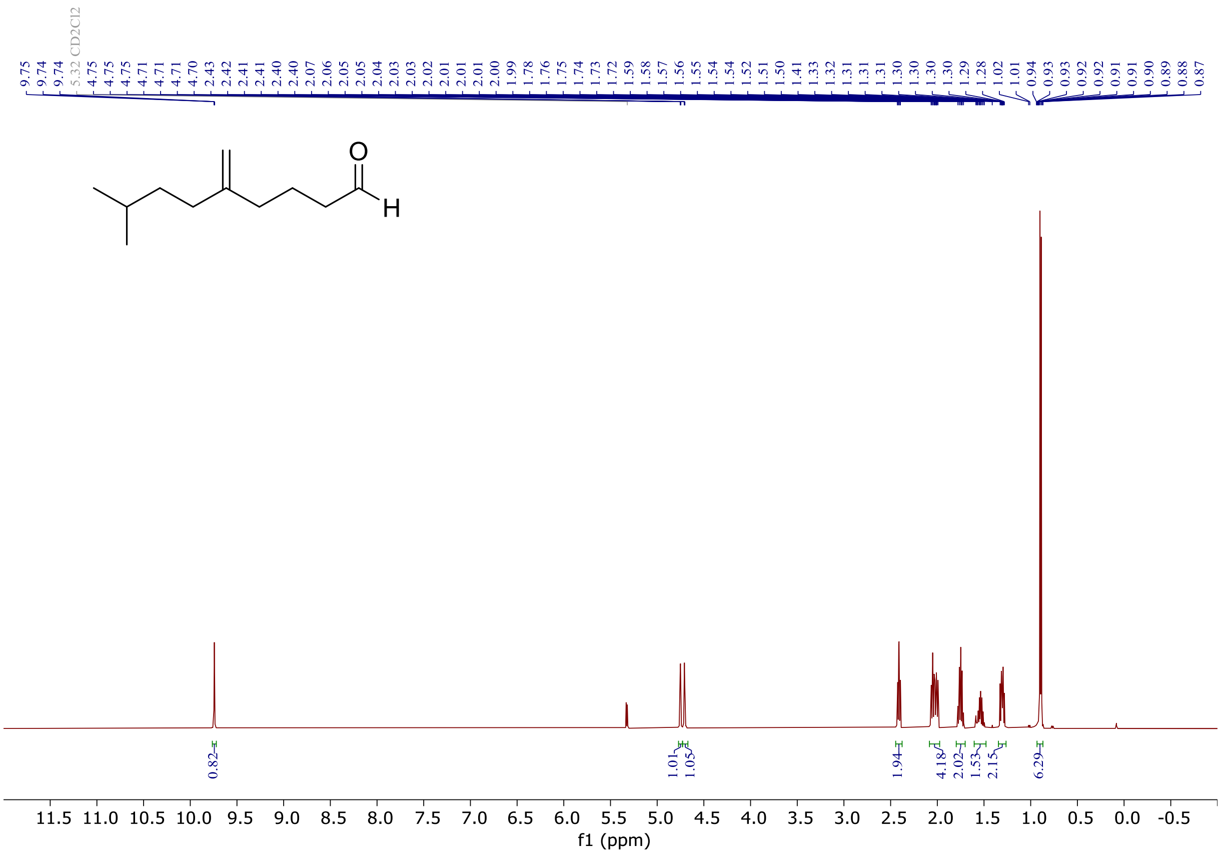

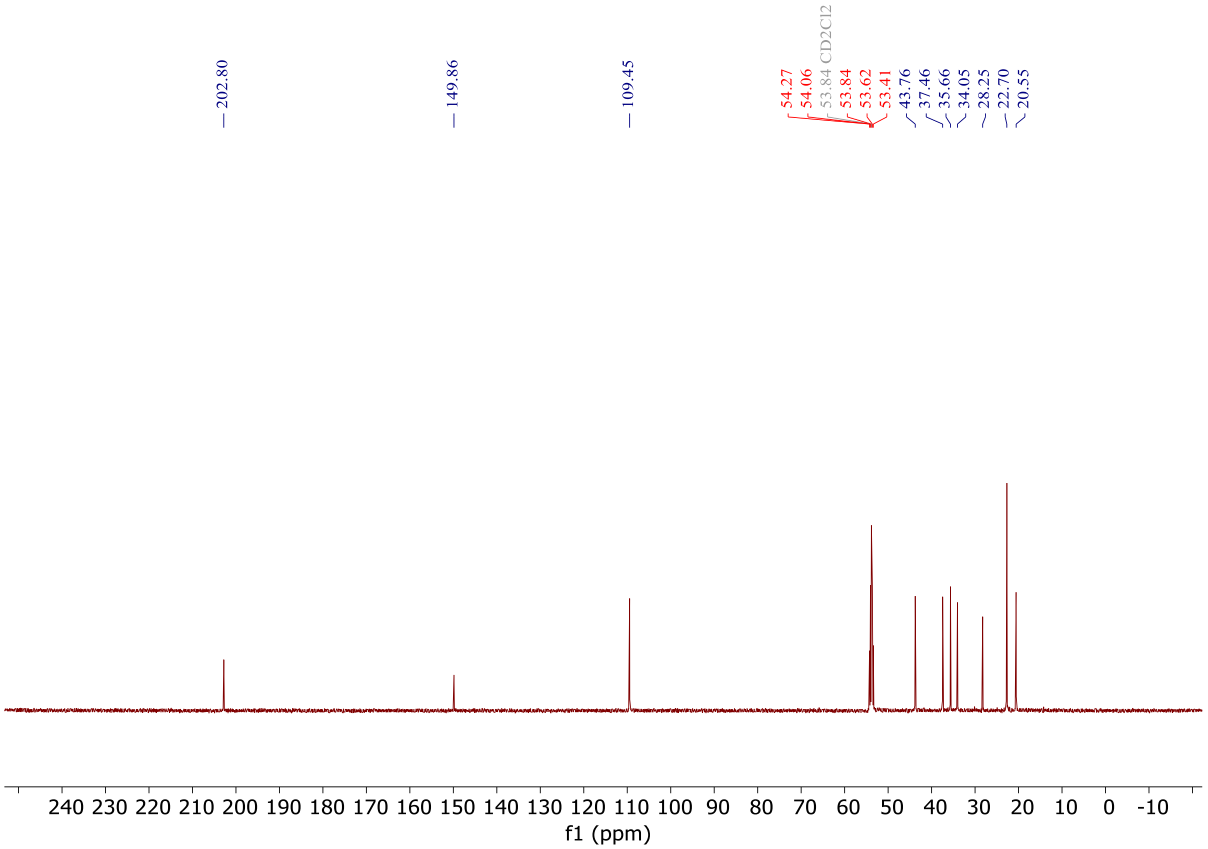

5-methylenenon-8-enal (1e)

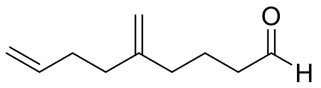

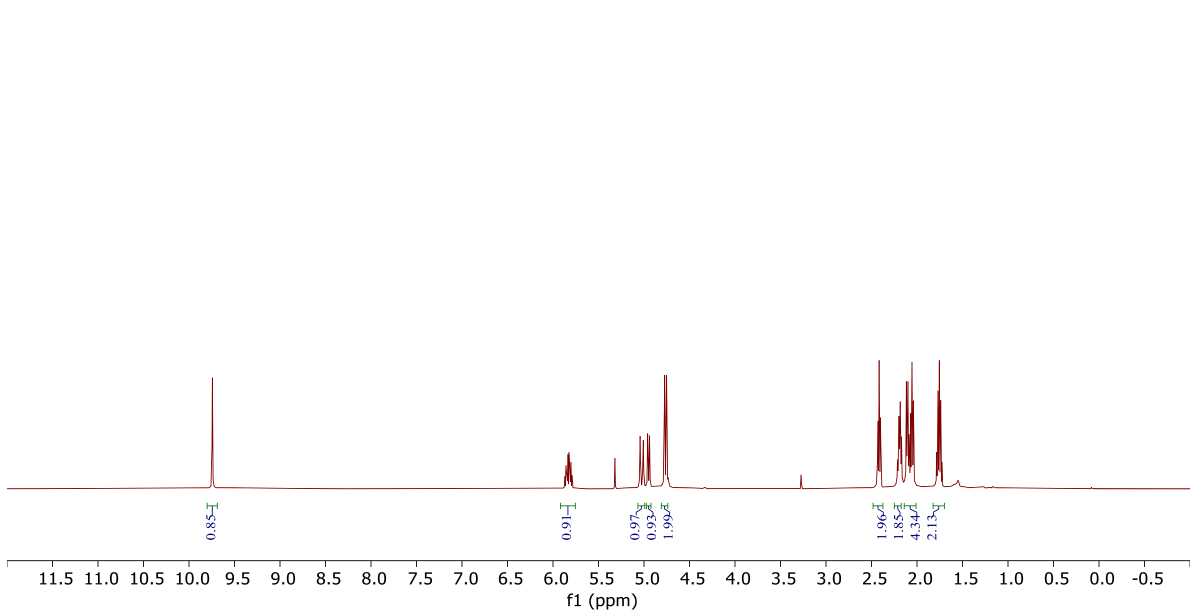

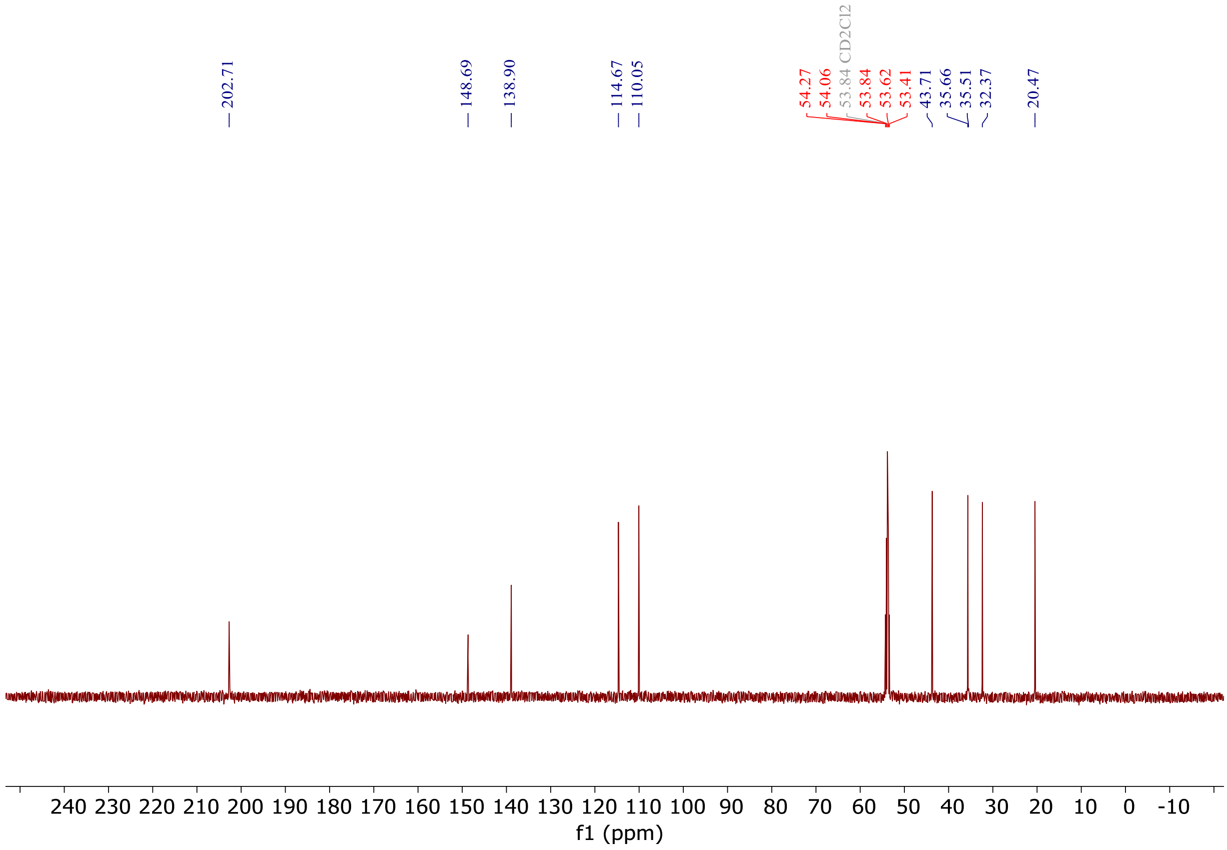

5-methylenedec-9-enal (1f)

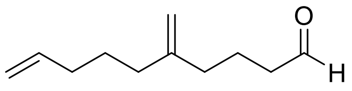

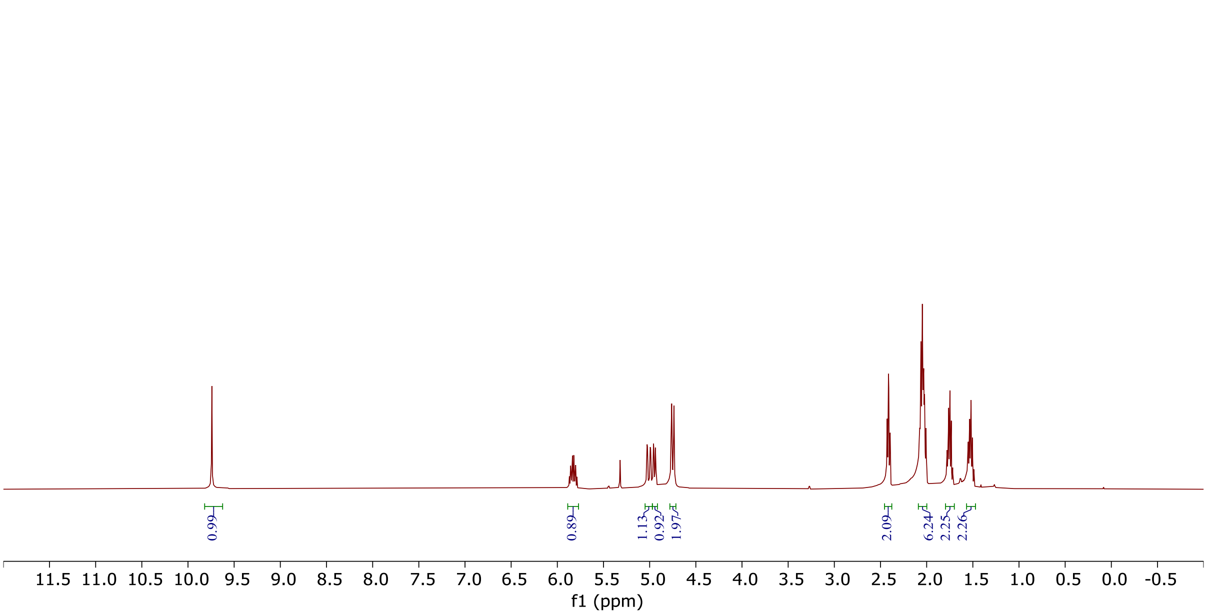

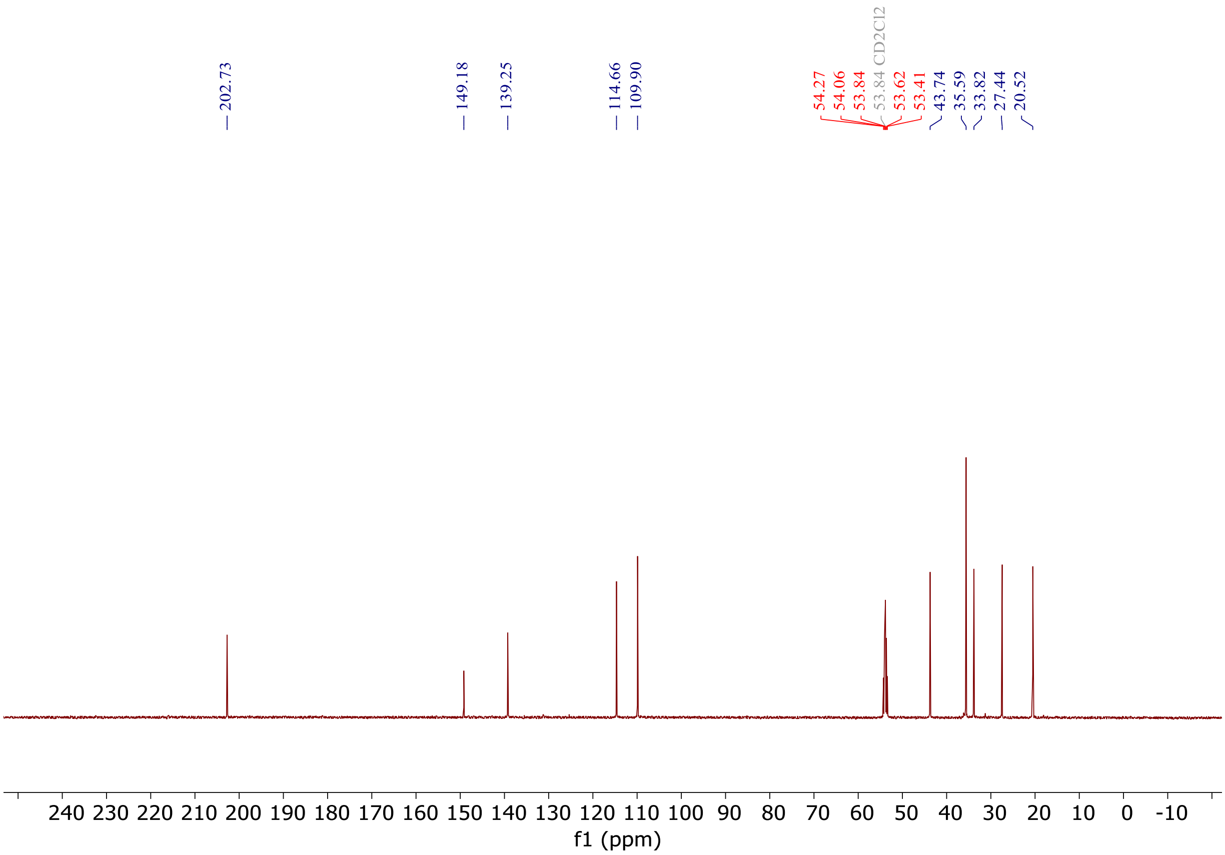

5-methylene-7-phenylheptanal (1g)

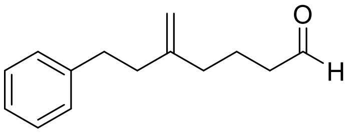

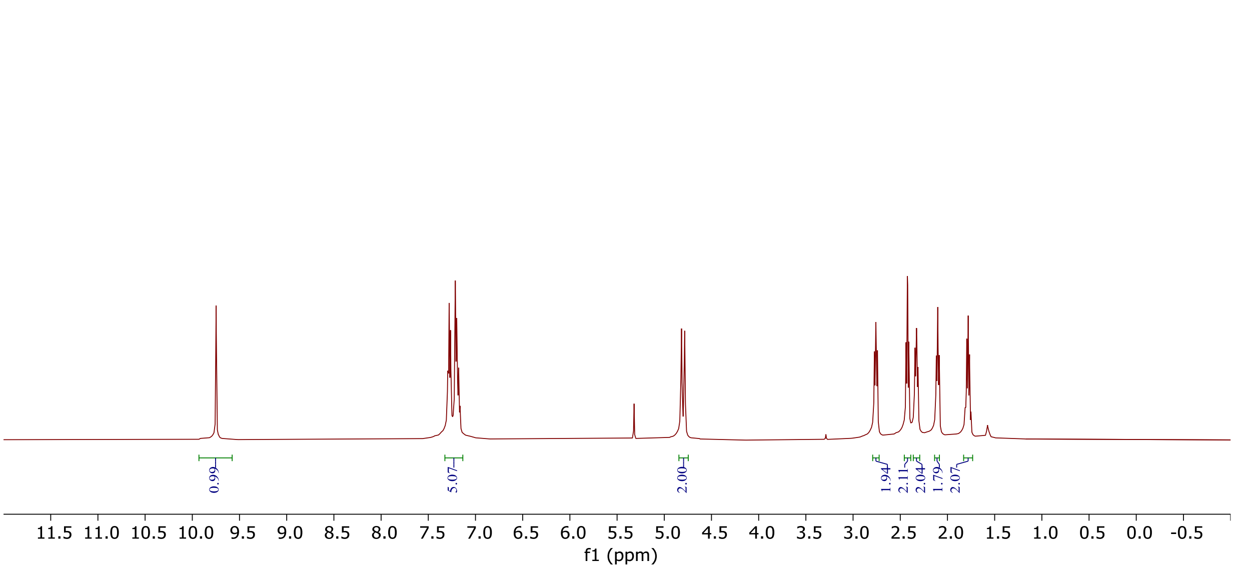

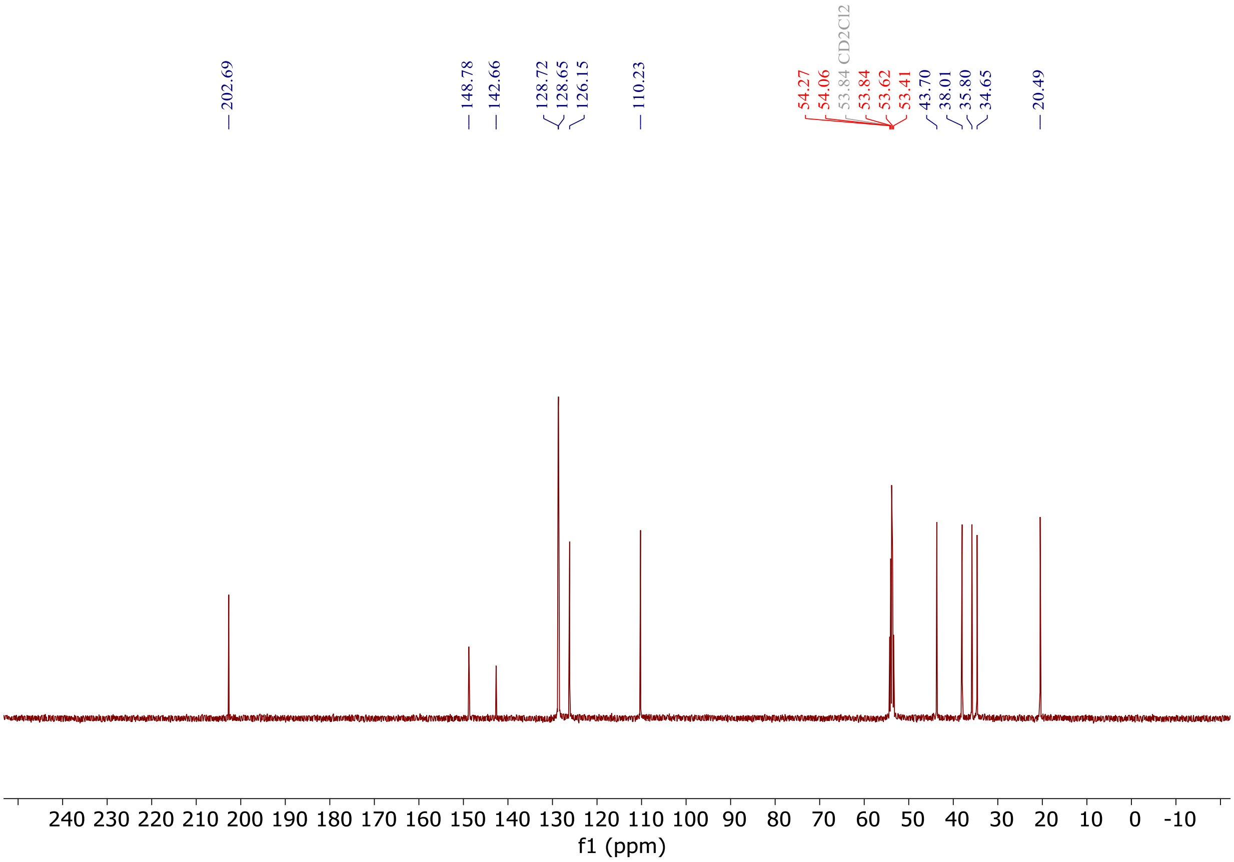

5-methylene-7-(*p*-tolyl)heptanal (1h)

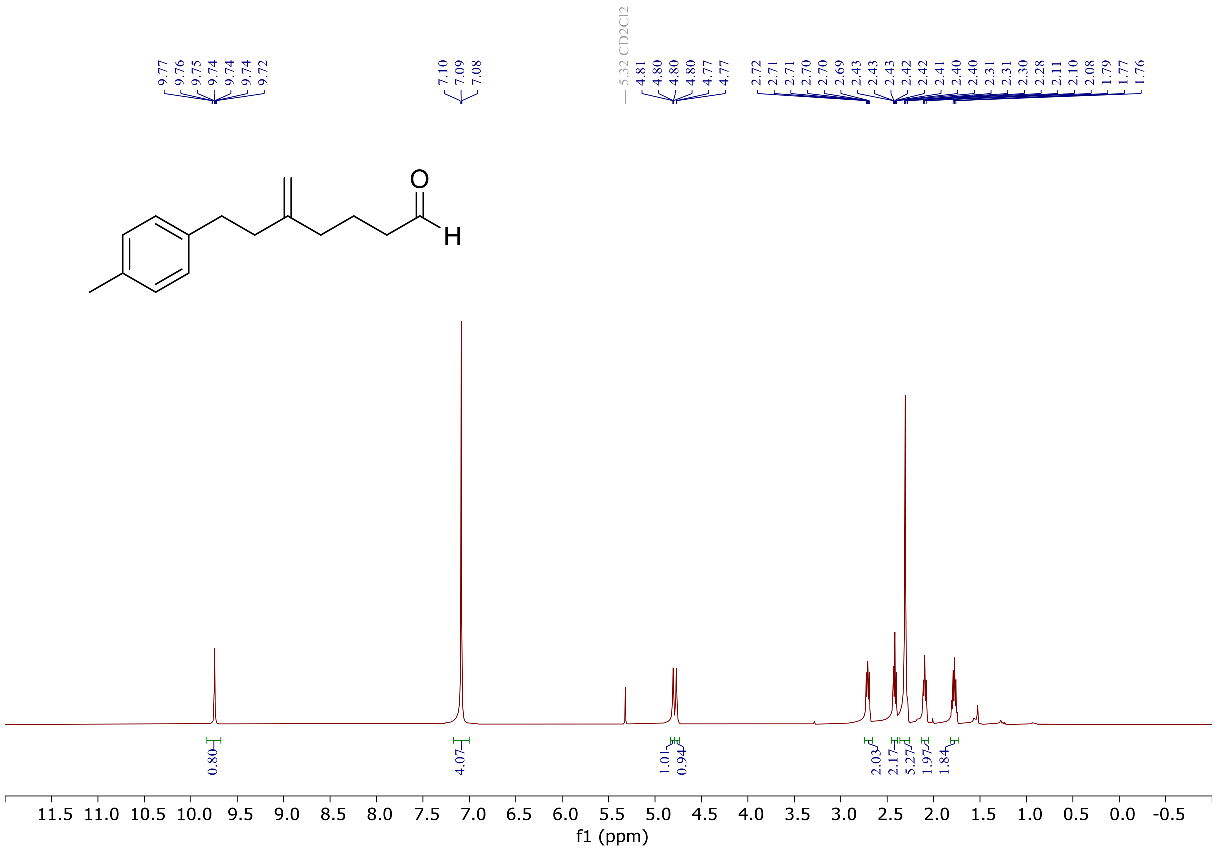

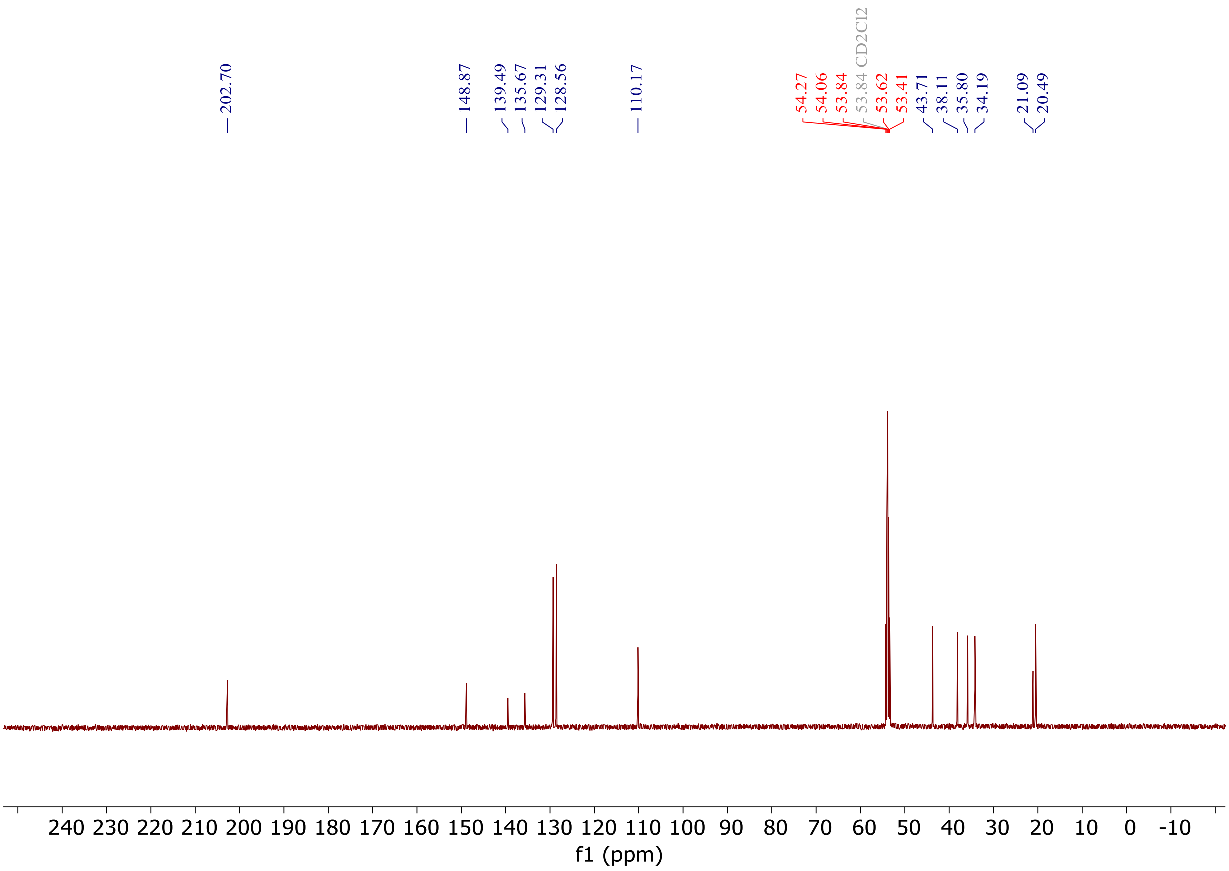

6-methyl-5-methyleneheptanal (1i)

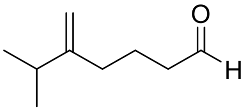

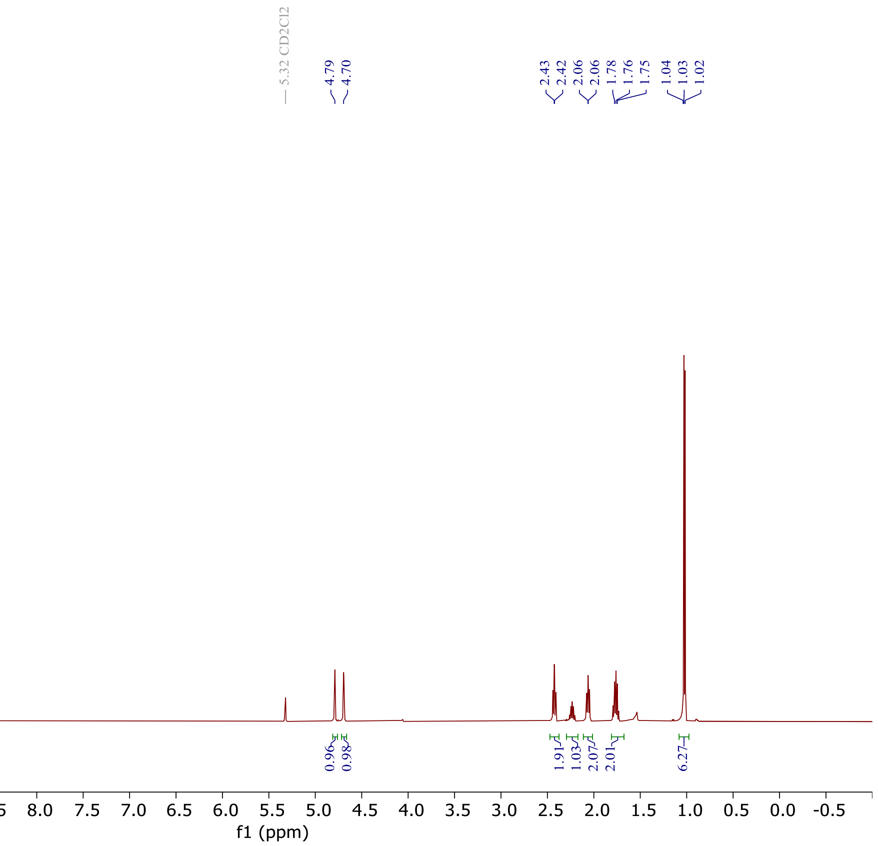

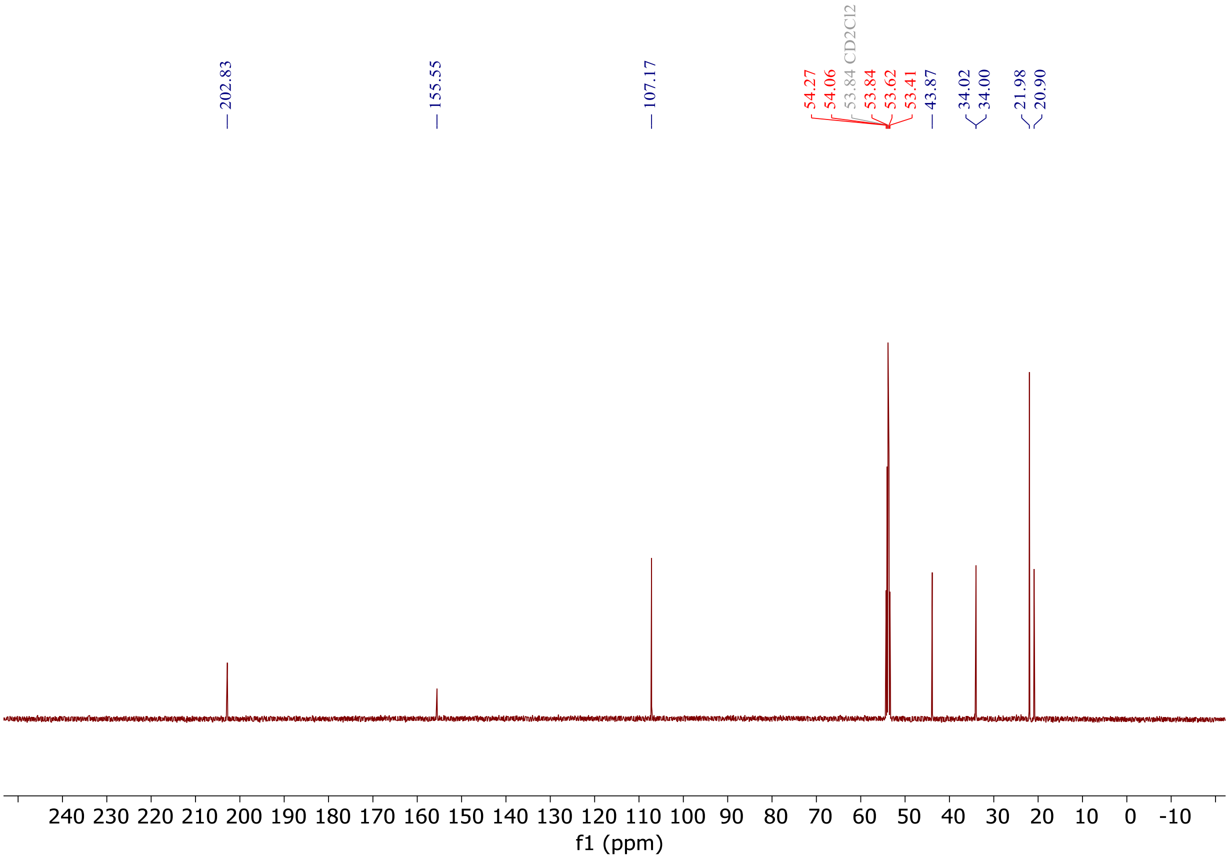

5-cyclopentylhex-5-enal (1j)

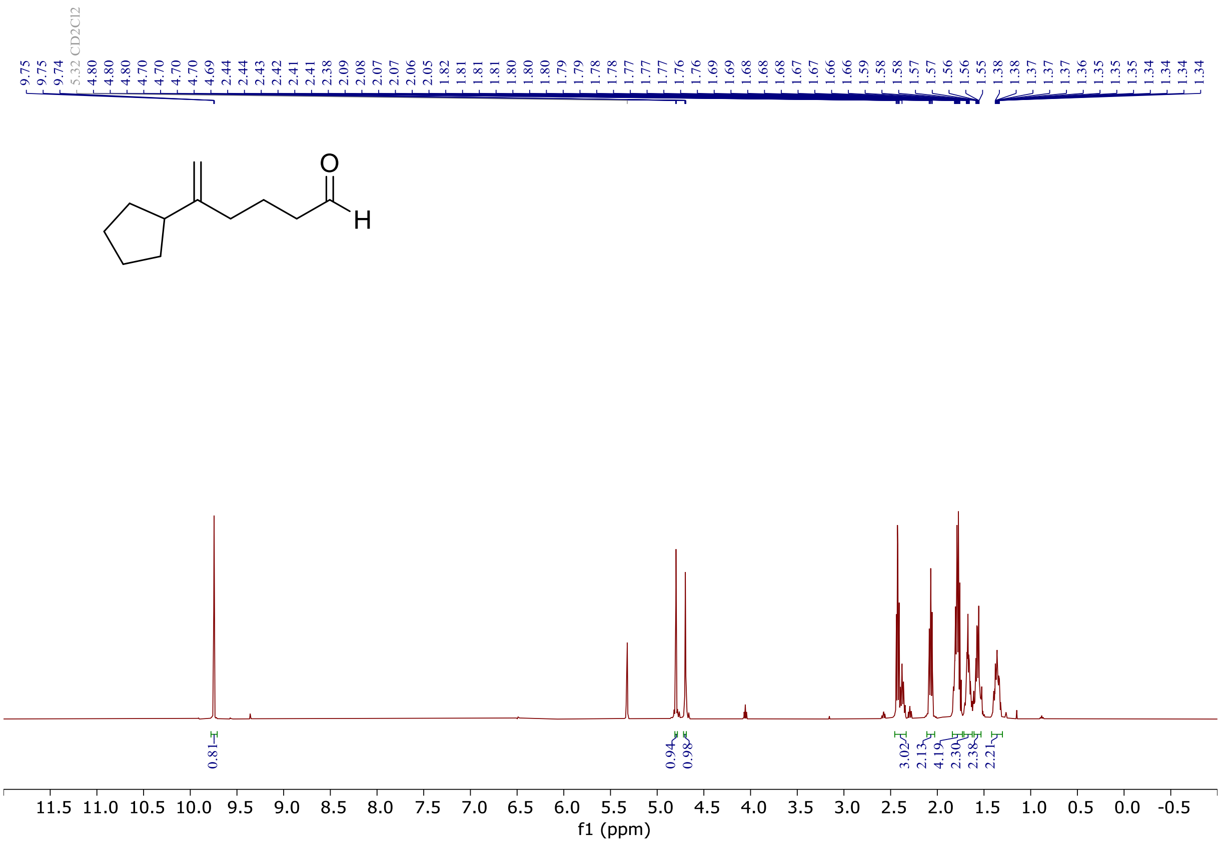

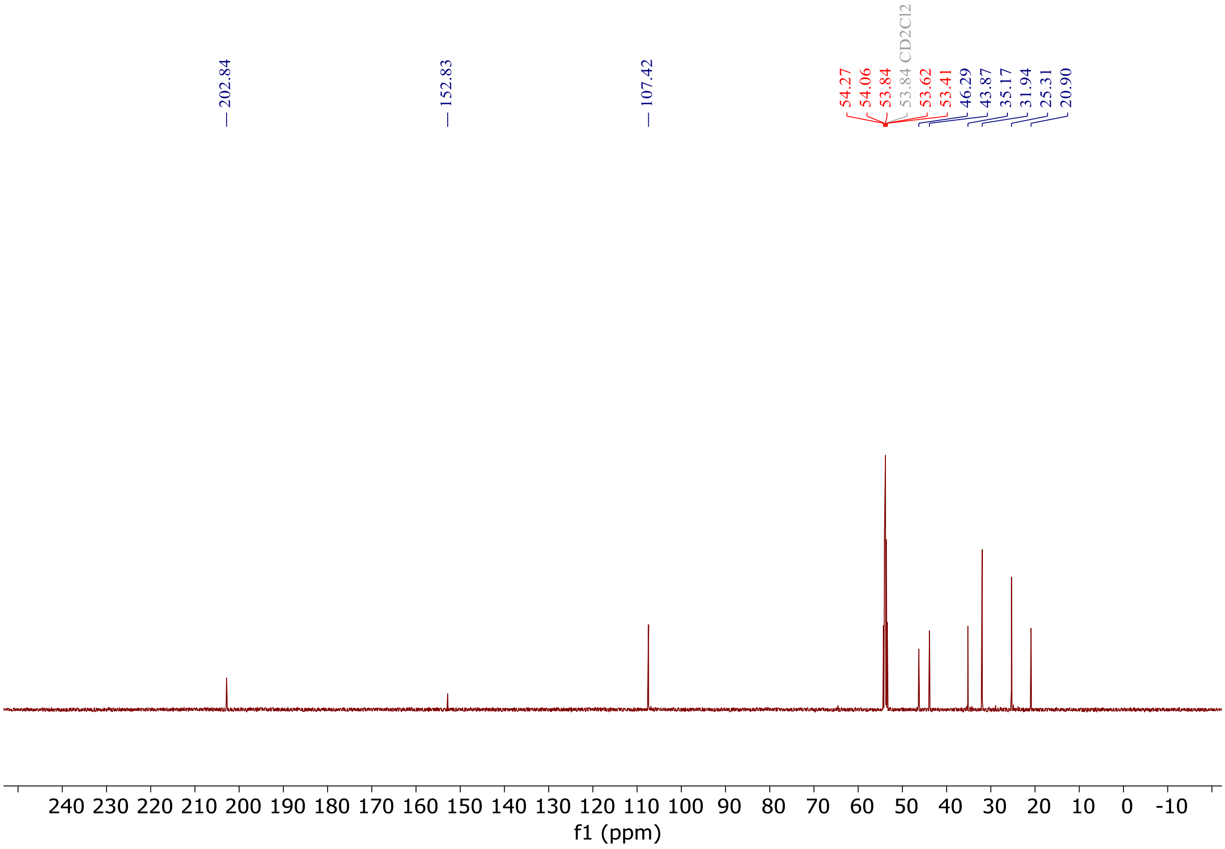

5-cyclohexylhex-5-enal (1k)

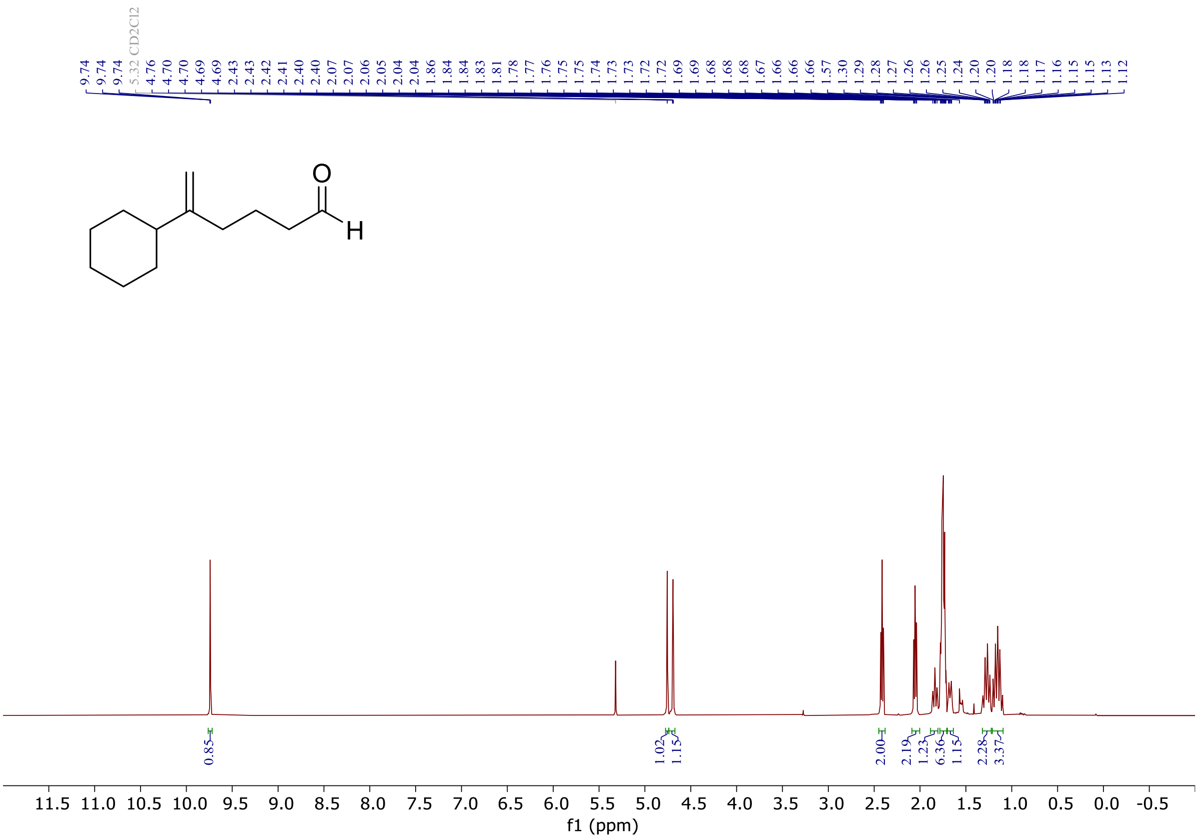

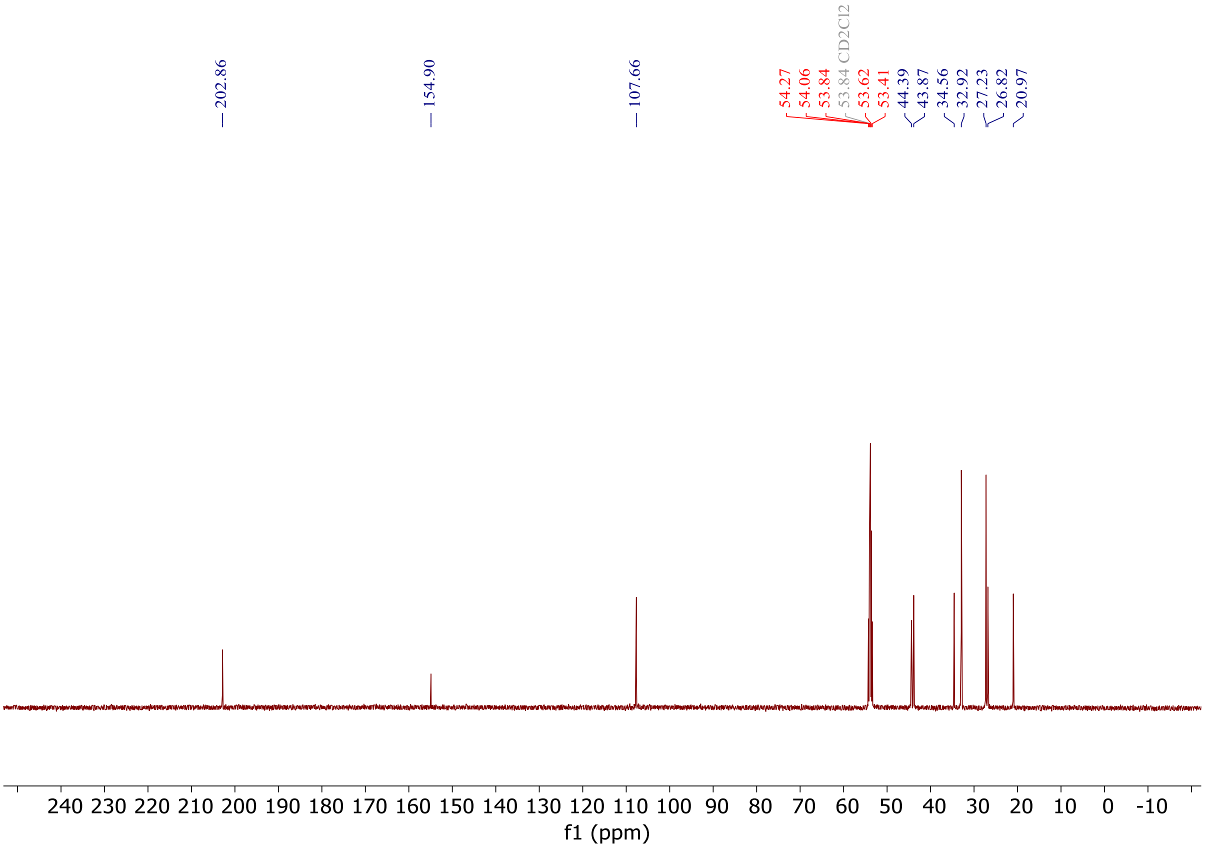

7-methyl-5-methyleneoctanal (1l)

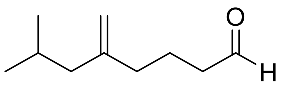

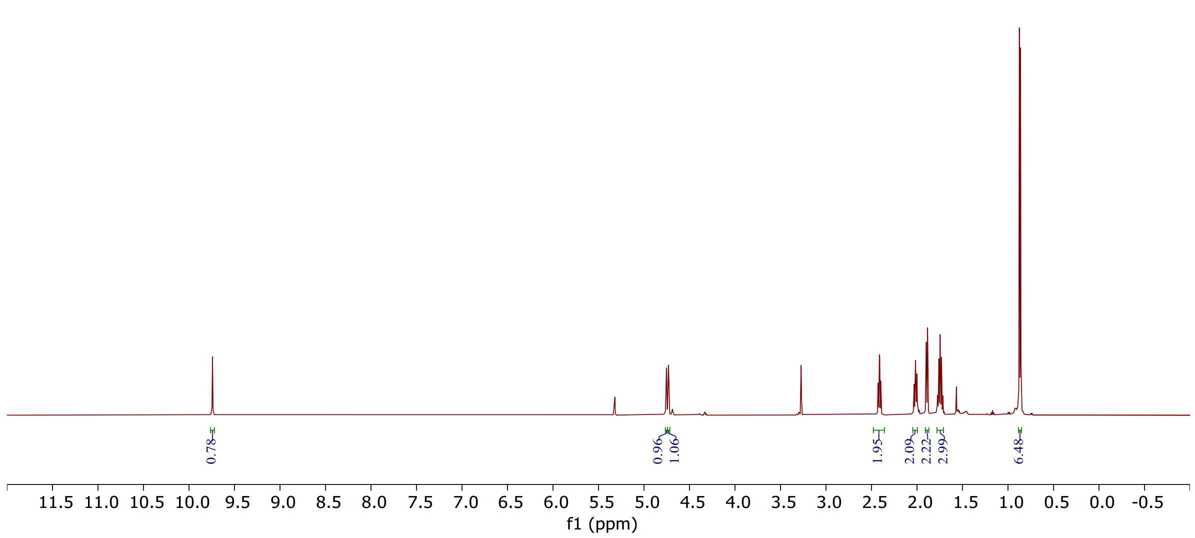

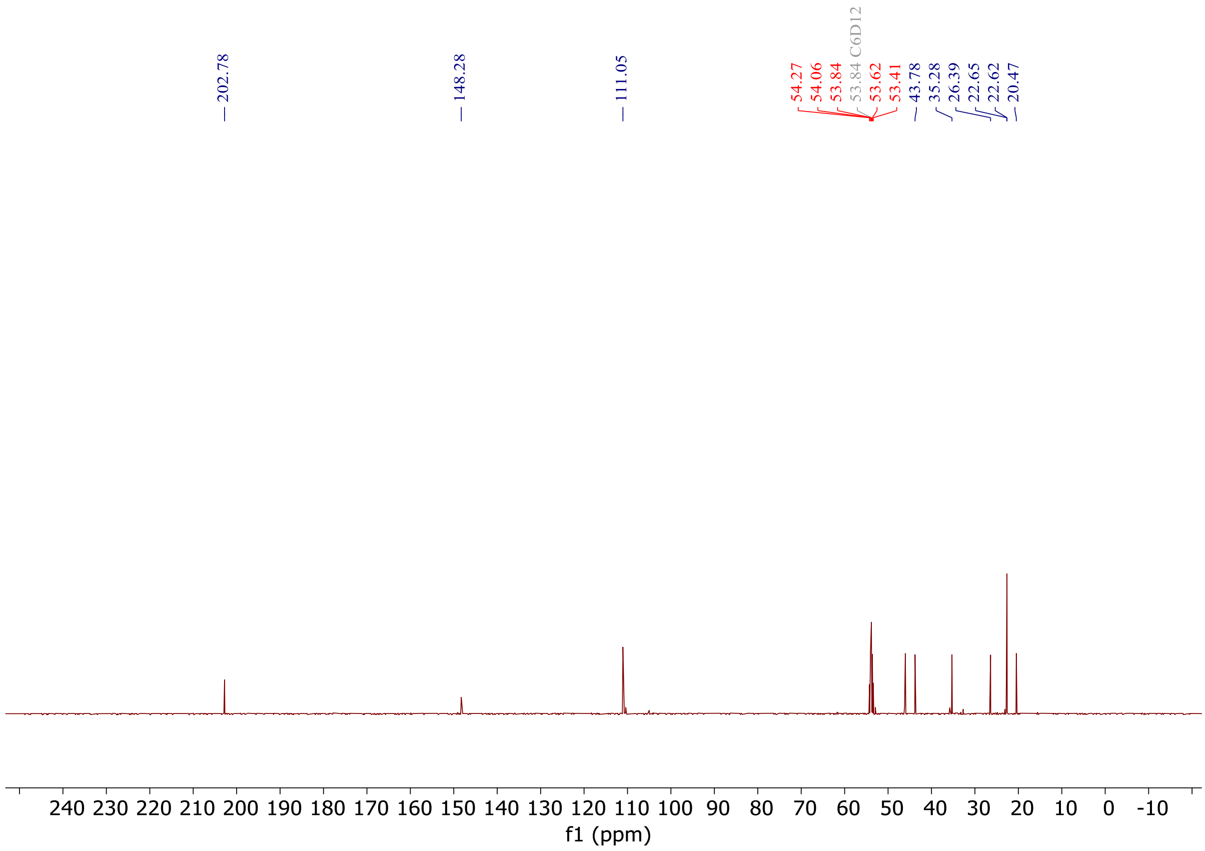

5-(cyclohexylmethyl)hex-5-enal (1m)

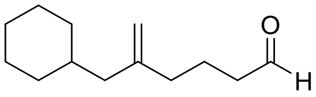

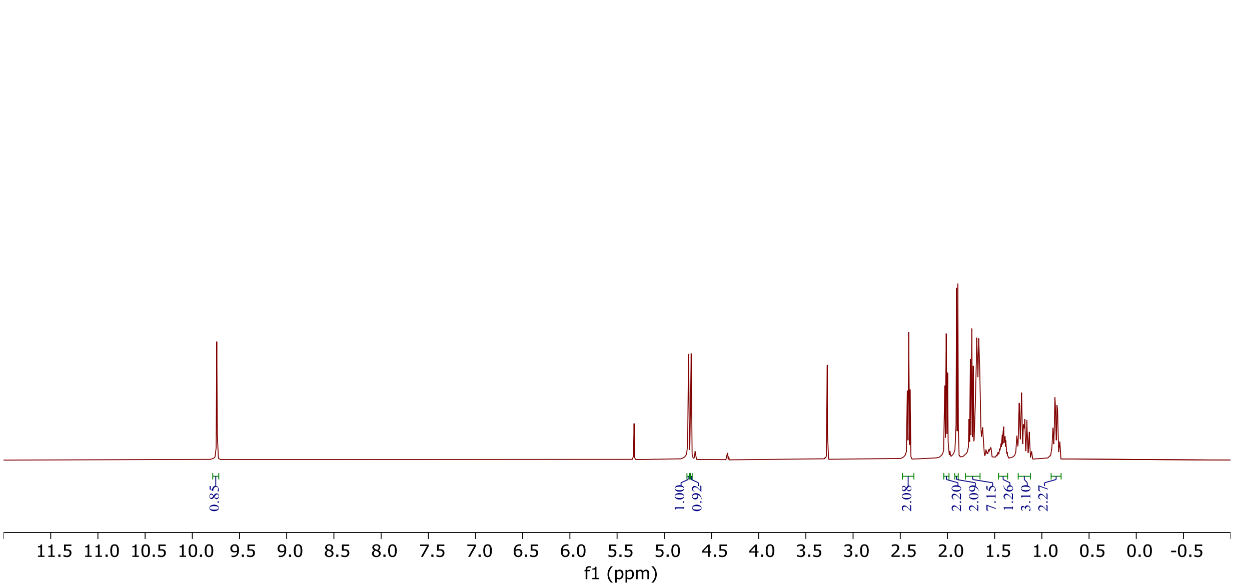

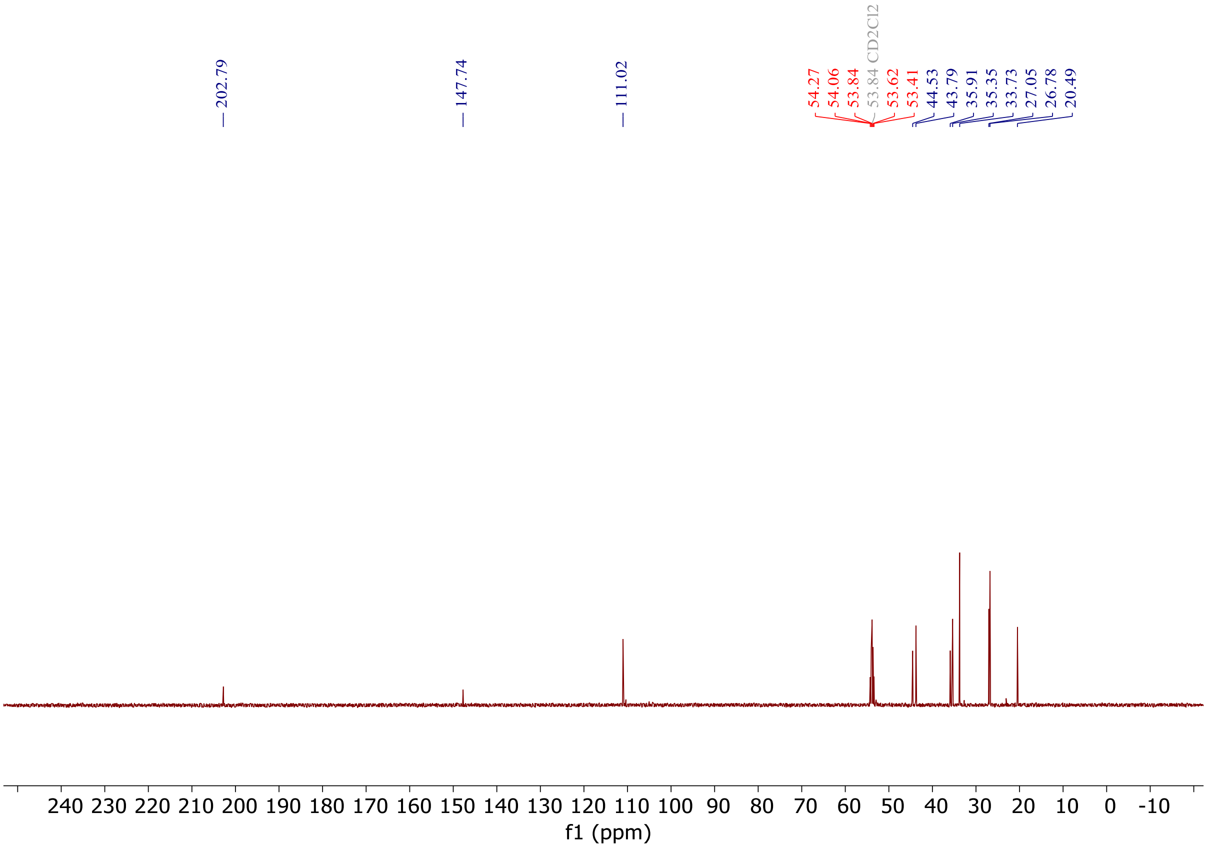

8-methoxy-5-methyleneoctanal (1n)

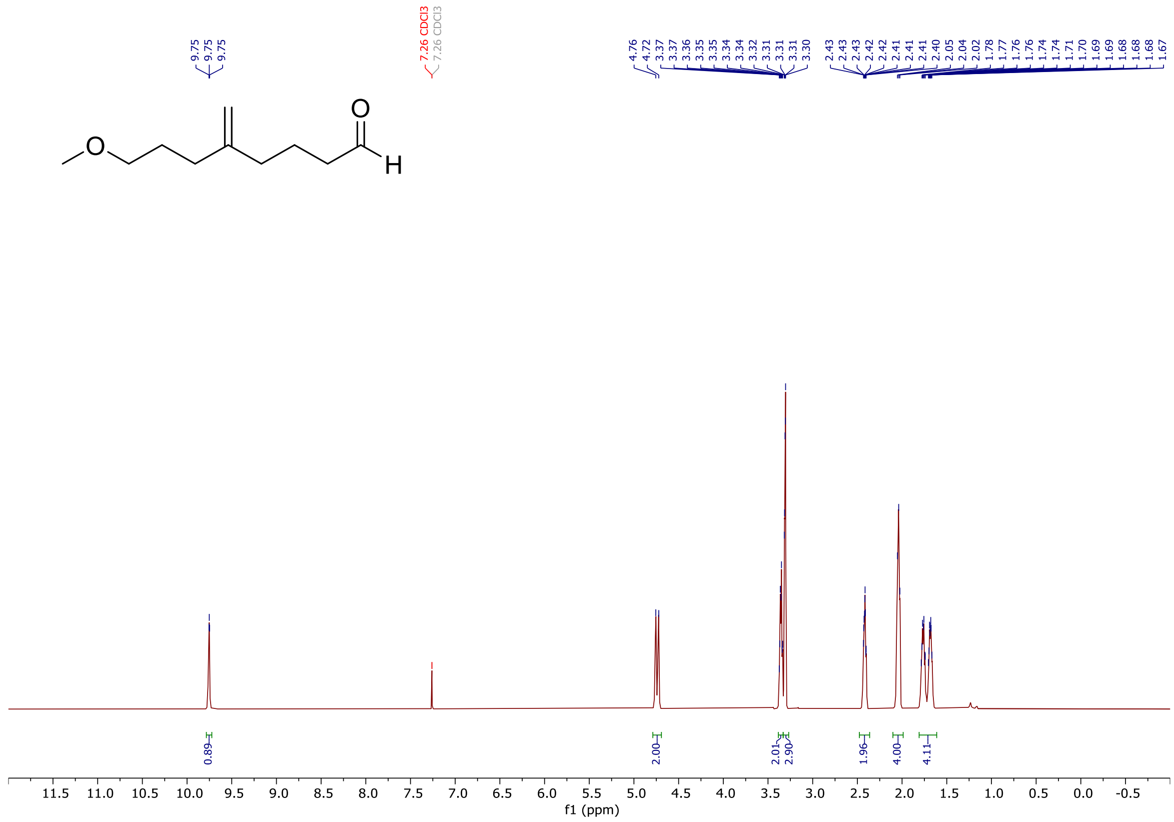

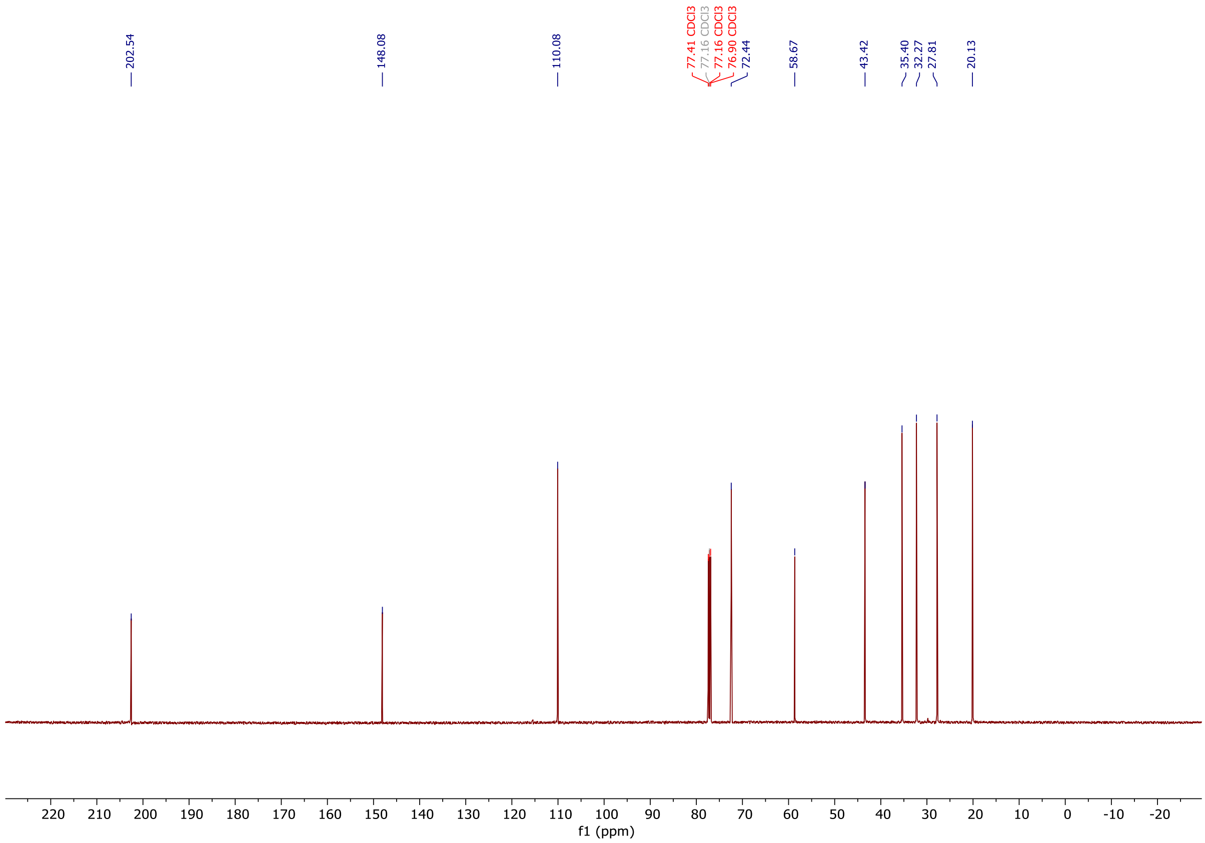

5-methylene-9-(thiophen-2-yl)nonanal (1o)

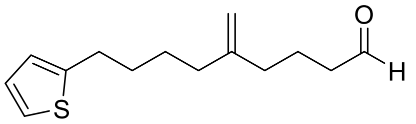

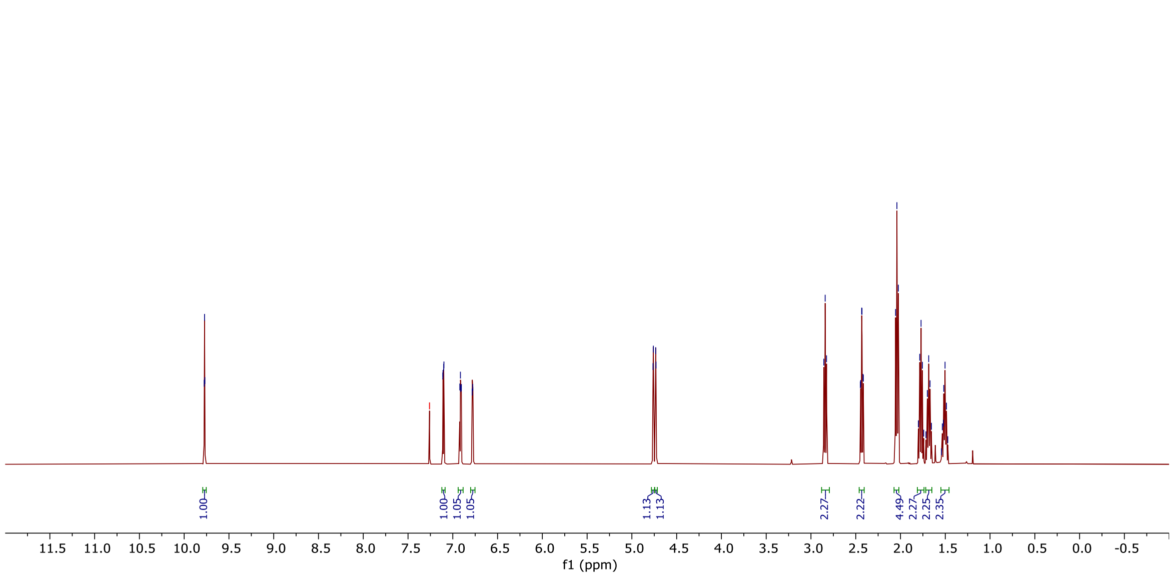

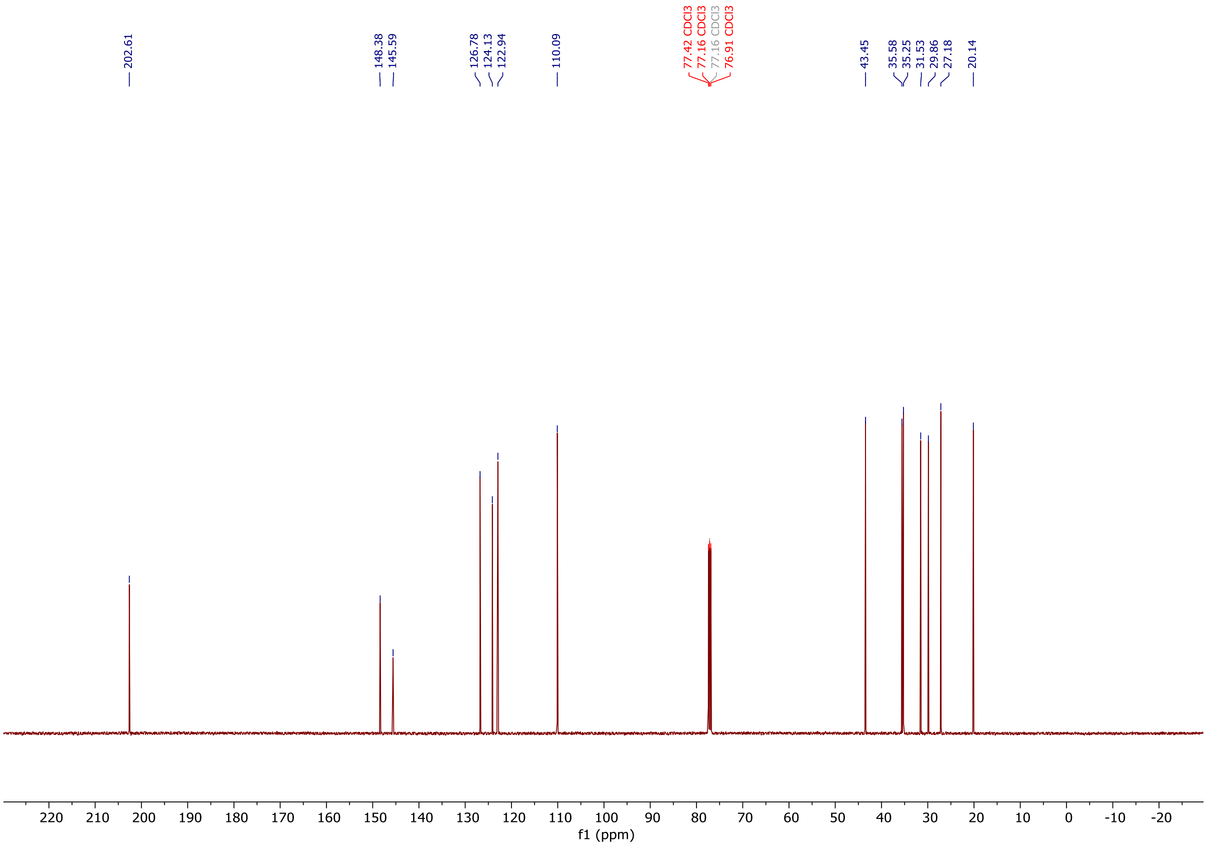

ethyl 9-((*tert*-butyldimethylsilyl)oxy)-5-oxononanoate (S1)

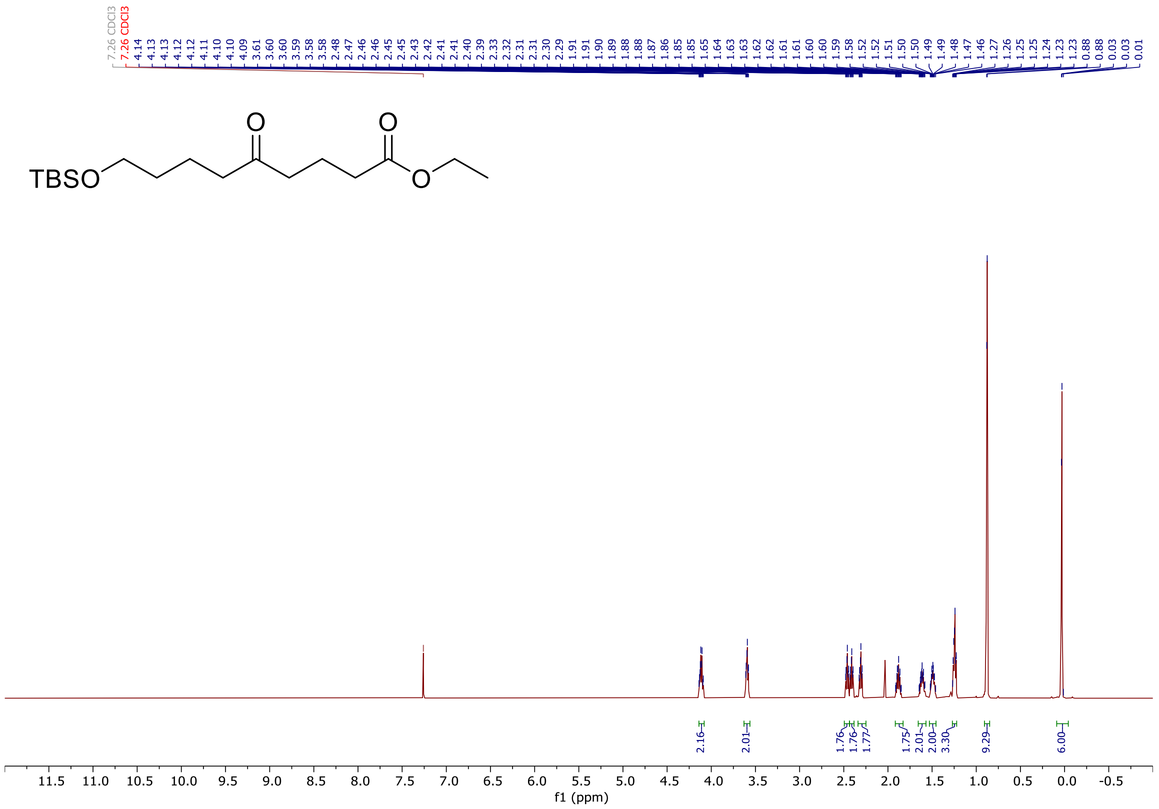

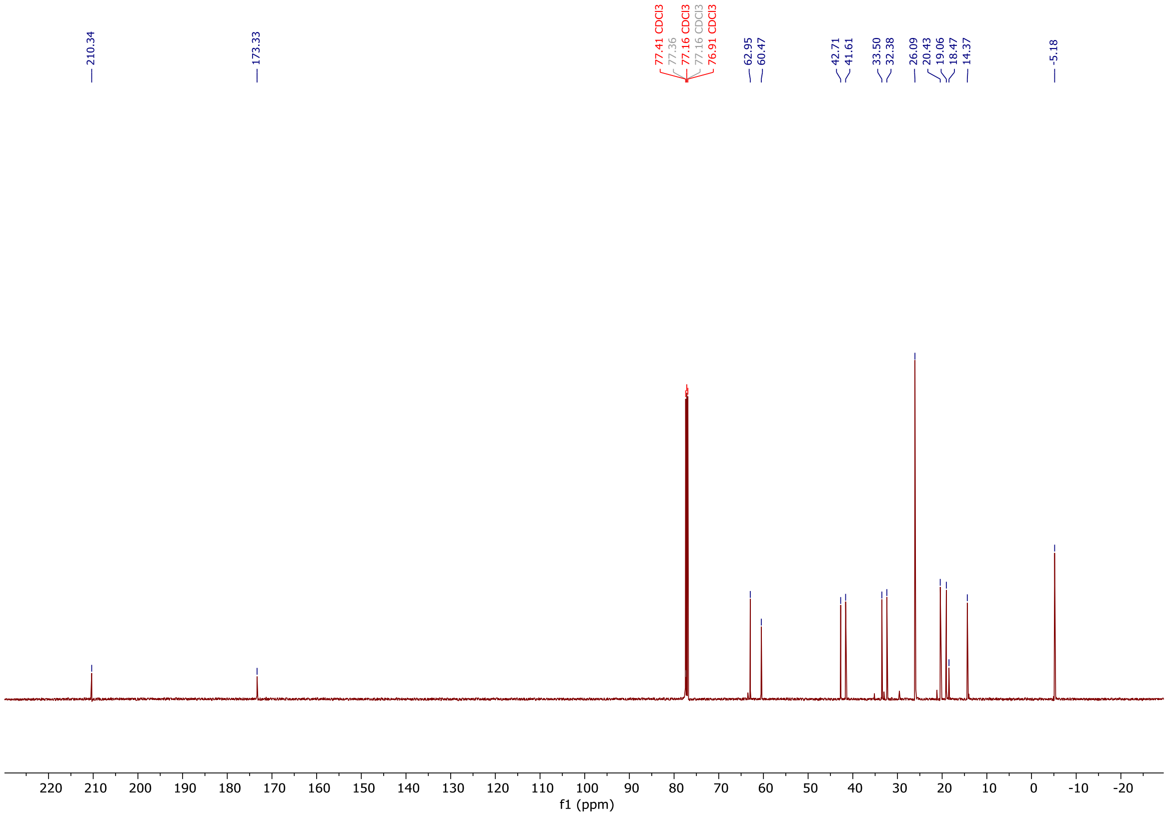

ethyl 9-((*tert*-butyldimethylsilyl)oxy)-5-methylenenonanoate (S2)

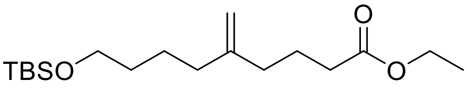

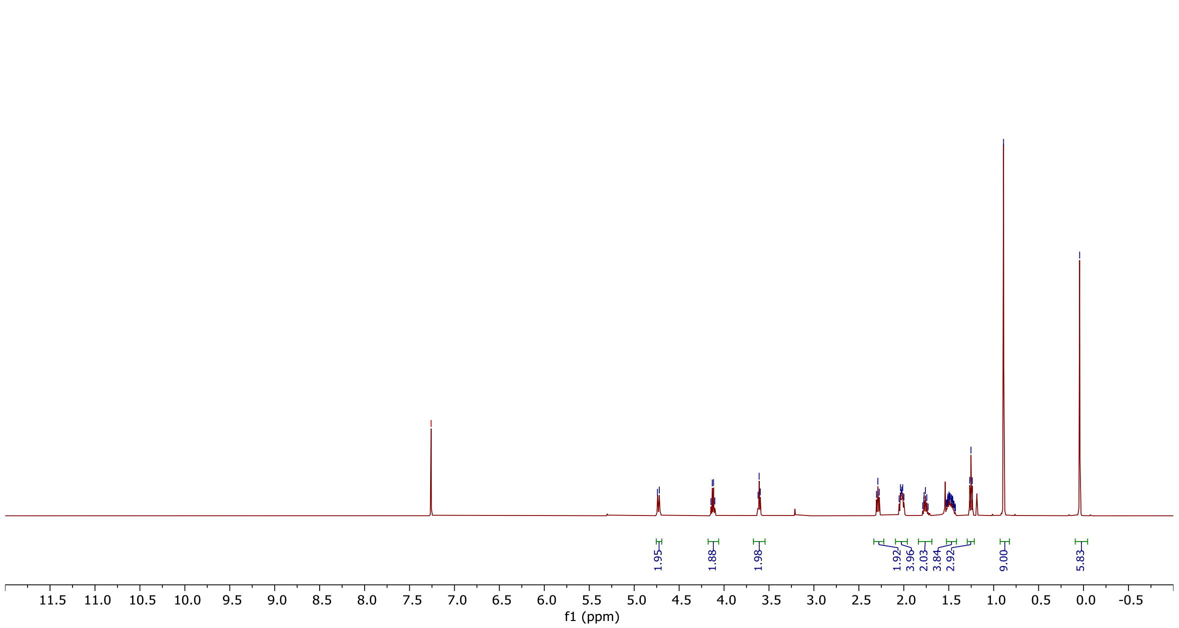

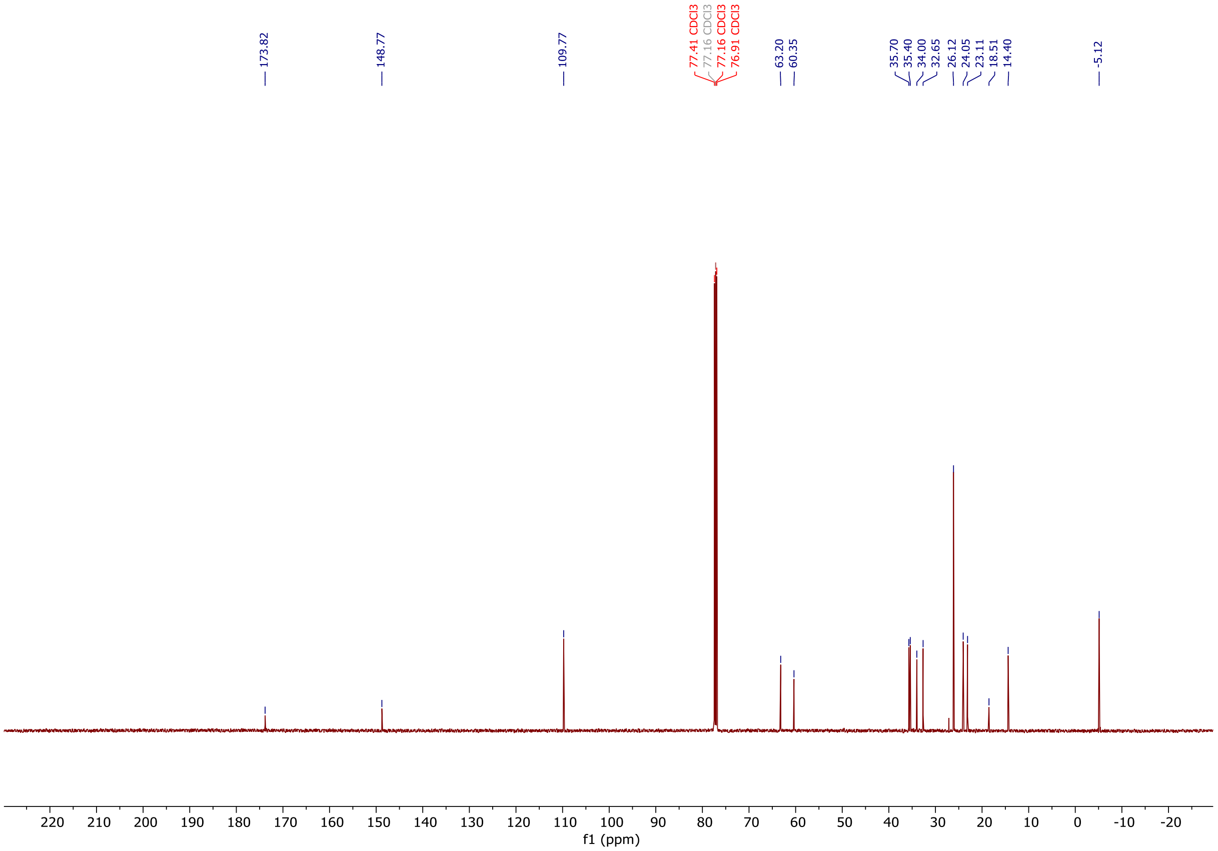

ethyl 9-hydroxy-5-methylenenonanoate (S3)

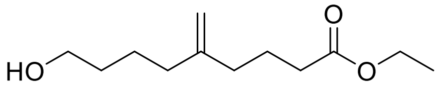

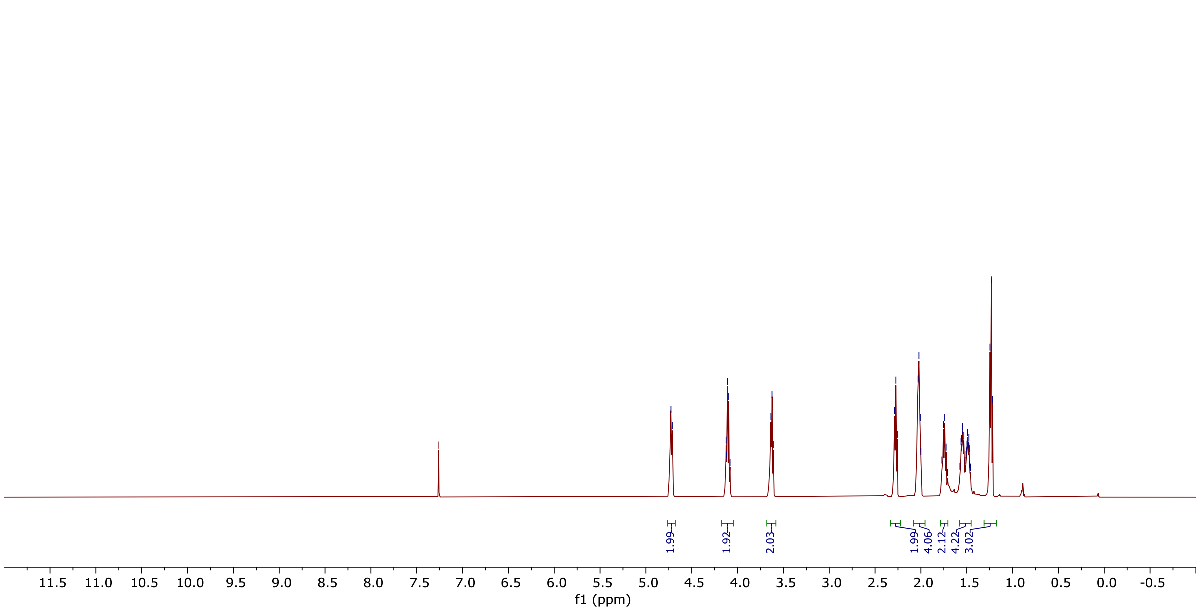

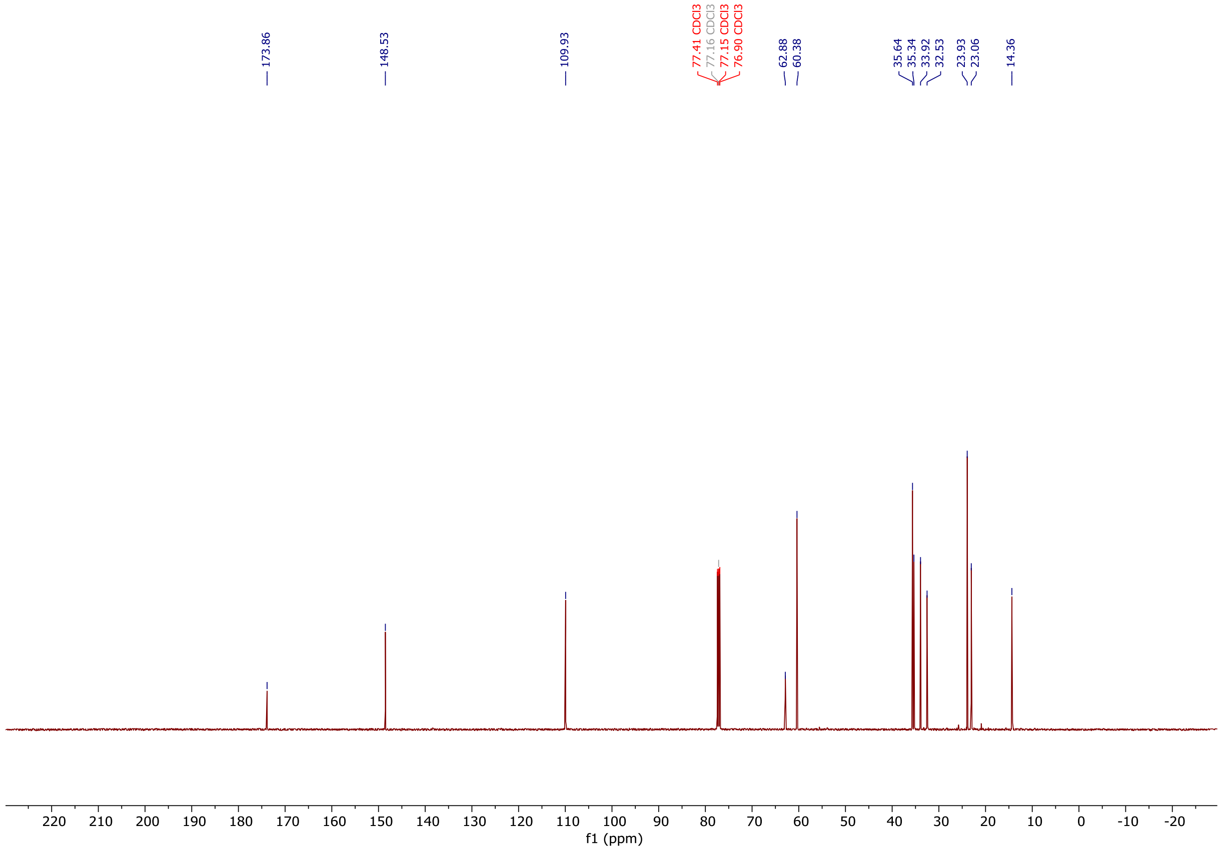

9-((*tert*-butyldimethylsilyl)oxy)-5-methylenenonan-1-ol (S4)

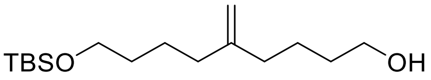

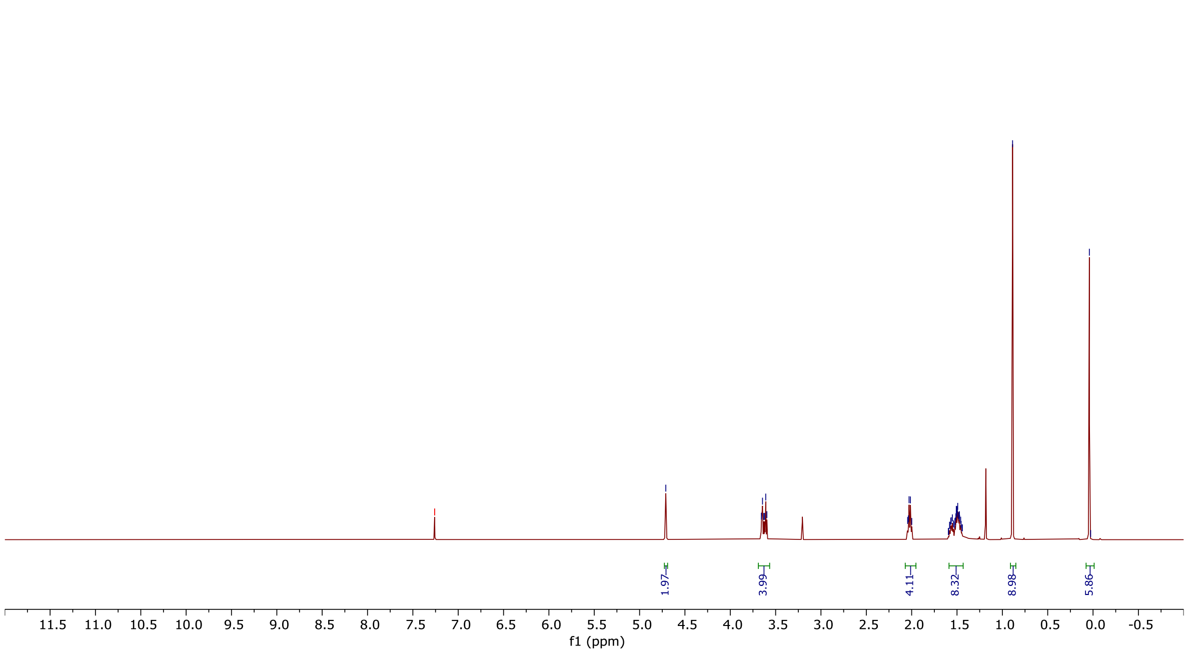

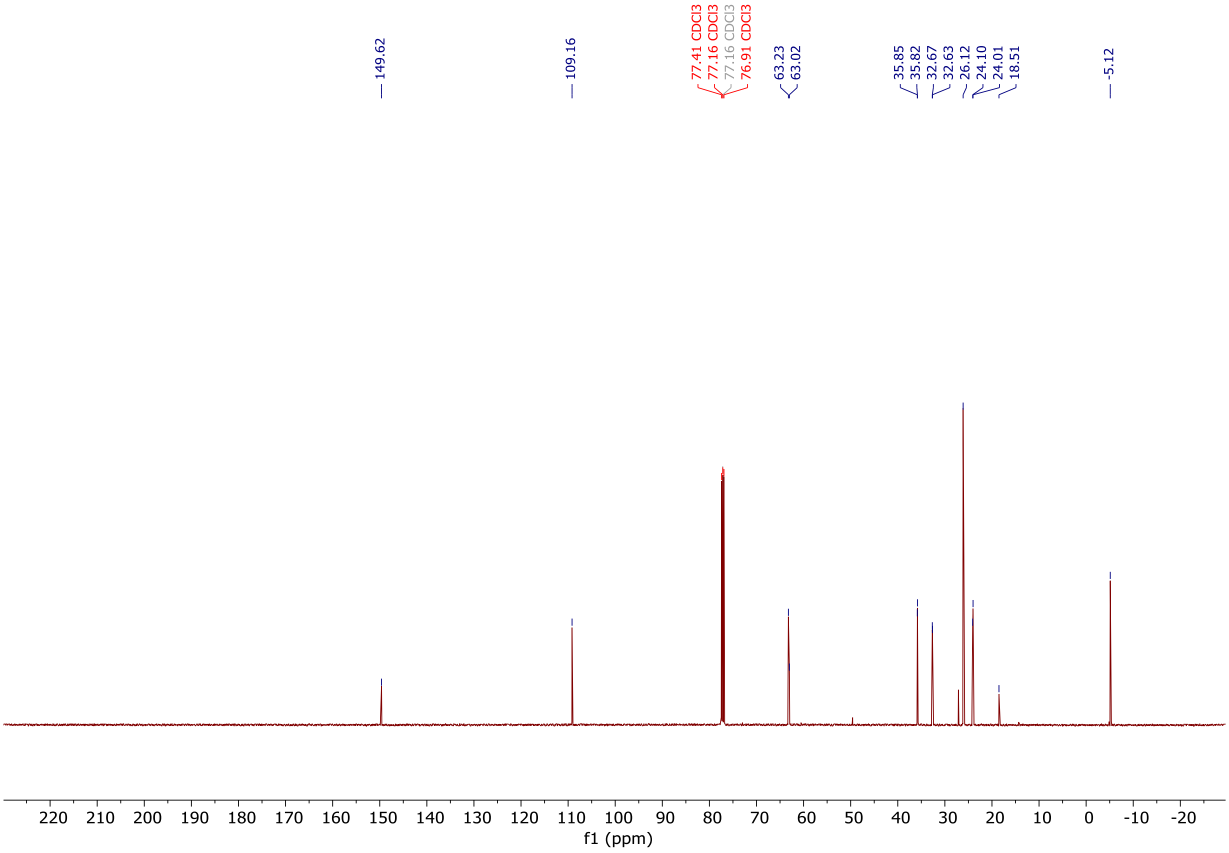

((9-bromo-5-methylenenonyl)oxy)(*tert*-butyl)dimethylsilane (S5)

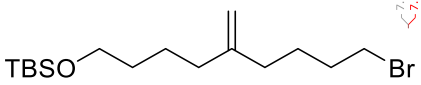

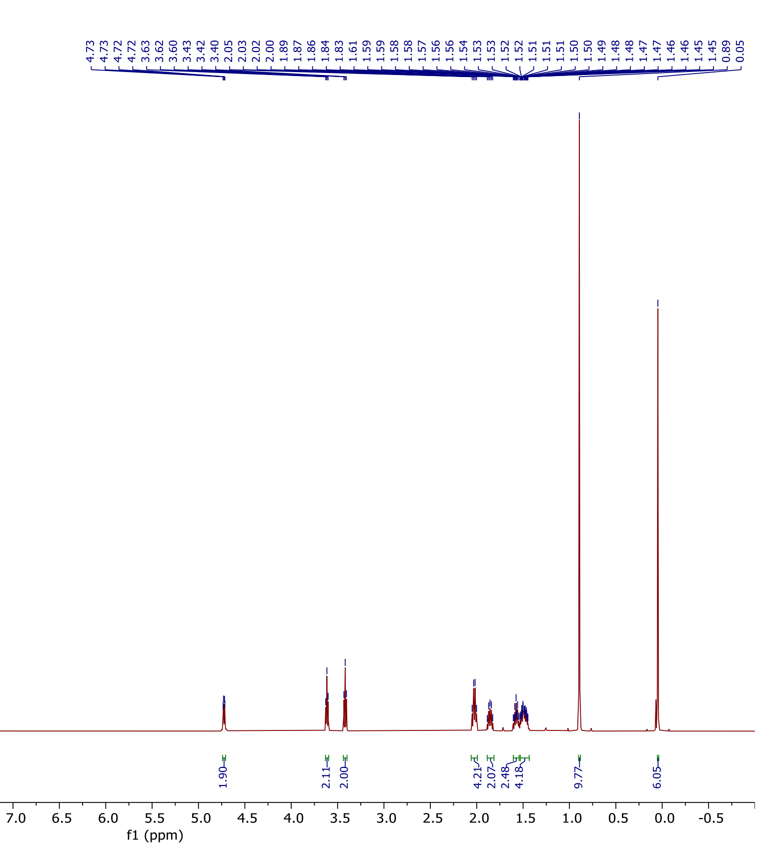

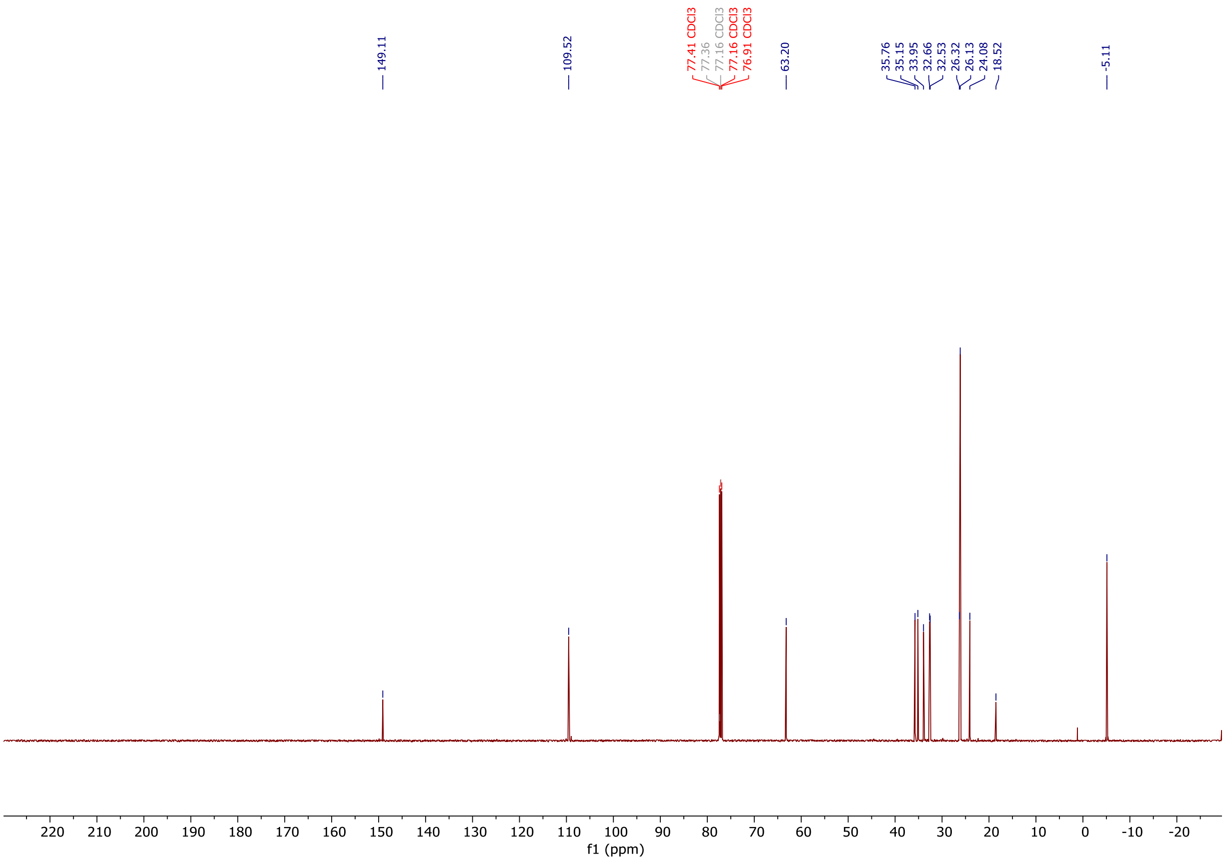

9-hydroxy-5-methylenenonanal (1p)

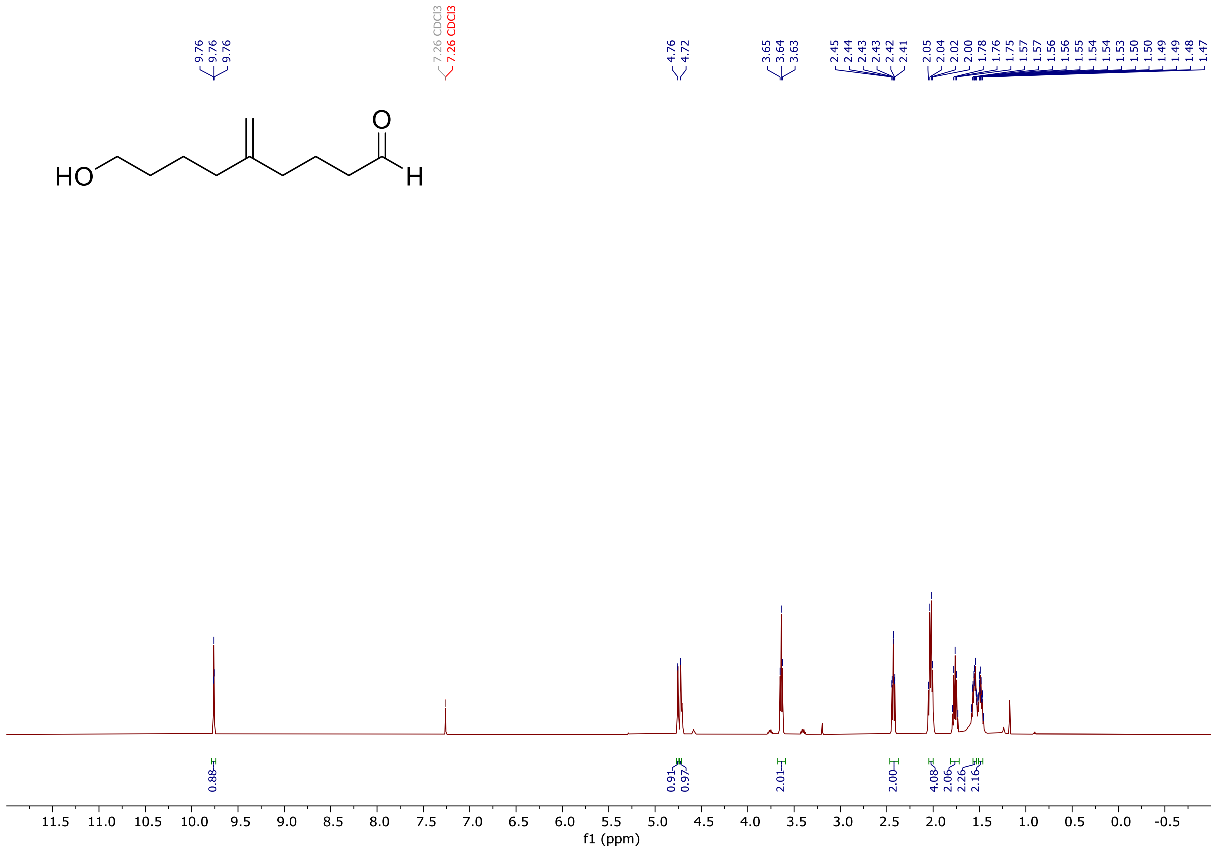

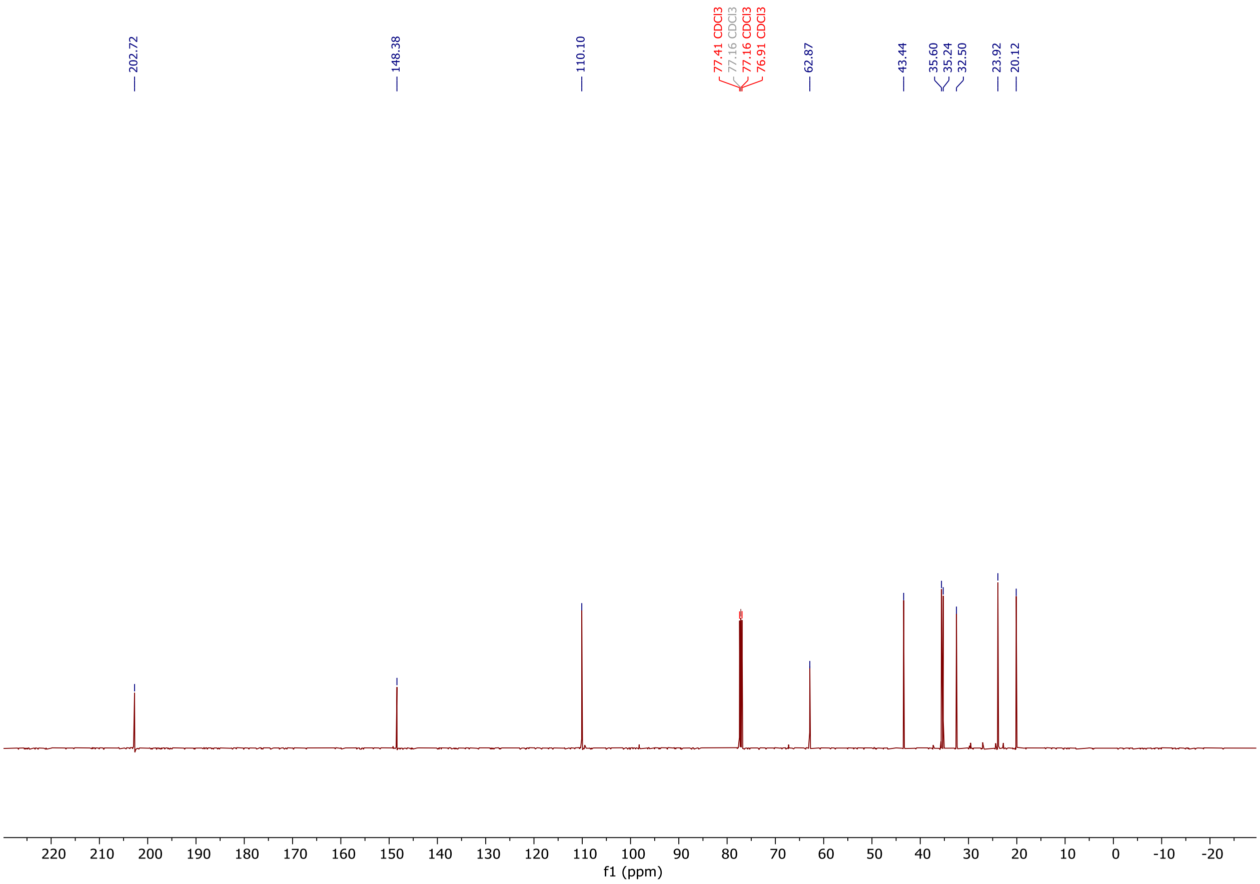

ethyl 5-methylene-9-oxononanoate (1q)

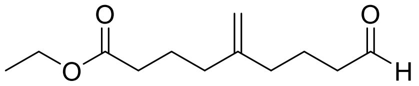

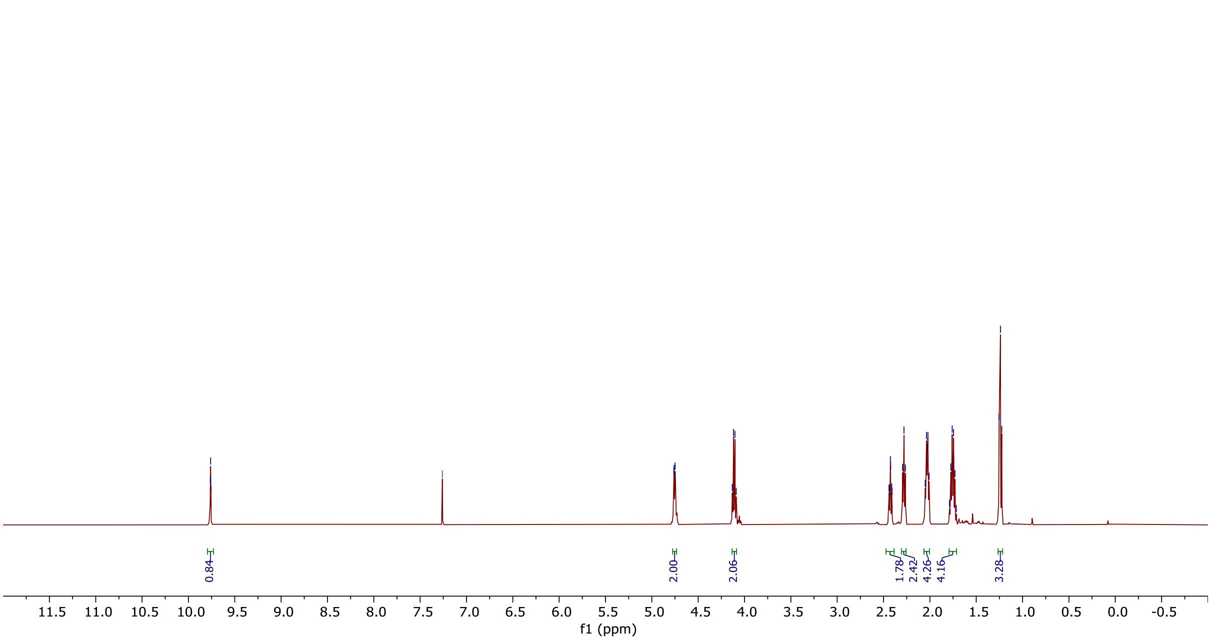

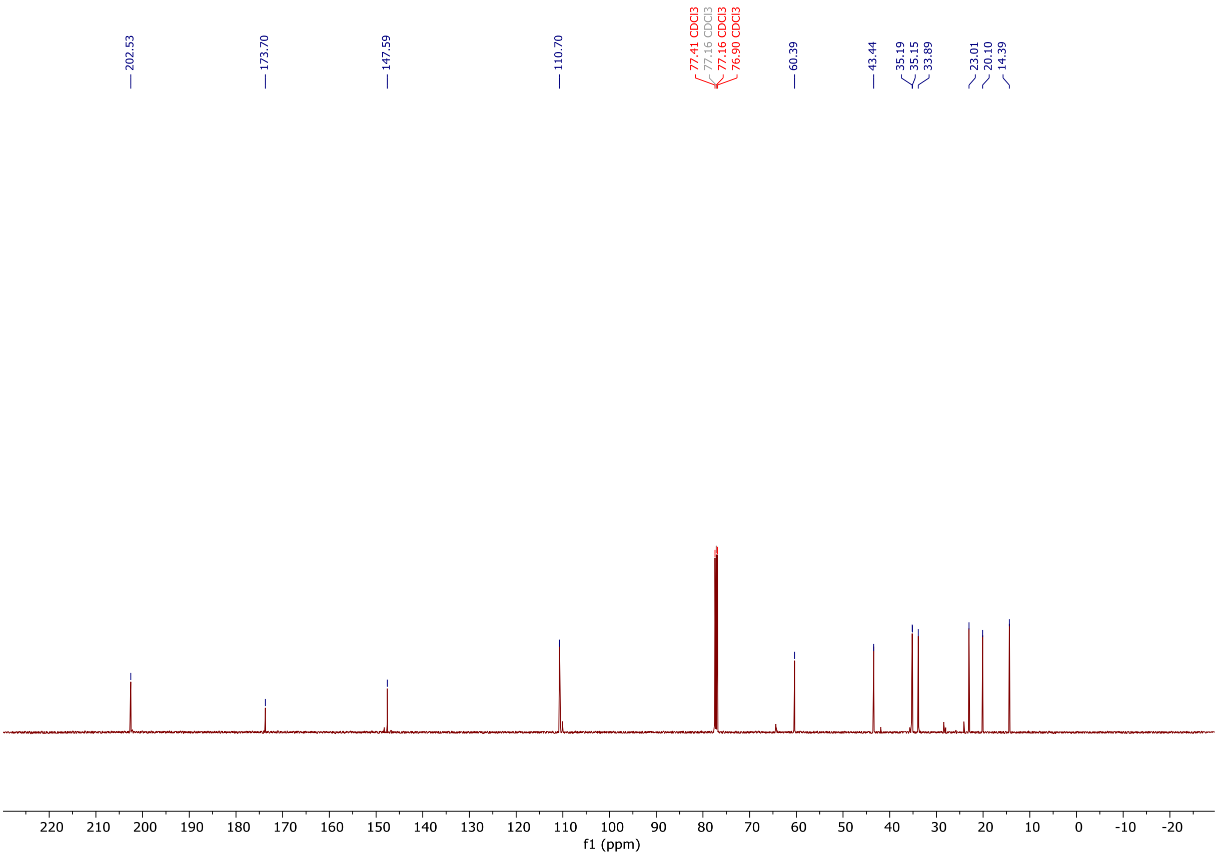

9-bromo-5-methylenenonanal (1r)

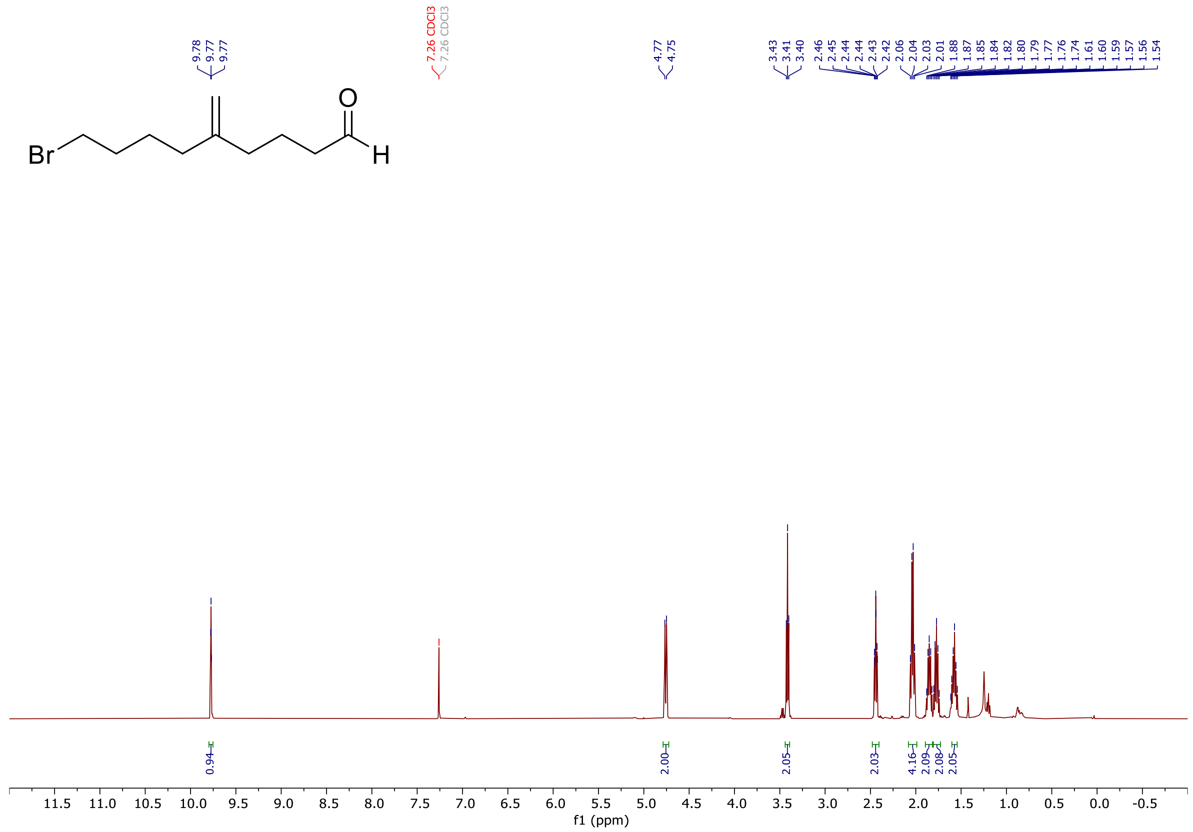

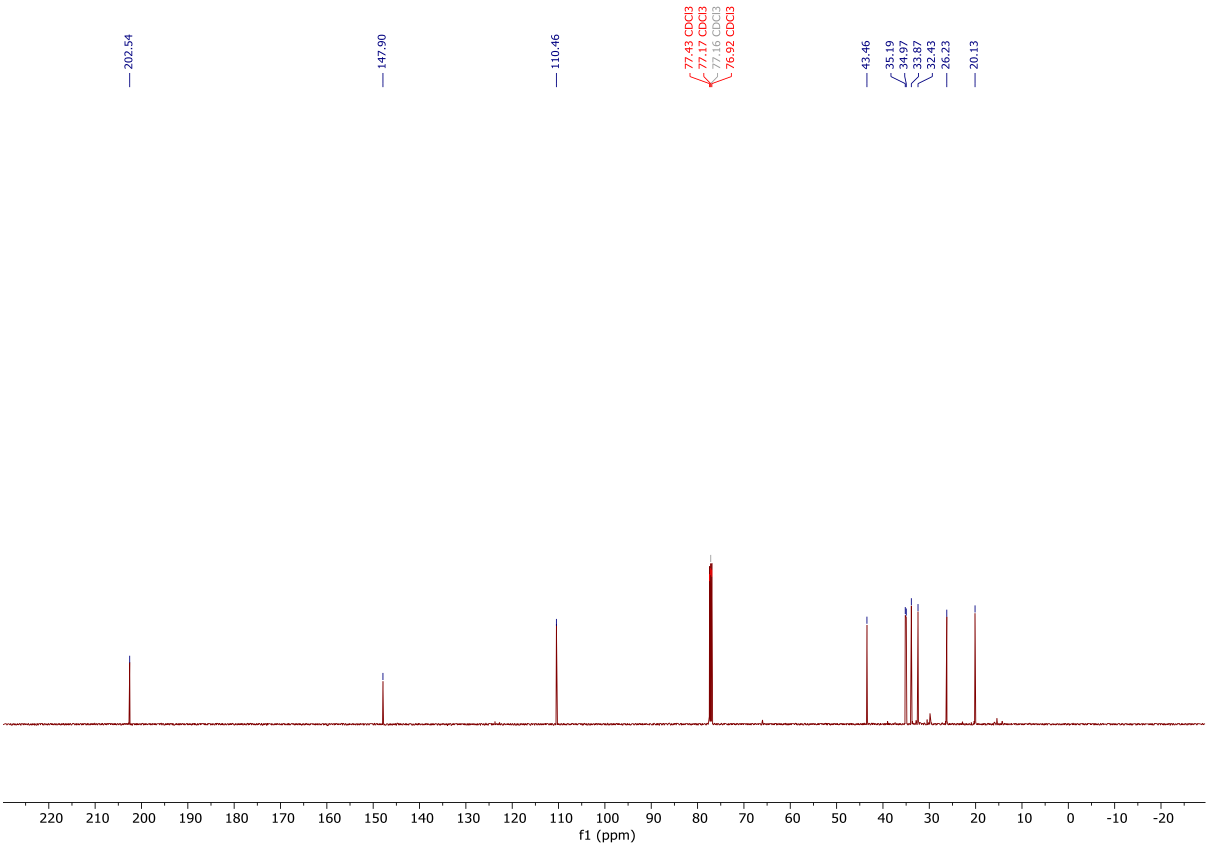

6-methylhept-6-enal (1s)

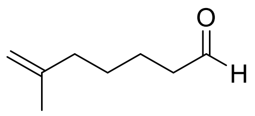

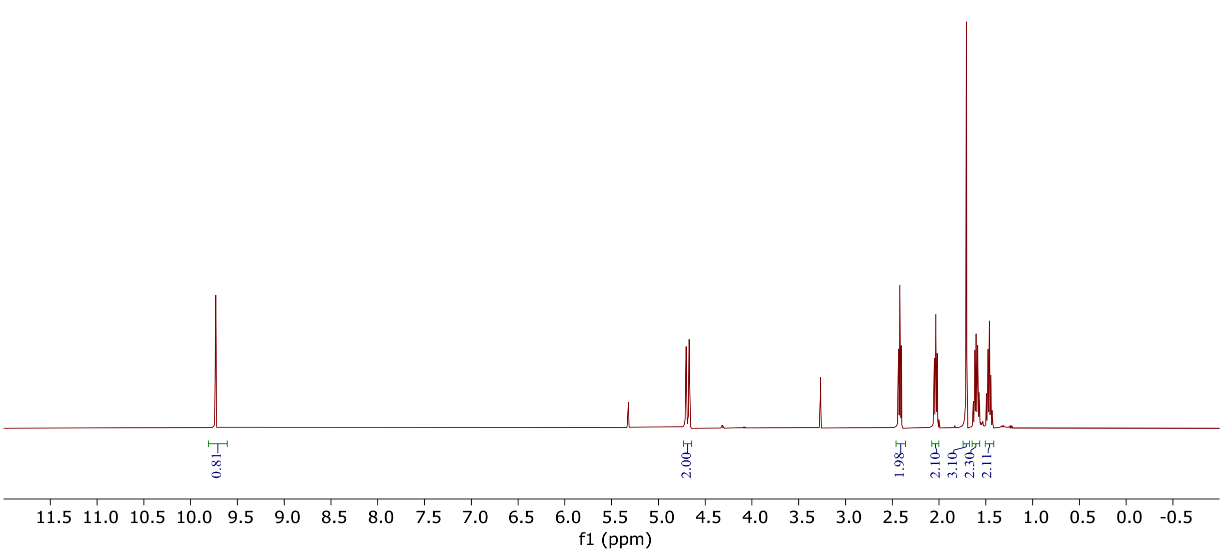

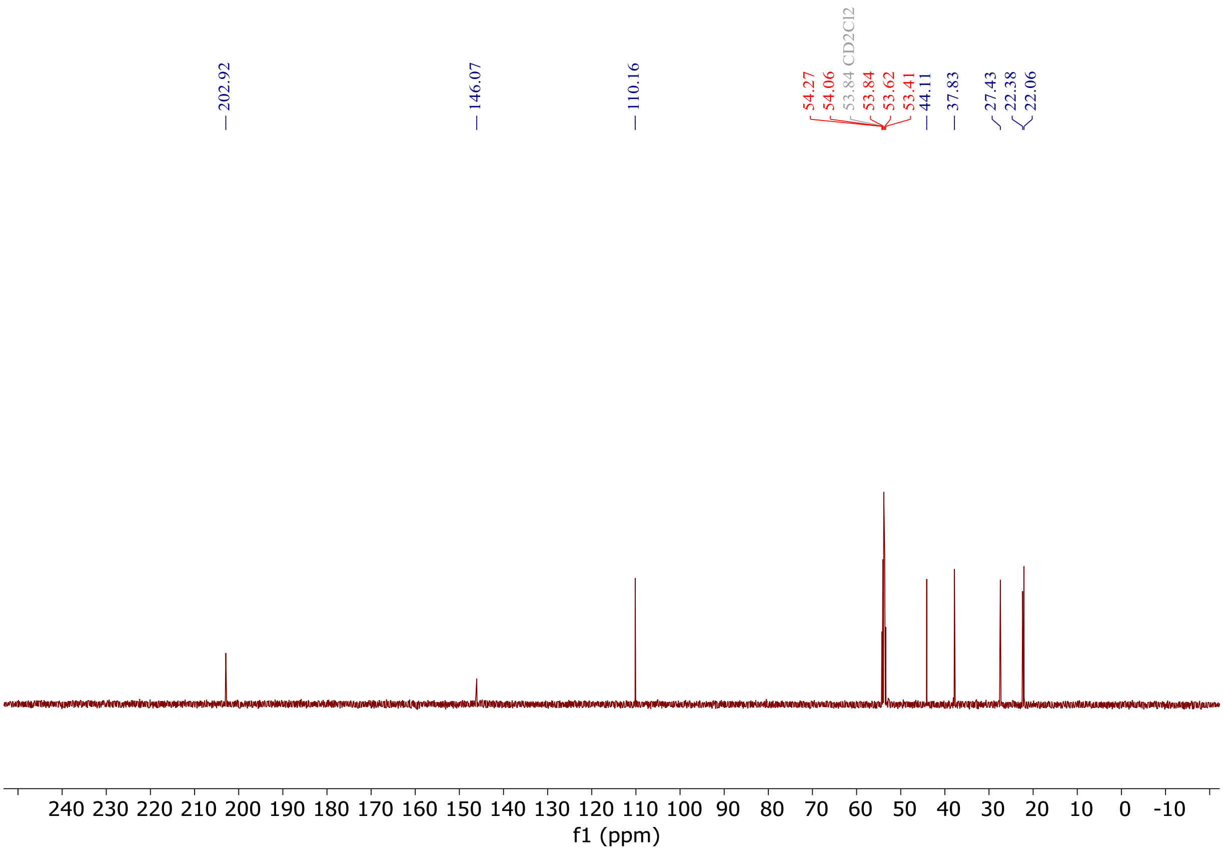

6-methylenedecanal (1t)

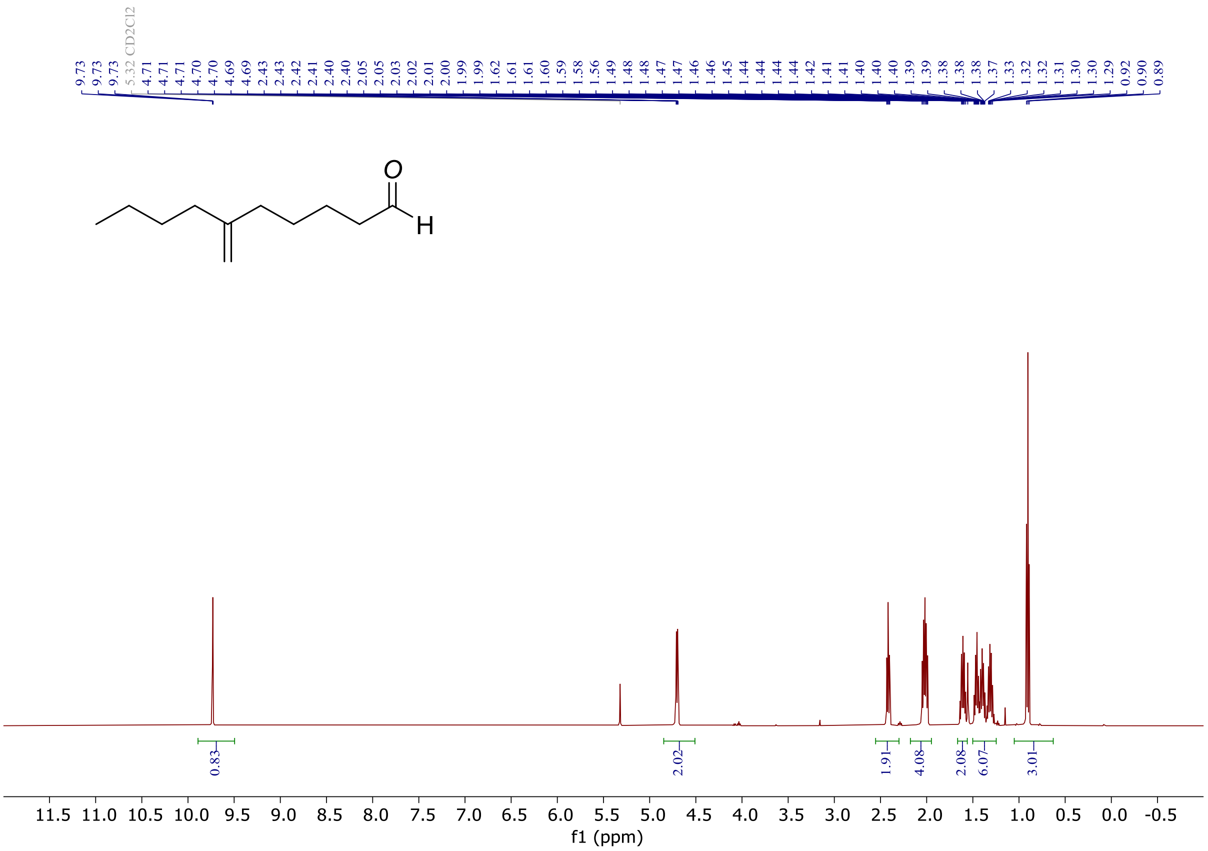

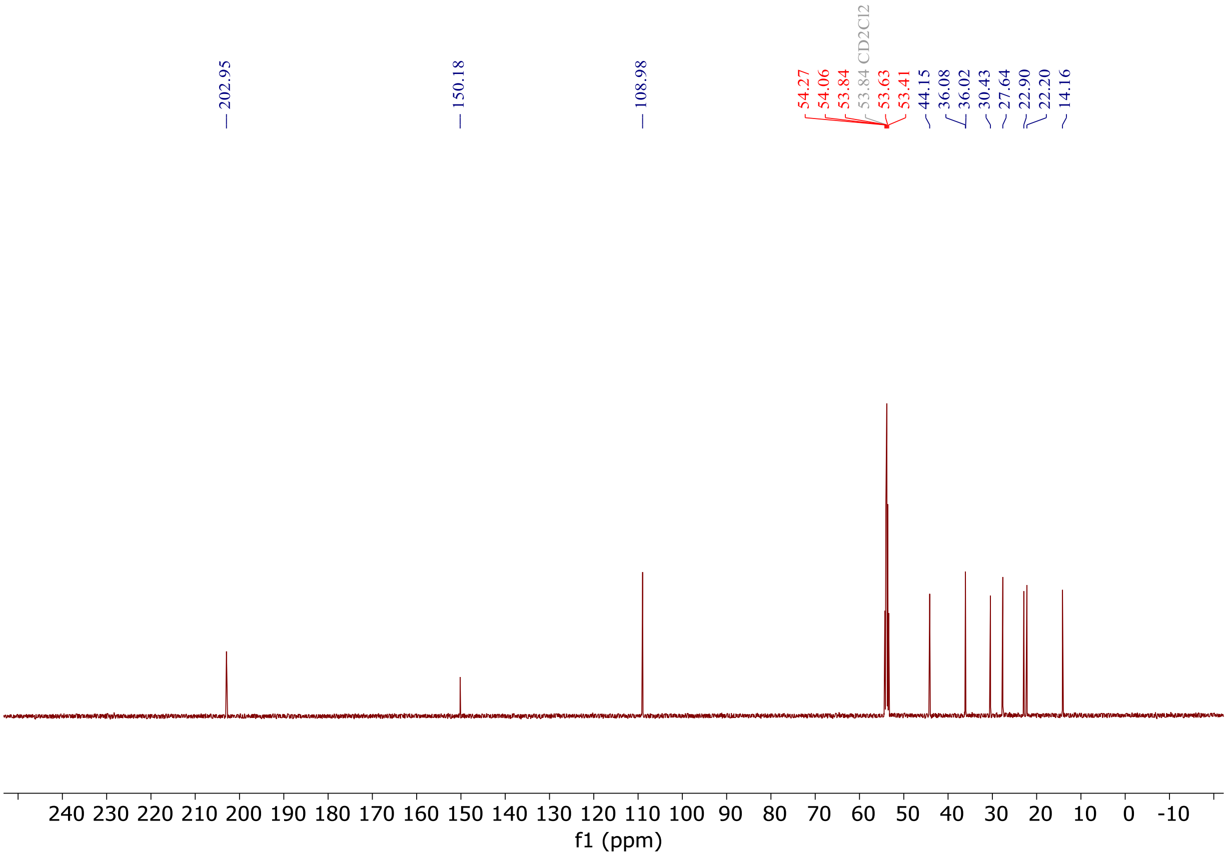

3,3,5-trimethylhex-5-enal (1u)

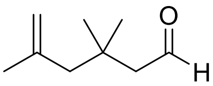

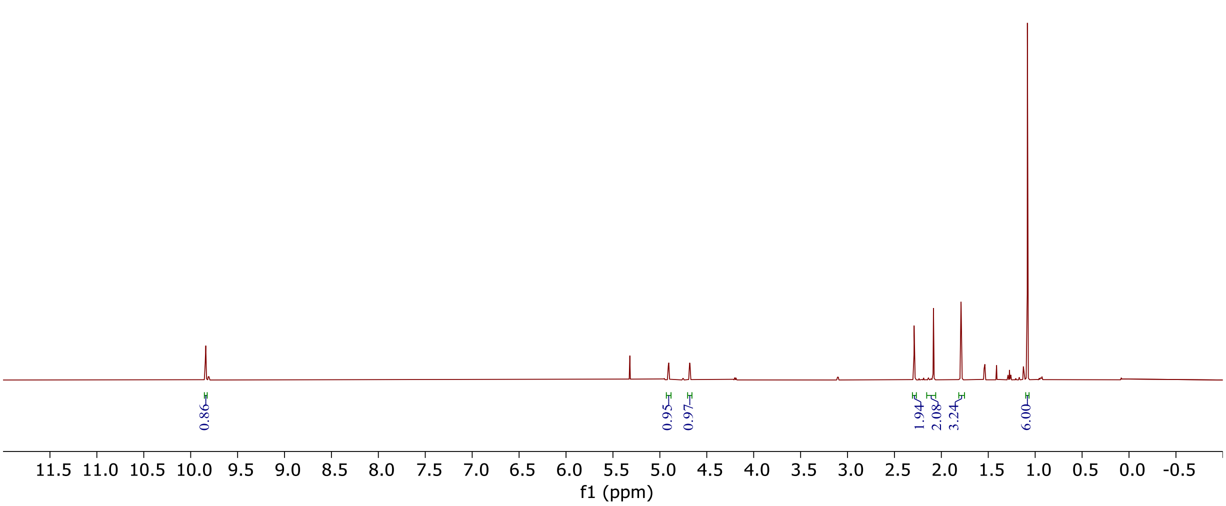

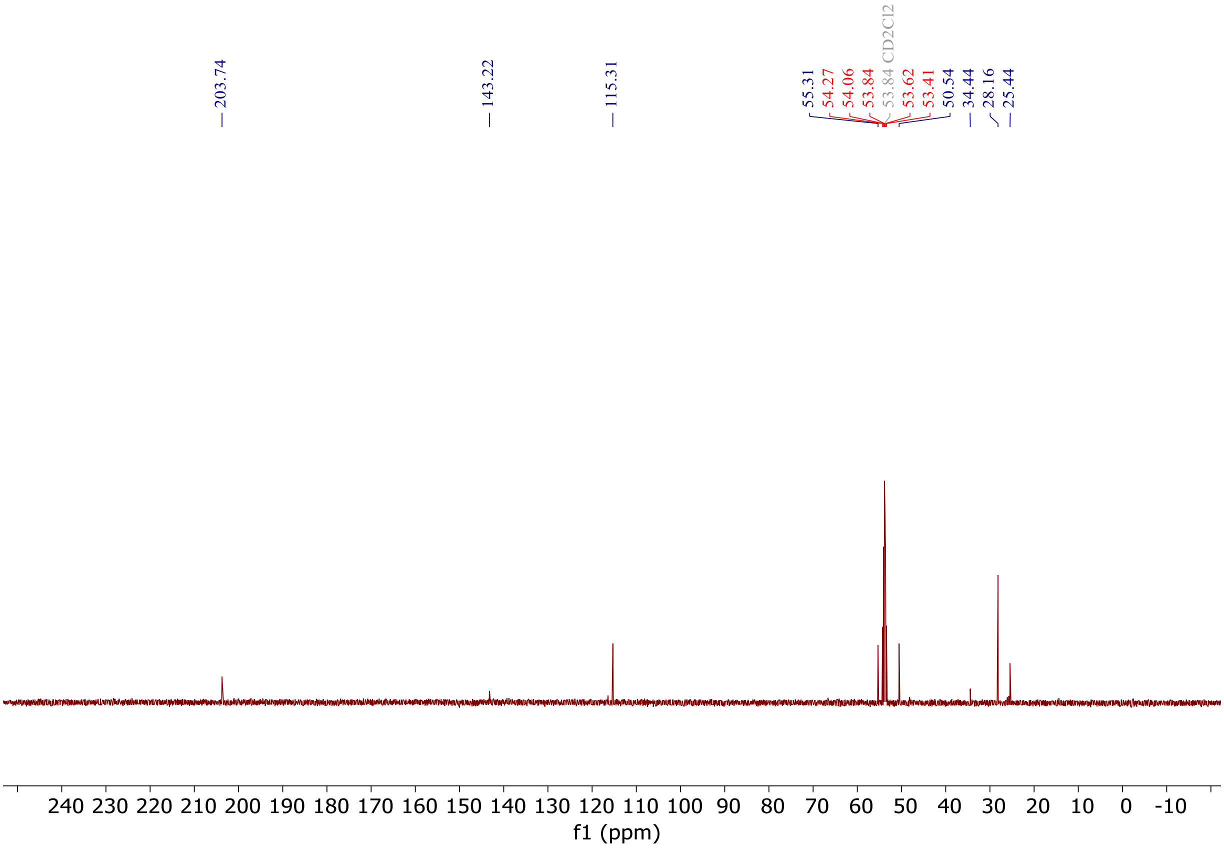

2-(2-methylallyl)benzaldehyde (1v)

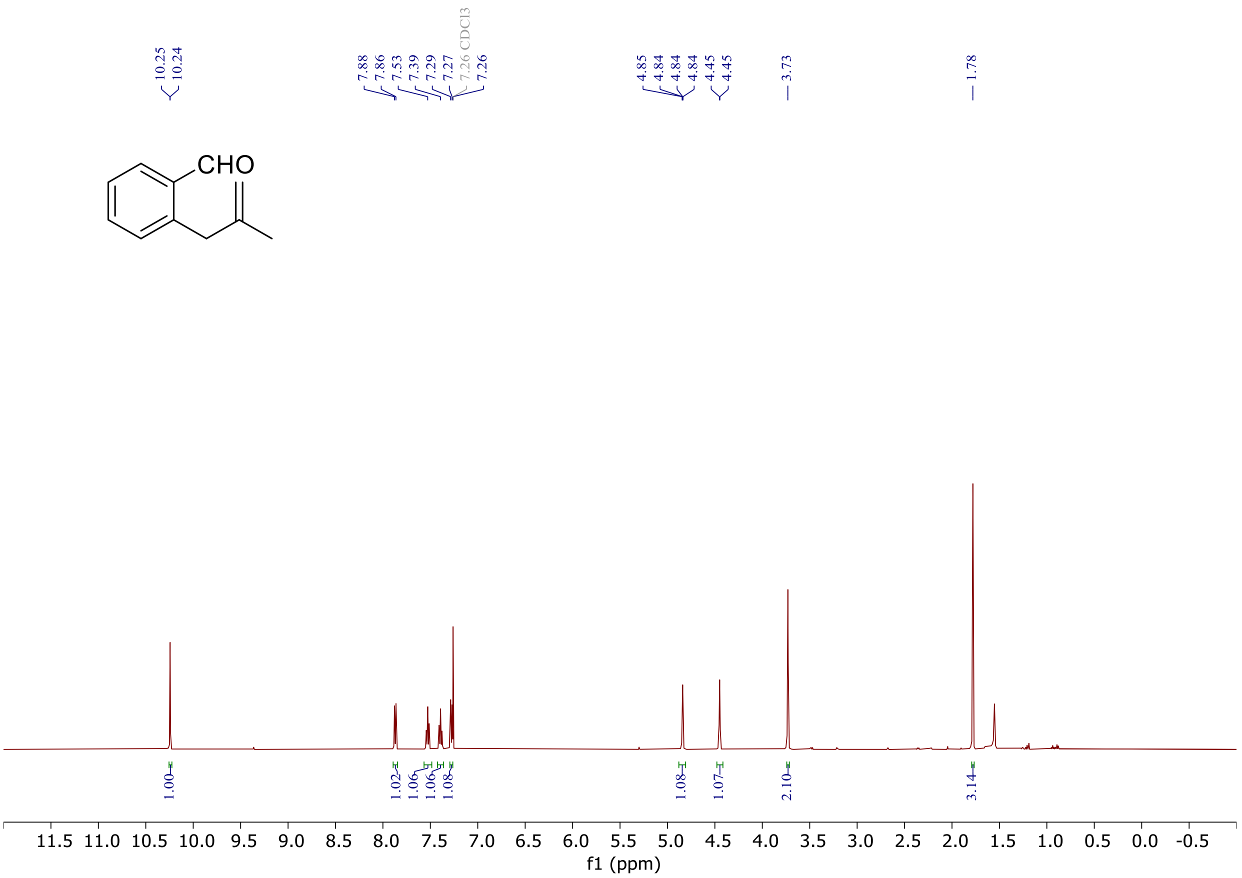

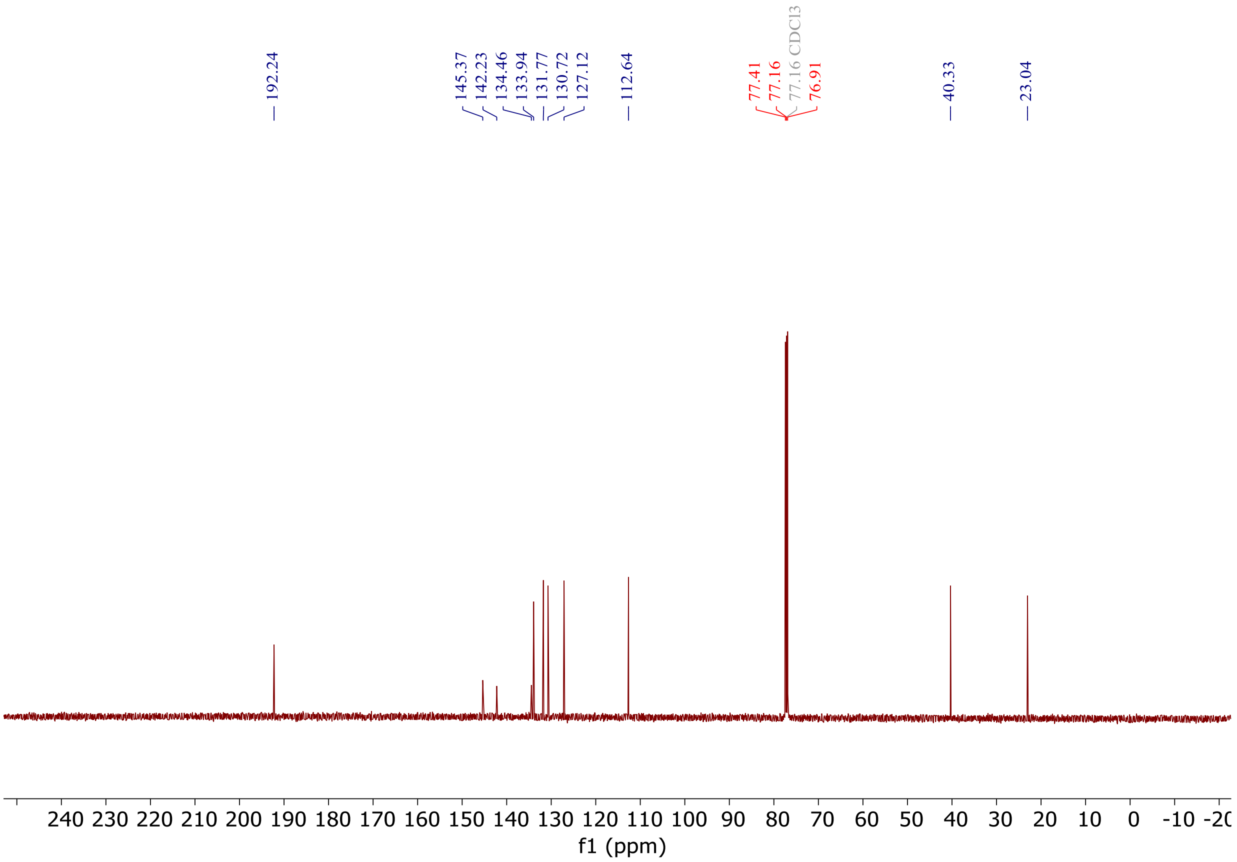

5-methylhex-5-enal-1-*d*

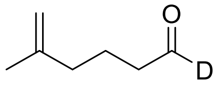

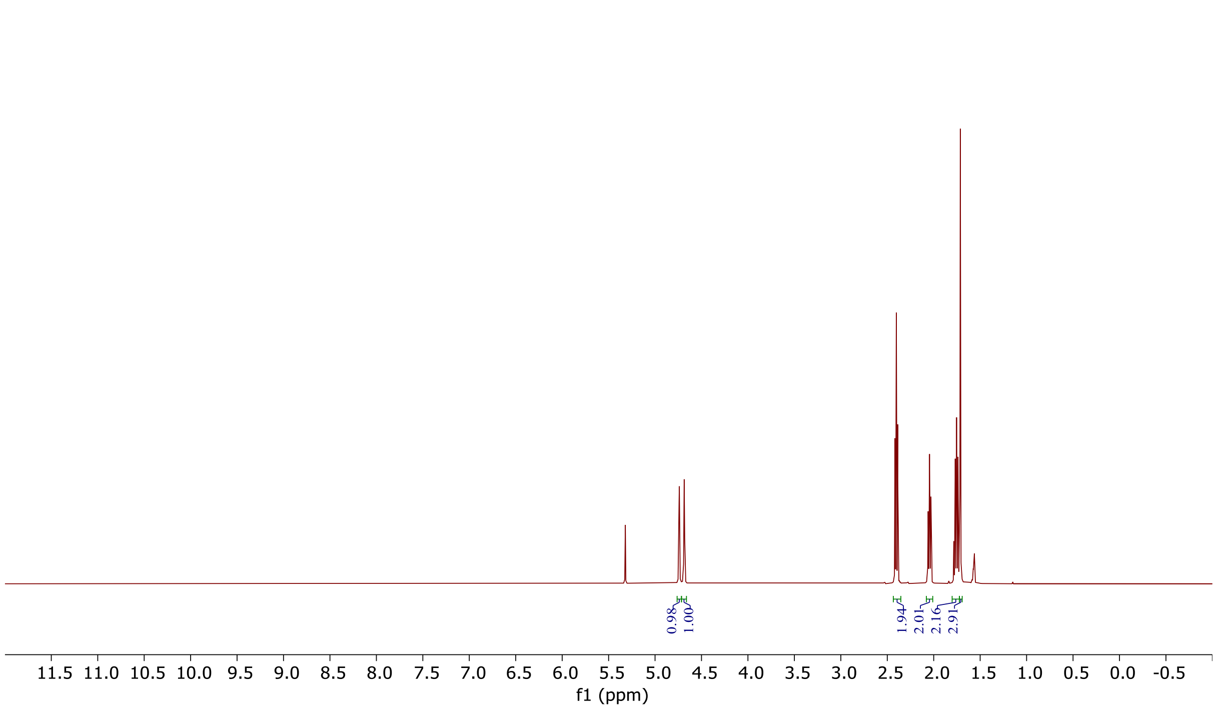

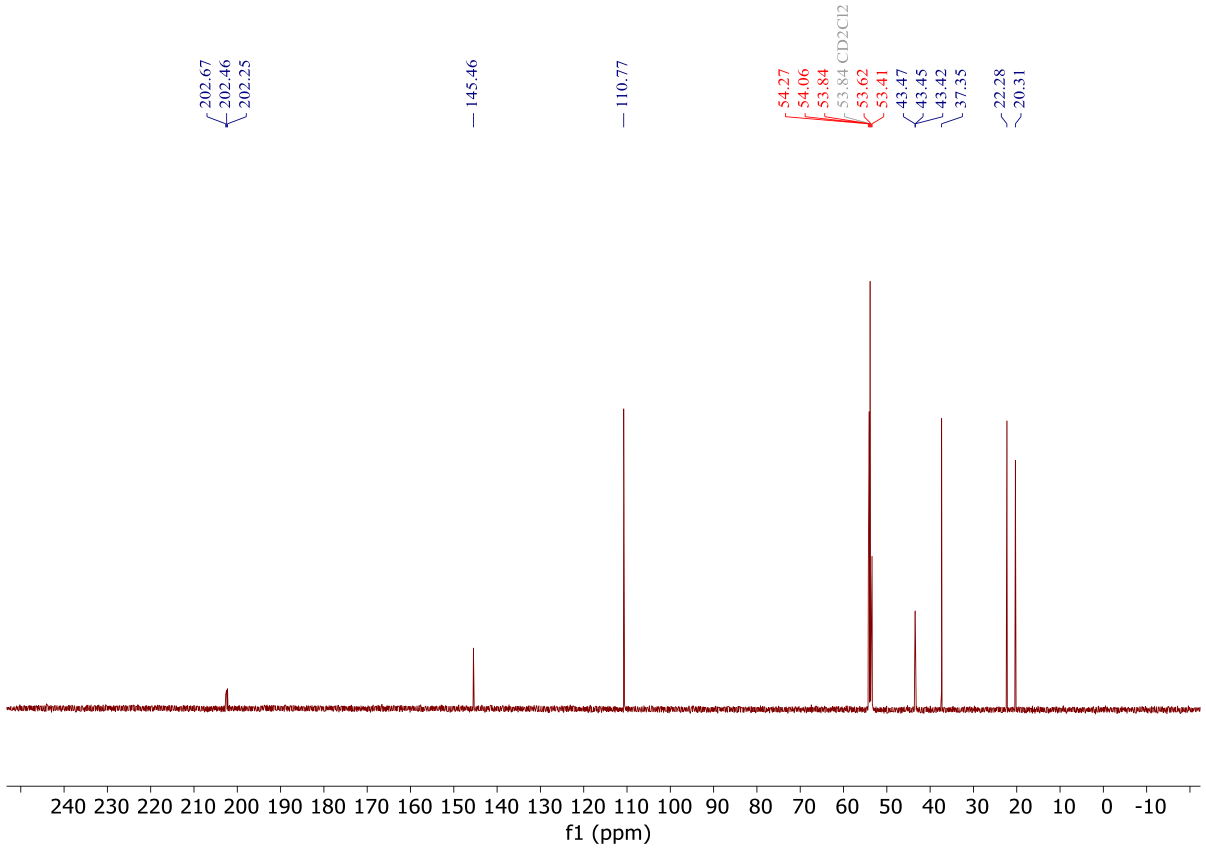

(*S*)-3-methylenecyclohexan-1-ol (2a)

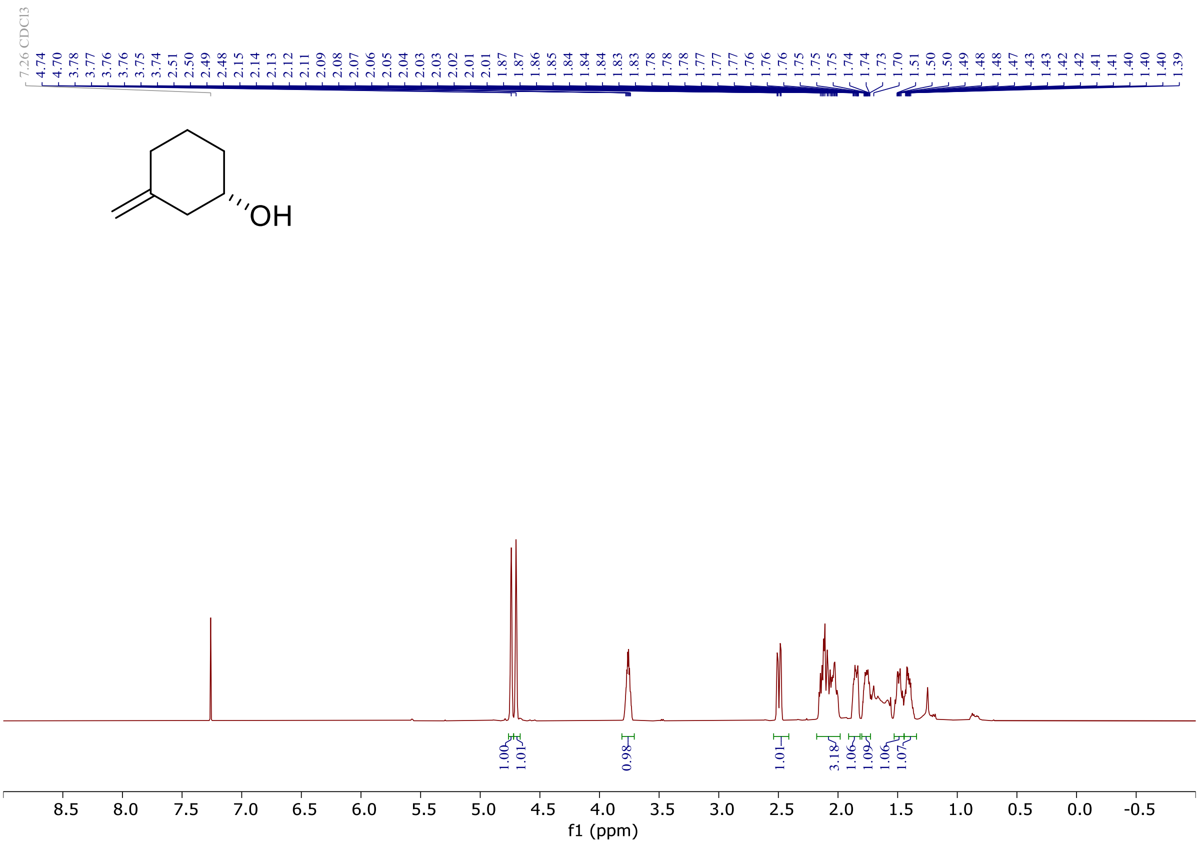

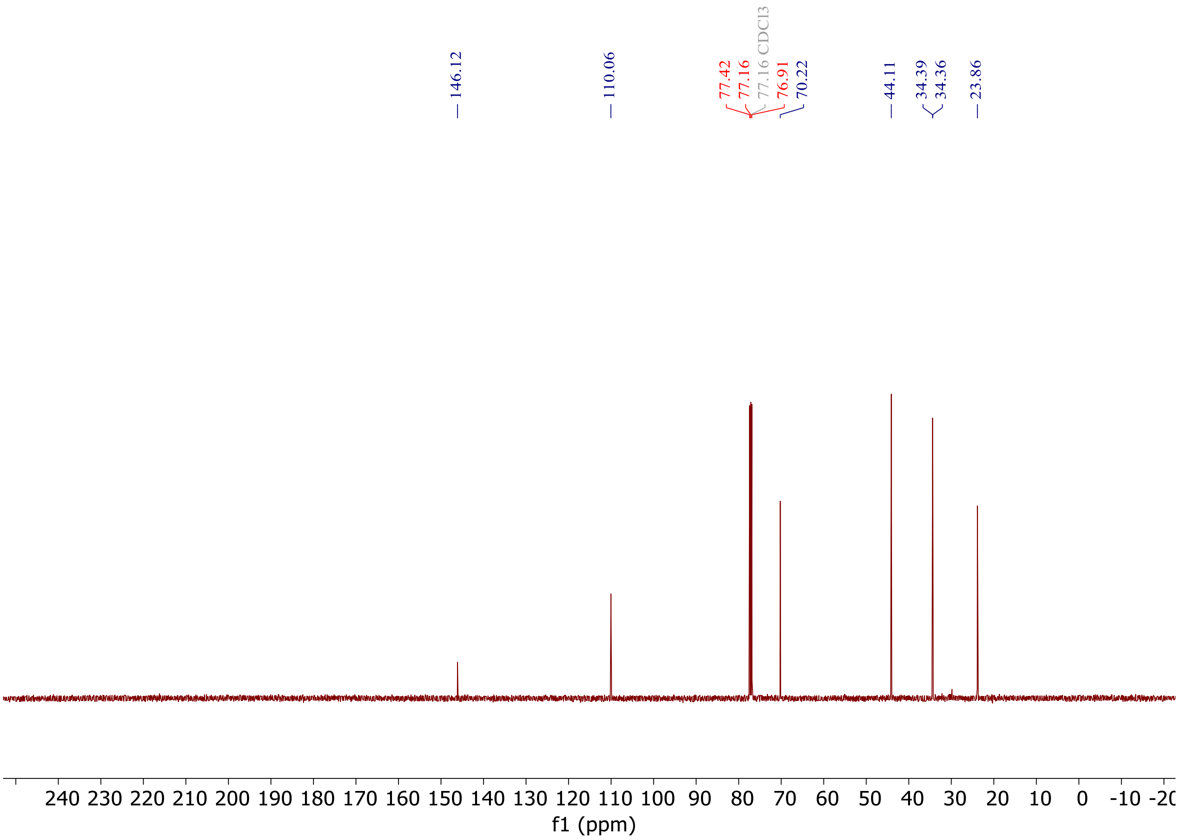

(*S*,*E*)-3-propylidenecyclohexan-1-ol (2b)

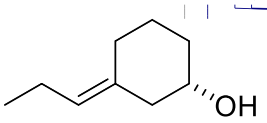

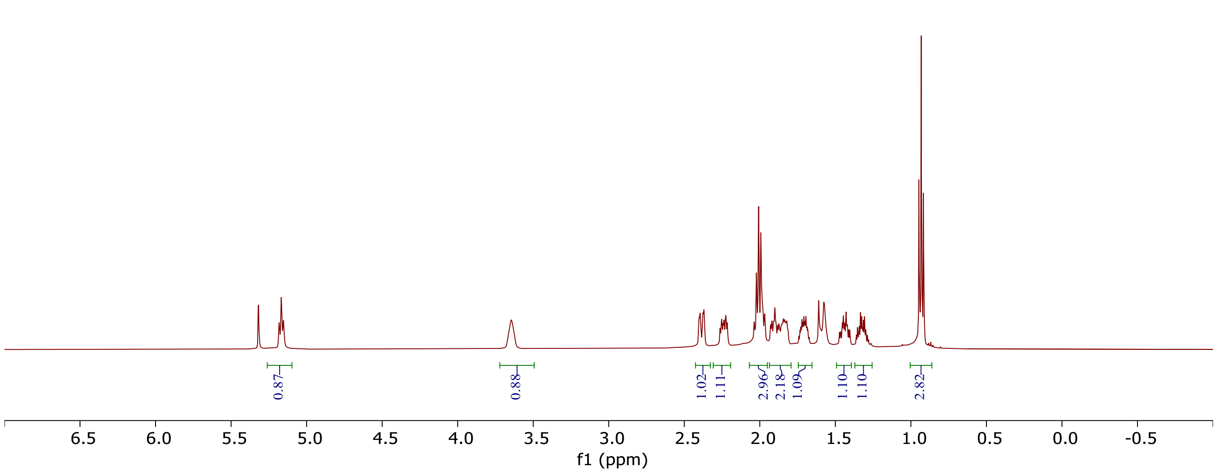

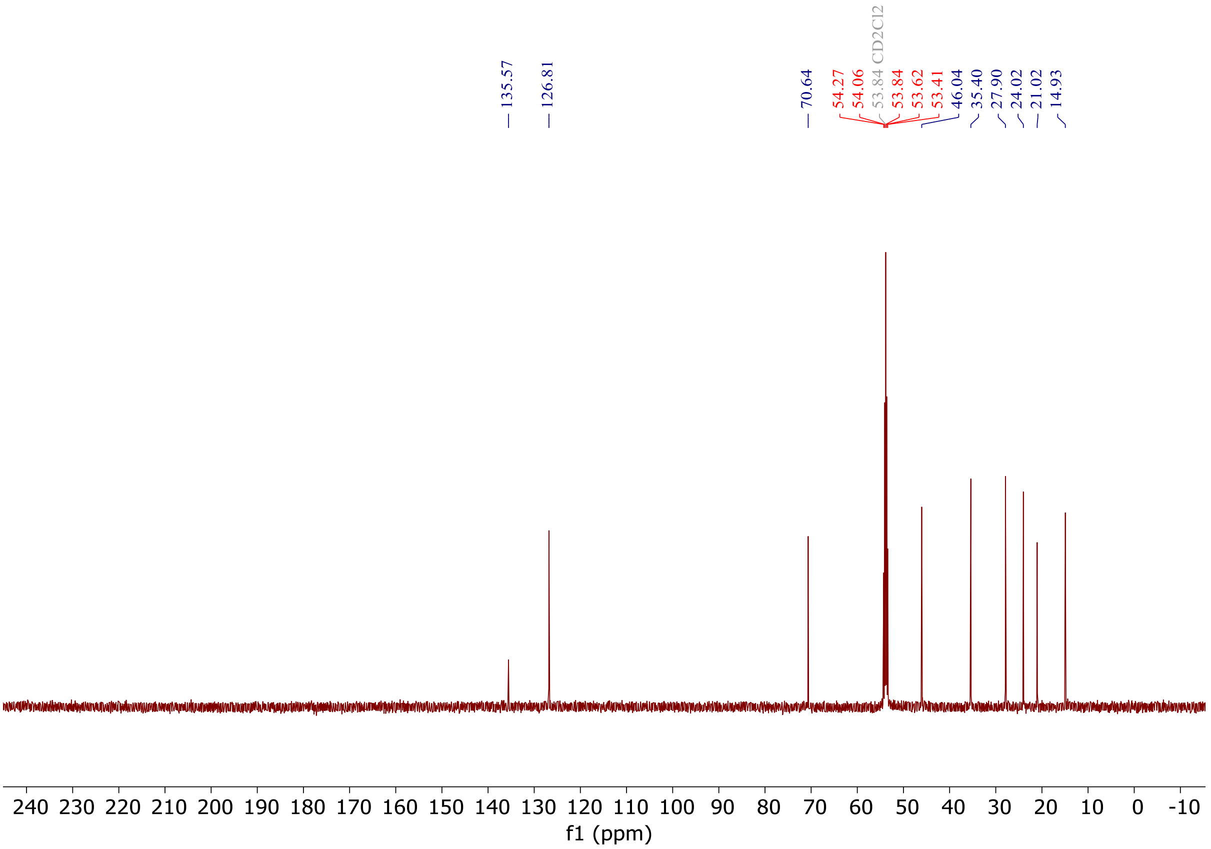

(*S*,*E*)-3-heptylidenecyclohexan-1-ol (2c)

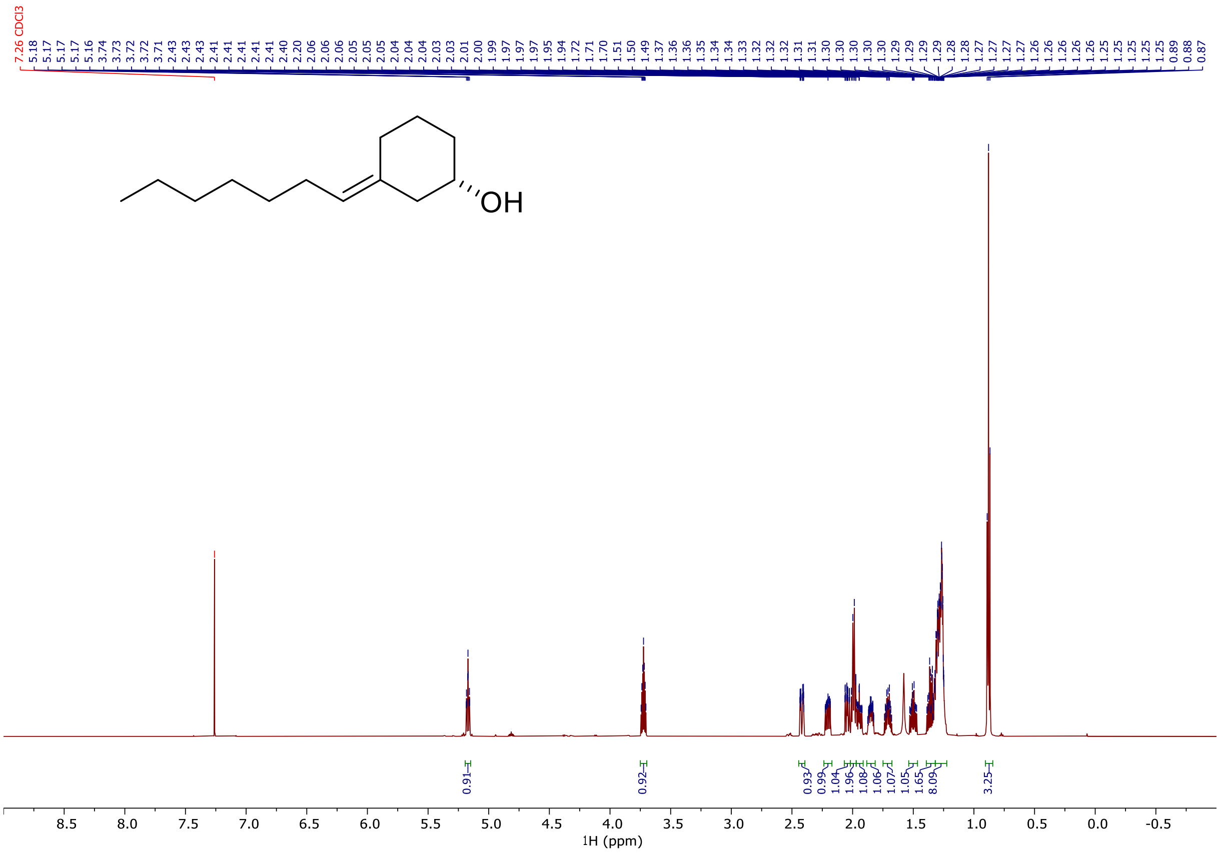

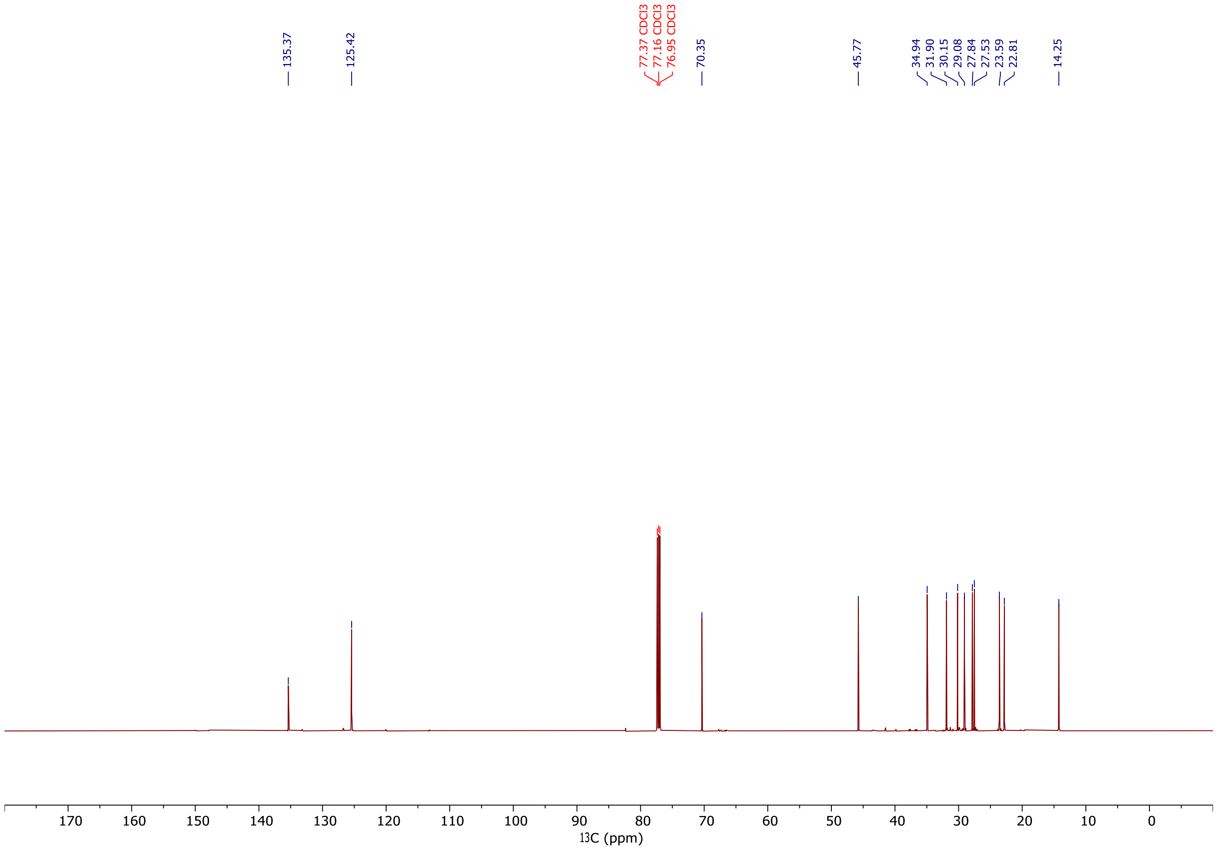

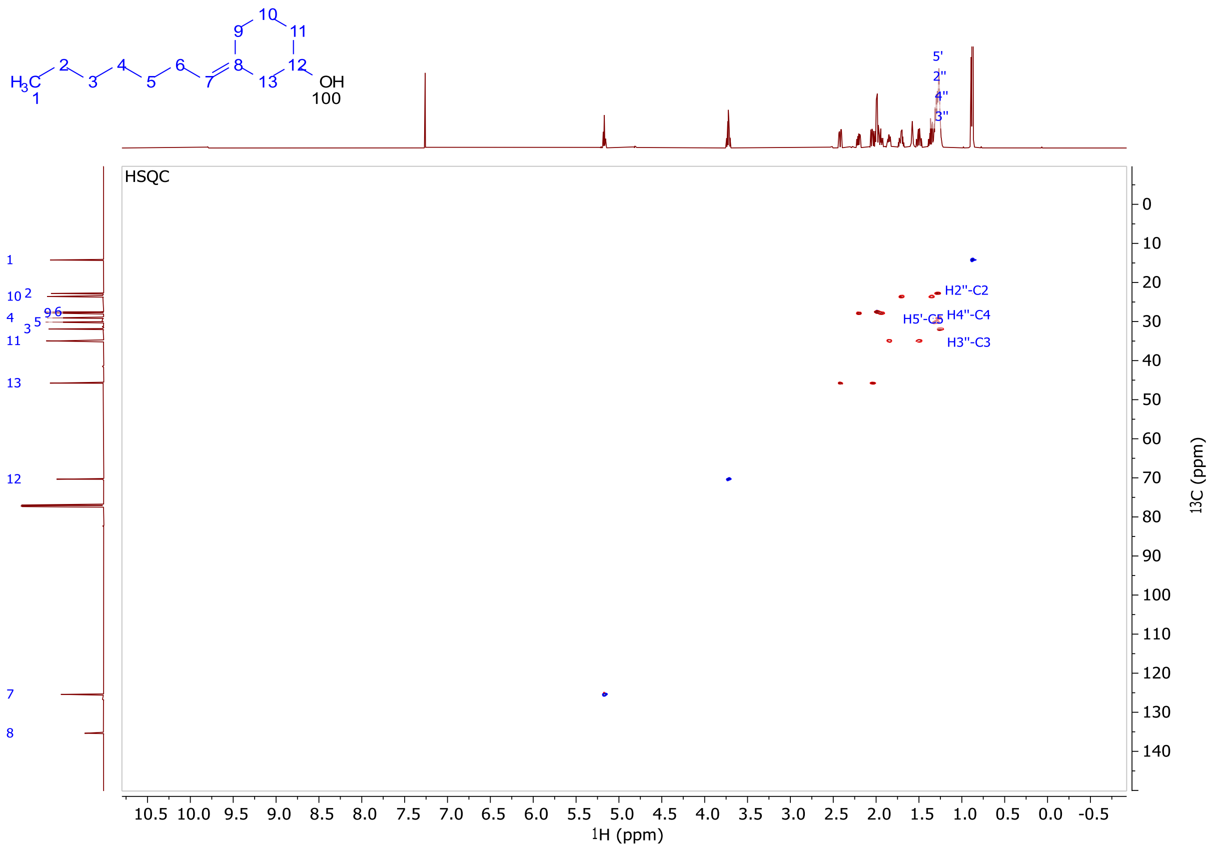
HSQC
H2''-C2
H5'-C5
H4''-C4
H3''-C3
1H (ppm)
13C (ppm)
OH
100

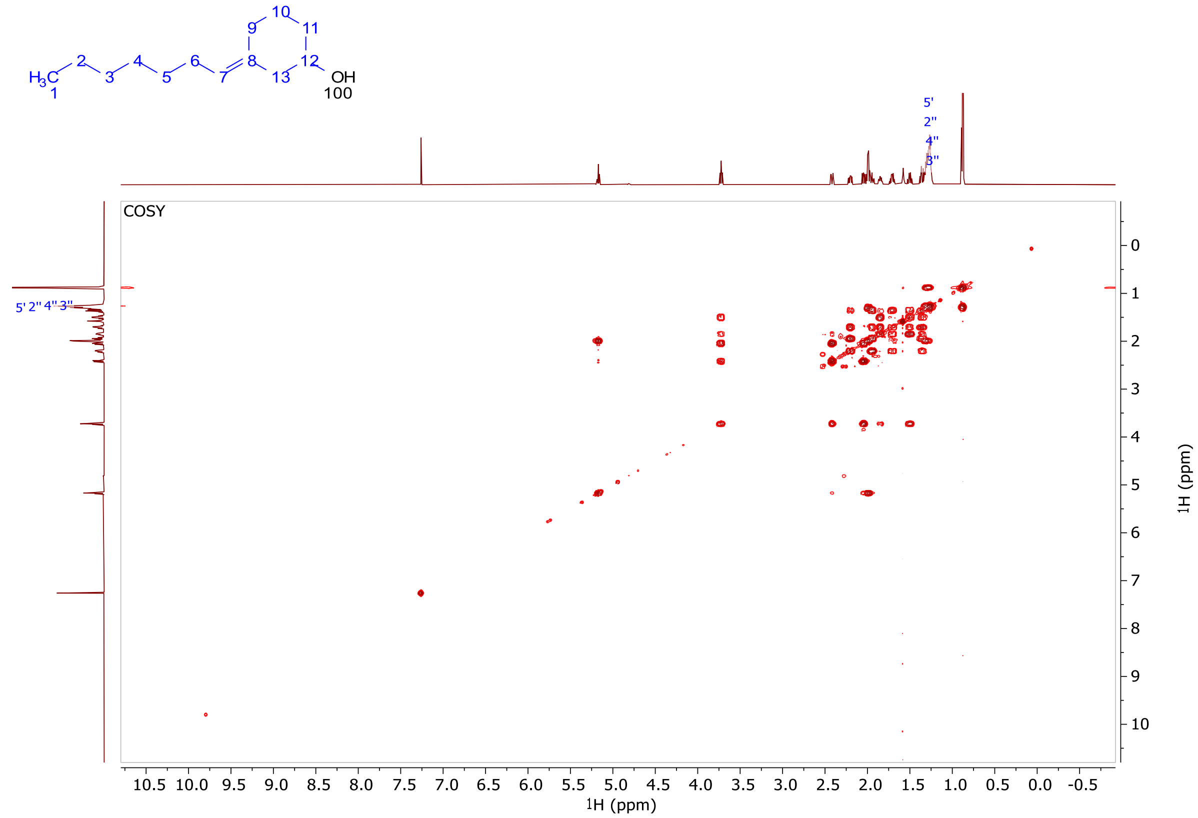
COSY
1H (ppm)
1H (ppm)
OH
100

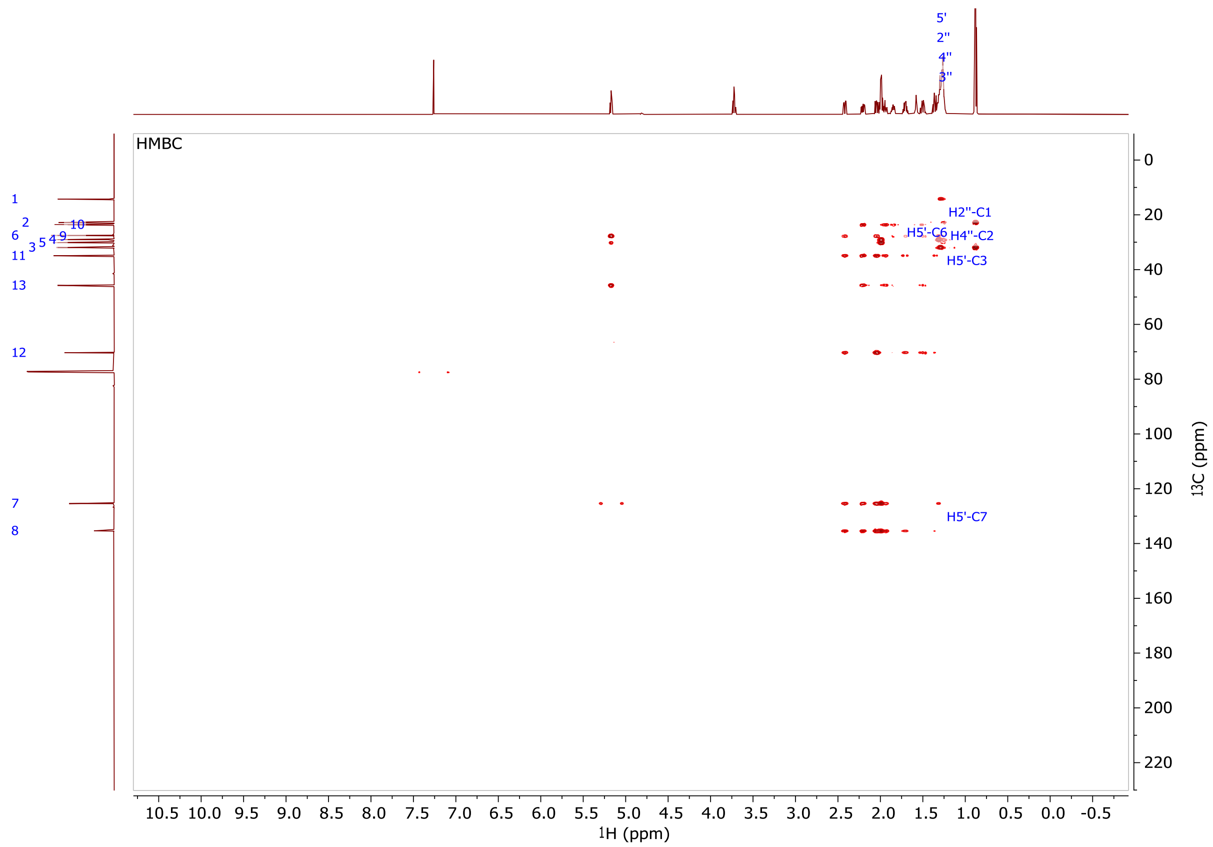
HMBC
5'
2''
4''
3''
1
2
10
6
3
5
4
9
11
13
12
7
8
H2''-C1
H5'-C6
H4''-C2
H5'-C3
H5'-C7
1H (ppm)
13C (ppm)

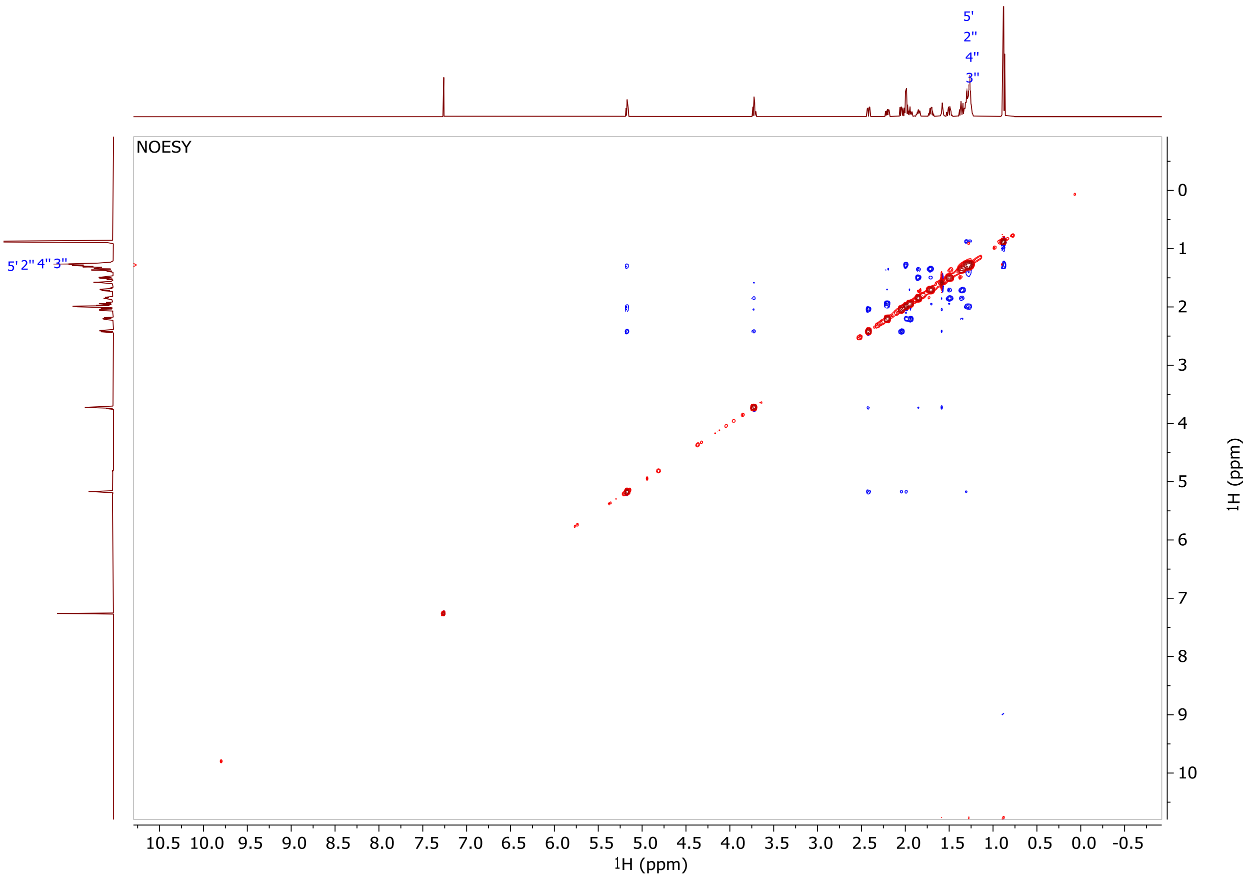
NOESY
5'
2''
4''
3''
5' 2'' 4'' 3''
1H (ppm)
1H (ppm)

(*S*,*E*)-3-(3-methylbutylidene)cyclohexan-1-ol (2d)

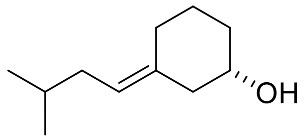

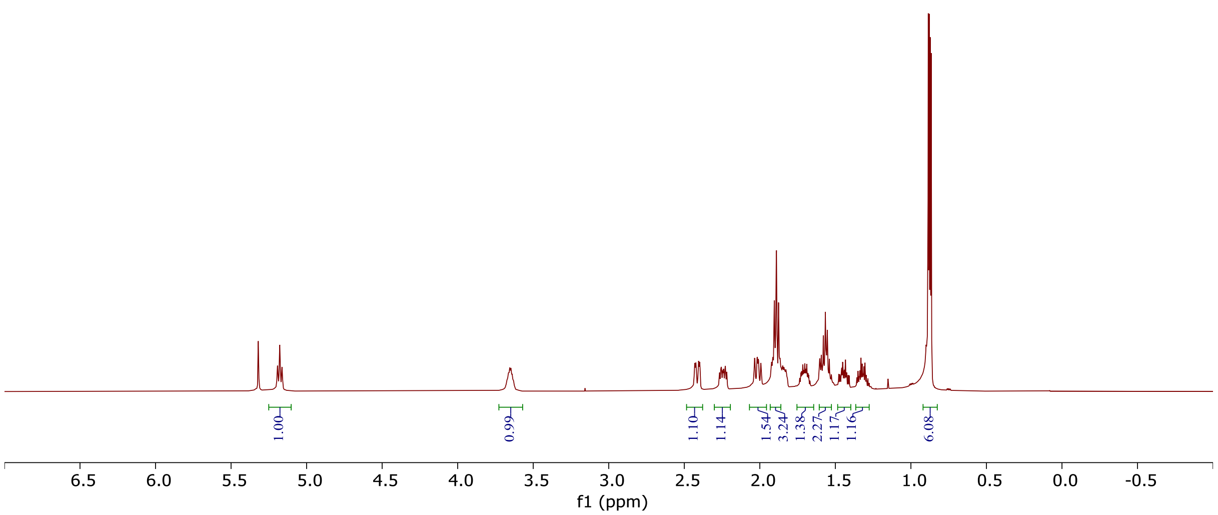

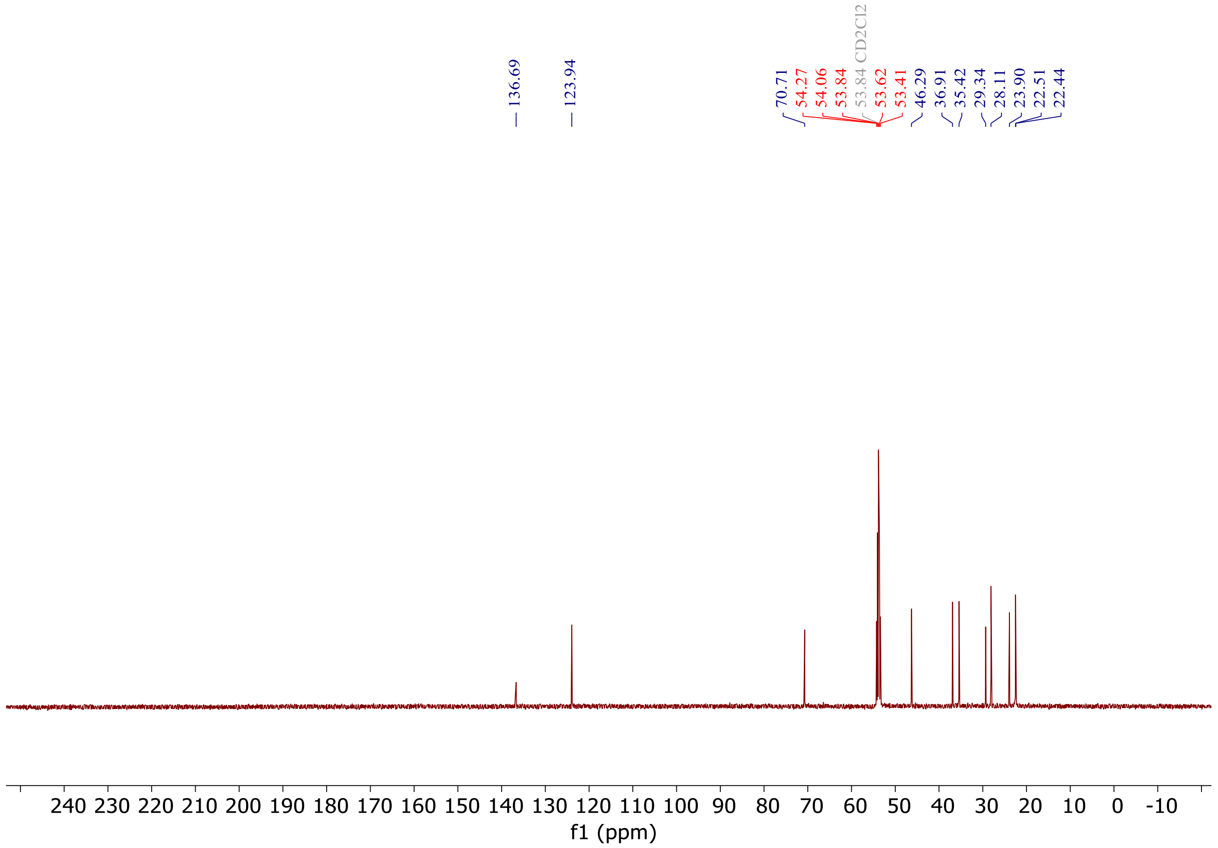

(*S*,*E*)-3-(but-3-en-1-ylidene)cyclohexan-1-ol (2e)

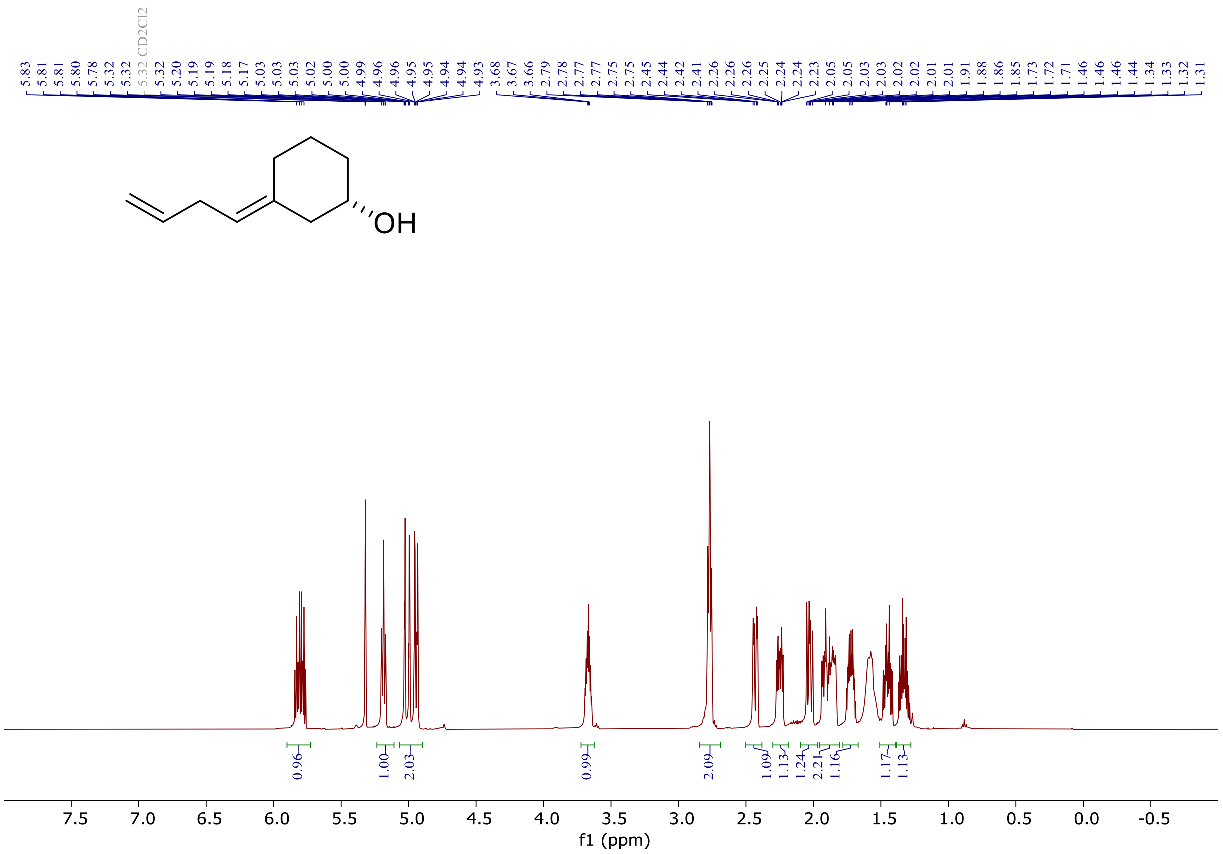

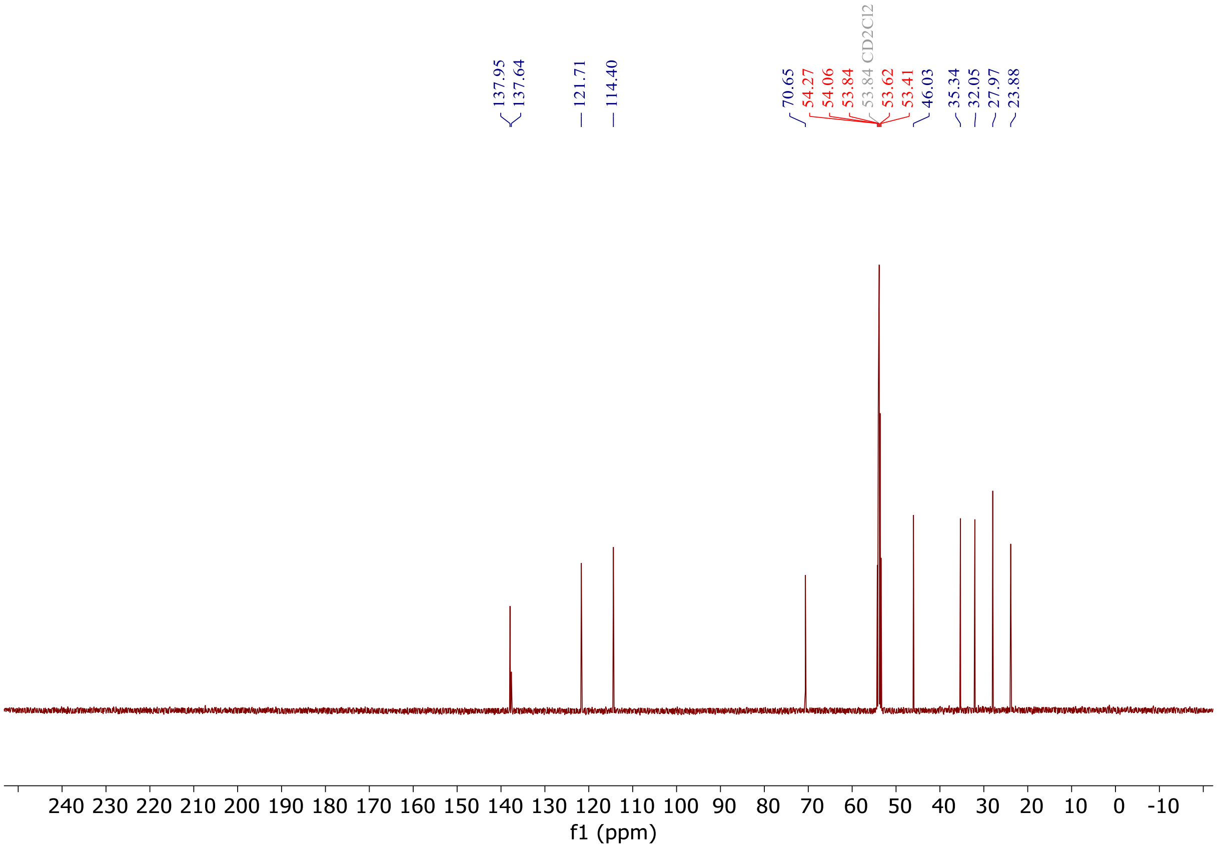

(*S*,*E*)-3-(pent-4-en-1-ylidene)cyclohexan-1-ol (2f)

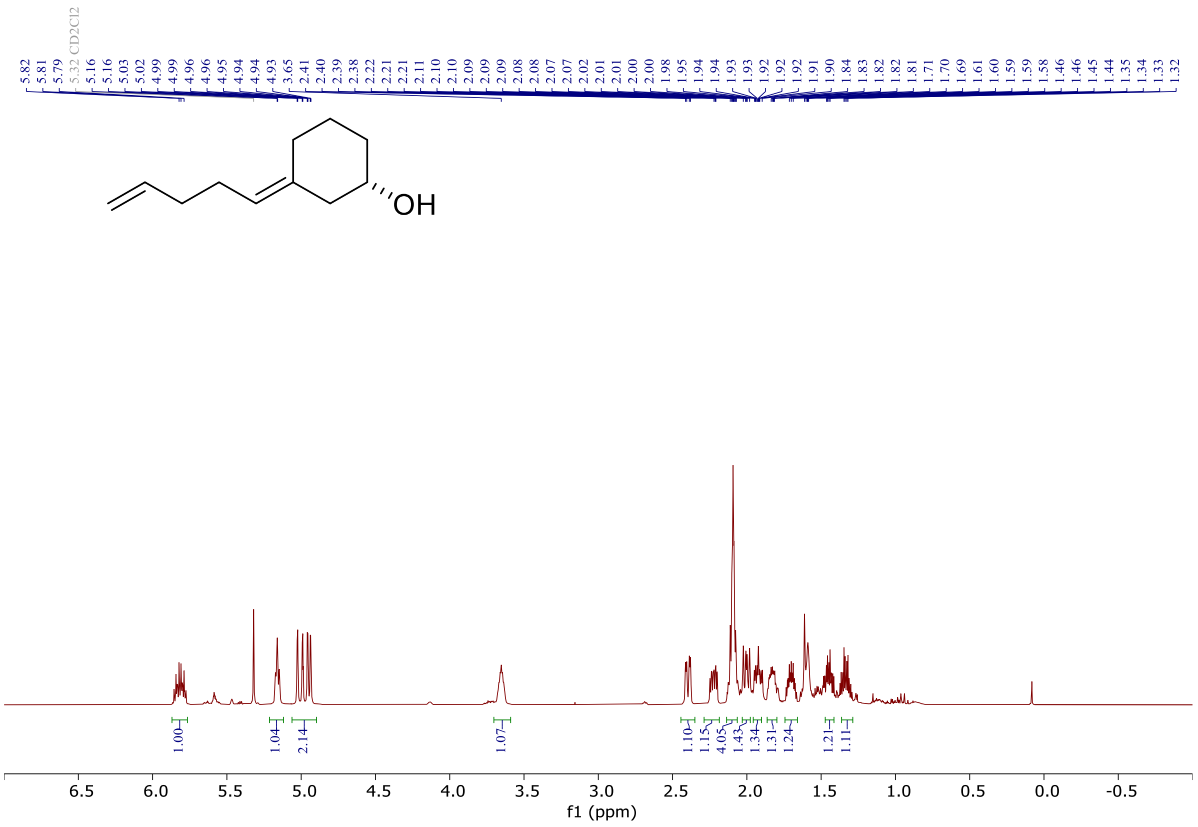

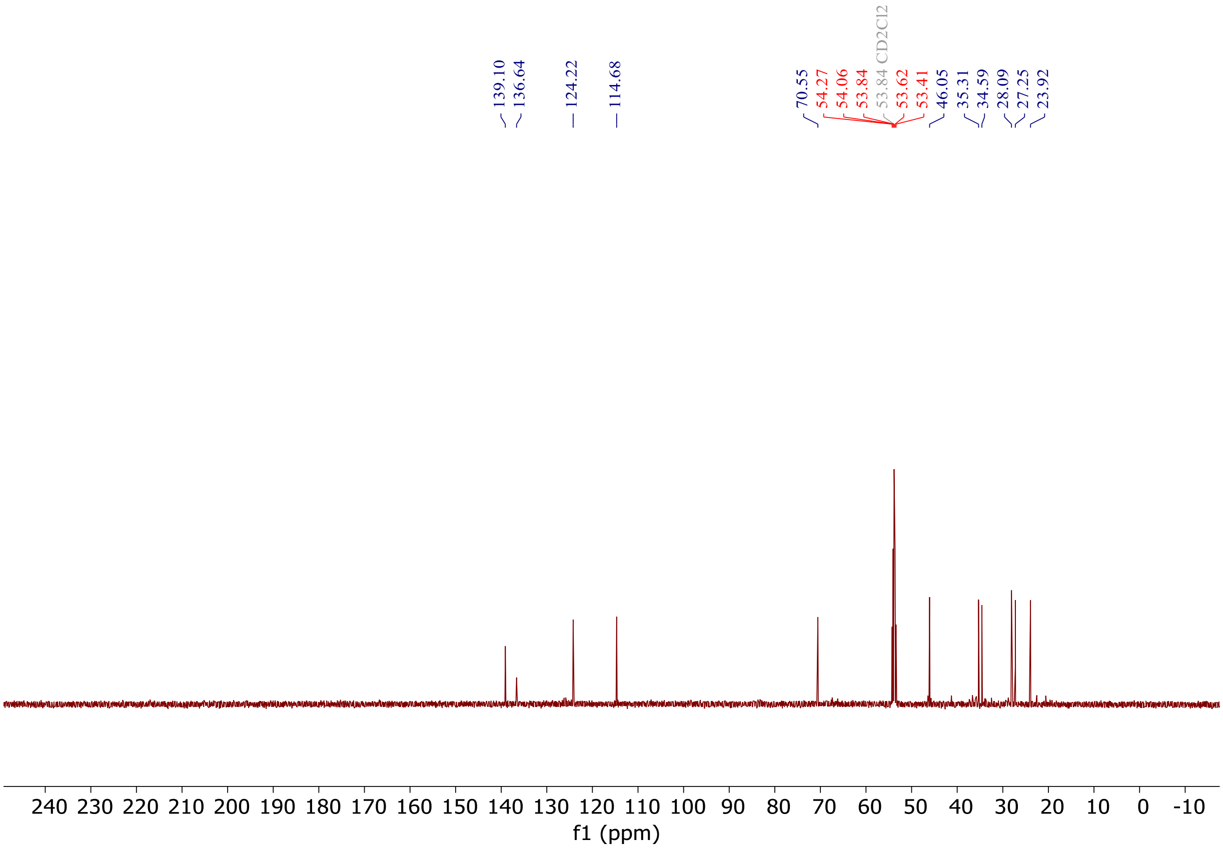

(*S*,*E*)-3-(2-phenylethylidene)cyclohexan-1-ol (2g)

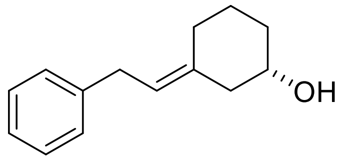

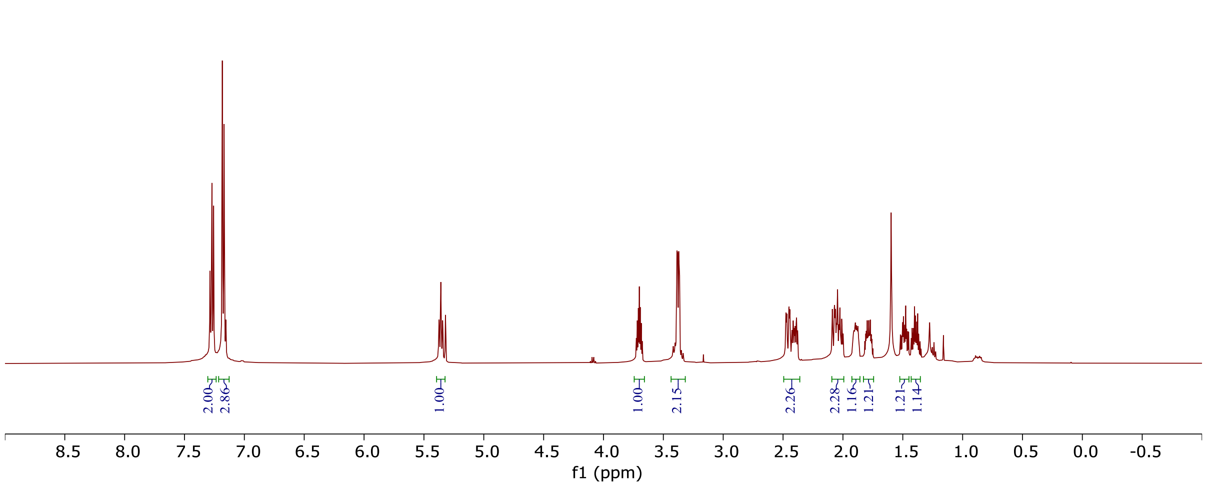

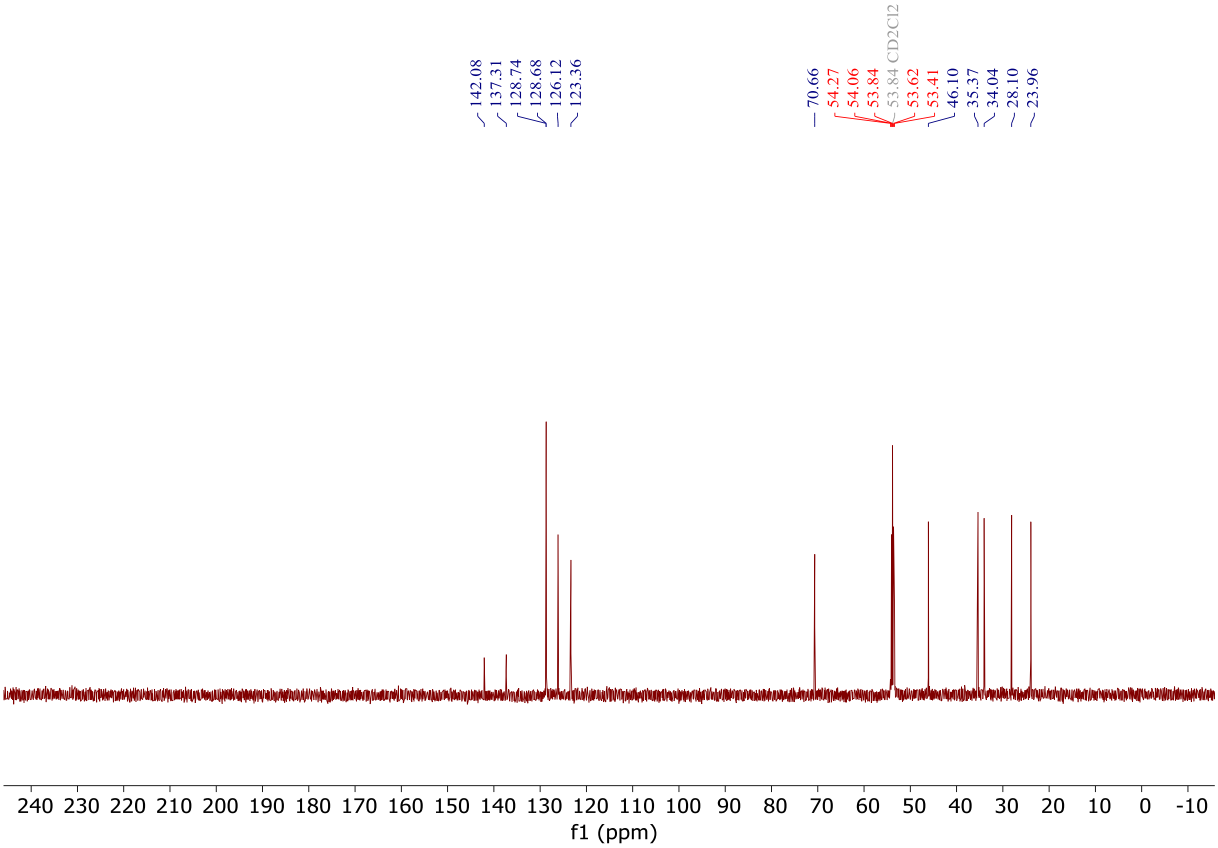

(*S*,*E*)-3-(2-(*p*-tolyl)ethylidene)cyclohexan-1-ol (2h)

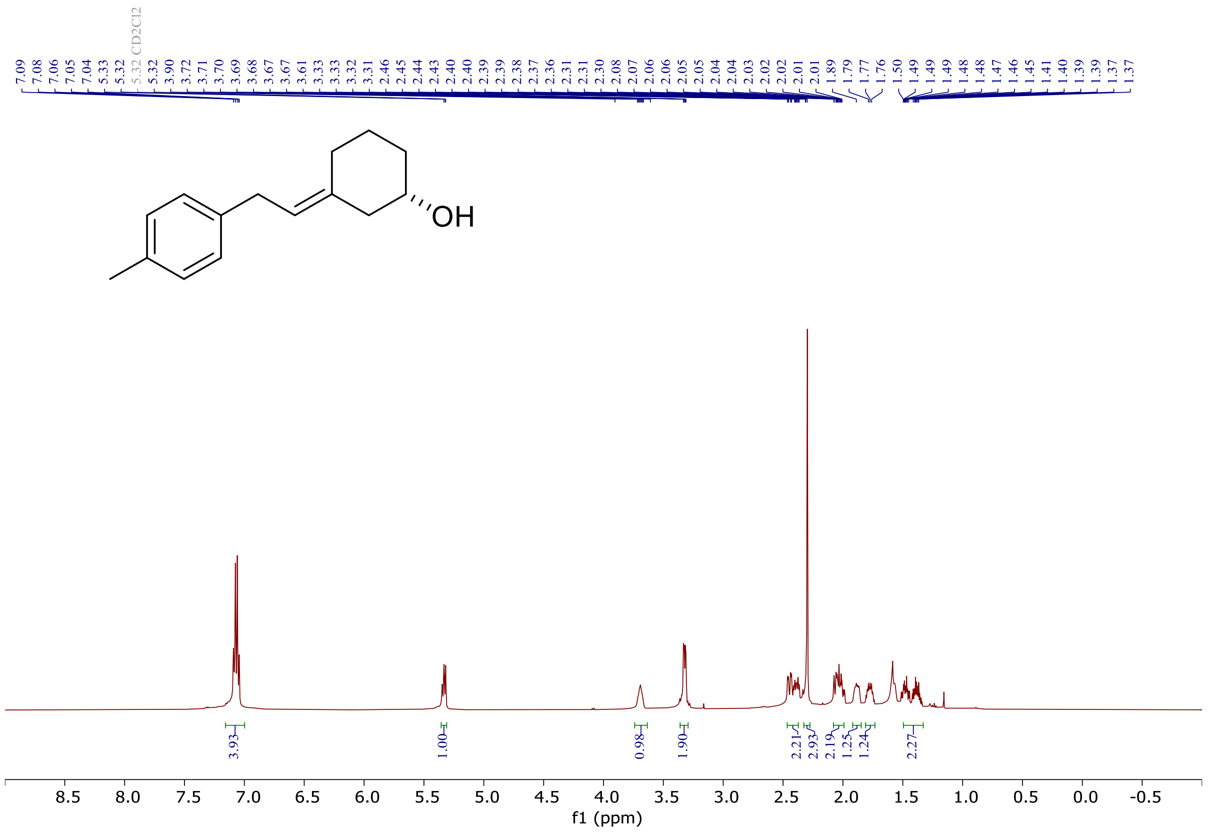

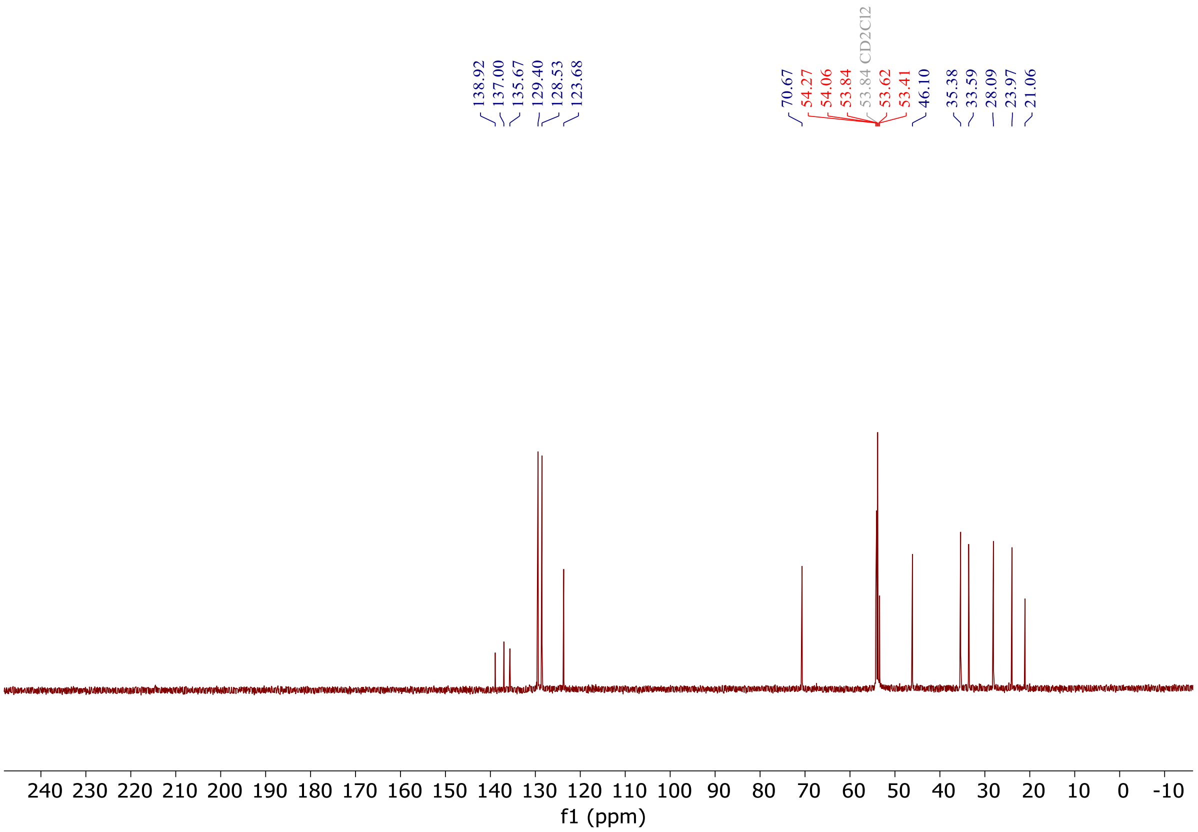

(*S*)-3-(propan-2-ylidene)cyclohexan-1-ol (2i)

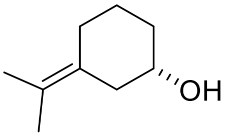

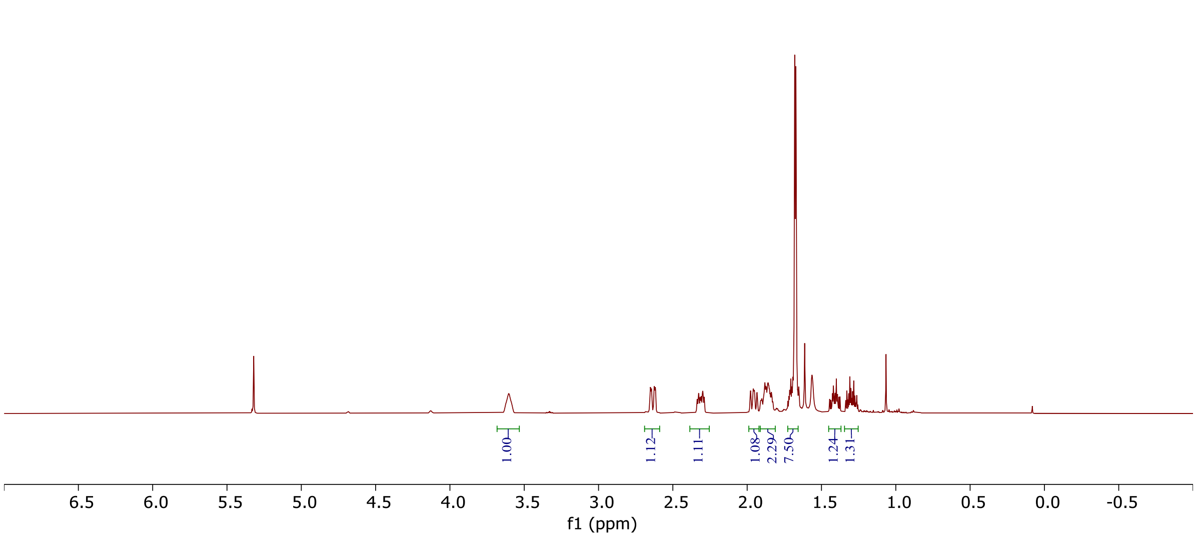

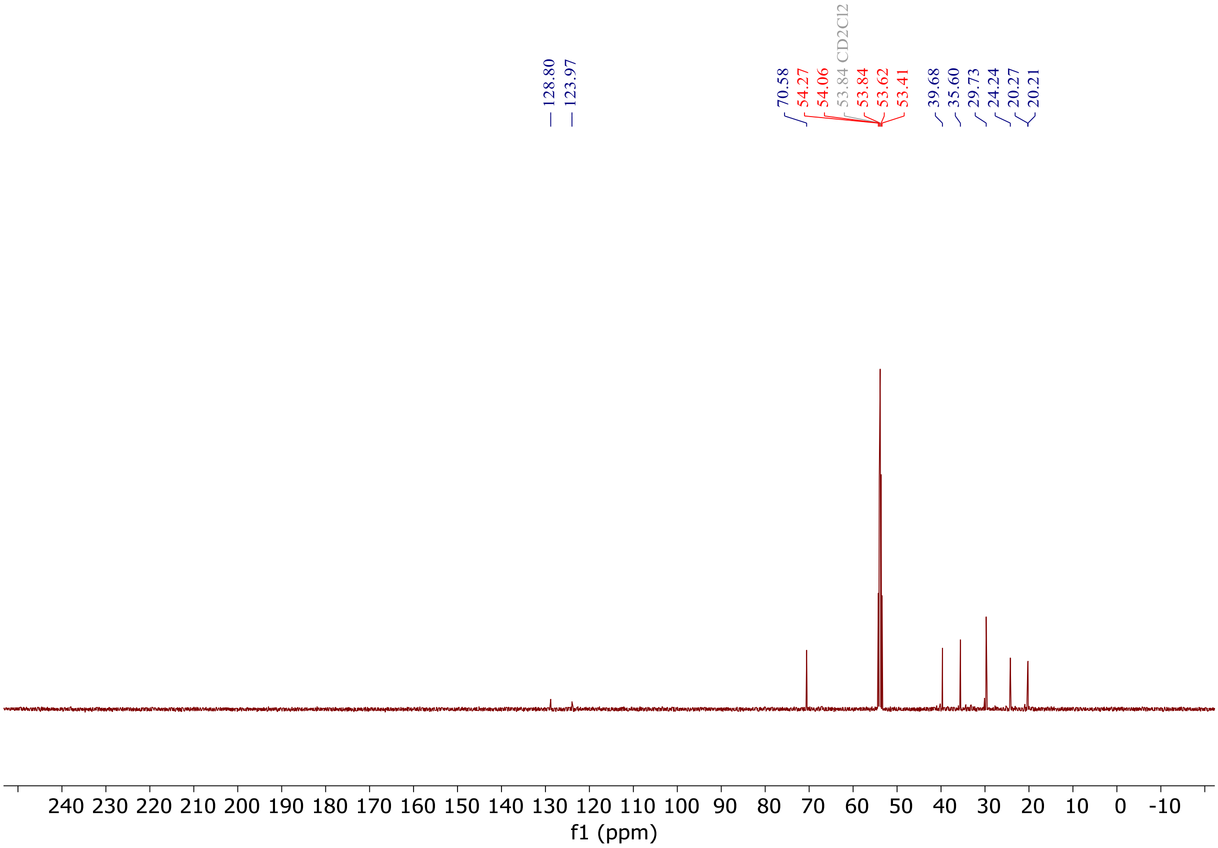

(*S*)-3-cyclopentylidenecyclohexan-1-ol (2j)

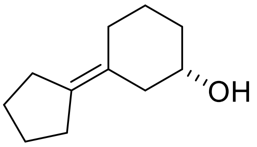

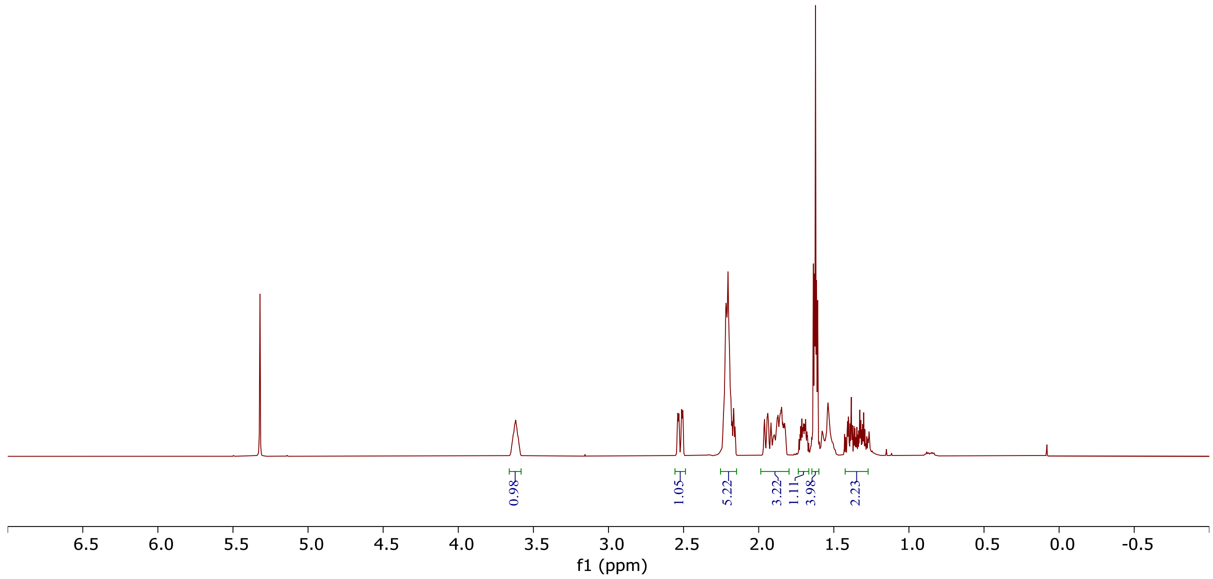

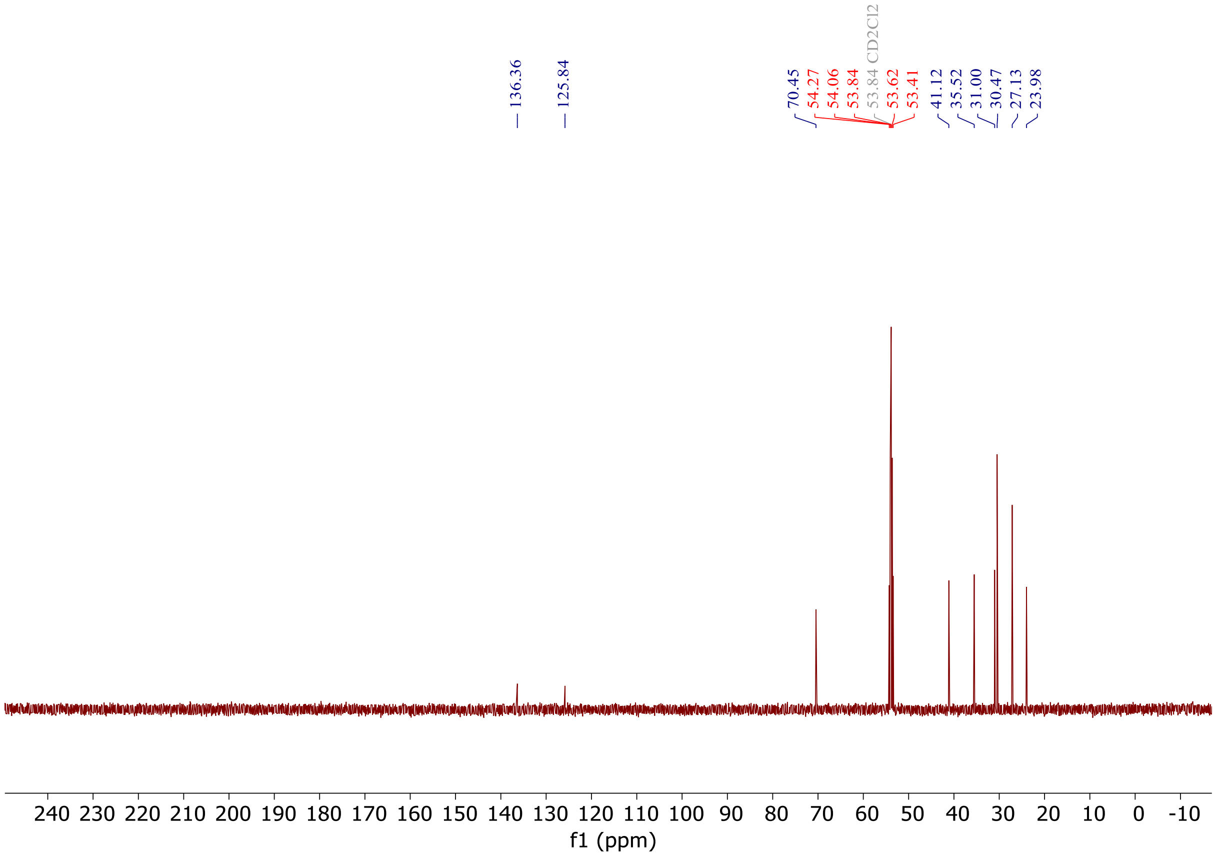

(*S*)-[1,1'-bi(cyclohexylidene)]-3-ol (2k)

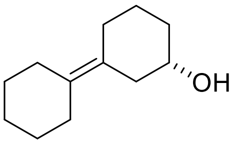

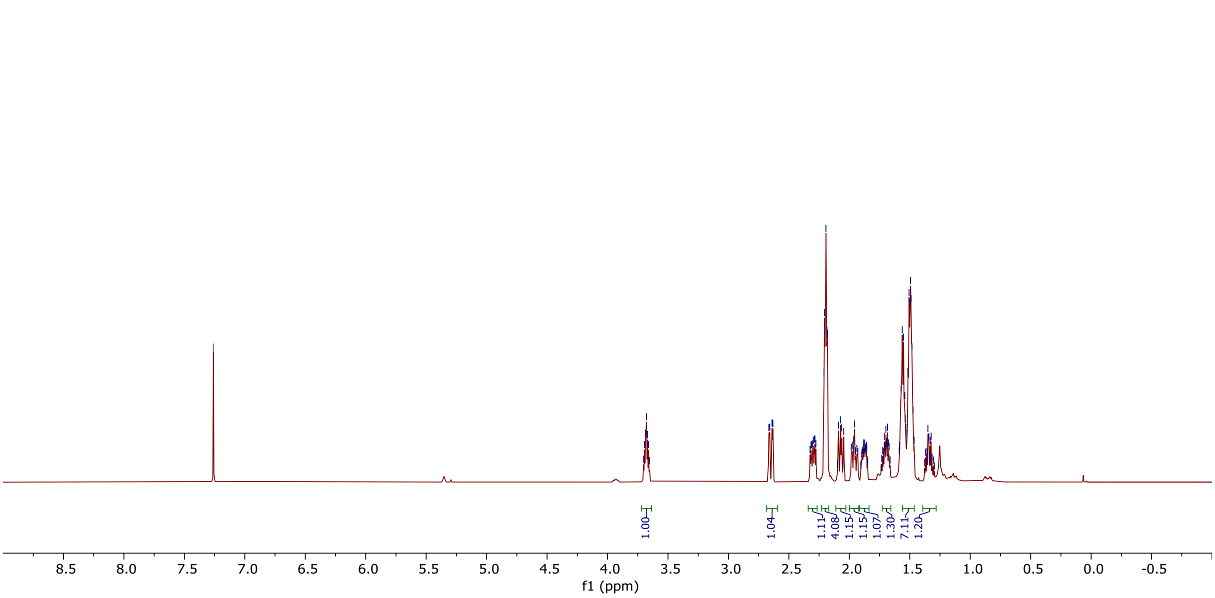

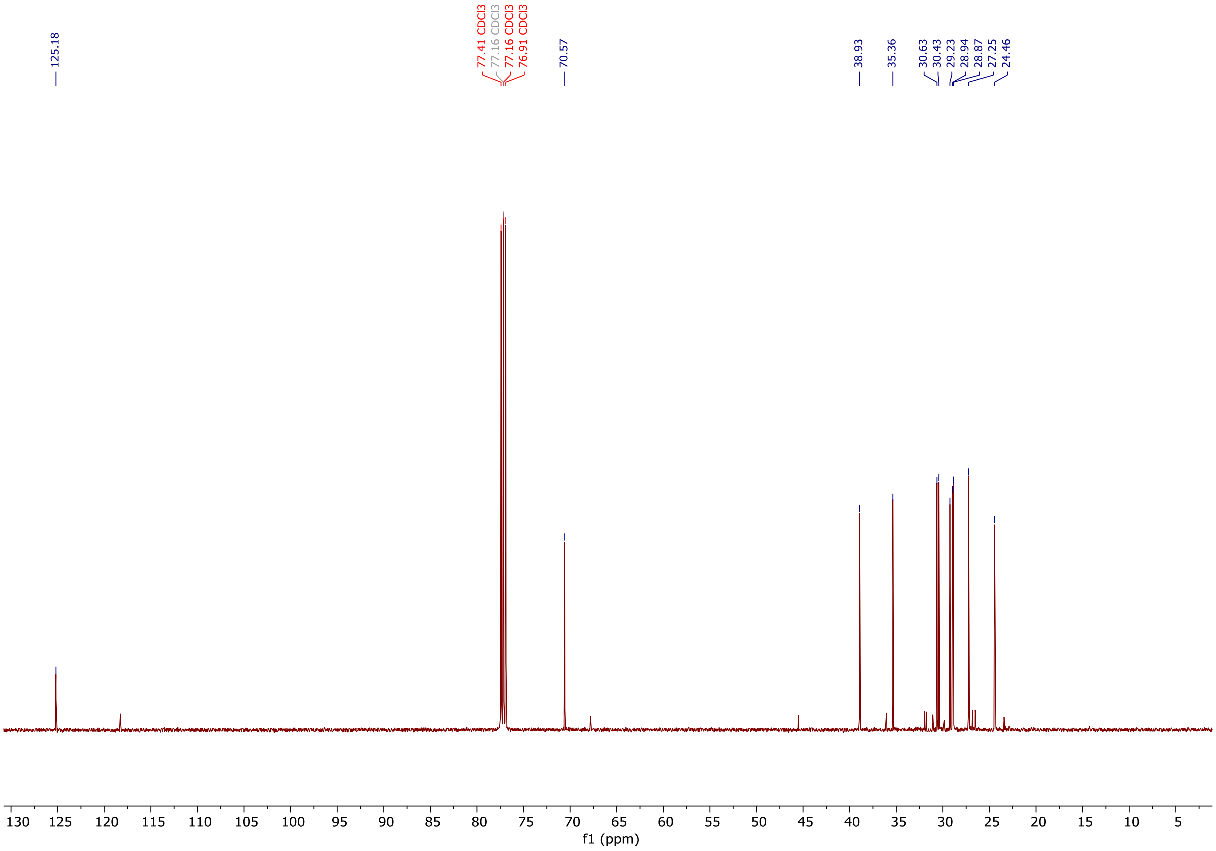

(*S*,*E*)-3-(2-methylpropylidene)cyclohexan-1-ol (2l)

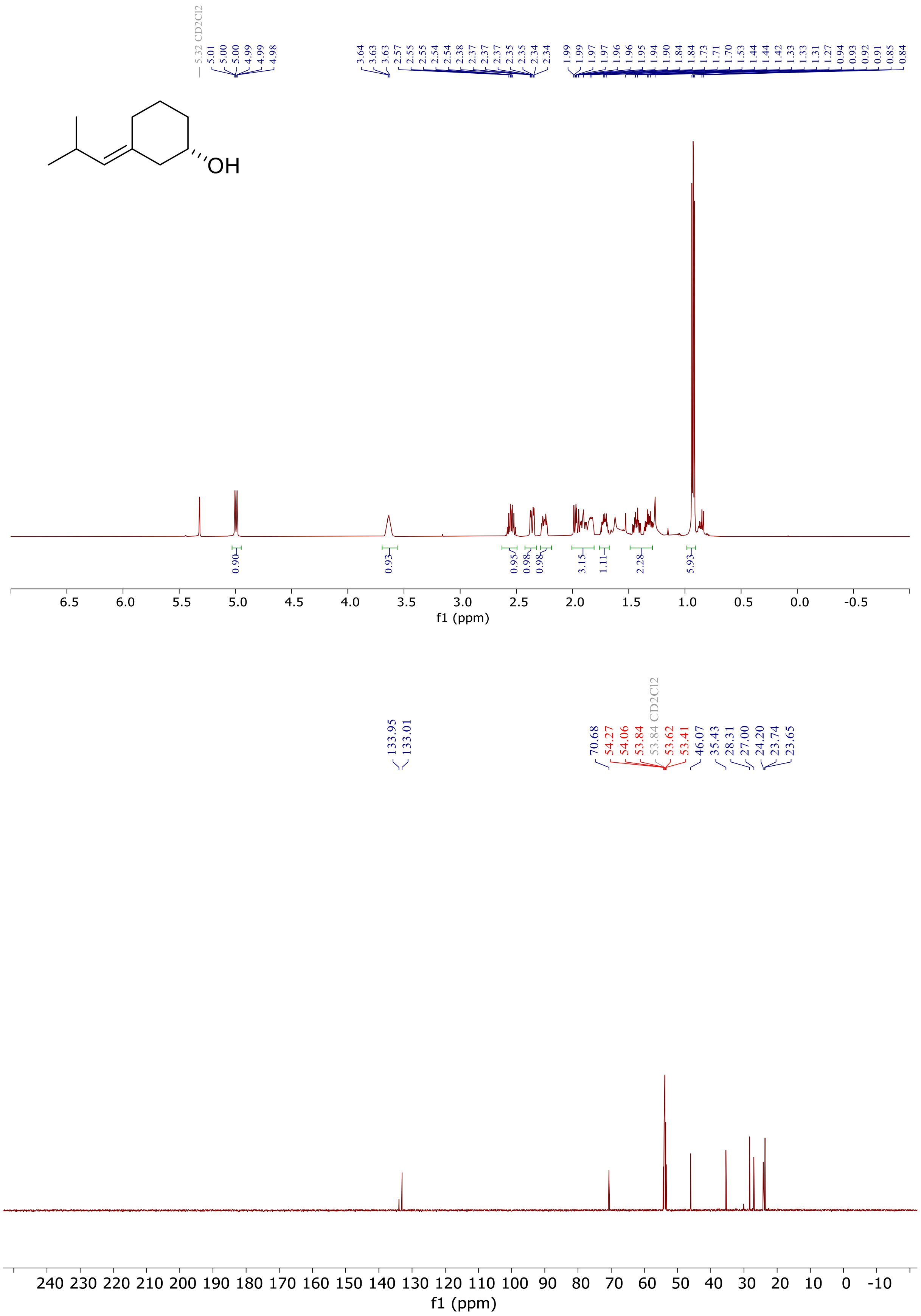

(*S*,*E*)-3-(cyclohexylmethylene)cyclohexan-1-ol (2m)

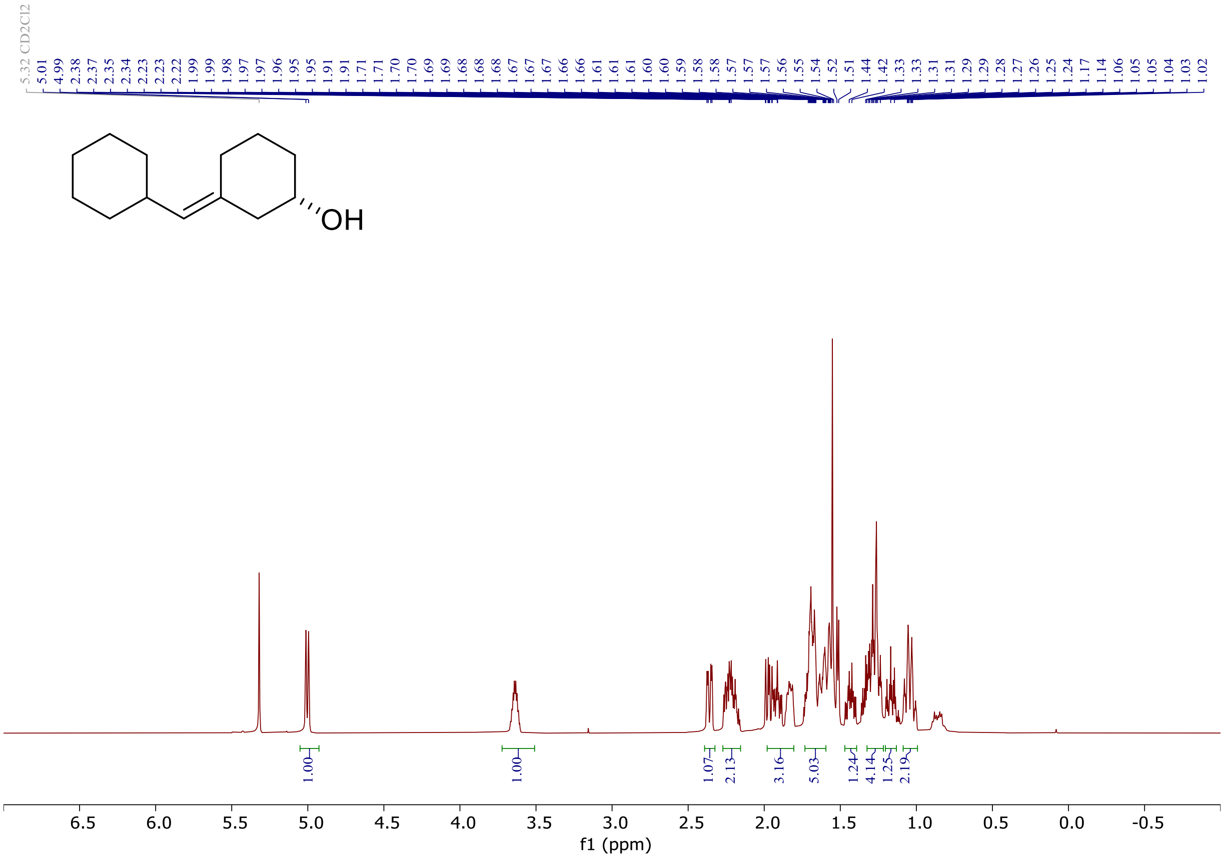

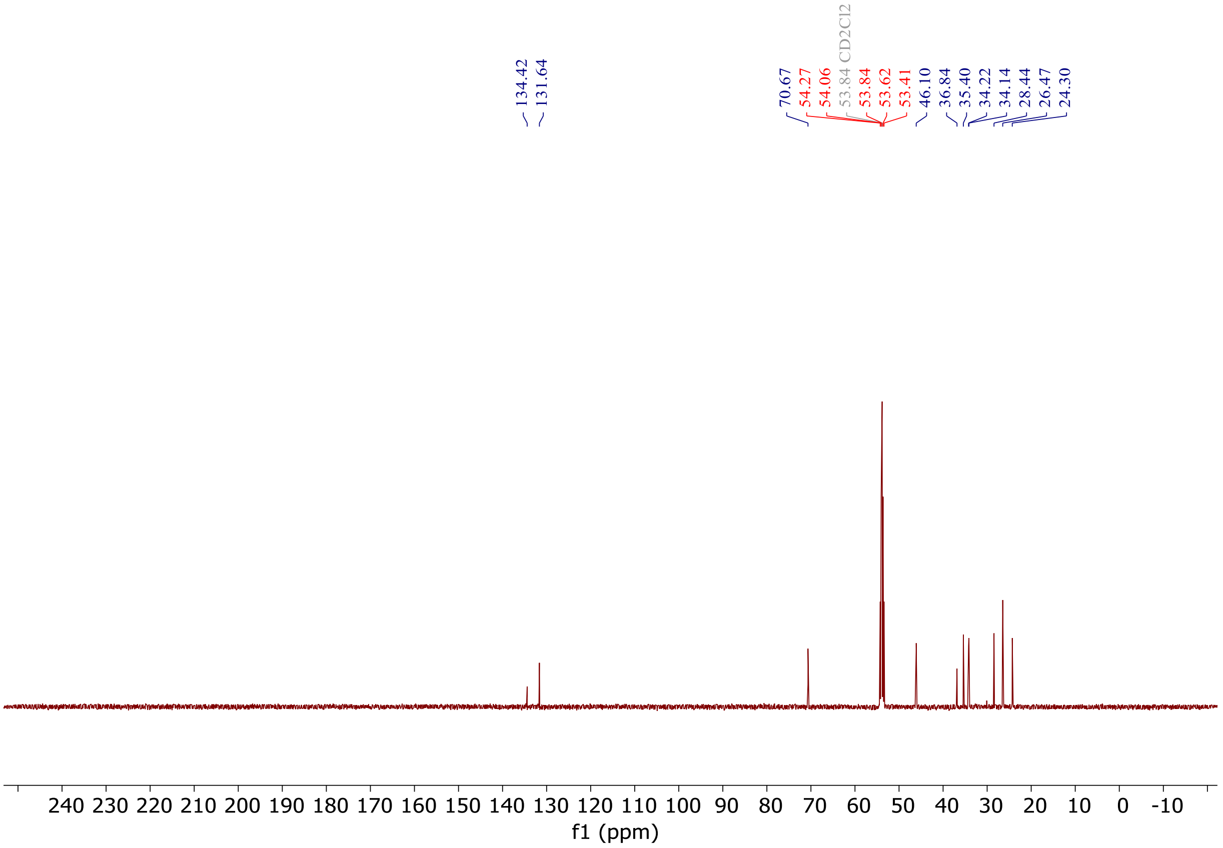

(*S*,*E*)-3-(3-methoxypropylidene)cyclohexan-1-ol (2n)

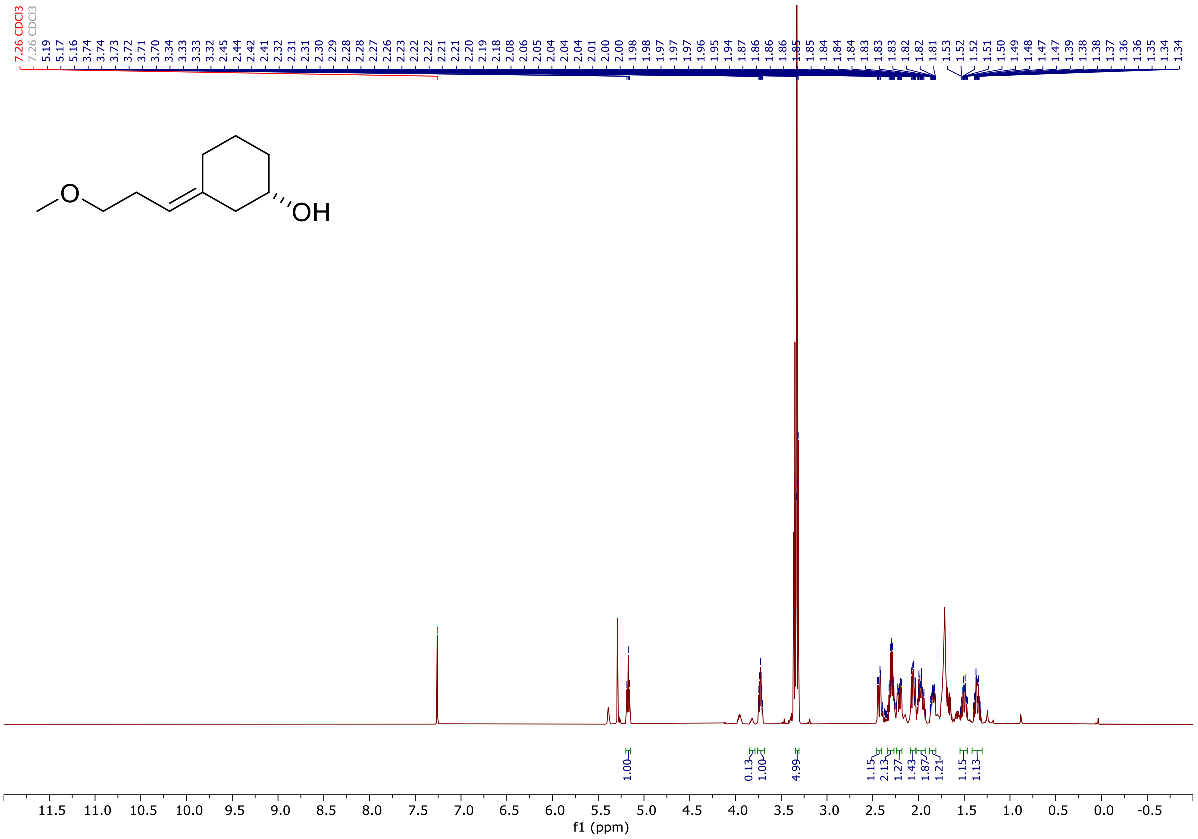

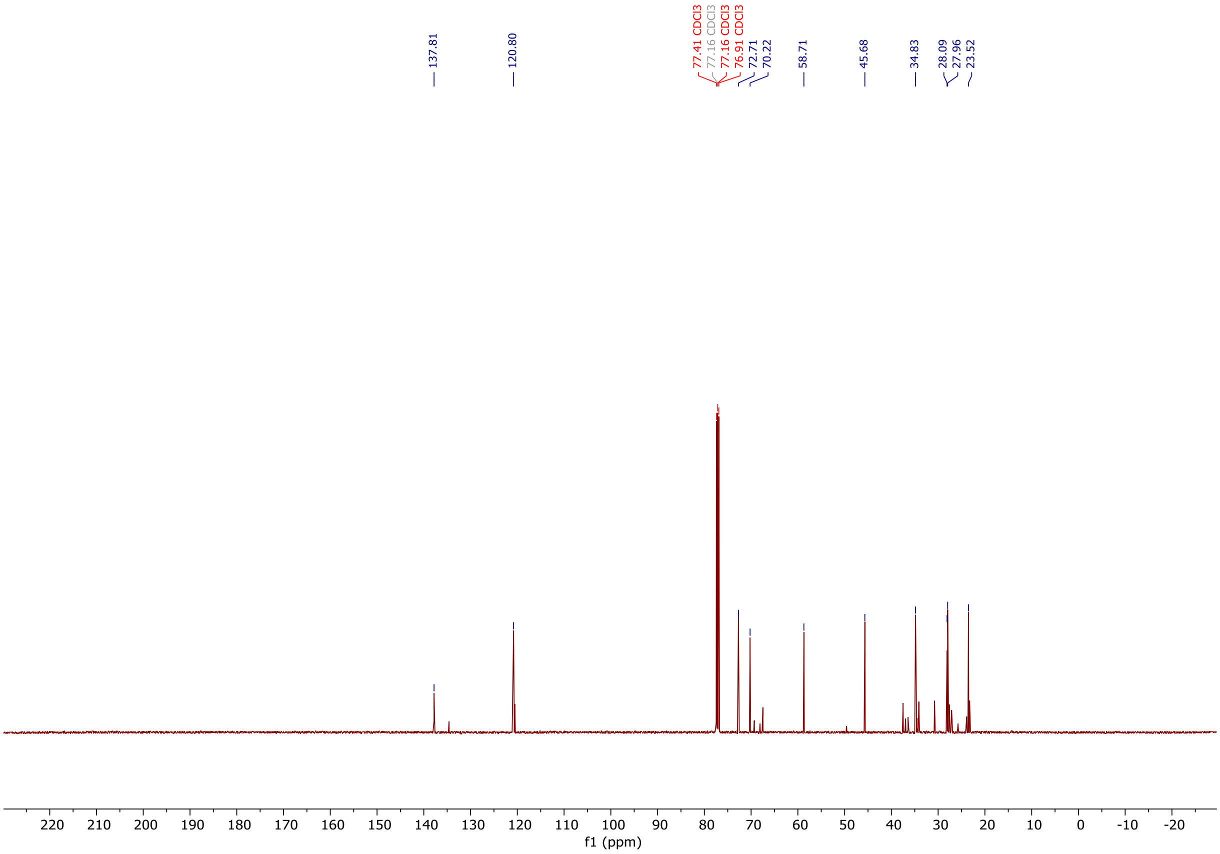

(*S*,*E*)-3-(4-(thiophen-2-yl)butylidene)cyclohexan-1-ol (2o)

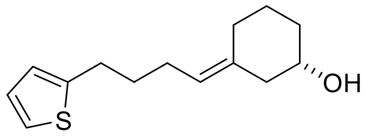

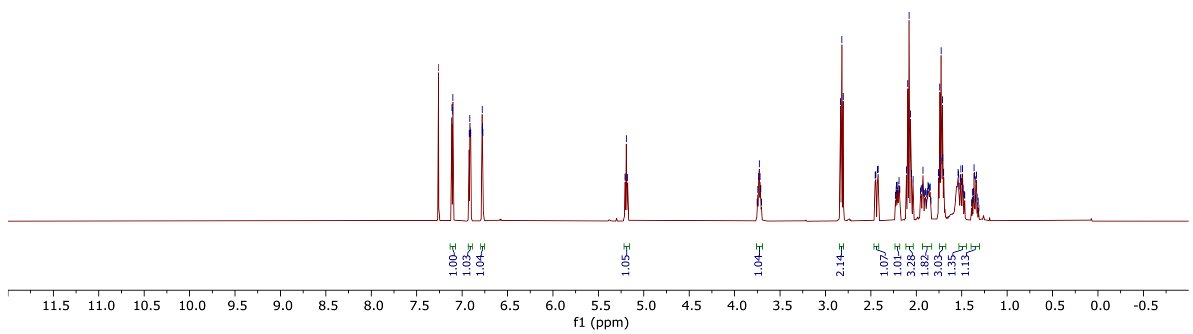

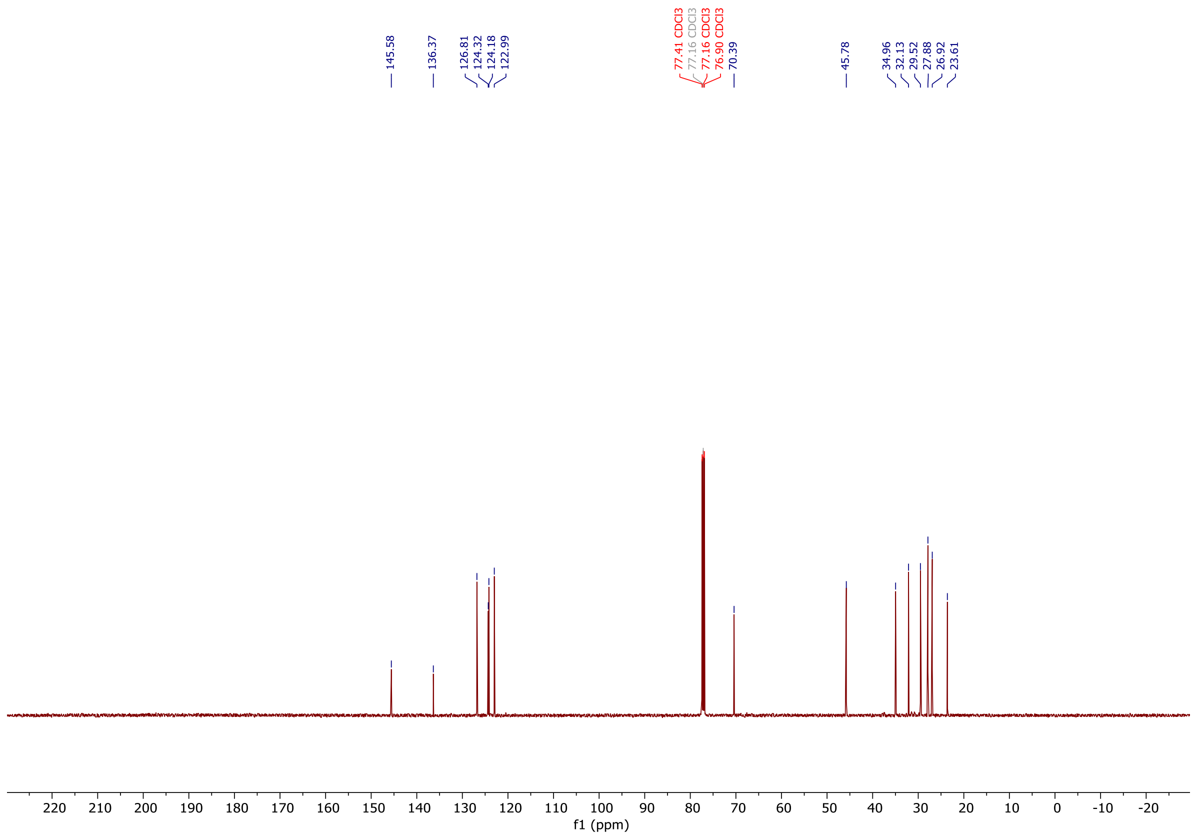

(*S*,*E*)-3-(4-hydroxybutylidene)cyclohexan-1-ol (2p)

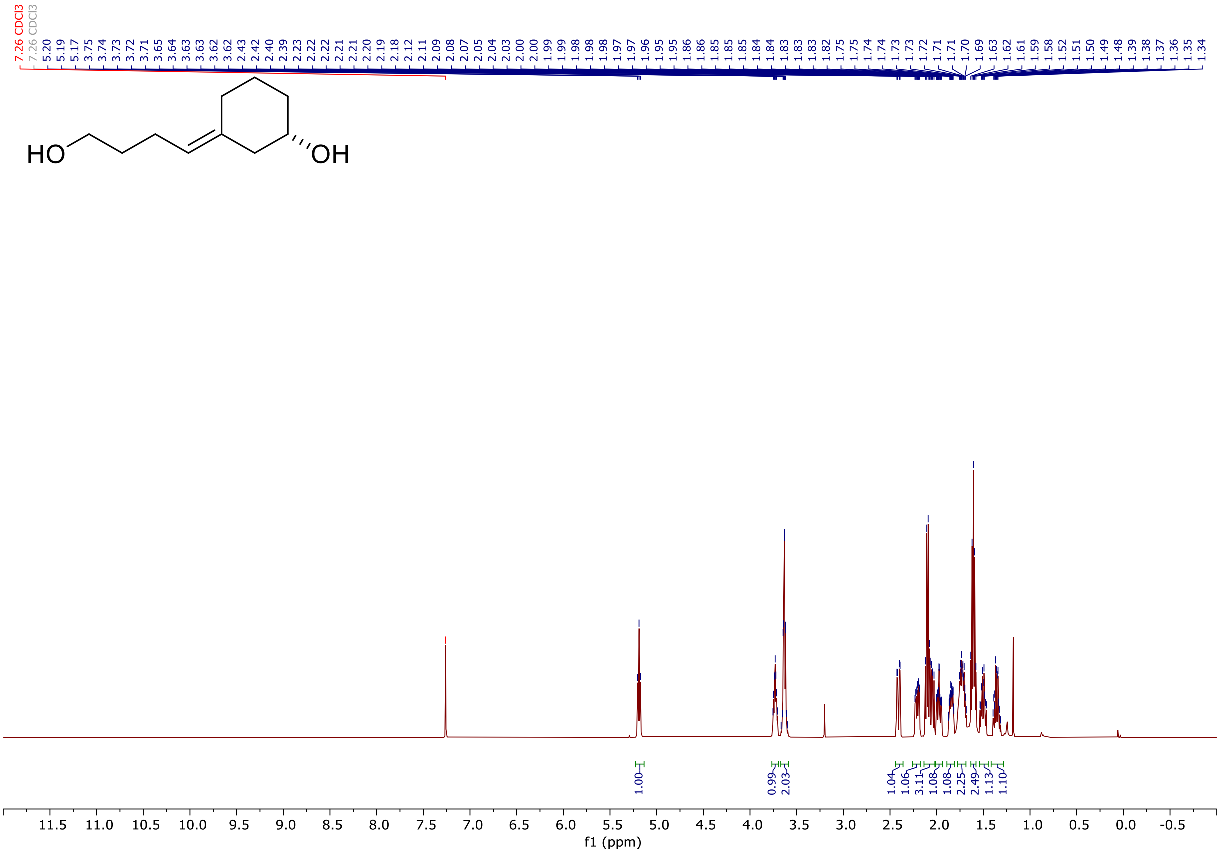

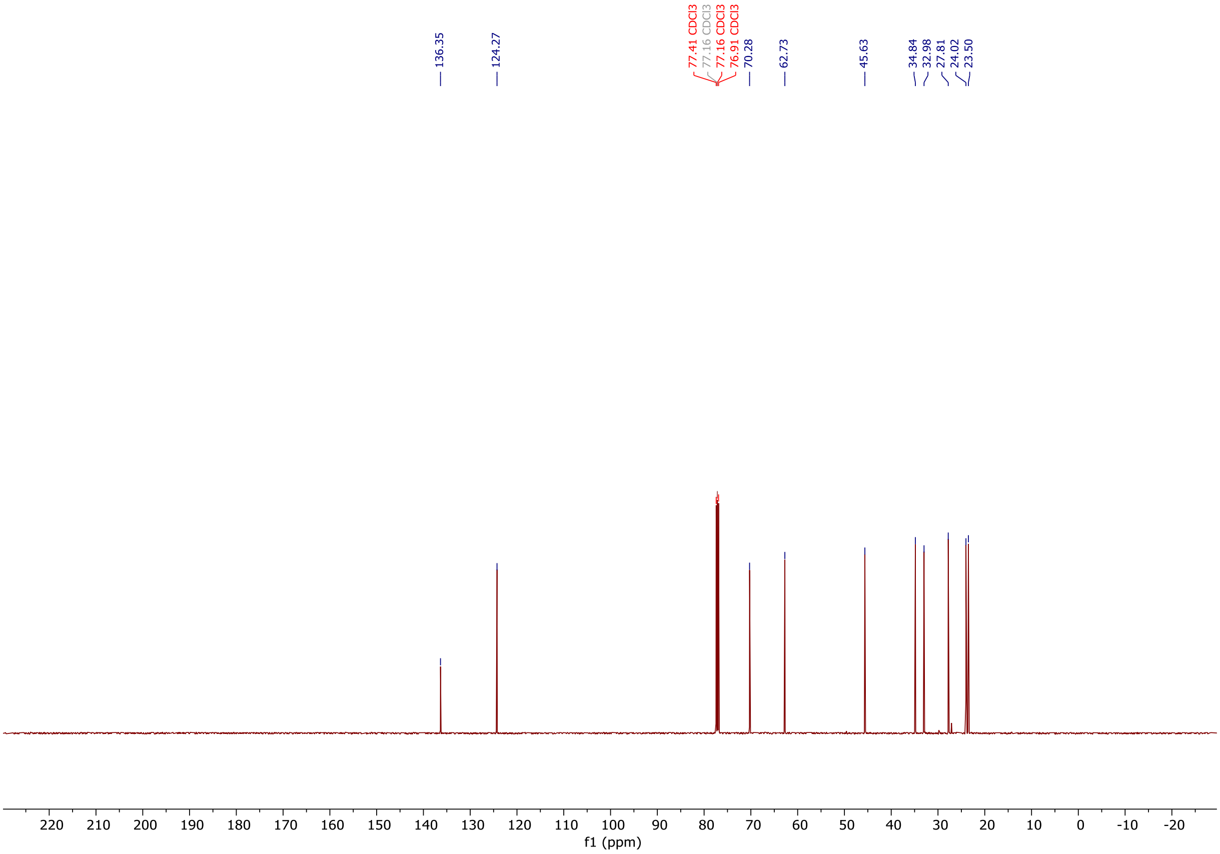

ethyl (*S*,*E*)-4-(3-hydroxycyclohexylidene)butanoate (2q)

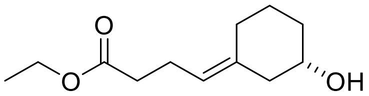

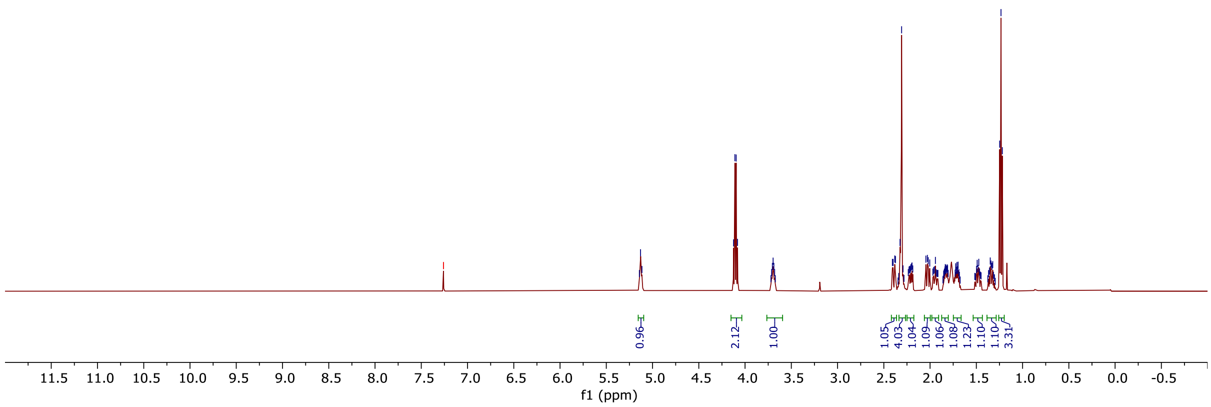

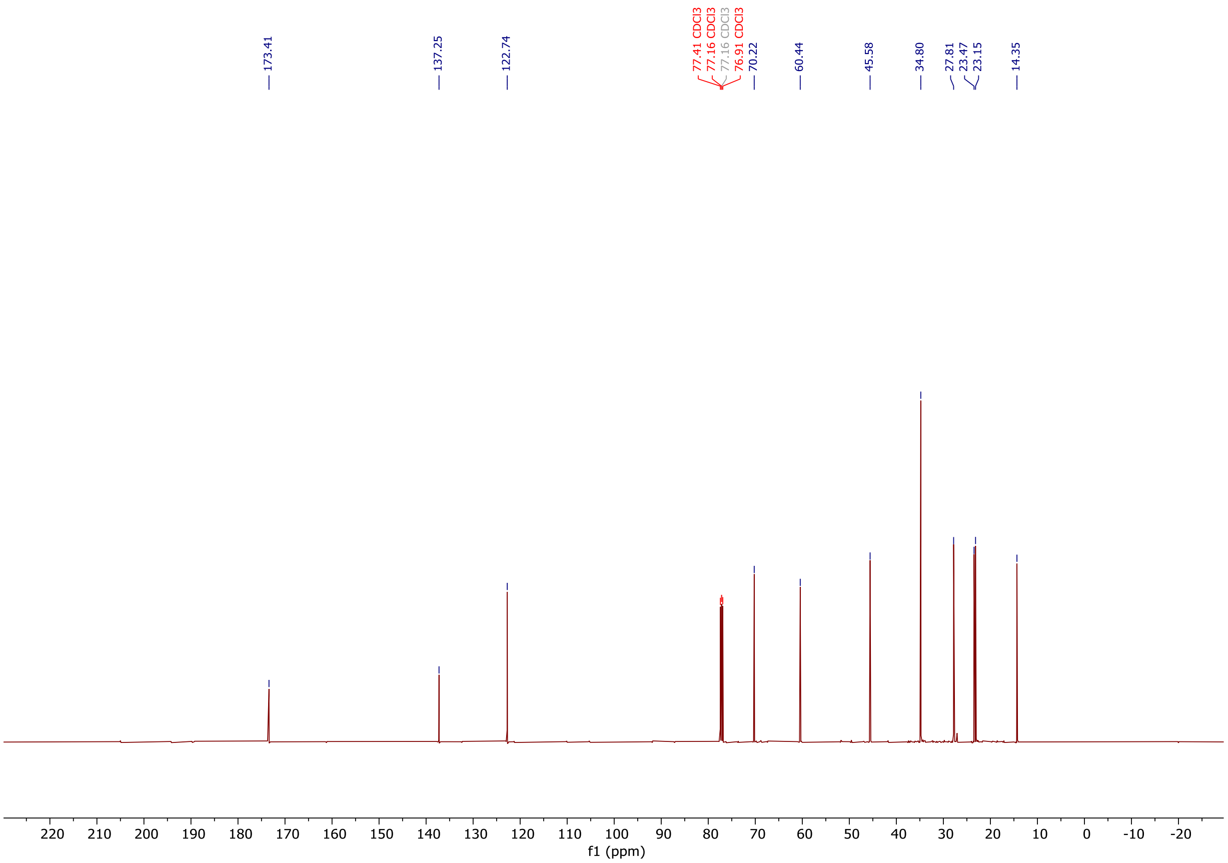

(*S*,*E*)-3-(4-bromobutylidene)cyclohexan-1-ol (2r)

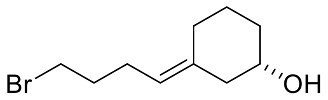

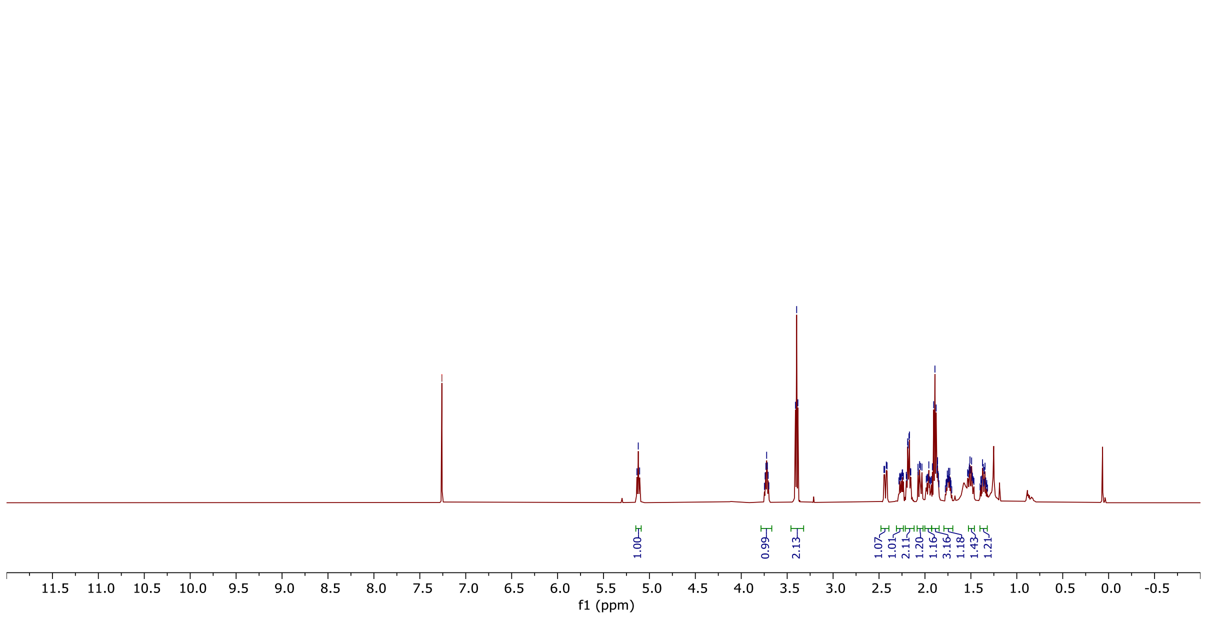

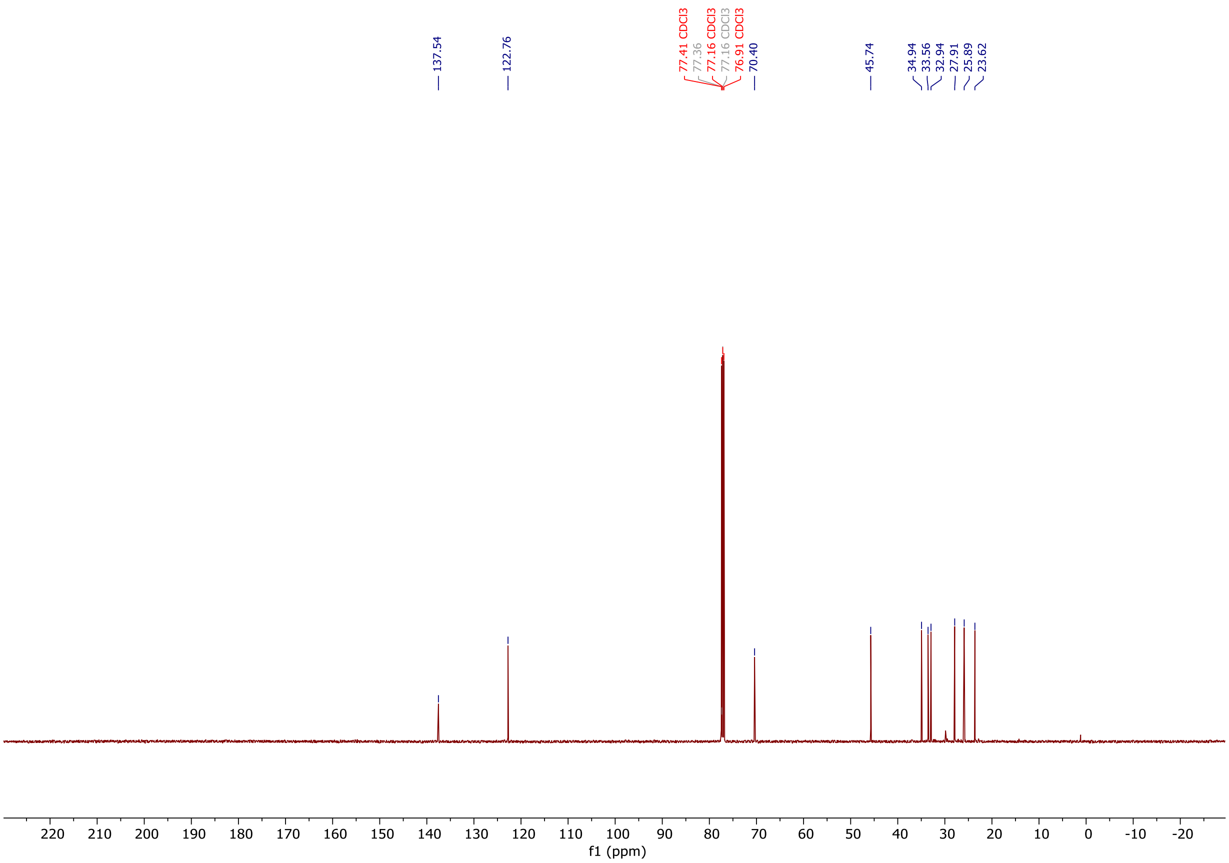

(*S*)-3-methylenecycloheptan-1-ol (2s)

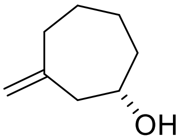

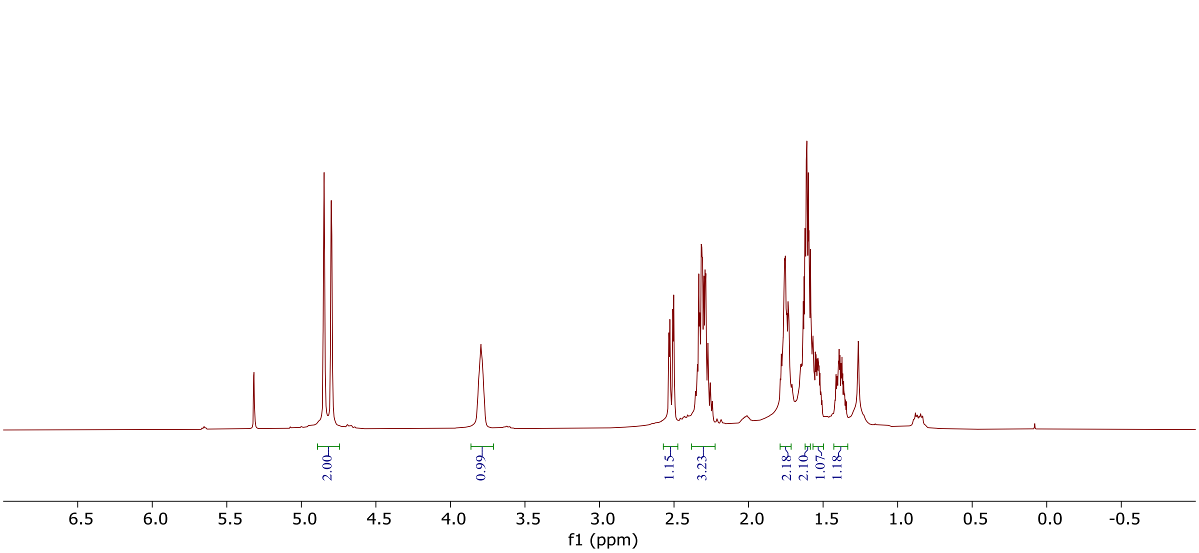

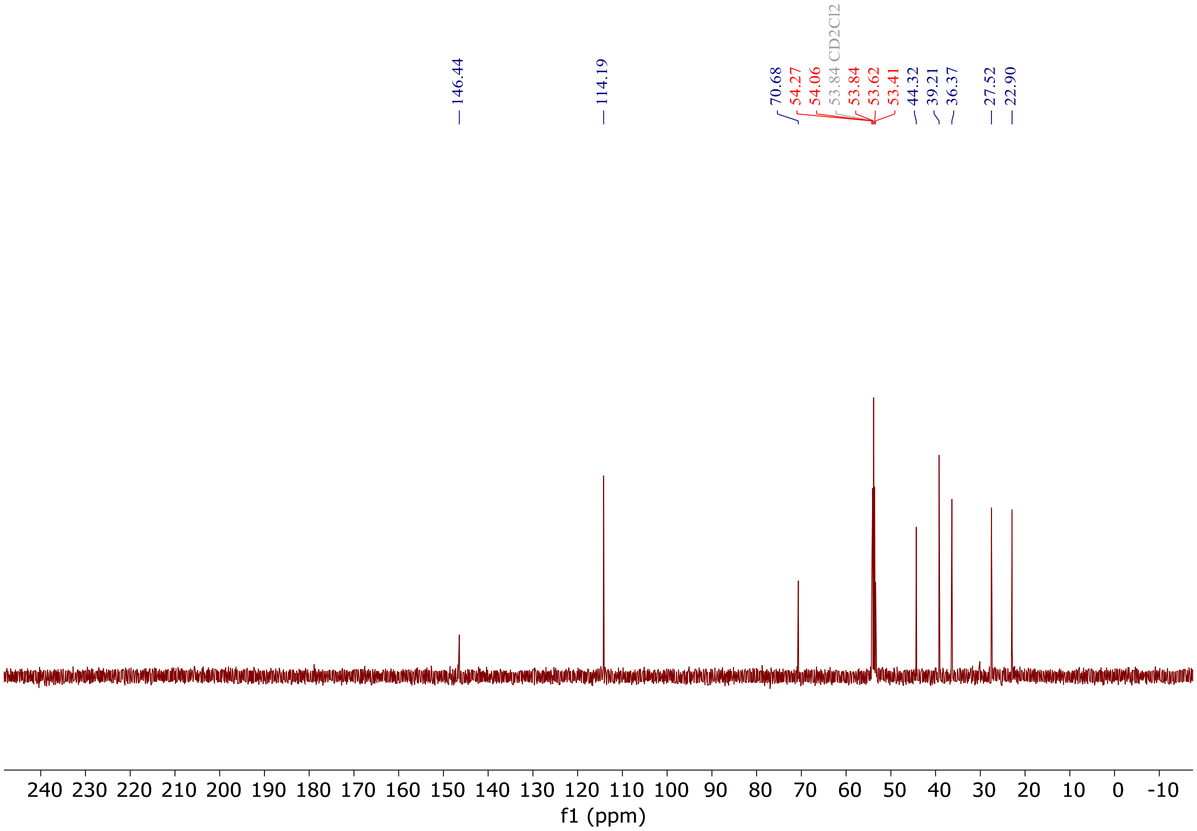

(*S*,*E*)-3-butylidenecycloheptan-1-ol (2t)

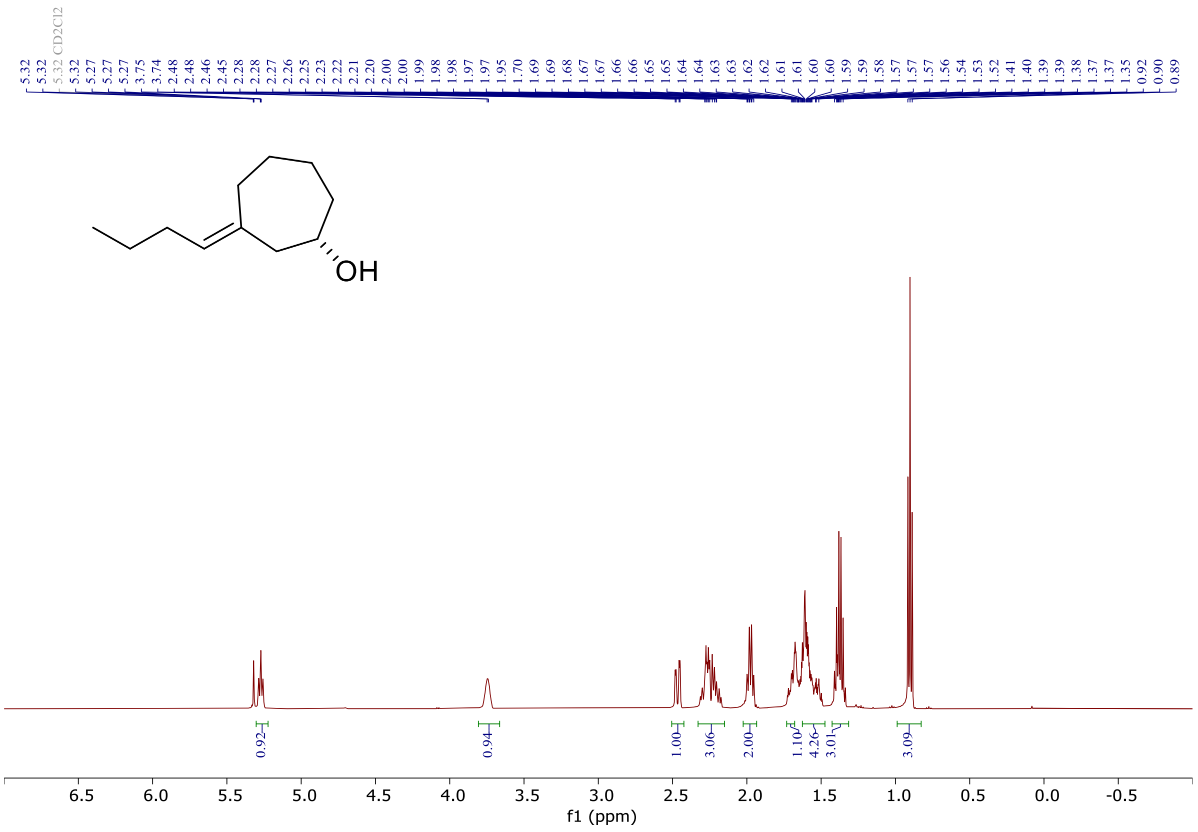

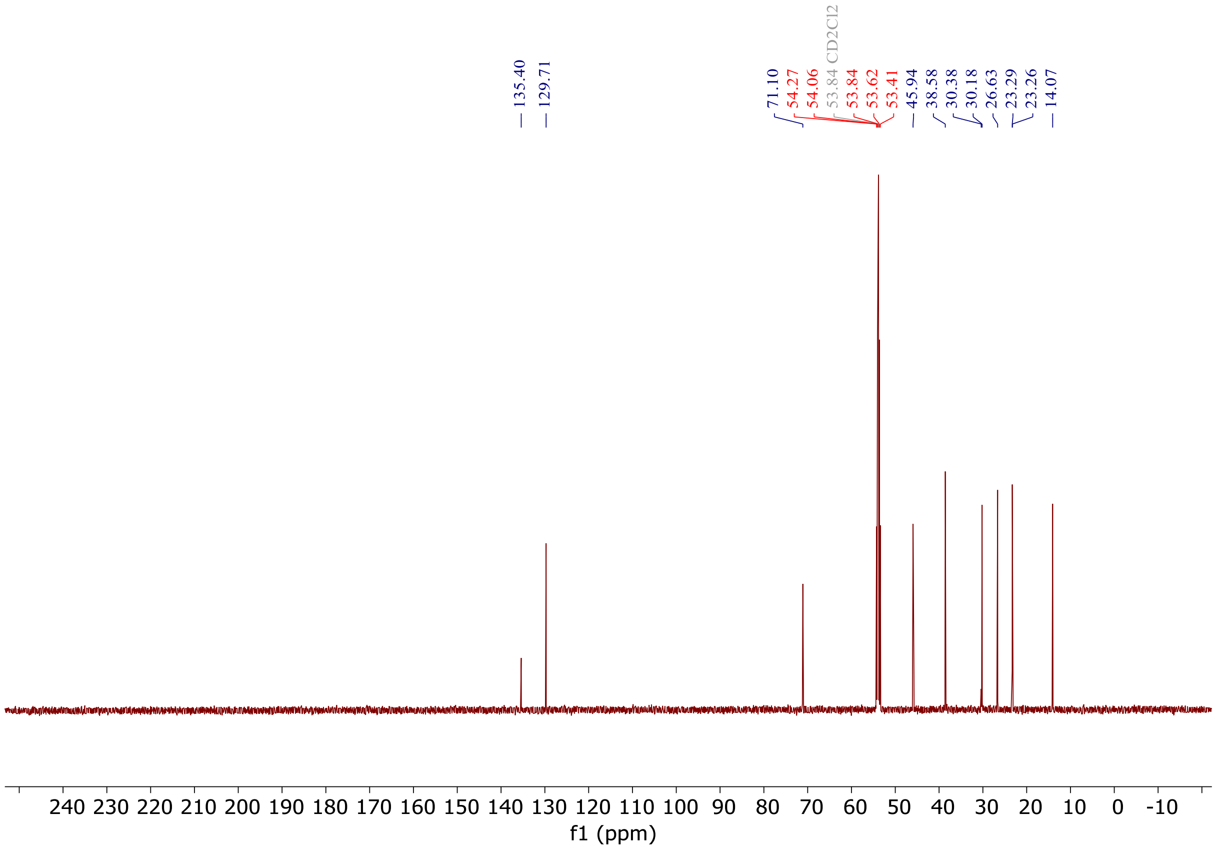

(*S*)-3,3-dimethyl-5-methylenecyclohexan-1-ol (2u)

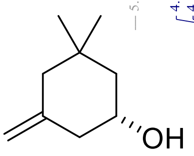

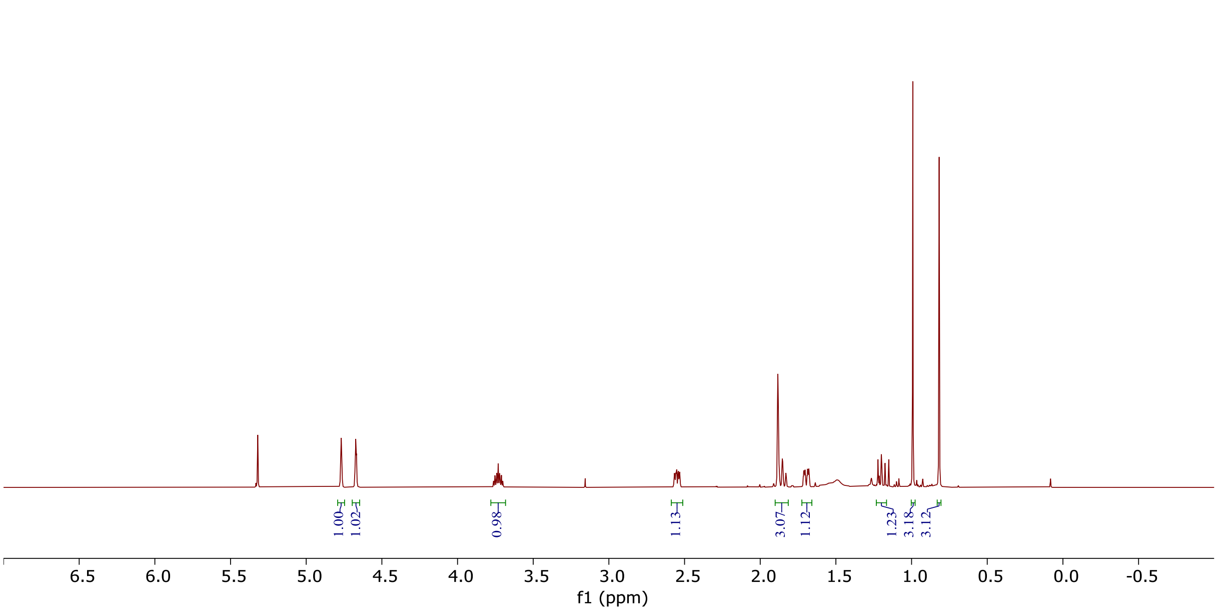

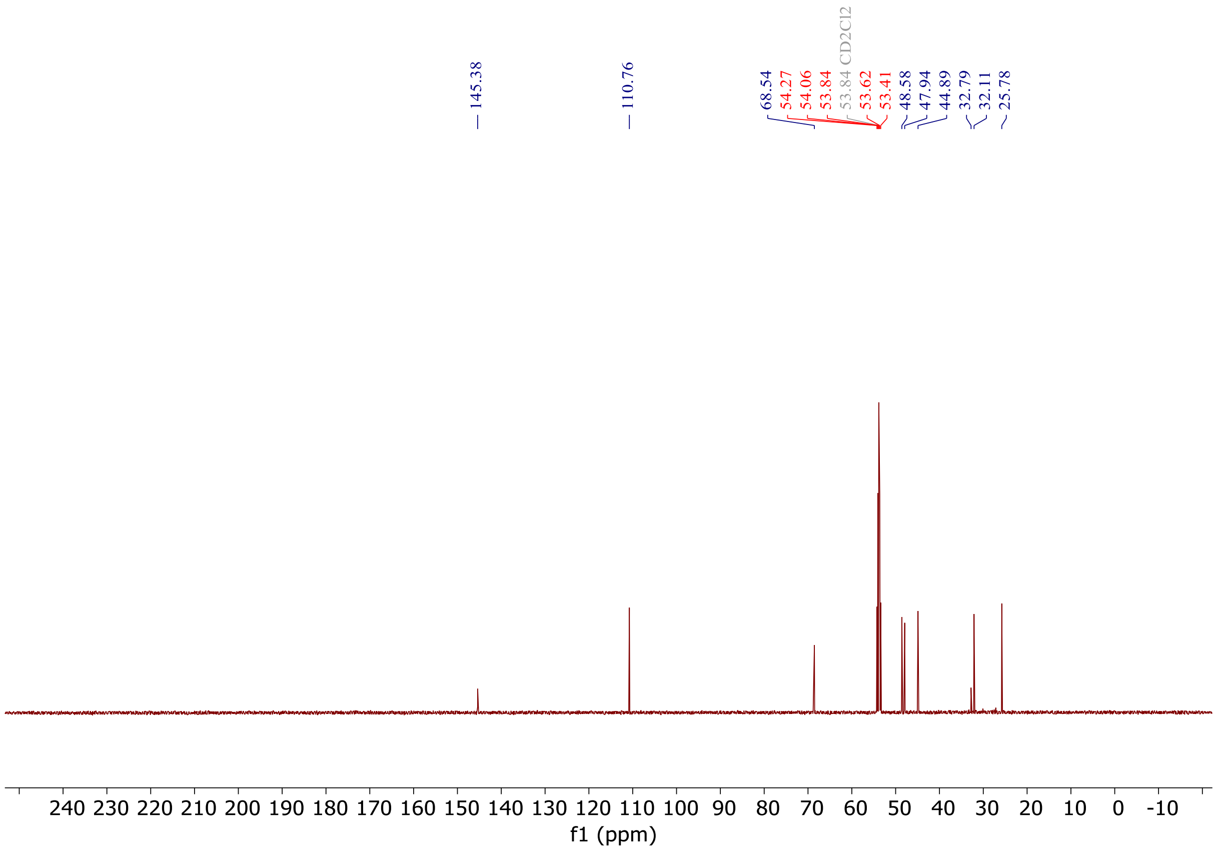

(*R*)-3-methylene-1,2,3,4-tetrahydronaphthalen-1-ol (2v)

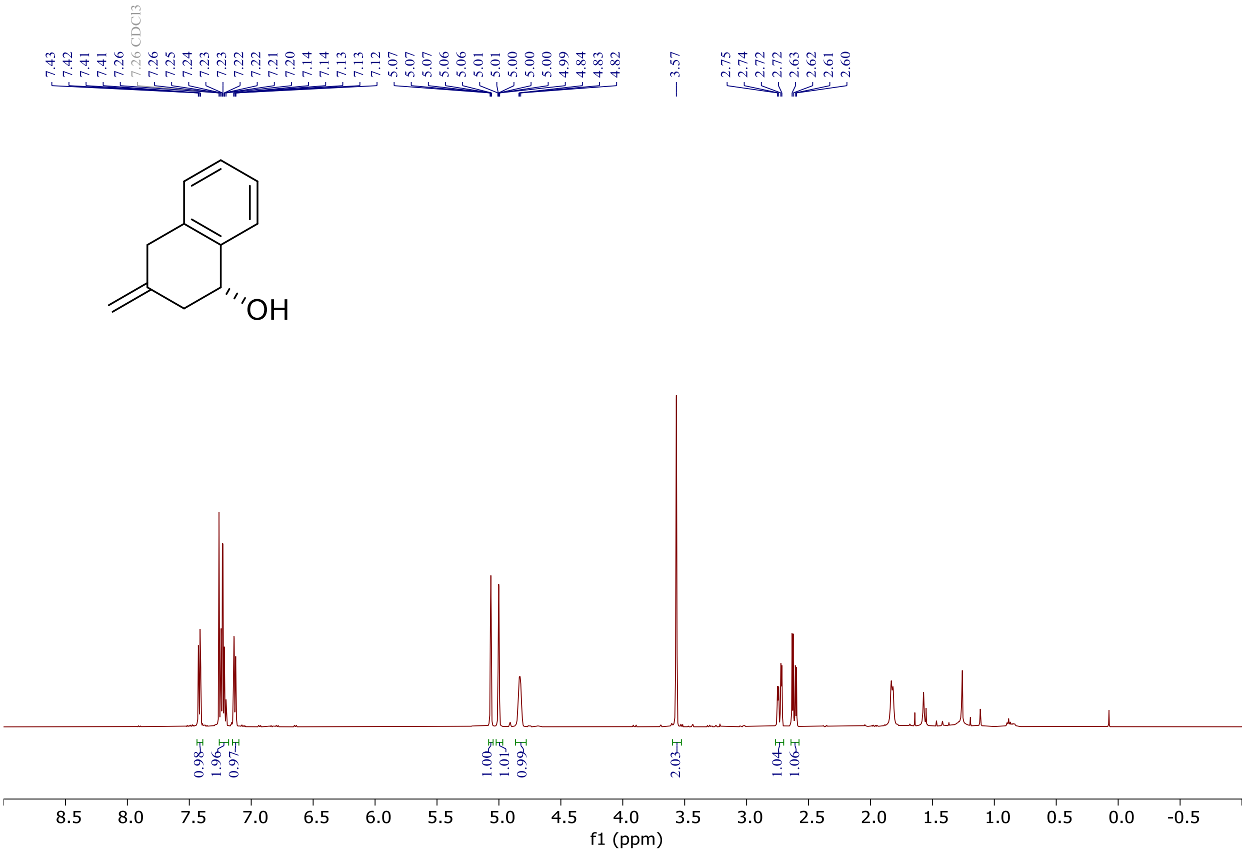

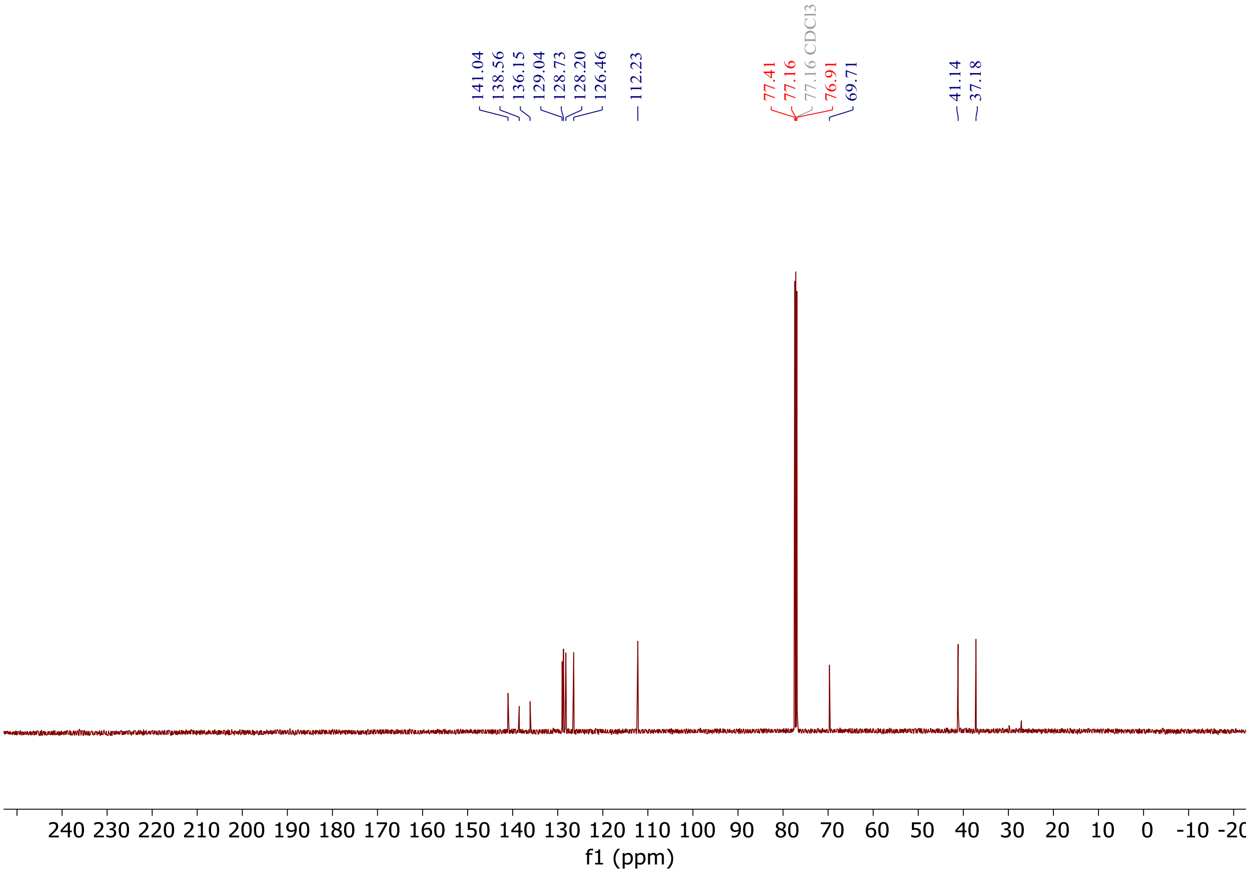

(*S*)-spiro[2.6]nonan-5-ol (8)

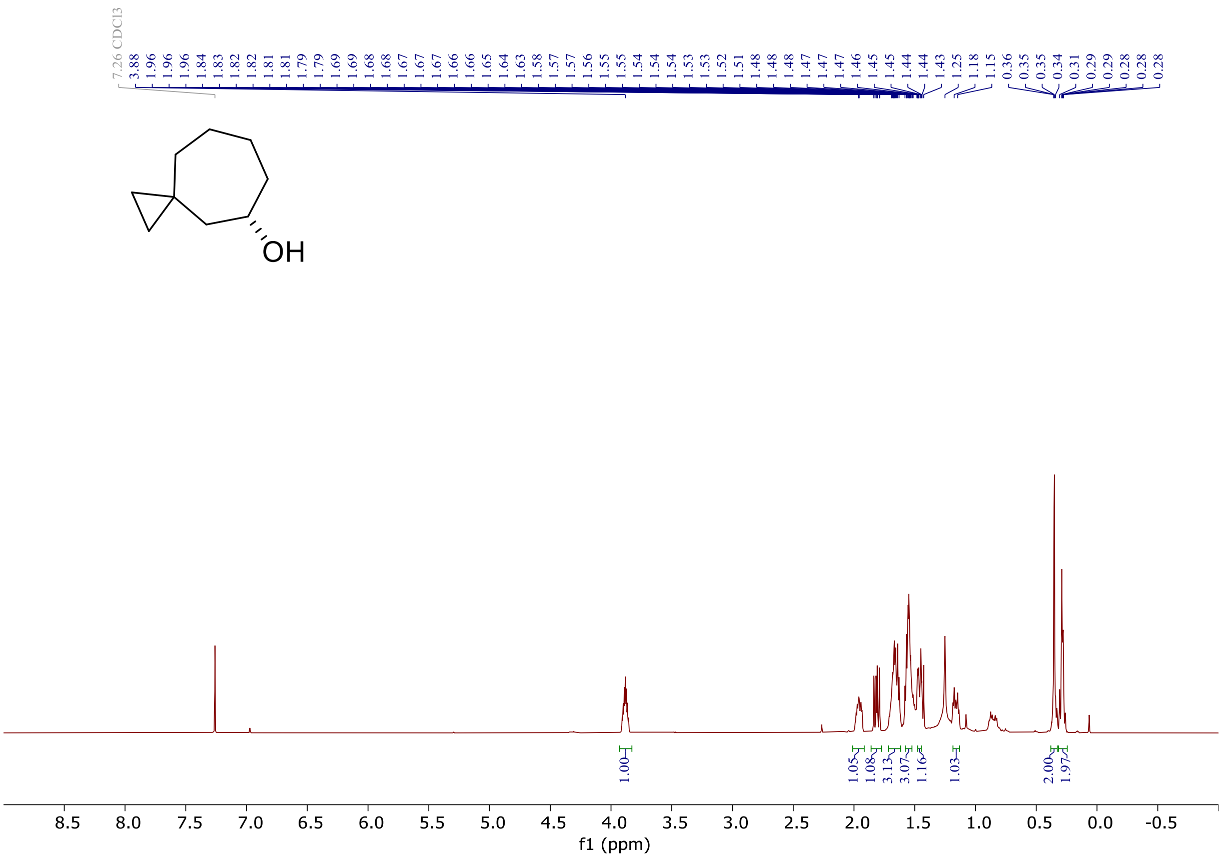

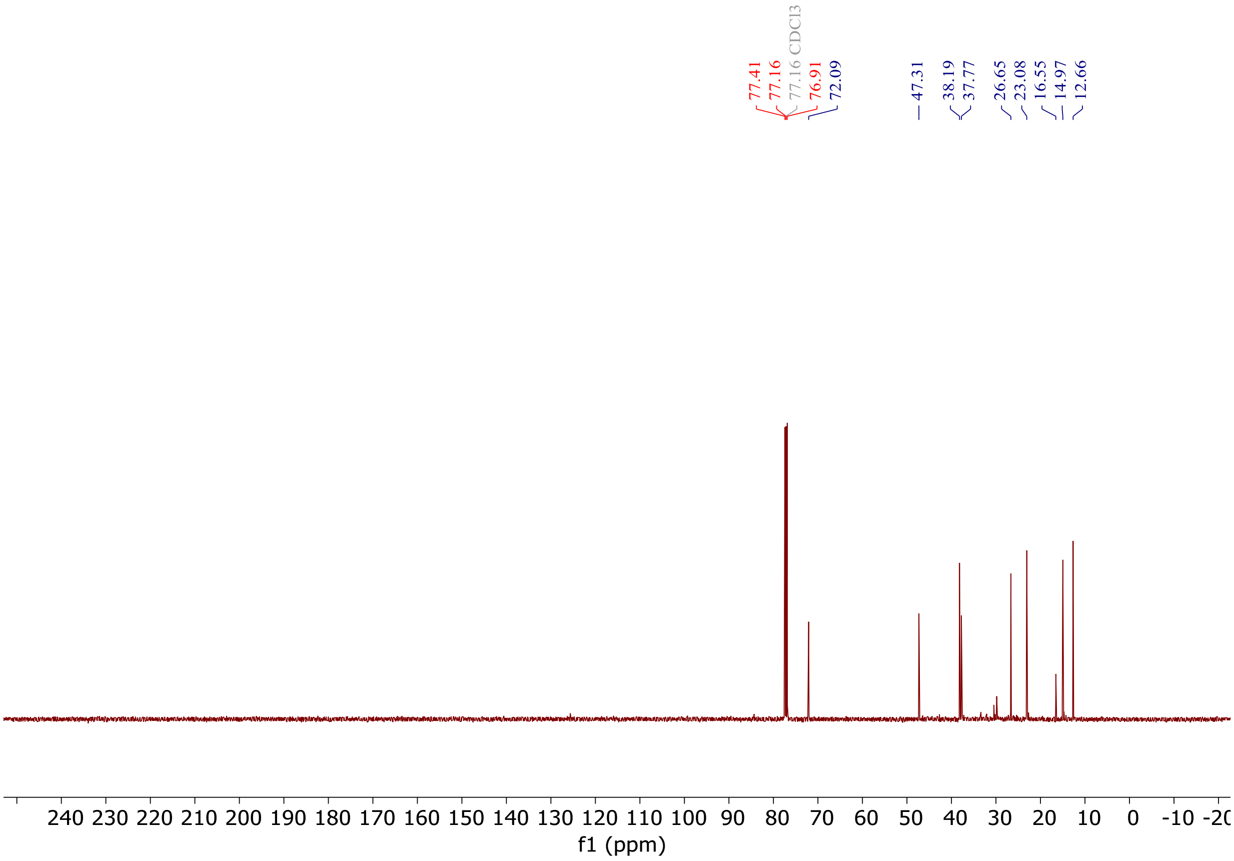

(*S*,*E*)-3-(2-phenylethylidene)cyclohexyl ferrocenecarboxylate (9)

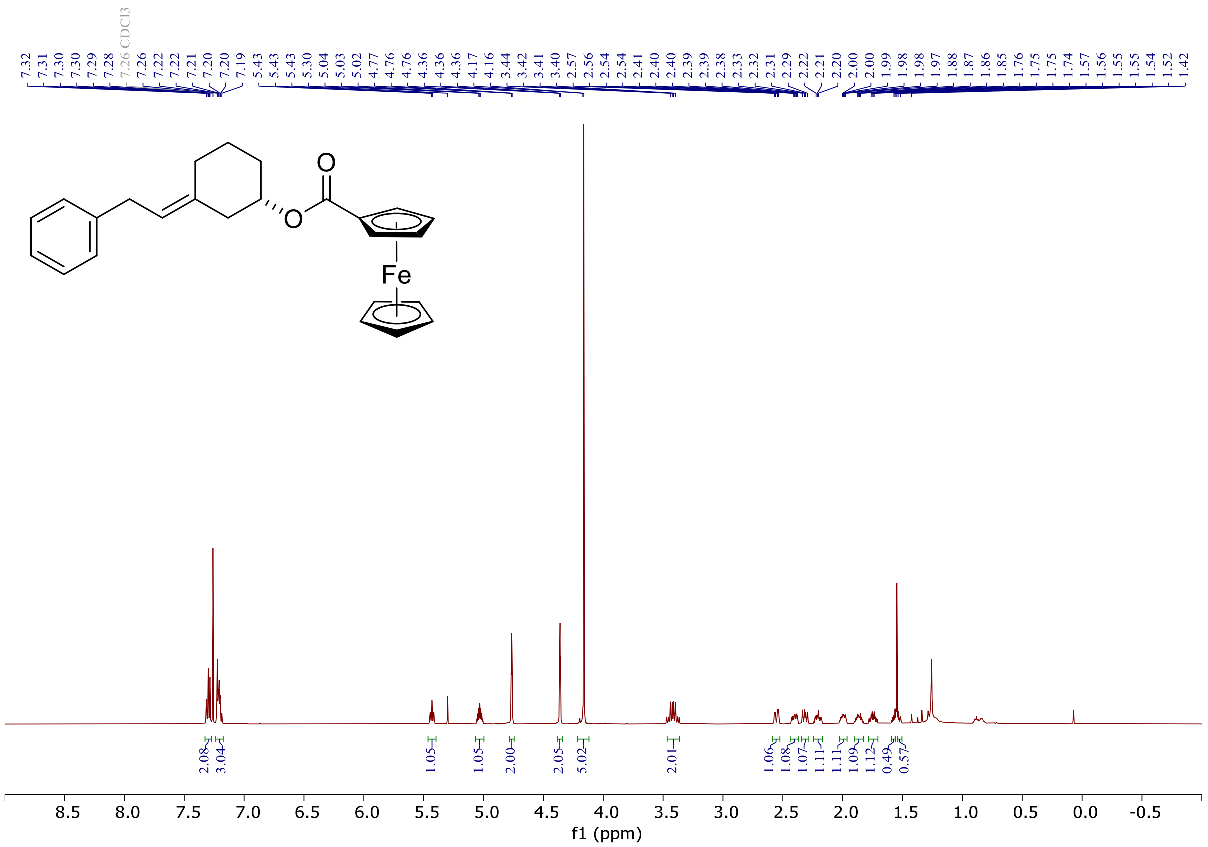

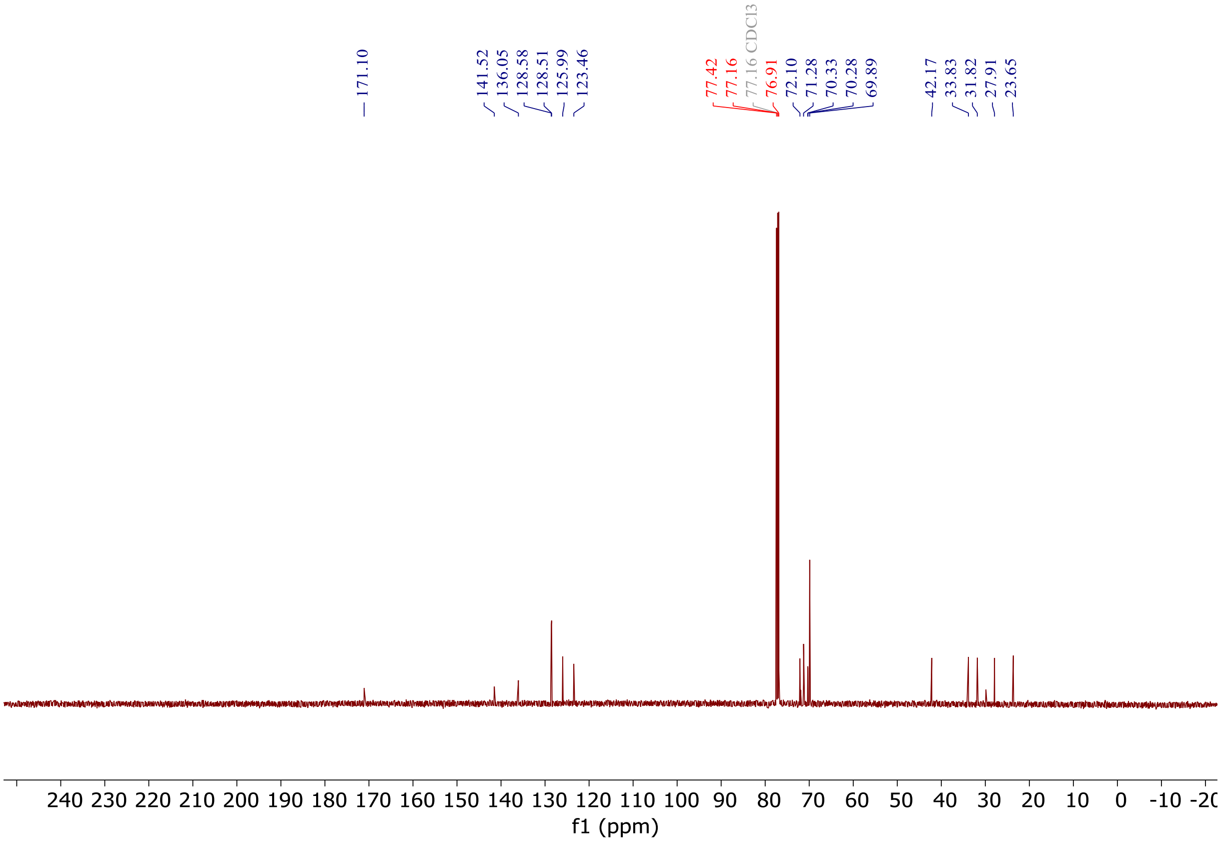

(1*S*,3*S*)-3-butylcycloheptan-1-ol (10)

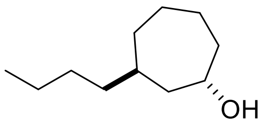

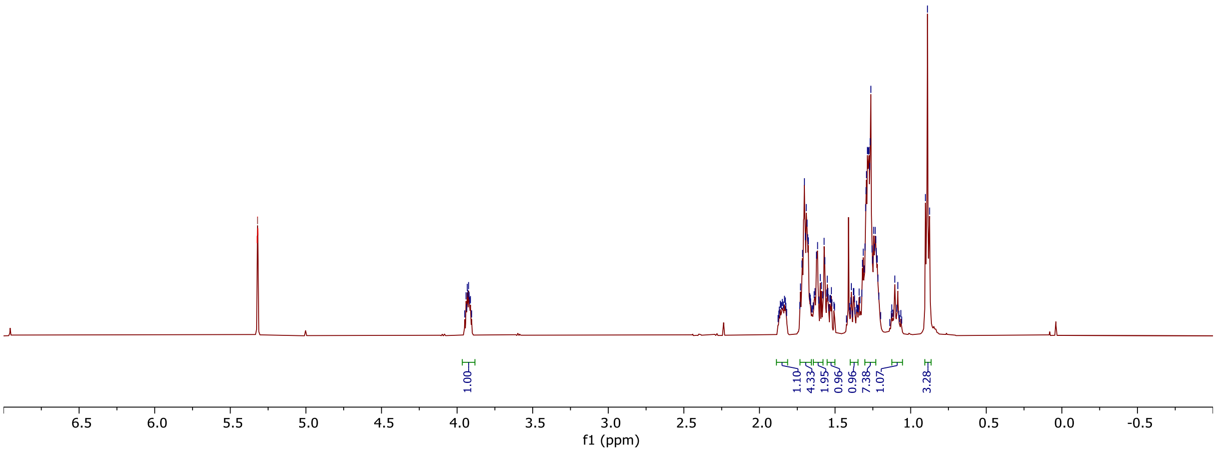

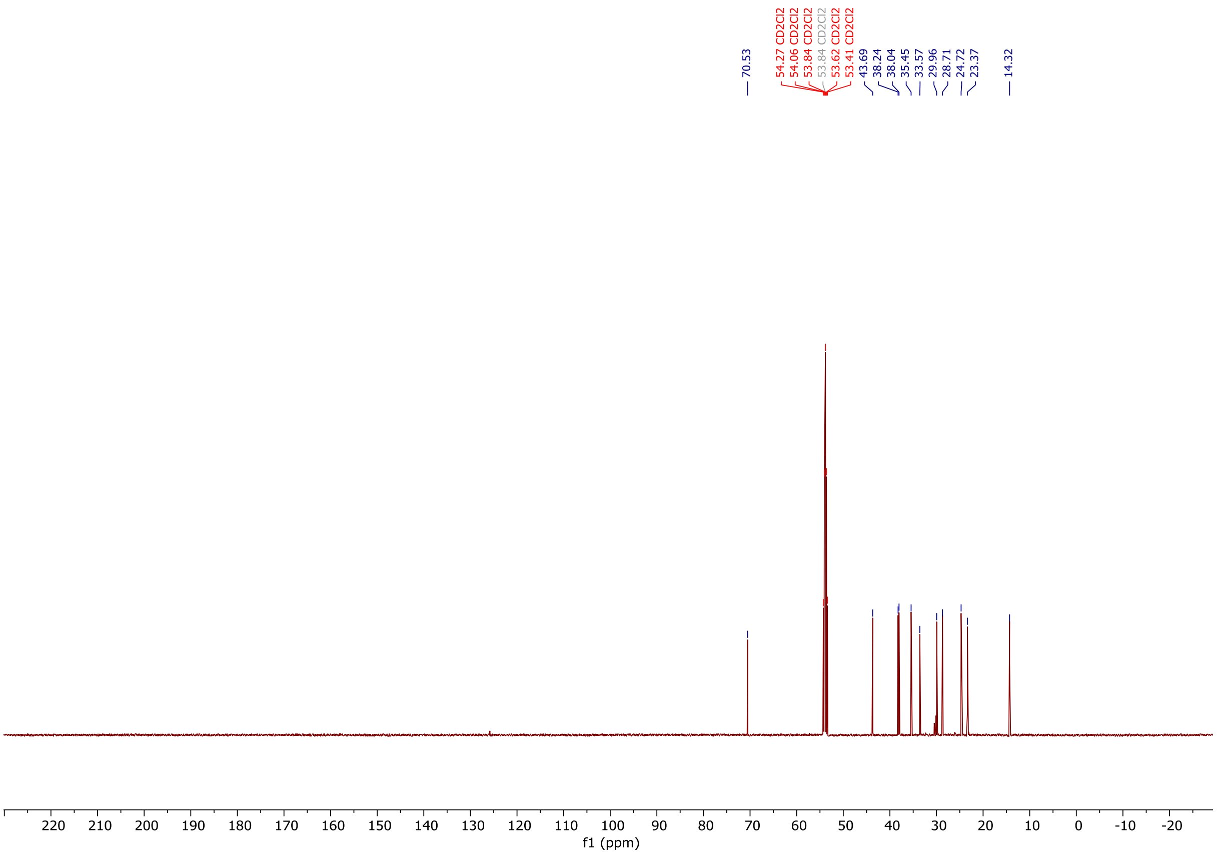

12. GC and HPLC Traces

(*S*)-3-methylenecyclohexan-1-ol (2a)

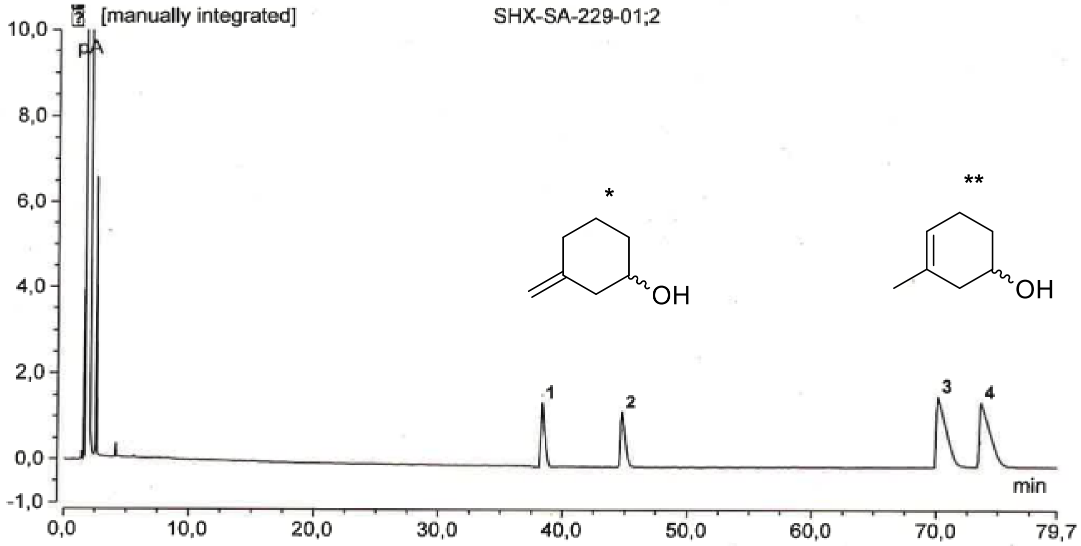

No.	Ret.Time min	Rel.Area %	Peak Name
1	38,32	12,71	*
2	44,72	13,03	*
3	70,09	37,06	**
4	73,54	37,20	**

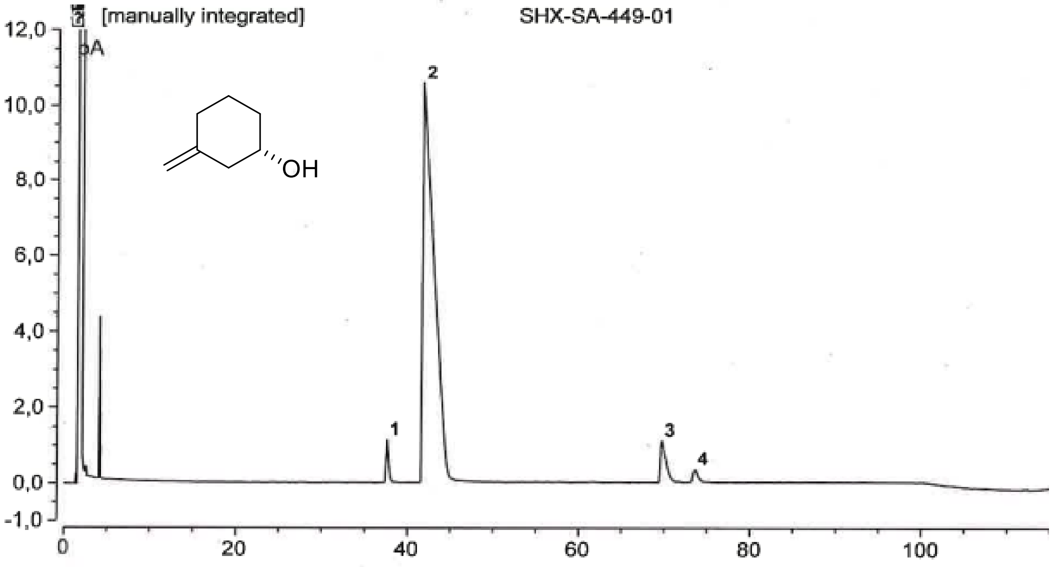

No.	Ret.Time min	Rel.Area %	Peak Name
1	37,57	2,10	*
2	41,61	91,74	*
3	69,64	4,92	**
4	73,52	1,24	**

(*S*,*E*)-3-propylidenecyclohexan-1-ol (2b)

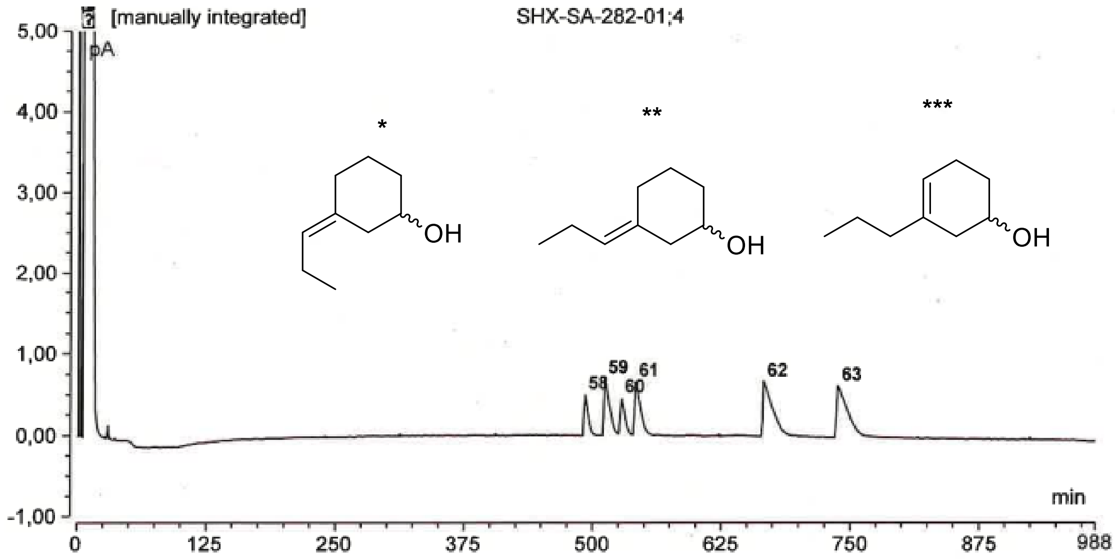

No.	Ret.Time min	Rel.Area %	Peak Name
58	492,39	8,32	*
59	511,27	15,58	**
60	527,77	8,35	*
61	541,57	15,43	**
62	665,74	25,85	***
63	737,56	26,46	***

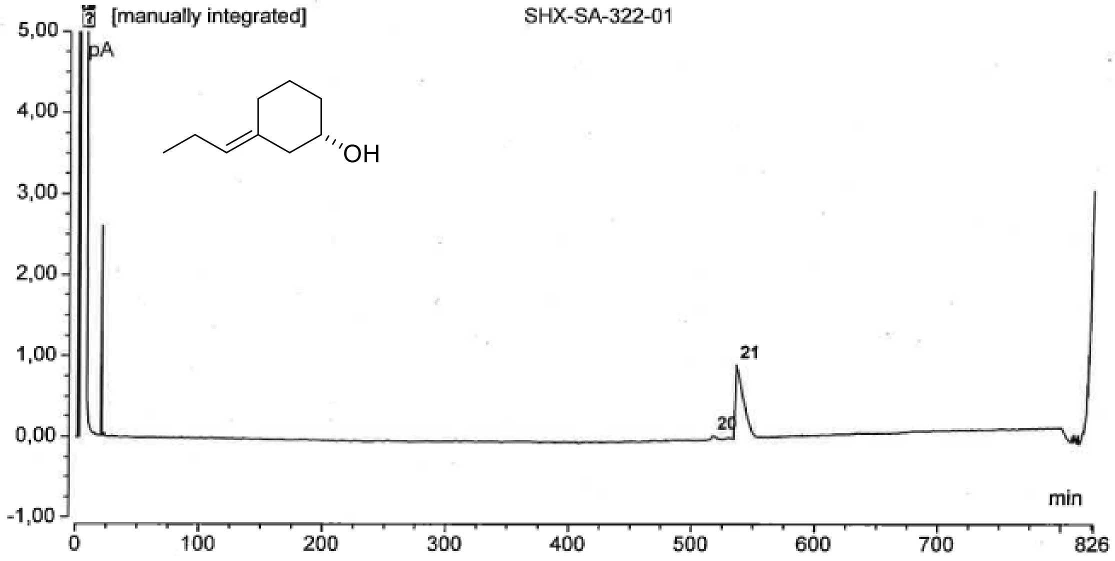

No.	Ret.Time min	Rel.Area %	Peak Name
20	517,69	2,49	**
21	536,07	97,51	**

(*S*,*E*)-3-heptylidenecyclohexan-1-ol (2c)

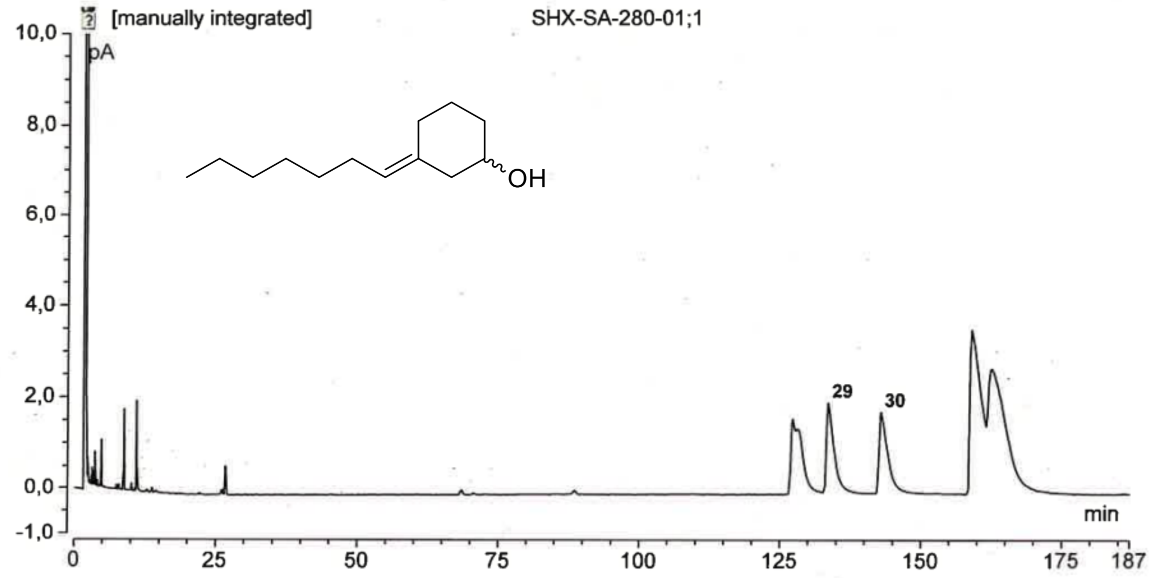

No.	Ret.Time min	Rel.Area %	Peak Name
29	133,47	50,10	**
30	142,80	49,90	**

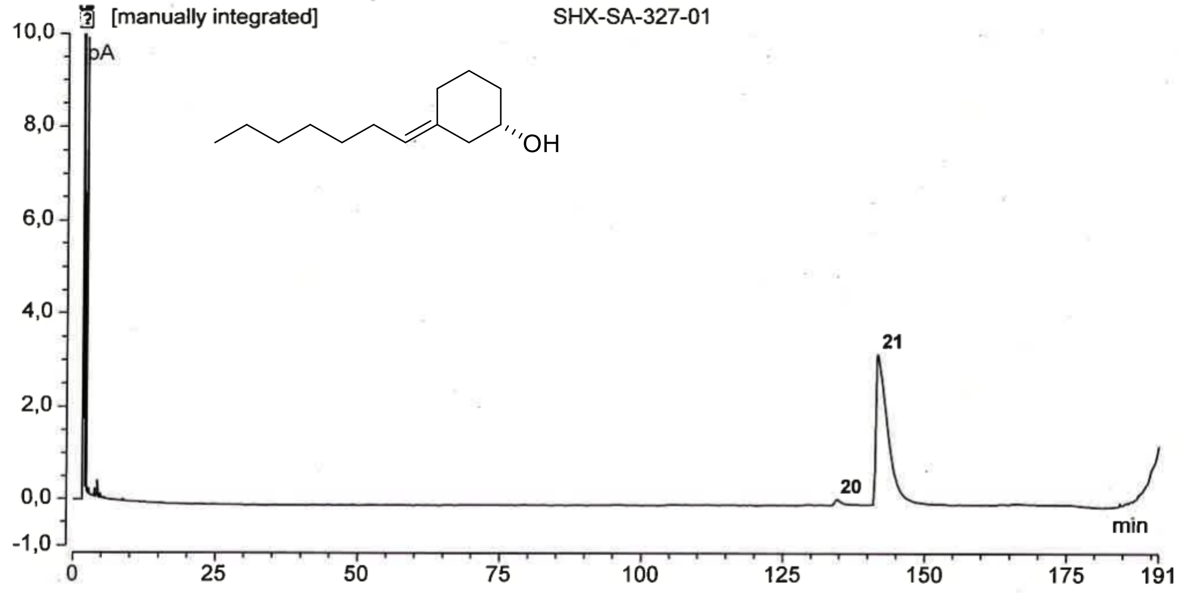

No.	Ret.Time min	Rel.Area %	Peak Name
20	134,46	2,12	**
21	141,56	97,88	**

(*S*,*E*)-3-(3-methylbutylidene)cyclohexan-1-ol (2d)

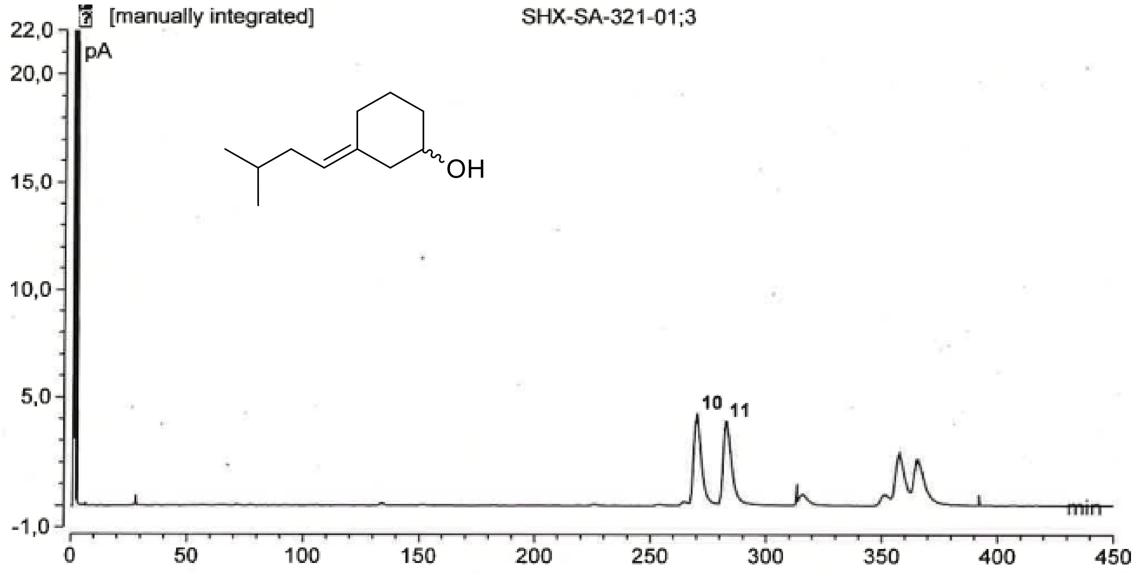

No.	Ret.Time min	Rel.Area %	Peak Name
10	270,11	49,84	.
11	282,44	50,16	.

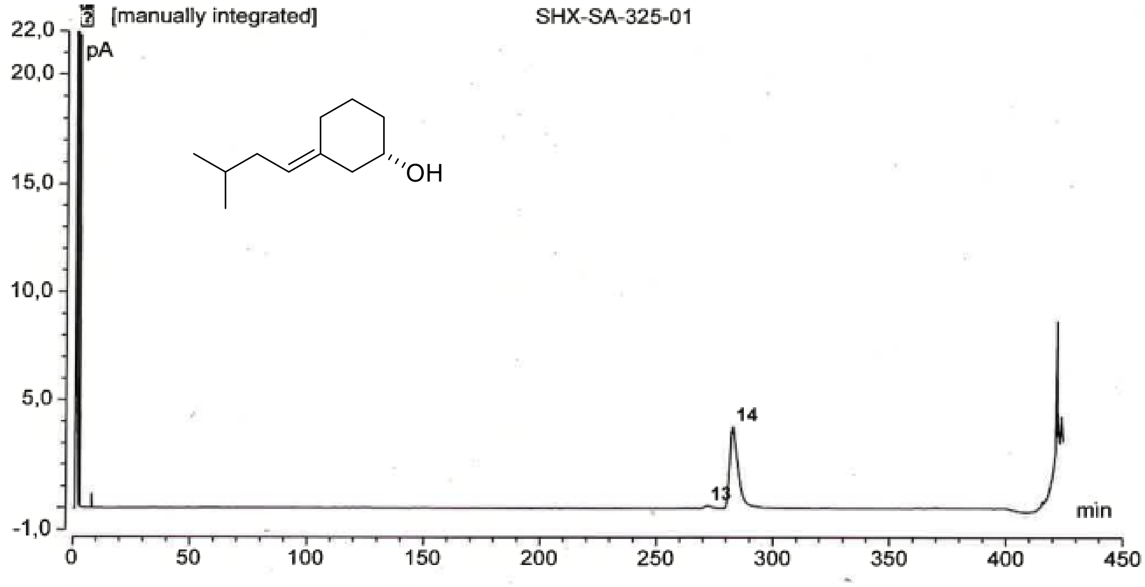

No.	Ret.Time min	Rel.Area %	Peak Name
13	271,67	2,85	.
14	282,46	97,15	.

(*S*,*E*)-3-(but-3-en-1-ylidene)cyclohexan-1-ol (2e)

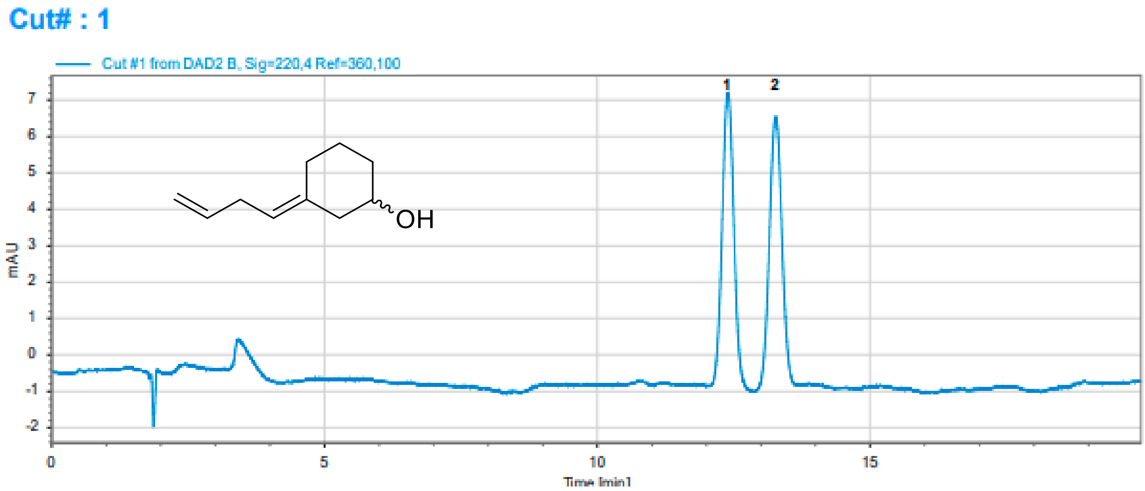

Component table

Signal: DAD2 B, Sig=220,4 Ref=360,100

chiral

Component	¹D Sampling range [min]	Ret.Time ²D [min]	Area	Area%	
1	15.44 - 15.48	12.395	122.065	50.369	1st enantiomer
2	15.44 - 15.48	13.268	120.276	49.631	2nd enantiomer

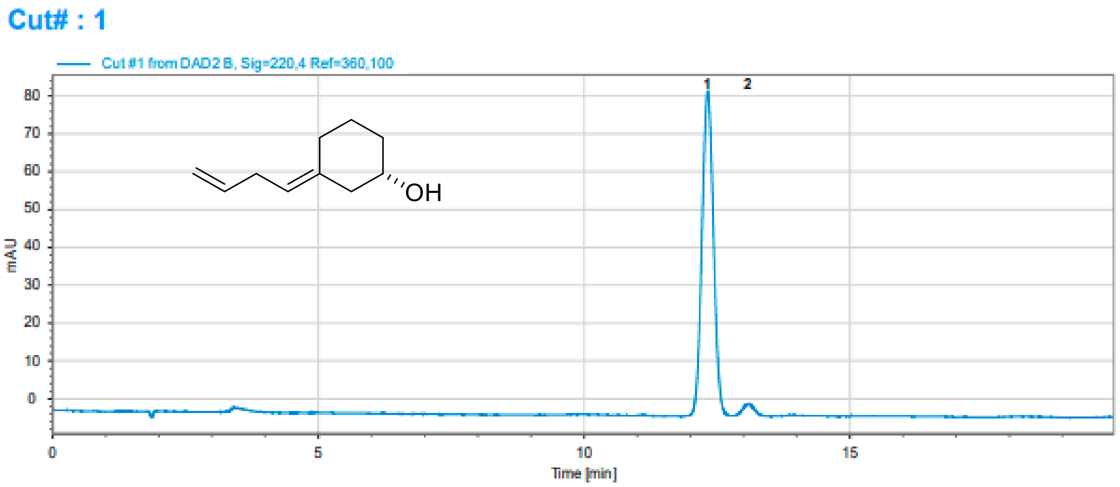

Component table

Signal: DAD2 B, Sig=220,4 Ref=360,100

chiral

Component	¹D Sampling range [min]	Ret.Time ²D [min]	Area	Area%	
1	15.47 - 15.51	12.325	1286.034	96.326	1st enantiomer
2	15.47 - 15.51	13.090	49.058	3.674	2nd enantiomer

= 92.7 % ee

(*S*,*E*)-3-(pent-4-en-1-ylidene)cyclohexan-1-ol (2f)

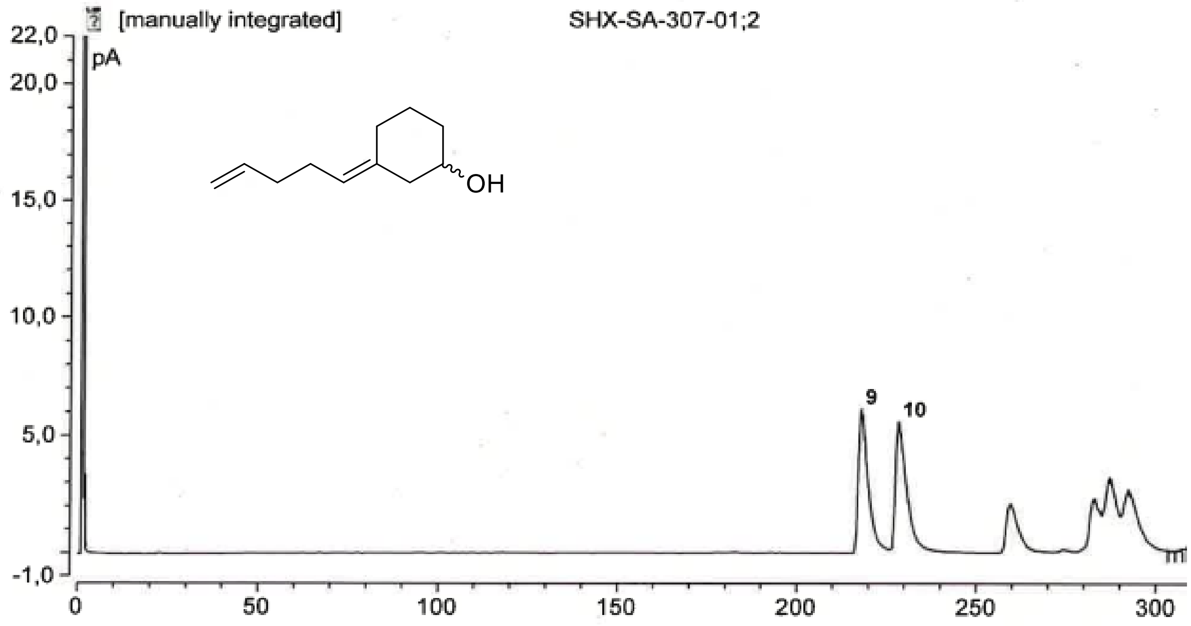

No.	Ret.Time min	Rel.Area %	Peak Name
9	217,84	49,59 .	
10	228,27	50,41 .	

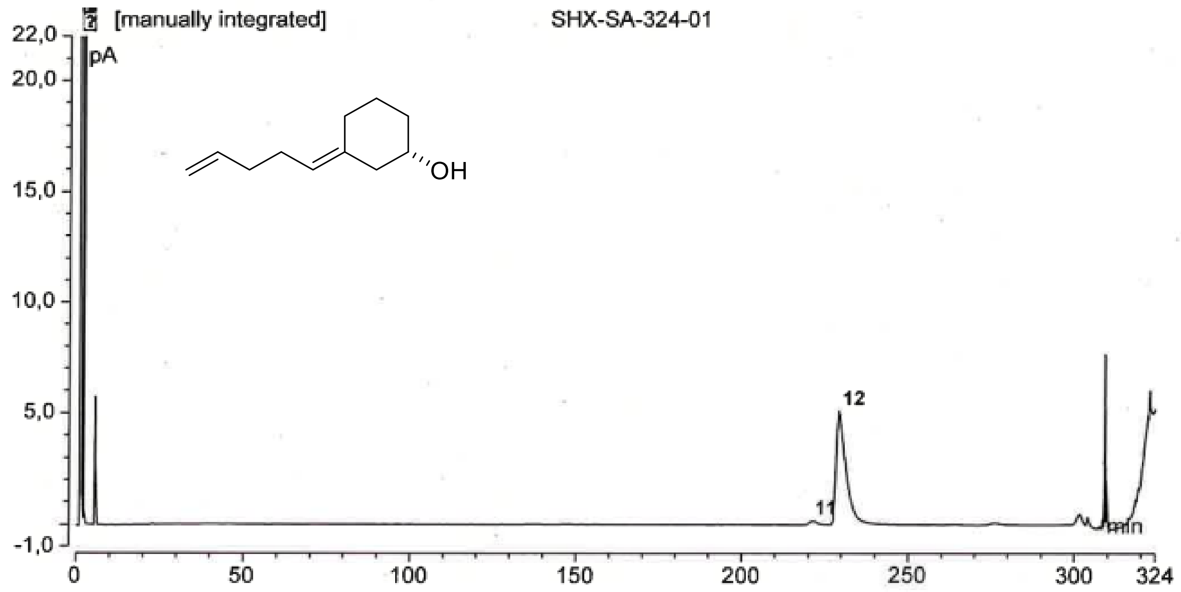

No.	Ret.Time min	Rel.Area %	Peak Name
11	221,14	3,12 .	
12	229,11	96,88 .	

(*S*,*E*)-3-(2-phenylethylidene)cyclohexan-1-ol (2g)

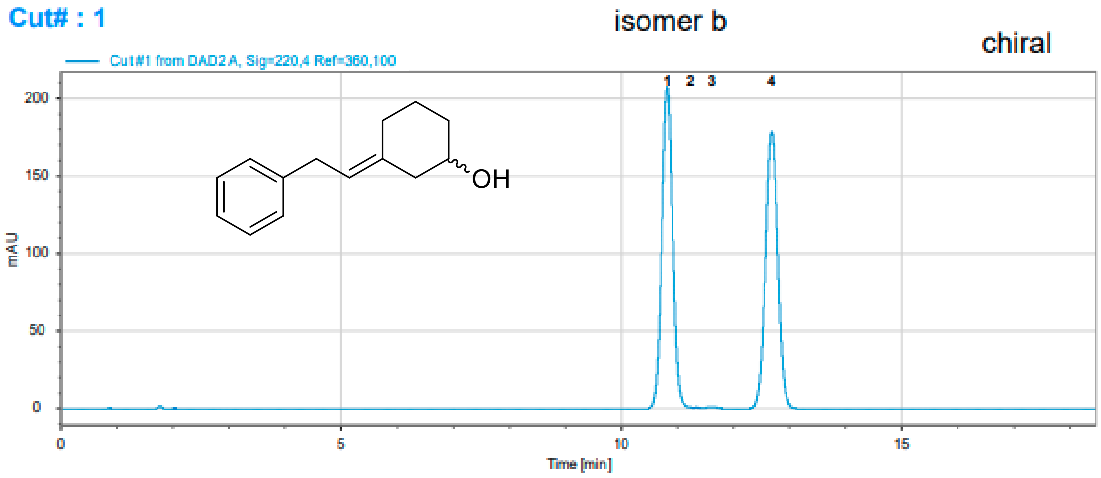

Compound	Cut	Ret.Time	Area	Width	Height	Symmetry
1	1	10.825	2779.540 **2779.540**	0.223	207.299	0.971
2	1	11.240	13.285 **13.285**	0.146	1.068	0.000
3	1	11.625	20.823 **20.823**	0.252	1.379	0.825
4	1	12.688	2751.701 **2751.701**	0.236	178.782	0.972

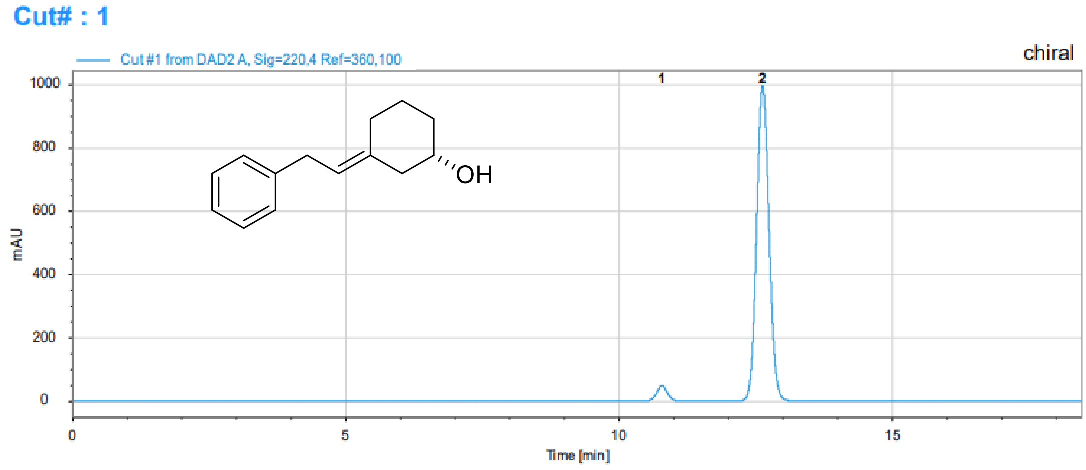

Component	^{1}D Sampling range [min]	Ret.Time ^{2}D [min]	Area	Area%
1	9.45 - 9.49	10.791	635.317	3.890
2	9.45 - 9.49	12.632	15695.502	96.110

ee = 92.2%

(*S*,*E*)-3-(2-(*p*-tolyl)ethylidene)cyclohexan-1-ol (2h)

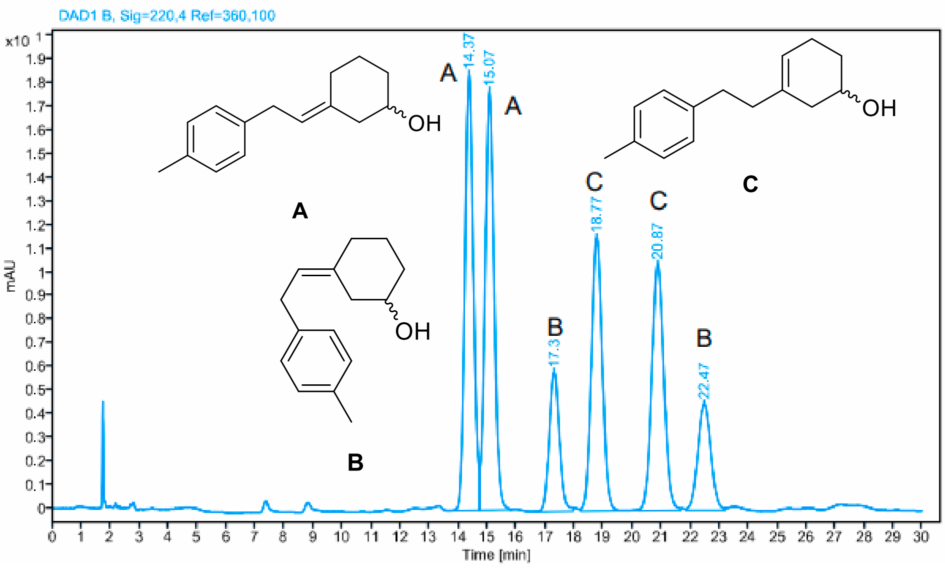

Signal: DAD1 B, Sig=220,4 Ref=360,100

RT [min]	Area%	Name
14.37	22.79	A 1st enantiomer
15.07	23.27	A 2nd enantiomer
17.30	8.68	B 1st enantiomer
18.77	18.27	C 1st enantiomer
20.87	18.47	C 2nd enantiomer
22.47	8.51	B 2nd enantiomer

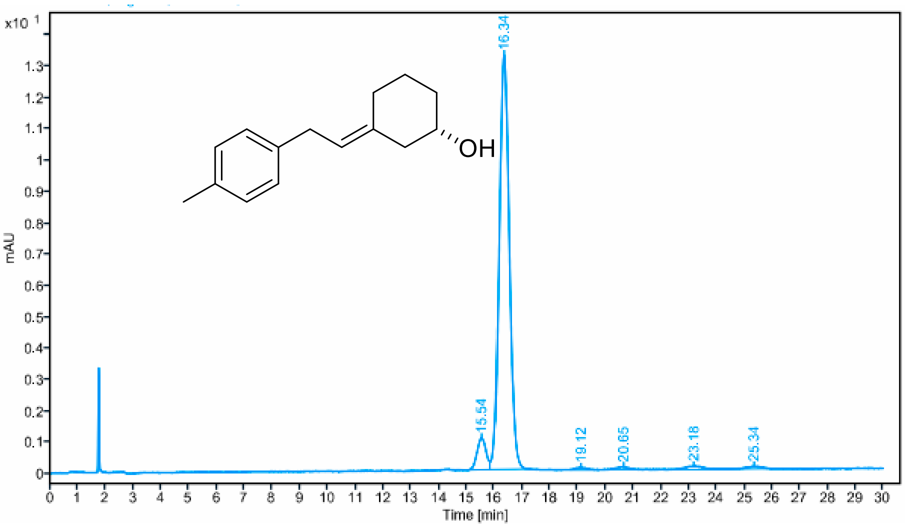

Signal: DAD1 B, Sig=220,4 Ref=360,100

RT [min]	Area%	Name
15.54	6.20	Diast. A - 1st enantiomer
16.34	90.29	Diast. A - 2nd enantiomer
19.12	0.68	Diast. B - 1st enantiomer
20.65	0.62	Diast. C - 1st enantiomer
23.18	1.36	Diast. C - 2nd enantiomer
25.34	0.85	Diast. B - 2nd enantiomer

= 87.1 % ee

(*S*)-3-(propan-2-ylidene)cyclohexan-1-ol (2i)

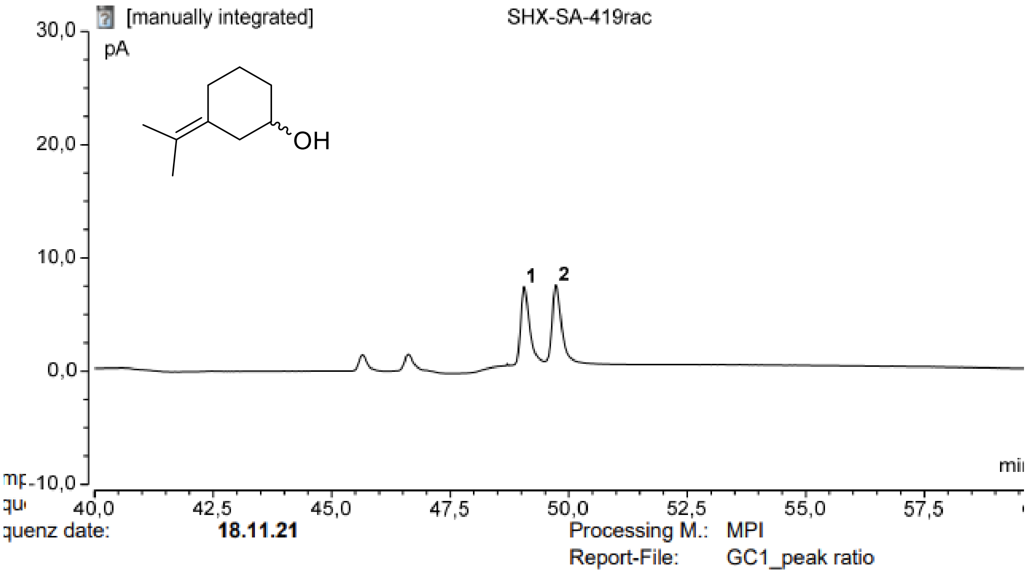

No.	Ret.Time min	Rel.Area %	Peak Name
1	49,06	49,83	Component 35
2	49,73	50,17	Component 36

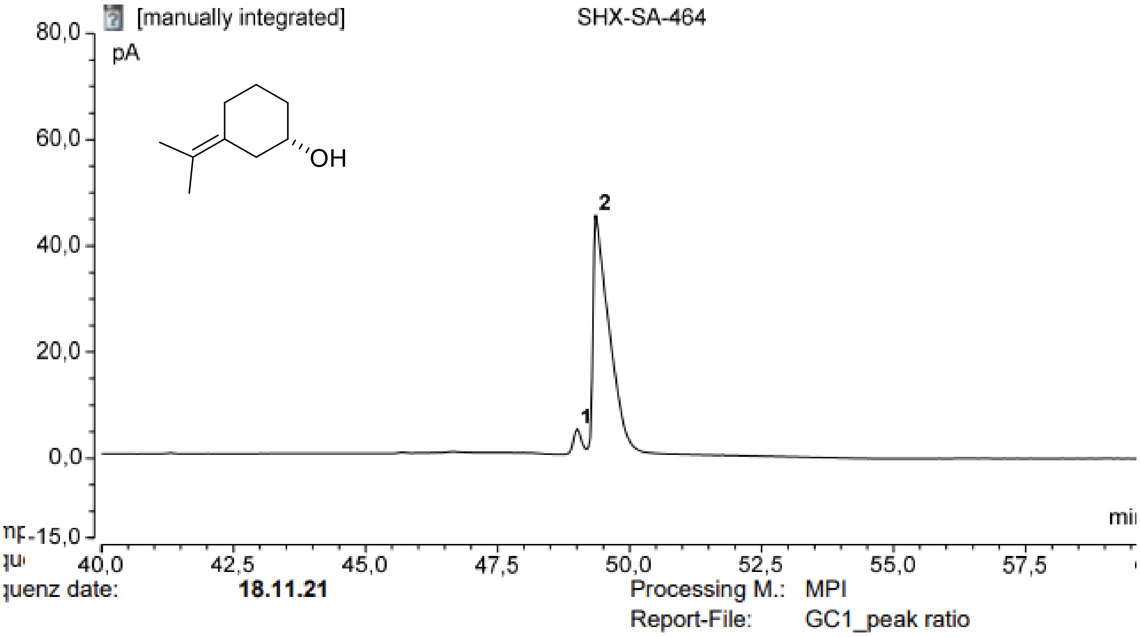

No.	Ret.Time min	Rel.Area %	Peak Name
1	49,01	4,75	Component 35
2	49,36	95,25	Component 36

(*S*)-3-cyclopentylidenecyclohexan-1-ol (2j)

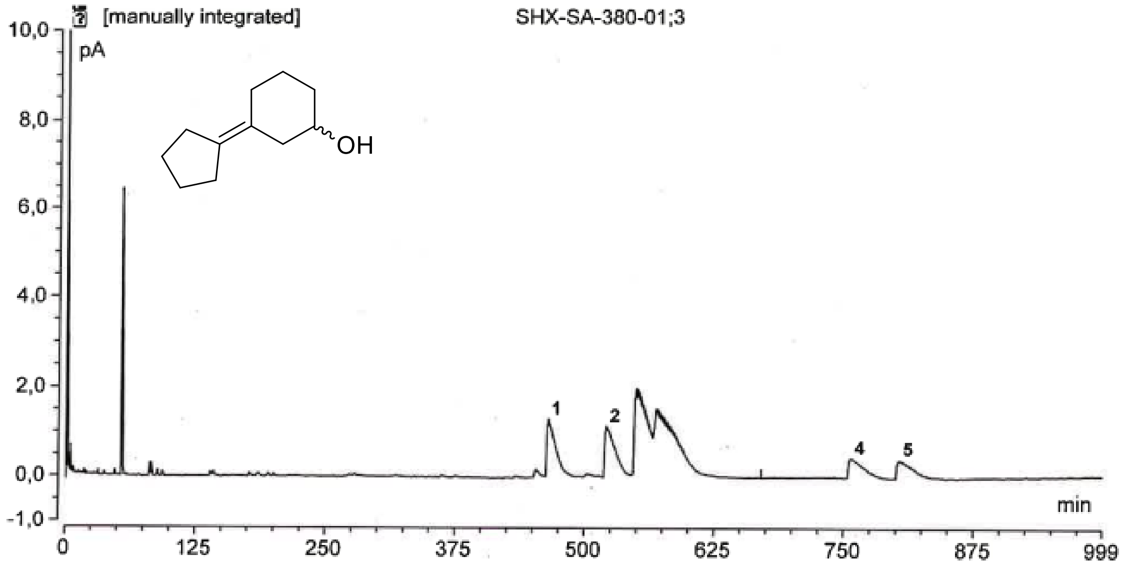

No.	Ret.Time min	Rel.Area %	Peak Name
1	464,77	32,00	***
2	520,88	33,31	***
4	757,05	17,60	**
5	803,66	17,08	**

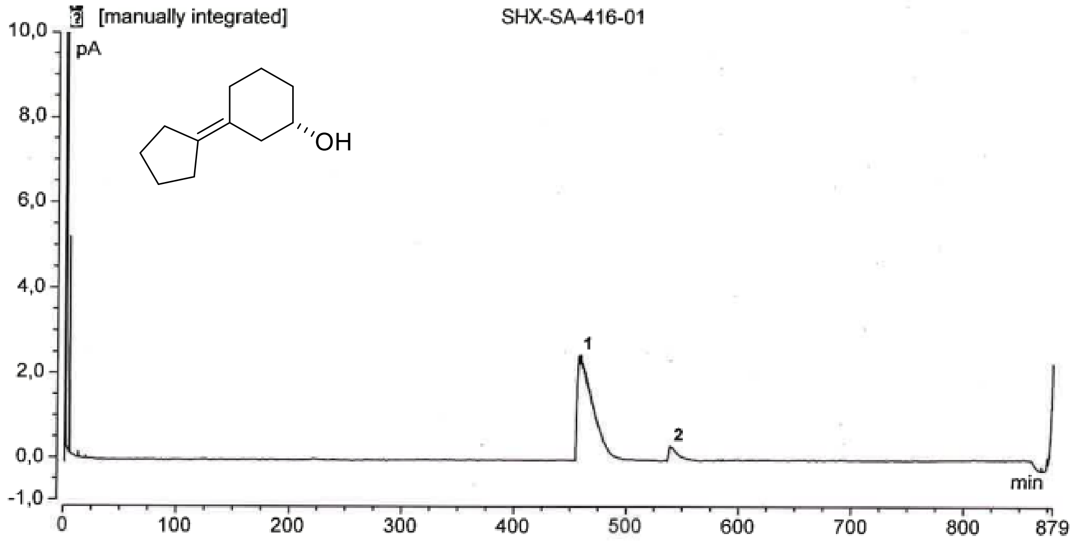

No.	Ret.Time min	Rel.Area %	Peak Name
1	458,24	91,74	***
2	539,02	8,26	***

(*S*)-[1,1'-bi(cyclohexylidene)]-3-ol (2k)

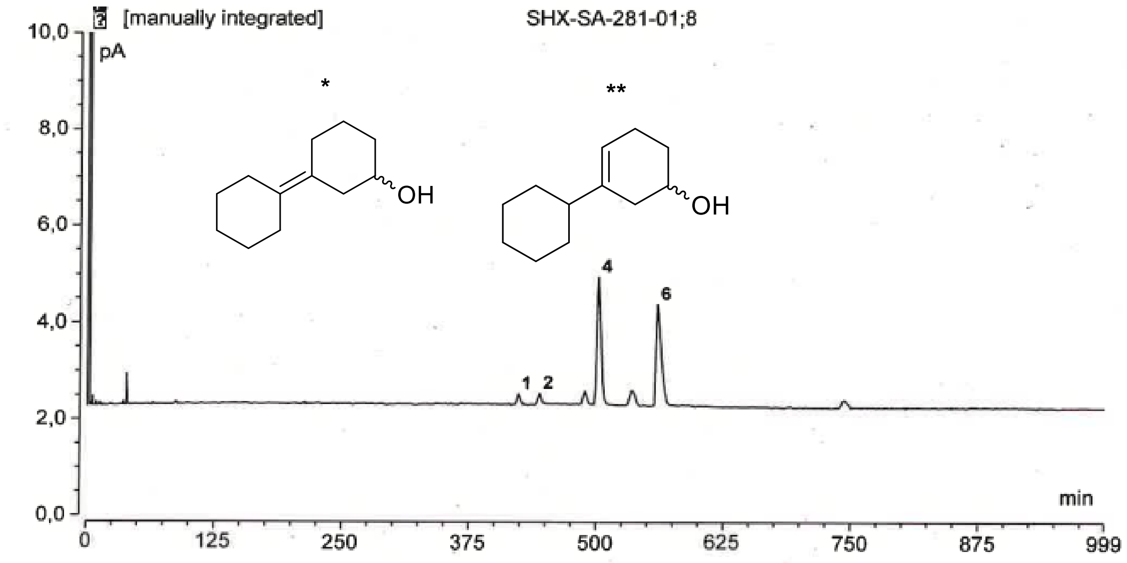

No.	Ret.Time min	Rel.Area %	Peak Name
1	422,91	3,35	*
2	443,79	3,52	*
4	501,38	46,65	**
6	559,10	46,48	**

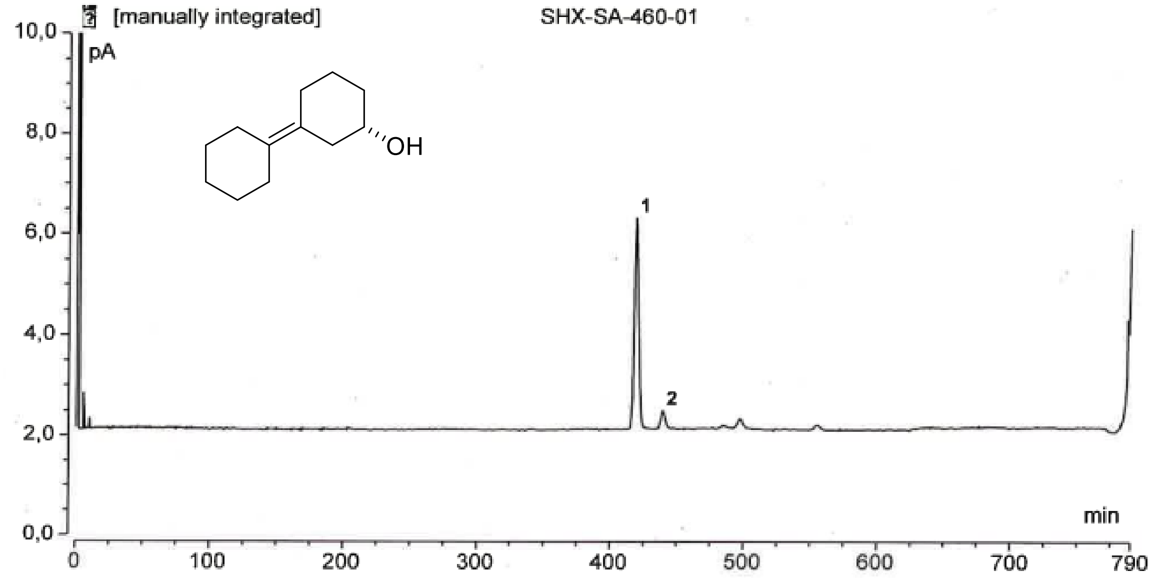

No.	Ret.Time min	Rel.Area %	Peak Name
1	418,67	91,04	*
2	439,26	8,96	*

(*S*,*E*)-3-(2-methylpropylidene)cyclohexan-1-ol (2l)

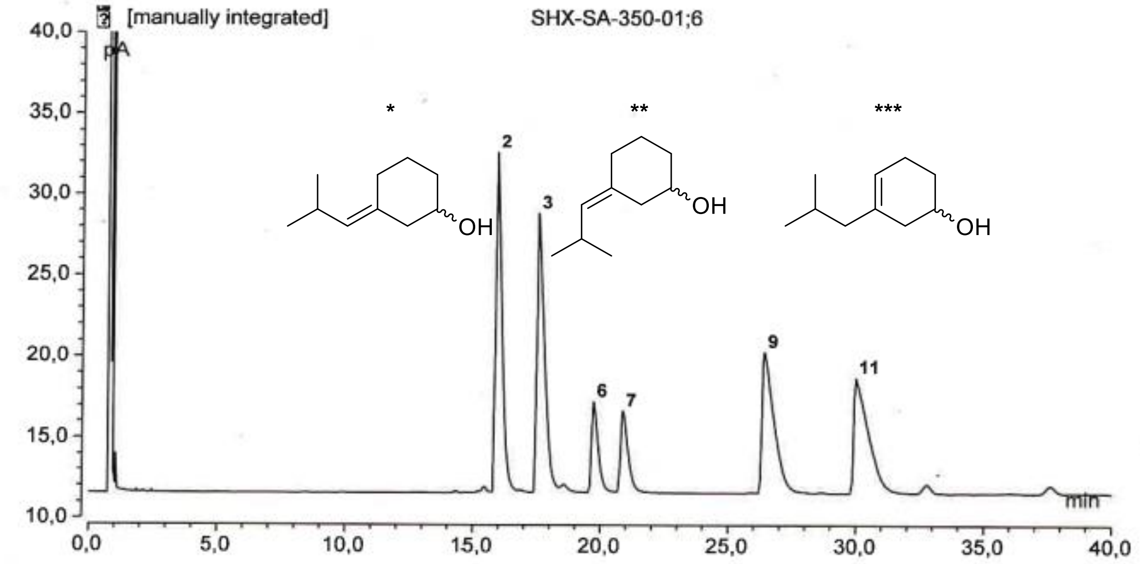

No.	Ret.Time min	Rel.Area %	Peak Name
2	15,88	23,66	*
3	17,51	24,03	*
6	19,71	6,91	**
7	20,85	6,98	**
9	26,37	19,18	***
11	29,97	19,23	***

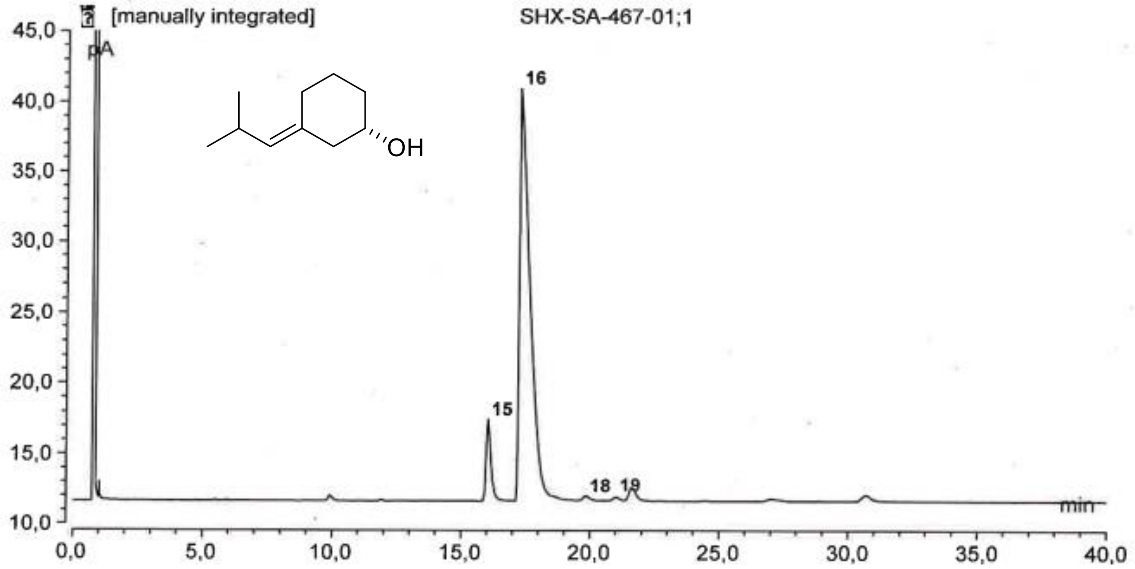

No.	Ret.Time min	Rel.Area %	Peak Name
15	16,03	8,16	*
16	17,22	90,62	*
18	19,85	0,68	**
19	21,02	0,54	**

(*S*,*E*)-3-(cyclohexylmethylene)cyclohexan-1-ol (2m)

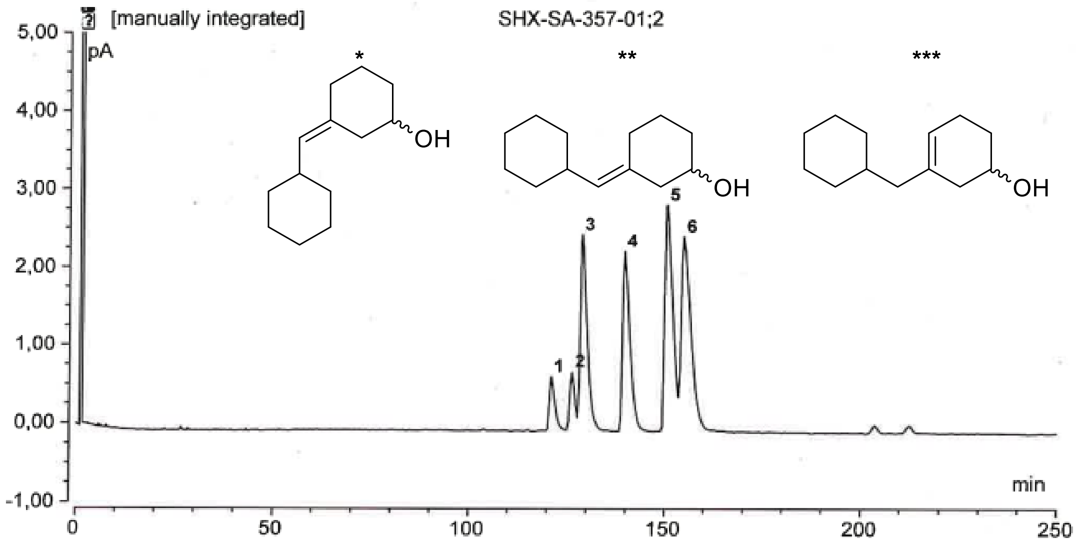

No.	Ret.Time min	Rel.Area %	Peak Name
1	121,10	4,35	*
2	126,26	4,12	*
3	128,75	19,61	**
4	139,59	19,18	**
5	150,39	25,39	***
6	154,72	27,35	***

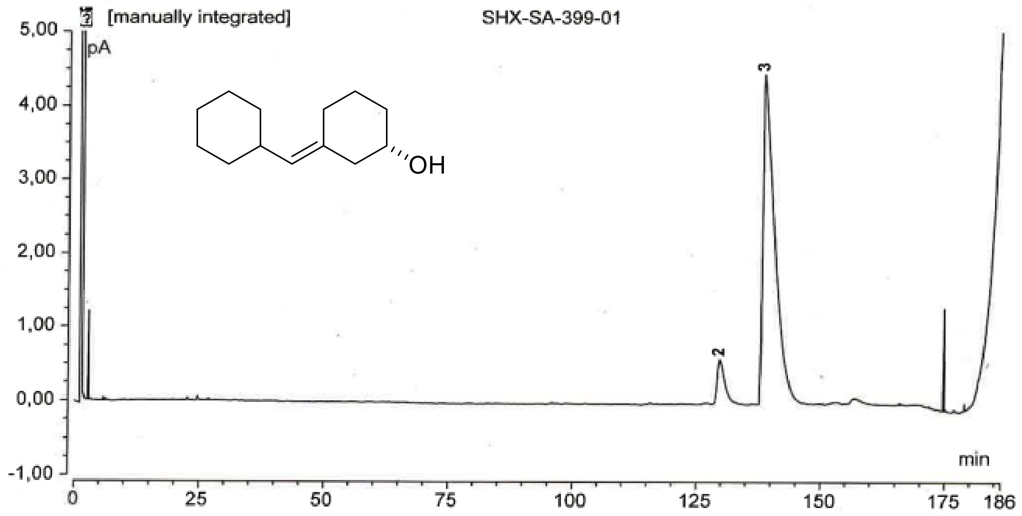

No.	Ret.Time min	Rel.Area %	Peak Name
2	129,69	8,16	**
3	138,41	91,84	**

(*S*,*E*)-3-(3-methoxypropylidene)cyclohexan-1-ol (2n)

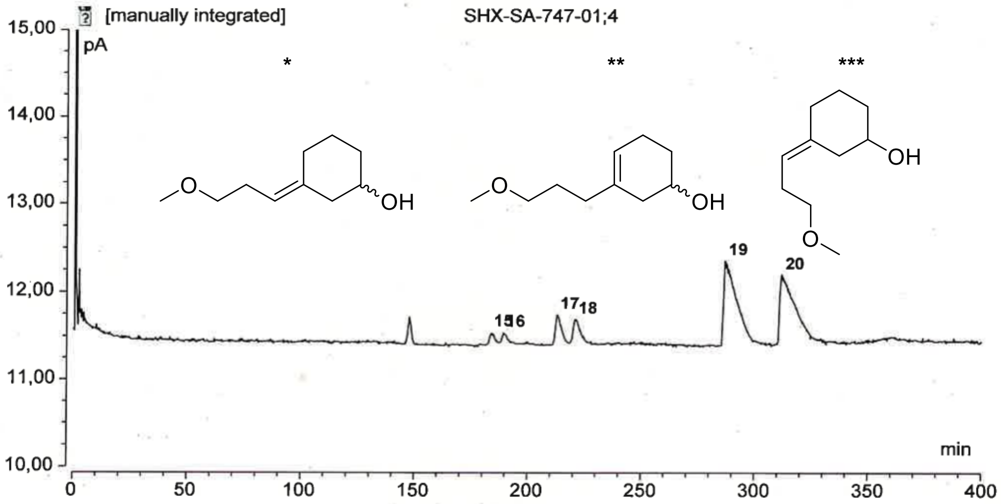

No.	Ret.Time min	Rel.Area %	Peak Name
15	183,91	2,22	***
16	189,28	2,29	***
17	213,02	6,55	*
18	221,06	6,21	*
19	286,98	42,02	**
20	311,78	40,71	**

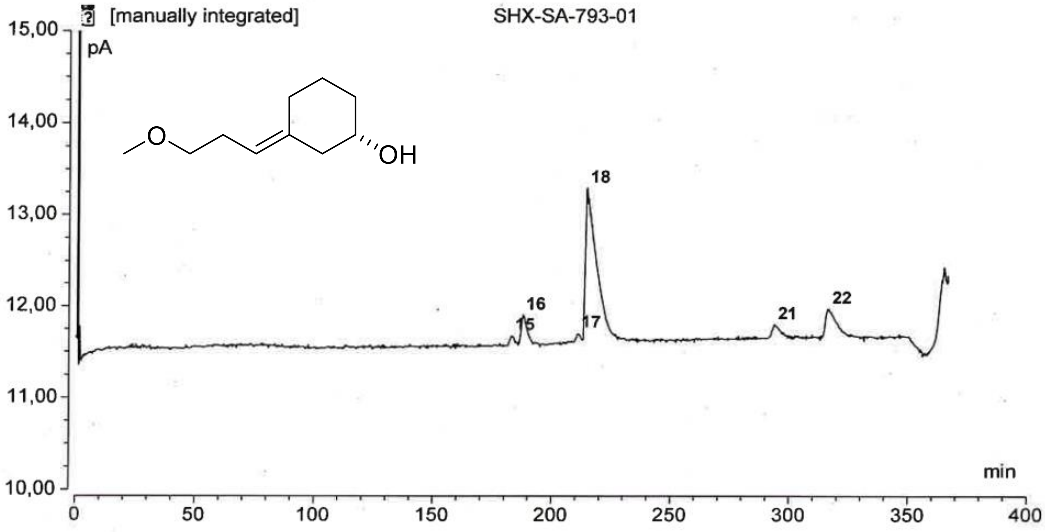

No.	Ret.Time min	Rel.Area %	Peak Name
15	183,33	1,75	***
16	187,85	7,14	***
17	210,97	1,33	*
18	214,60	72,63	*
21	293,60	3,83	**
22	316,36	13,33	**

(*S*,*E*)-3-(4-(thiophen-2-yl)butylidene)cyclohexan-1-ol (2o)

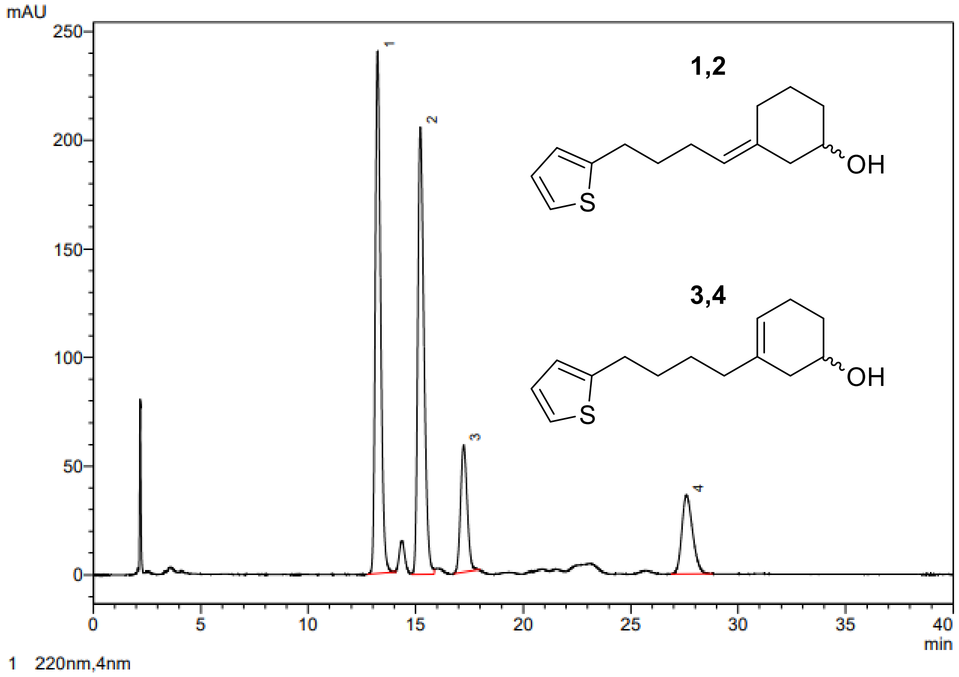

1 220nm,4nm

PDA Ch2 220nm

Peak #	Ret. Time	Area %	Name
1	13,22	38,42	
2	15,22	38,28	
3	17,24	11,62	
4	27,60	11,67	
Total		100,00	

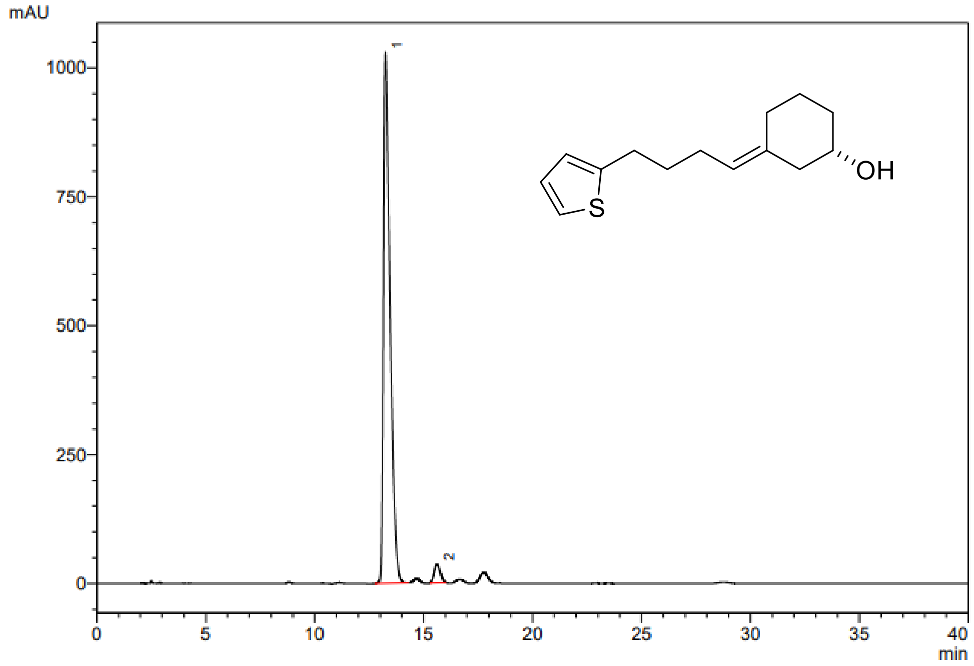

1 220nm,4nm

PDA Ch2 220nm

Peak #	Ret. Time	Area %	Name
1	13,24	96,94	
2	15,62	3,06	
Total		100,00	

(*S*,*E*)-3-(4-hydroxybutylidene)cyclohexan-1-ol (2p)

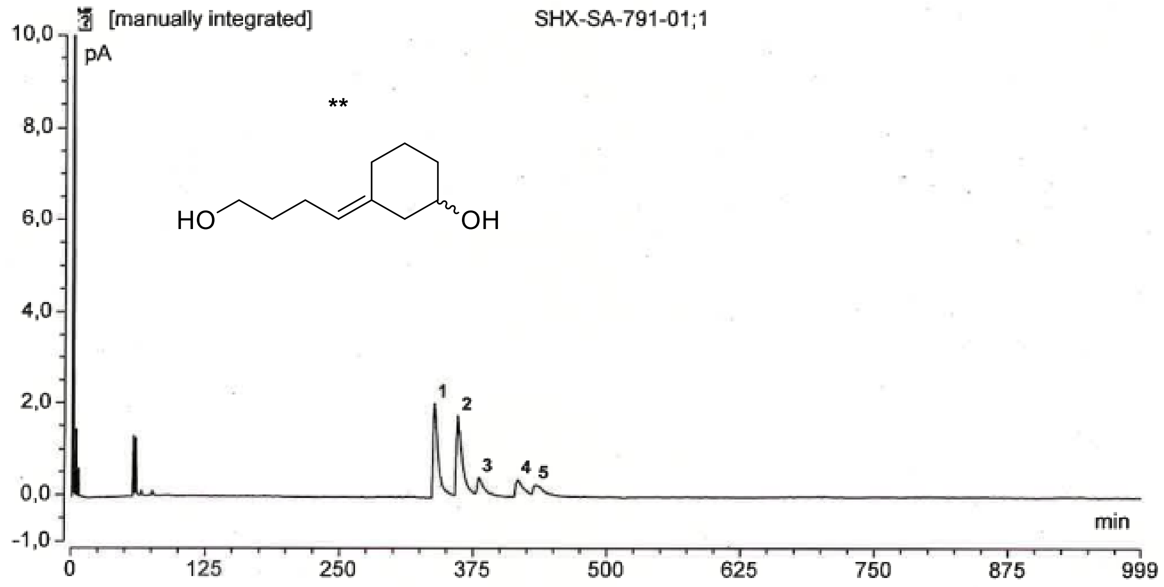

No.	Ret.Time min	Rel.Area %	Peak Name
1	337,97	35,14	**
2	360,24	34,50	**
3	379,93	11,21	***?
4	416,36	9,57	*?
5	432,52	9,58	*?

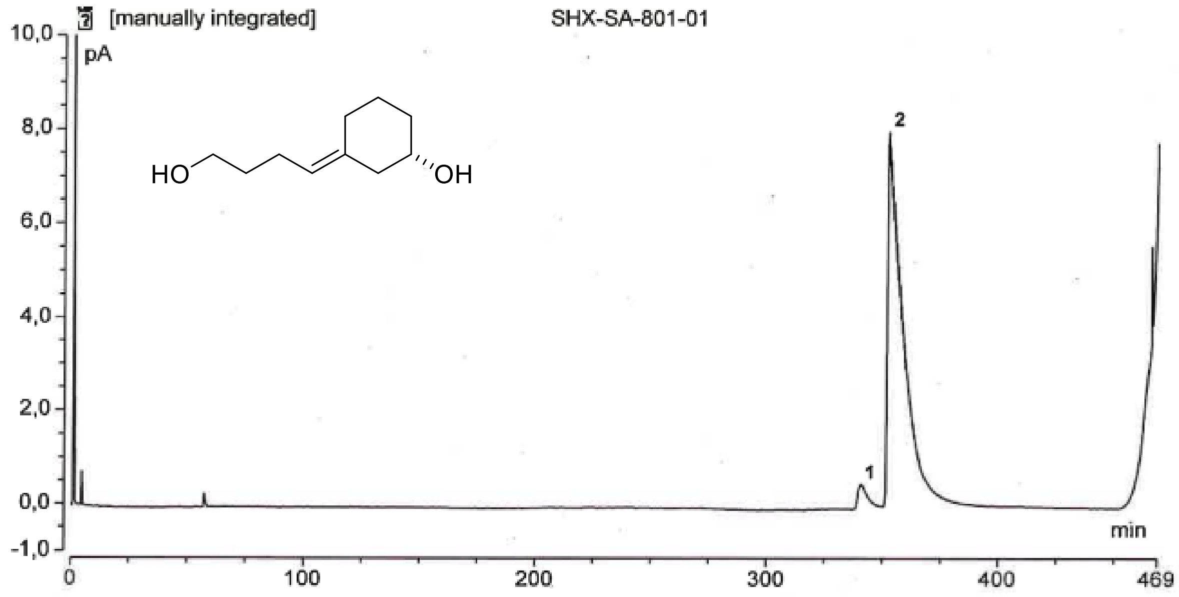

No.	Ret.Time min	Rel.Area %	Peak Name
1	340,77	3,86	**
2	352,53	96,14	**

ethyl (*S*,*E*)-4-(3-hydroxycyclohexylidene)butanoate (2q)

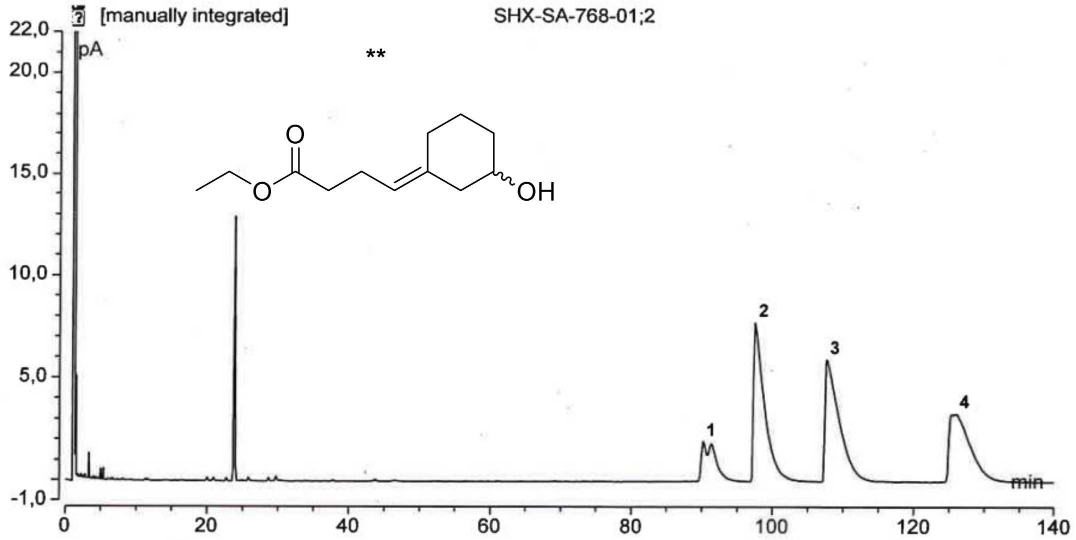

No.	Ret.Time min	Rel.Area %	Peak Name
1	90,16	9,91	*
2	97,45	31,94	**
3	107,57	31,93	**
4	125,96	26,22	***

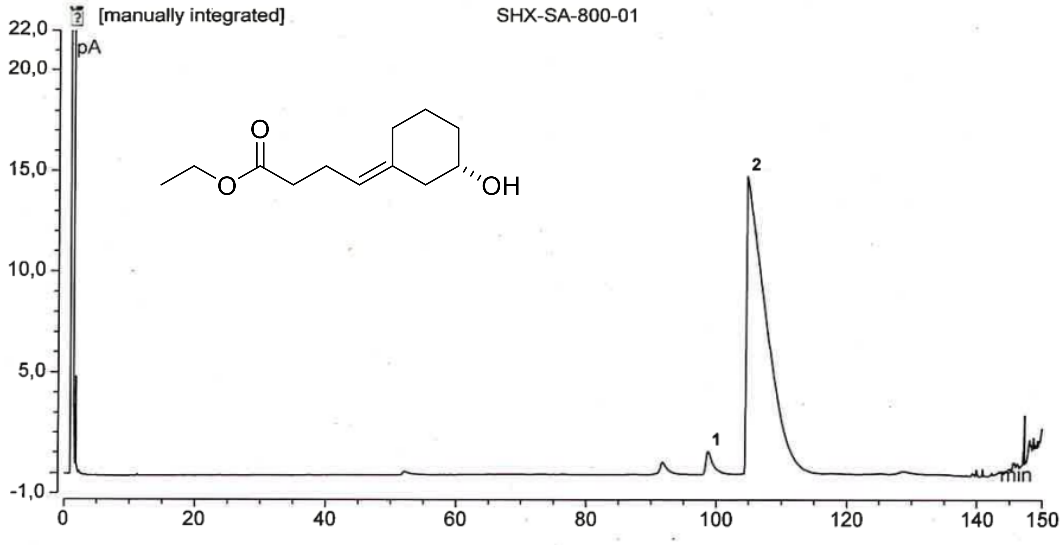

No.	Ret.Time min	Rel.Area %	Peak Name
1	98,69	2,65	**
2	104,56	97,35	**

(*S*,*E*)-3-(4-bromobutylidene)cyclohexan-1-ol (2r)

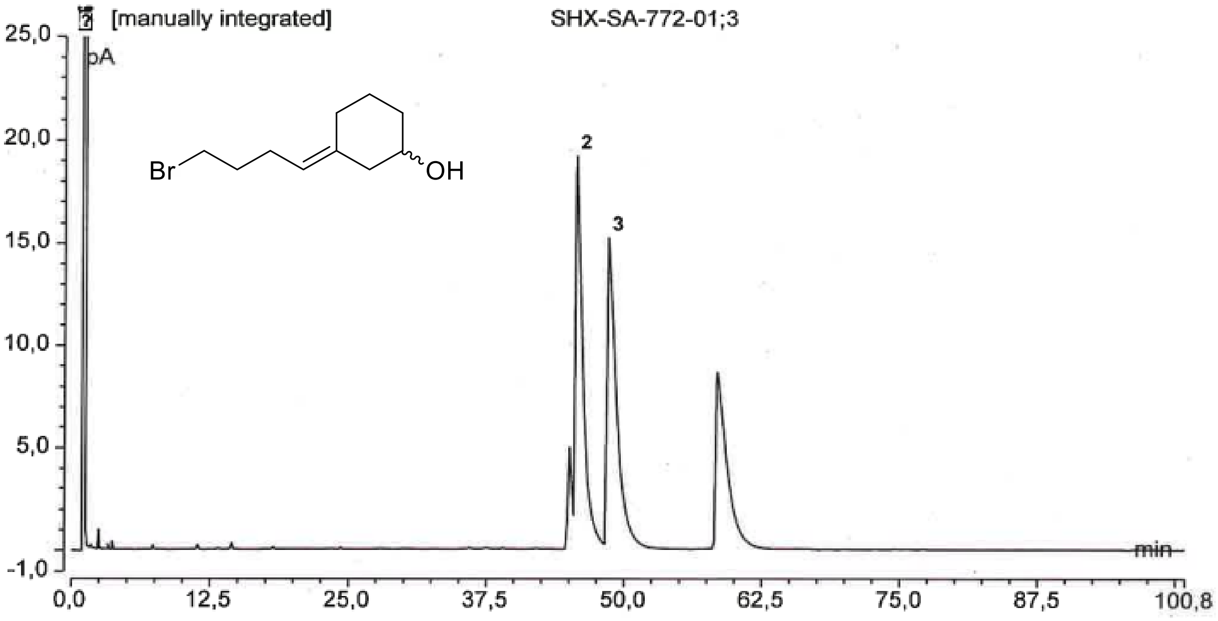

No.	Ret.Time min	Rel.Area %	Peak Name
2	45,57	50,31	.
3	48,48	49,69	.

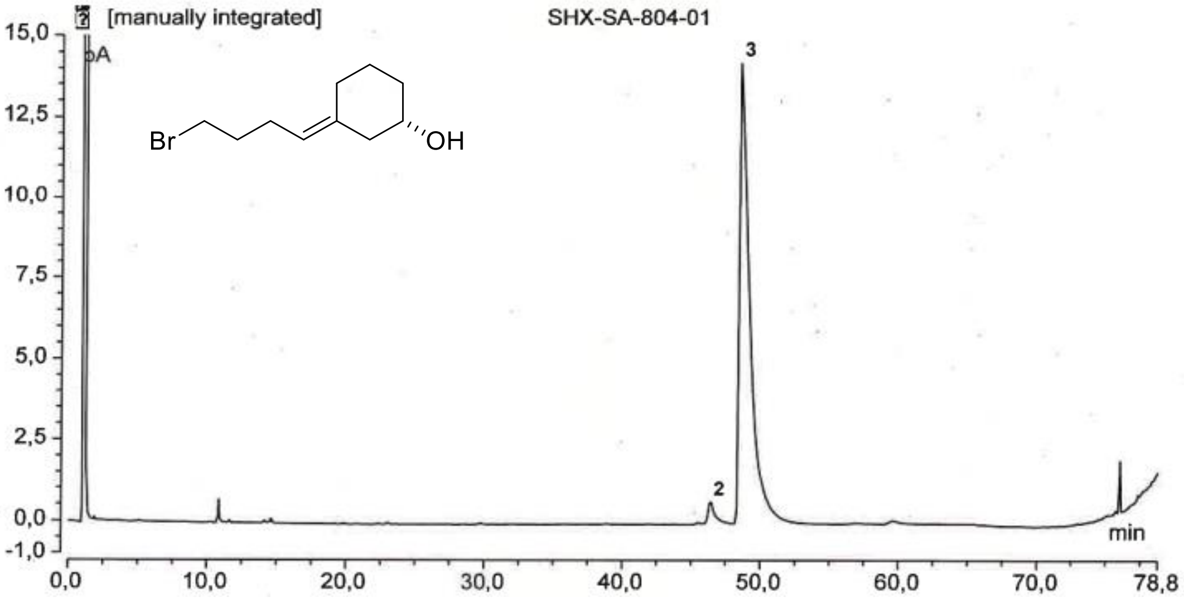

No.	Ret.Time min	Rel.Area %	Peak Name
2	46,41	3,33	.
3	48,48	96,67	.

(*S*)-3-methylenecycloheptan-1-ol (2s)

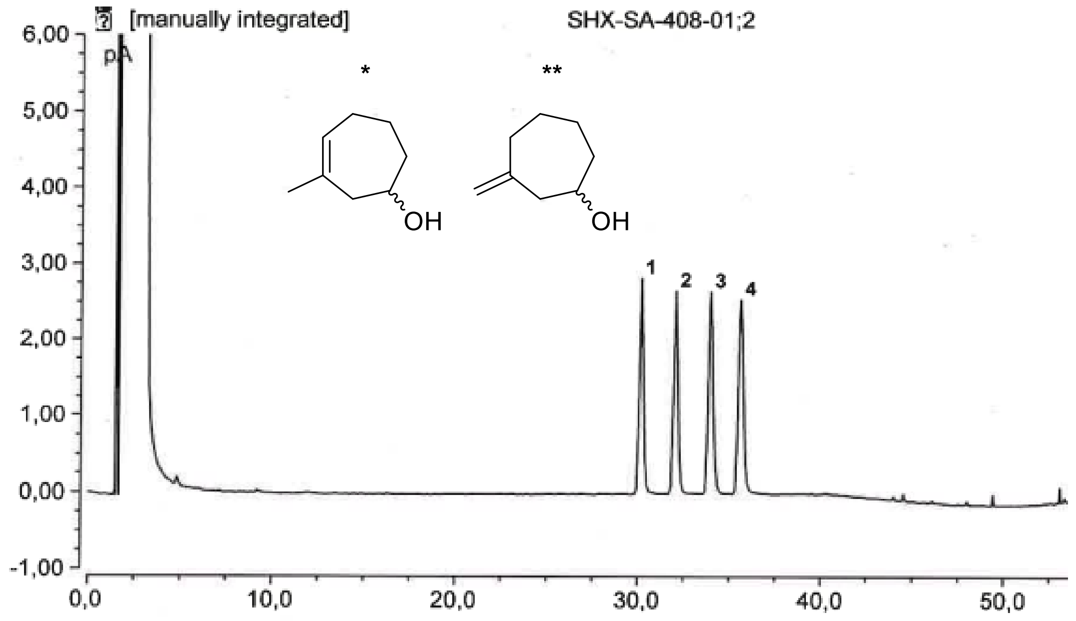

No.	Ret.Time min	Rel.Area %	Peak Name
1	30,14	23,69	*
2	32,01	23,81	*
3	33,91	25,93	**
4	35,58	26,57	**

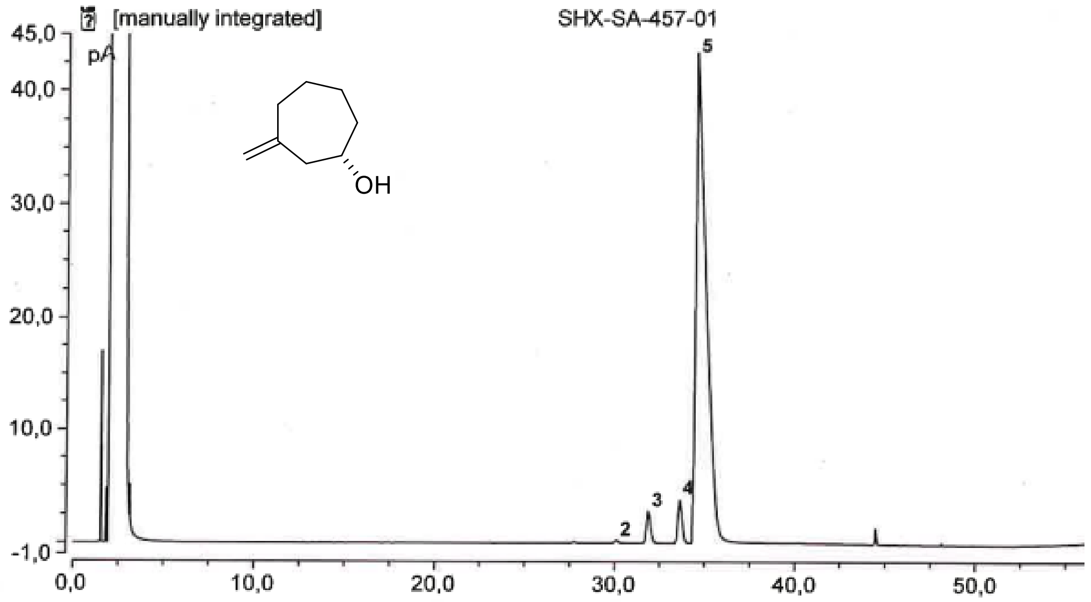

No.	Ret.Time min	Rel.Area %	Peak Name
2	30,10	0,21	*
3	31,85	2,37	*
4	33,58	3,19	**
5	34,40	94,23	**

(*S*,*E*)-3-butylidenecycloheptan-1-ol (2t)

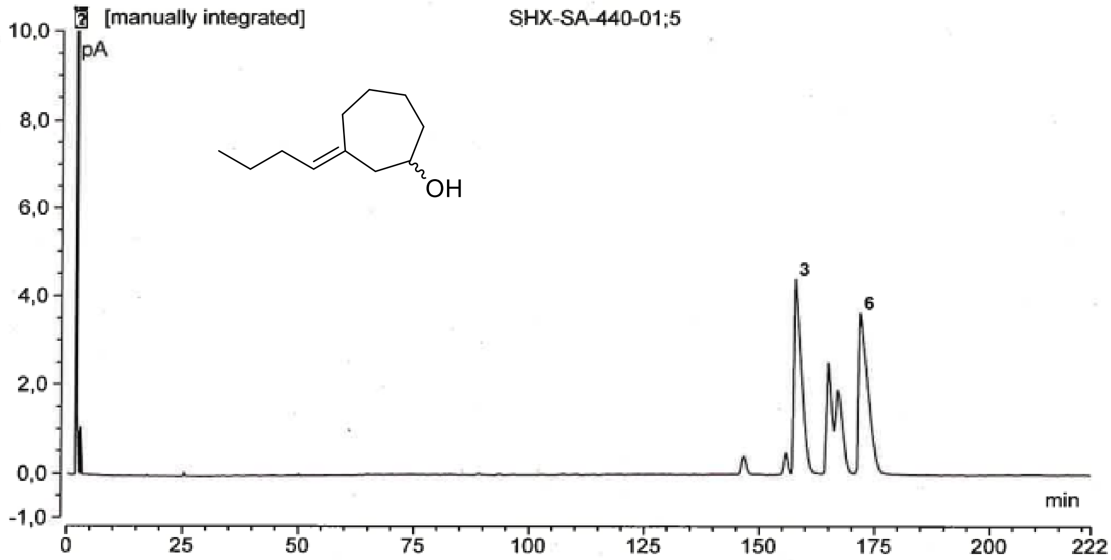

No.	Ret.Time min	Rel.Area %	Peak Name
3	157,67	49,61	
6	171,72	50,39	

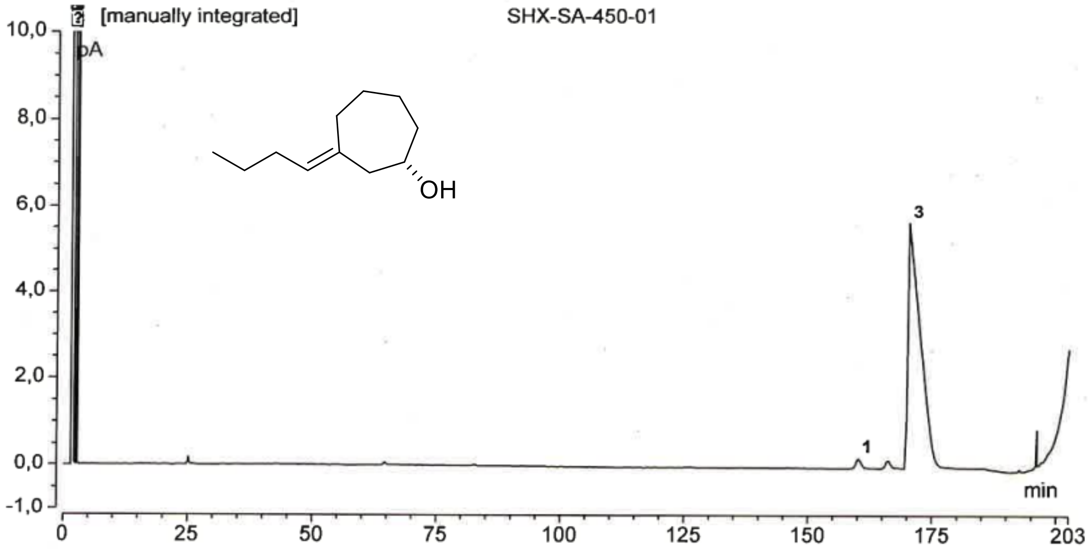

No.	Ret.Time min	Rel.Area %	Peak Name
1	160,11	1,80	
3	170,07	98,20	

(*S*)-3,3-dimethyl-5-methylenecyclohexan-1-ol (2u)

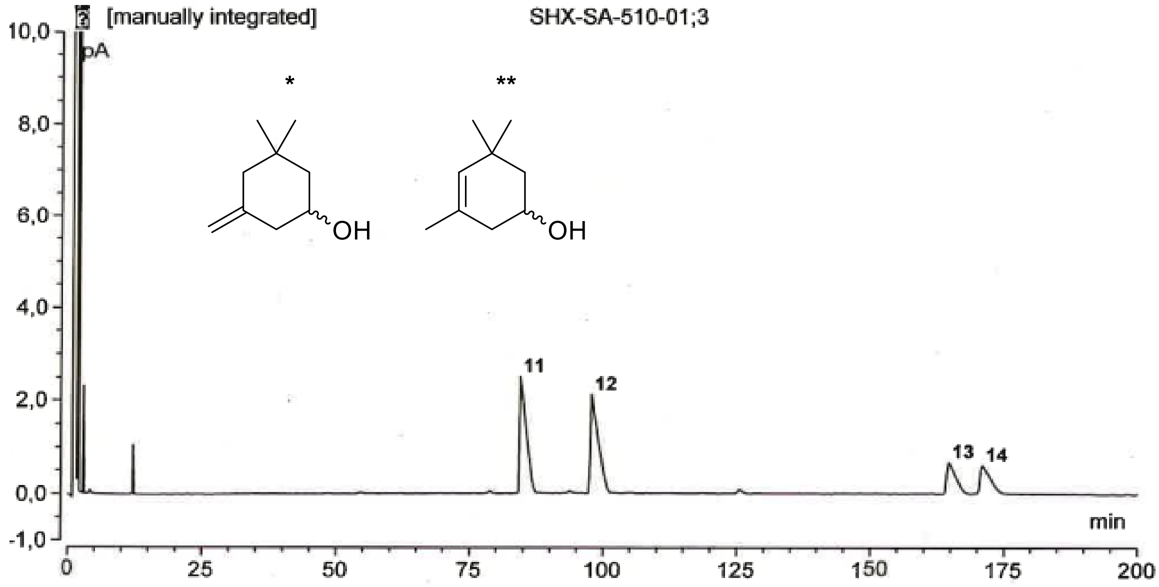

No.	Ret.Time min	Rel.Area %	Peak Name
11	84,41	36,29	*
12	97,74	36,36	*
13	164,65	13,57	**
14	170,91	13,77	**

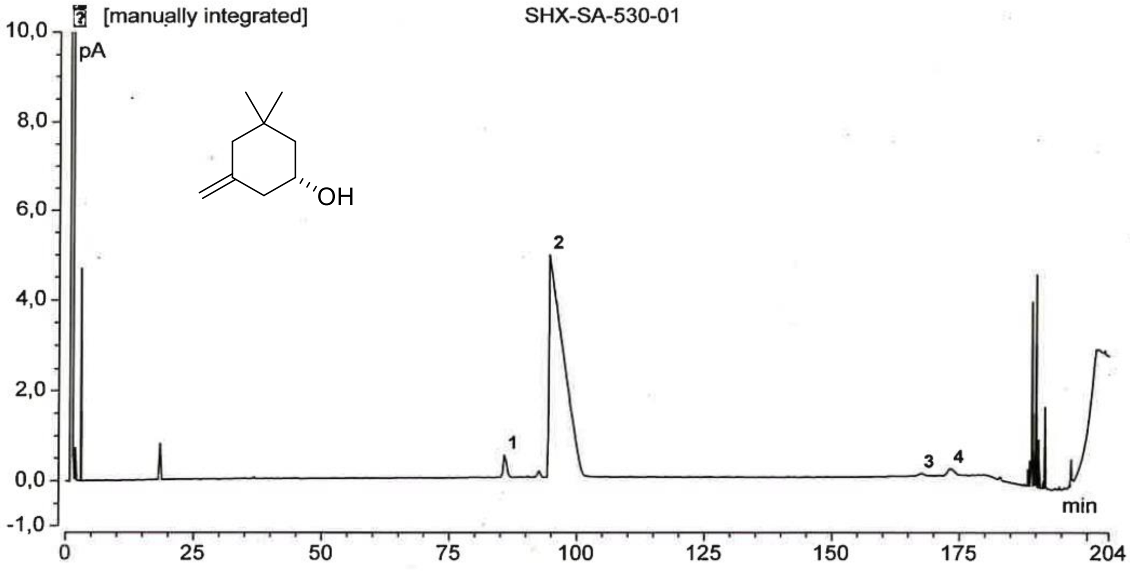

No.	Ret.Time min	Rel.Area %	Peak Name
1	85,74	2,32	*
2	94,45	95,89	*
3	167,29	0,39	**
4	173,10	1,41	**

(*R*)-3-methylene-1,2,3,4-tetrahydronaphthalen-1-ol (2v)

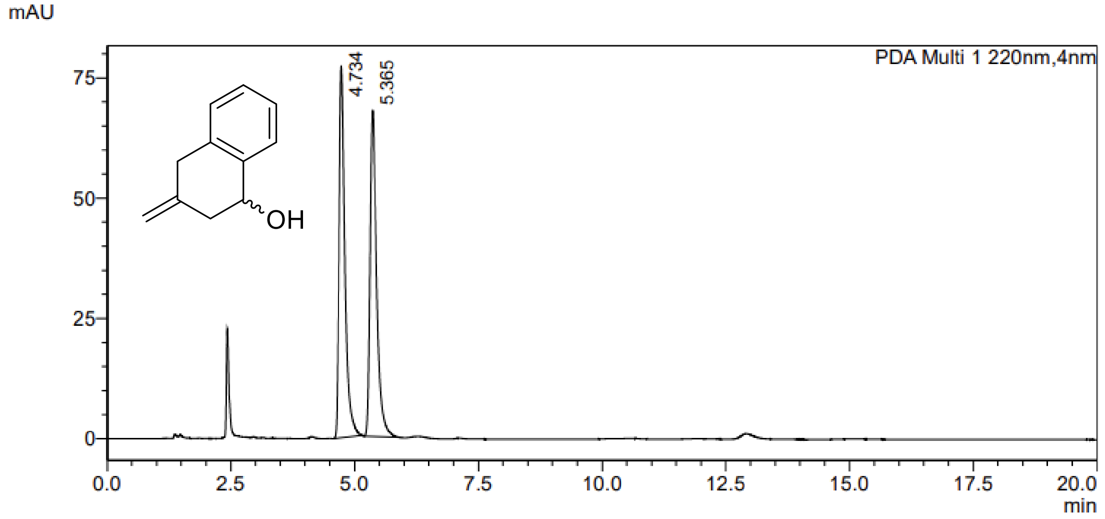

PDA Ch1 220nm

Peak#	Ret. Time	Area%
1	4.734	49.837
2	5.365	50.163
Total		100.000

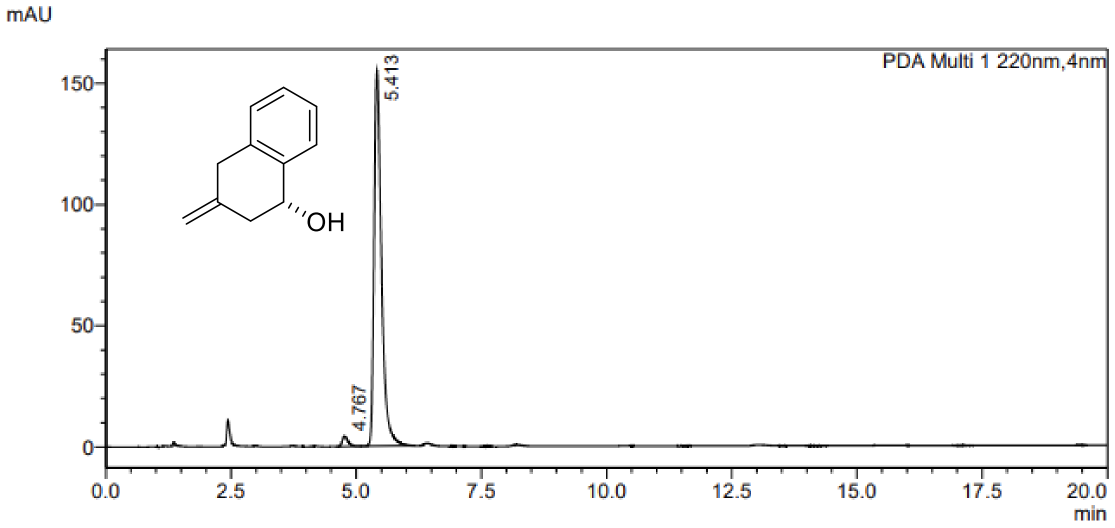

PDA Ch1 220nm

Peak#	Ret. Time	Area%
1	4.767	2.164
2	5.413	97.836
Total		100.000

(*S*)-spiro[2.6]nonan-5-ol (8)

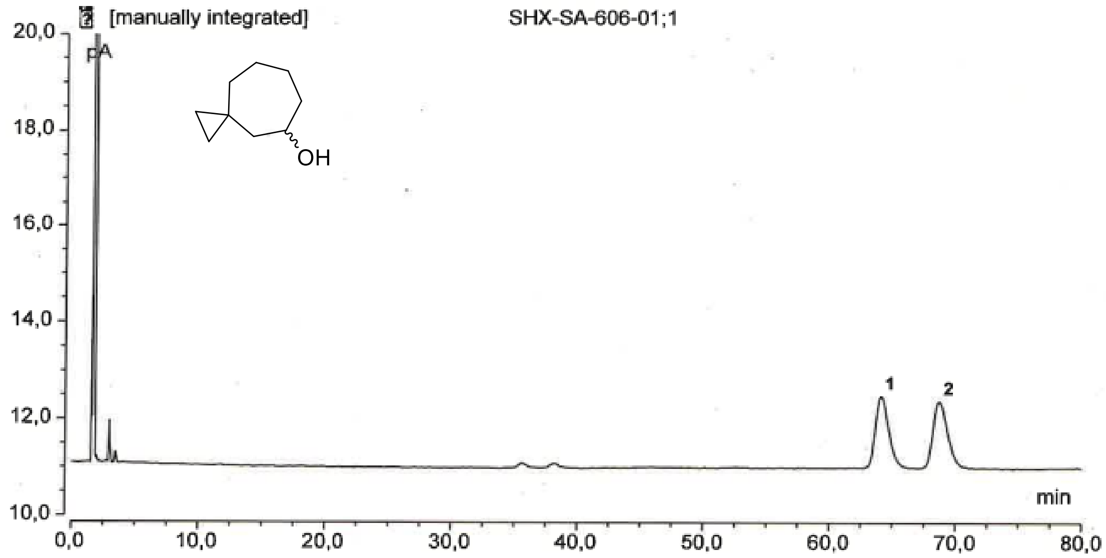

No.	Ret.Time min	Rel.Area %	Peak Name
1	64,09	50,06 *	
2	68,68	49,94 *	

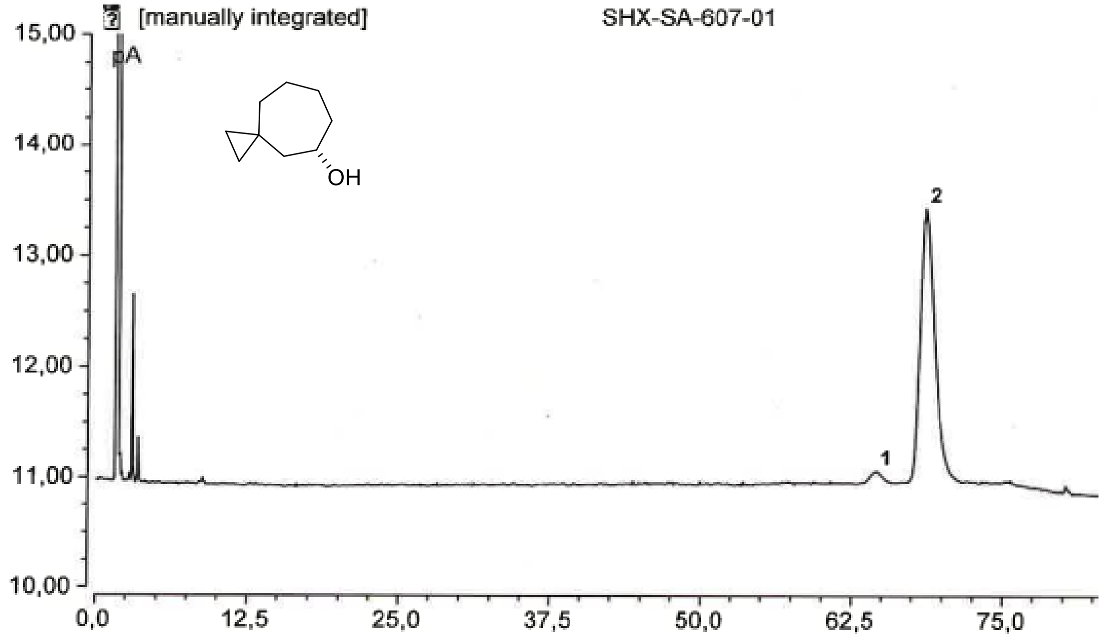

No.	Ret.Time min	Rel.Area %	Peak Name
1	64,51	3,61 *	
2	68,47	96,39 *	

(1*S*,3*S*)-3-butylcycloheptan-1-ol (10)

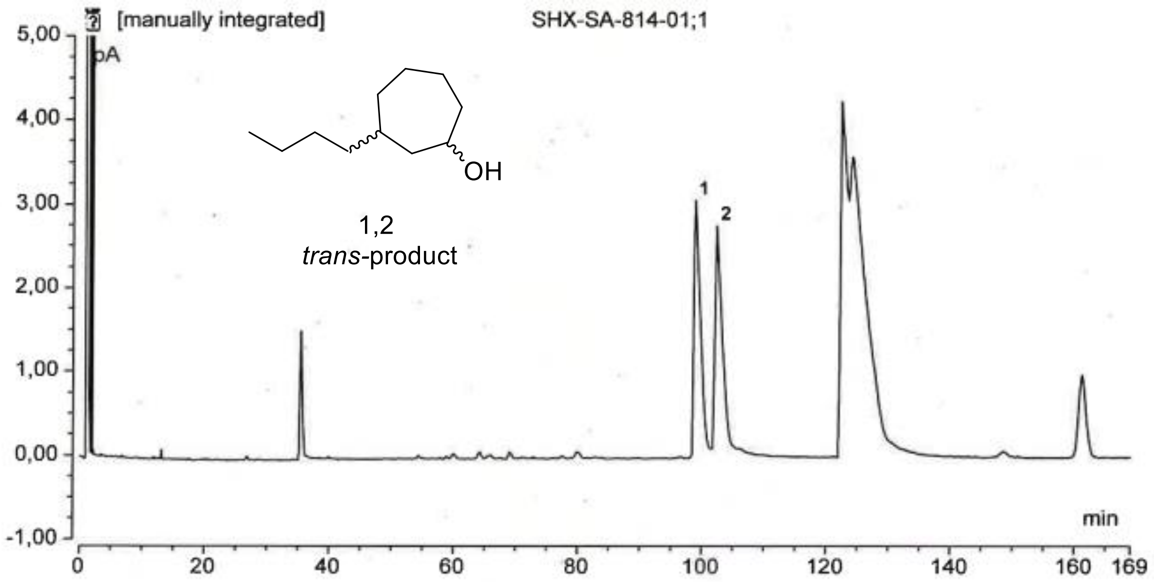

No.	Ret.Time min	Rel.Area %	Peak Name
1	98,82	47,53	.
2	102,28	52,47	.

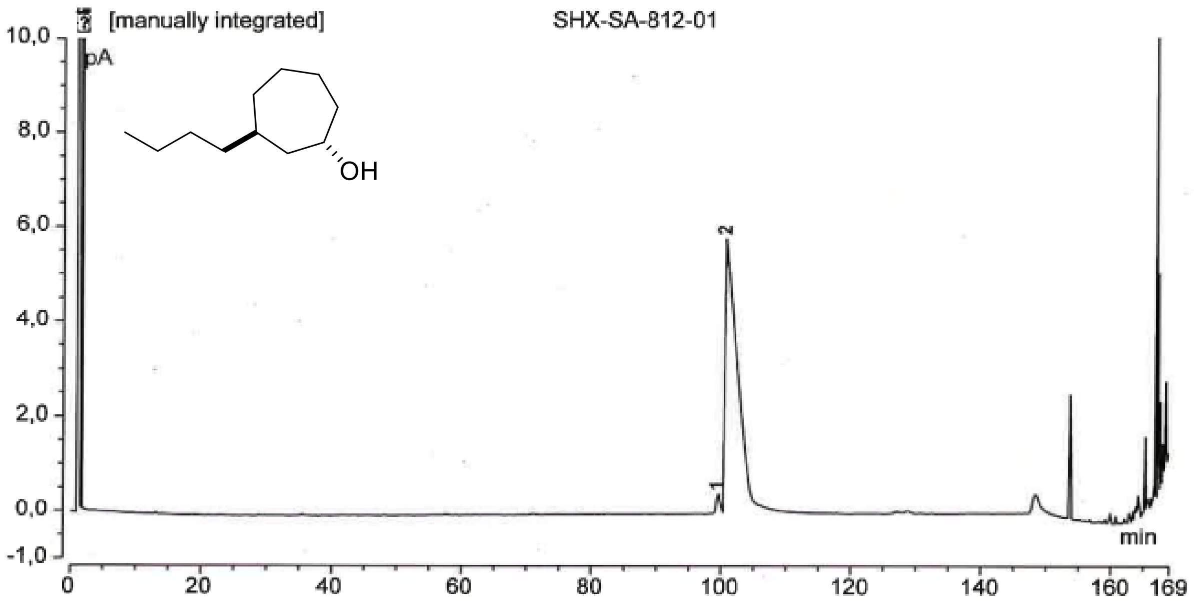

No.	Ret.Time min	Rel.Area %	Peak Name
1	99,58	2,13	.
2	100,70	97,87	.

13. References

(1) Fulmer, G. R.; Miller, A. J. M.; Sherden, N. H.; Gottlieb, H. E.; Nudelman, A.; Stoltz, B. M.; Bercaw, J. E.; Goldberg, K. I., NMR Chemical Shifts of Trace Impurities: Common Laboratory Solvents, Organics, and Gases in Deuterated Solvents Relevant to the Organometallic Chemist. *Organometallics* **2010**, *29* (9), 2176–2179.

(2) Gottlieb, H. E.; Kotlyar, V.; Nudelman, A., NMR Chemical Shifts of Common Laboratory Solvents as Trace Impurities. *J. Org. Chem.* **1997**, *62* (21), 7512–7515.

(3) Kočovský, P.; Ahmed, G.; Šrogl, J.; Malkov, A. V.; Steele, J., New Lewis-Acidic Molybdenum(II) and Tungsten(II) Catalysts for Intramolecular Carbonyl Ene and Prins Reactions. Reversal of the Stereoselectivity of Cyclization of Citronellal. *J. Org. Chem.* **1999**, *64* (8), 2765–2775.

(4) Huang, Z.; Guan, R.; Shanmugam, M.; Bennett, E. L.; Robertson, C. M.; Brookfield, A.; McInnes, E. J. L.; Xiao, J., Oxidative Cleavage of Alkenes by O_2 with a Non-Heme Manganese Catalyst. *J. Am. Chem. Soc.* **2021**, *143* (26), 10005–10013.

(5) Kobayashi, T.; Yamanoue, K.; Abe, H.; Ito, H., Diastereoselective Total Synthesis of (±)-Toxicodenane A. *Eur. J. Org. Chem.* **2017**, *2017* (45), 6693–6699.

(6) Watanabe, H.; Nakajima, K.; Ekuni, K.; Edagawa, R.; Akagi, Y.; Okuda, Y.; Wakamatsu, K.; Orita, A., Custom-Made Pyrene Photocatalyst-Promoted Desulfonylation of Arylethenyl Sulfones Using Green-Light-Emitting Diodes. *Synthesis* **2021**, *53* (17), 2984–2994.

(7) Ley, S. V.; Norman, J.; Pinel, C., Studies Towards the Total Synthesis of Rapamycin: Preparation of the C_{10} C_{17} Carbon Unit. *Tetrahedron Lett.* **1994**, *35* (13), 2095–2098.

(8) Wu, L.; Qiu, S.; Liu, G., Brønsted Base-Modulated Regioselective Pd-Catalyzed Intramolecular Aerobic Oxidative Amination of Alkenes: Formation of Seven-Membered Amides and Evidence for Allylic C–H Activation. *Org. Lett.* **2009**, *11* (12), 2707–2710.

(9) Fernández, D. F.; Gulías, M.; Mascareñas, J. L.; López, F., Iridium(I)-Catalyzed Intramolecular Hydrocarbonation of Alkenes: Efficient Access to Cyclic Systems Bearing Quaternary Stereocenters. *Angew. Chem., Int. Ed.* **2017**, *56* (32), 9541–9545.

(10) Watson, I. D. G.; Ritter, S.; Toste, F. D., Asymmetric Synthesis of Medium-Sized Rings by Intramolecular Au(I)-Catalyzed Cyclopropanation. *J. Am. Chem. Soc.* **2009**, *131* (6), 2056–2057.

(11) Escudero, J.; Bellosta, V.; Cossy, J., Rhodium-Catalyzed Cyclization of *O*,ω-Unsaturated Alkoxyamines: Formation of Oxygen-Containing Heterocycles. *Angew. Chem., Int. Ed.* **2018**, *57* (2), 574–578.

(12) Wübbolt, S.; Cheong, C. B.; Frost, J. R.; Christensen, K. E.; Donohoe, T. J., A Vinyl Cyclopropane Ring Expansion and Iridium-Catalyzed Hydrogen Borrowing Cascade. *Angew. Chem., Int. Ed.* **2020**, *59* (28), 11339–11344.

(13) Zhu, C.; Das, S.; Guin, A.; De, C. K.; List, B., Organocatalytic Regio- and Stereoselective Cyclopropanation of Olefins. *Nat. Catal.* **2025**, *8* (5), 487–494.

(14) Evans, D. A.; Morrissey, M. M.; Dow, R. L., Hydroxyl-Directed Hydrogenation of Homoallylic Alcohols. Effects of Achiral and Chiral Rhodium Catalysts on 1,3 Stereocontrol. *Tetrahedron Lett.* **1985**, *26* (49), 6005–6008.

(15) Bannwarth, C.; Ehlert, S.; Grimme, S., GFN2-xTB–An Accurate and Broadly Parametrized Self-Consistent Tight-Binding Quantum Chemical Method with Multipole Electrostatics and Density-Dependent Dispersion Contributions. *J. Chem. Theory Comput.* **2019**, *15* (3), 1652–1671.

(16) Neese, F., The ORCA Program System. *WIREs Comput. Mol. Sci.* **2012**, *2* (1), 73–78.

(17) Maeda, S.; Harabuchi, Y.; Takagi, M.; Taketsugu, T.; Morokuma, K., Artificial Force Induced Reaction (AFIR) Method for Exploring Quantum Chemical Potential Energy Surfaces. *Chem. Rec.* **2016**, *16* (5), 2232–2248.

(18) Maeda, S.; Ohno, K.; Morokuma, K., Systematic Exploration of the Mechanism of Chemical Reactions: The Global Reaction Route Mapping (GRRM) Strategy Using the ADDF and AFIR Methods. *Phys. Chem. Chem. Phys.* **2013**, *15* (11), 3683–3701.

(19) Grimme, S.; Hansen, A.; Ehlert, S.; Mewes, J.-M., r^2SCAN-3c: A "Swiss Army Knife" Composite Electronic-Structure Method. *J. Chem. Phys.* **2021**, *154* (6), 064103.

(20) Neese, F., Software Update: The ORCA Program System–Version 6.0. *WIREs Comput. Mol. Sci.* **2025**, *15* (2), e70019.

(21) Marenich, A. V.; Cramer, C. J.; Truhlar, D. G., Universal Solvation Model Based on Solute Electron Density and on a Continuum Model of the Solvent Defined by the Bulk Dielectric Constant and Atomic Surface Tensions. *J. Phys. Chem. B* **2009**, *113* (18), 6378–6396.

(22) Mardirossian, N.; Head-Gordon, M., ωB97M-V: A Combinatorially Optimized, Range-Separated Hybrid, meta-GGA Density Functional with VV10 Nonlocal Correlation. *J. Chem. Phys.* **2016**, *144* (21), 214110.

(23) Weigend, F.; Ahlrichs, R., Balanced Basis Sets of Split Valence, Triple Zeta Valence and Quadruple Zeta Valence Quality for H to Rn: Design and Assessment of Accuracy. *Phys. Chem. Chem. Phys.* **2005**, *7* (18), 3297–3305.

(24) Weigend, F., Accurate Coulomb-Fitting Basis Sets for H to Rn. *Phys. Chem. Chem. Phys.* **2006**, *8* (9), 1057-1065.

(25) Meng, E. C.; Goddard, T. D.; Pettersen, E. F.; Couch, G. S.; Pearson, Z. J.; Morris, J. H.; Ferrin, T. E., UCSF ChimeraX: Tools for Structure Building and Analysis. *Protein Sci.* **2023**, *32* (11), e4792.

(26) Blender Online Community. *Blender*, version 4.0; Blender Foundation: Amsterdam, **2025**. https://www.blender.org (accessed 2025-08-13).
